# Electrospray–Mass Spectrometry-Guided Targeted Isolation of Indole Alkaloids from Leaves of *Catharanthus roseus* by Using High-Performance Countercurrent Chromatography

**DOI:** 10.3390/molecules30102115

**Published:** 2025-05-09

**Authors:** Mahdi Yahyazadeh, Dirk Selmar, Gerold Jerz

**Affiliations:** 1 Research Institute of Forests and Rangelands, Agricultural Research, Education and Extension Organization (AREEO), Tehran 1496793612, Iran; yahyazadeh58@yahoo.com; 2 Institute of Plant Biology, TU Braunschweig, Mendelssohnstrasse 4, 38106 Braunschweig, Germany; d.selmar@tu-braunschweig.de; 3 Institute of Food Chemistry, TU Braunschweig, Schleinitzstrasse 20, 38106 Braunschweig, Germany

**Keywords:** *Catharanthus roseus*, indole alkaloid, high-performance countercurrent chromatography, *off-line* injection ESI-MS/MS profiling, 1D/2D NMR

## Abstract

Electrospray mass spectrometry *off-line* profiling monitored the recovery of targeted indole alkaloids from a fortified crude extract of *Catharanthus roseus* (790 mg) using semi-preparative *high-performance countercurrent chromatography* (HPCCC) fractionation. Visualization of selected single-ion traces projected the HPCCC molecular weight elution profile. Experimental *partition-ratio* values *K_D_* and peak widths for detected metabolites were determined. Structural characterization of metabolites and co-elution effects were monitored in the scan range *m*/*z* 100–2000. In this study, the biphasic solvent system containing *n*-hexane–*n*-butanol–water with 0.5% ion-pair reagent trifluoro-acetic acid [1:1:2, *v*/*v*/*v*] was used based on *partition ratio K_D_*-value *liquid chromatography–electrospray ionization–mass spectrometry* (LC-ESI-MS) analysis prediction. The monitoring of target ions resulted in the isolation of six major concentrated indole alkaloids (akuammicine, catharanthine, perivine, vindoline, vindorosine, and *19R*-vindolinine), which were fully elucidated by 1D and 2D *nuclear magnetic resonance* (NMR) spectroscopy.

## 1. Introduction

*Catharanthus roseus* (L.) G. Don. (Madagascar periwinkle) from the family Apocynaceae is one of the best investigated medicinal plants due to the occurrence of a large variety of therapeutically valuable indole alkaloids (IAs). Aerial parts of the *C. roseus* contain between 0.2 and 1% of a mixture of around 130 different alkaloids, whereby the most abundant are monomeric alkaloids such as catharanthine and vindoline alkaloids [1,2,3,4]. The respective dimers of alkaloids are pharmaceutically used as standard anti-cancer chemotherapeutic drugs of the highest activity and result from the joining of two monomeric alkaloids by a C-C covalent linkage. The most well-known dimeric alkaloids, vinblastine and vincristine, are used in chemotherapy of leukemia and the treatment of Hodgkin’s lymphoma [1,2,3]. In addition to the natural dimeric forms of the plant alkaloids, some semi-synthetic derivatives like vinorelbine and vindesine synthesized from vinblastine cure different malignancies. Besides the natural and semi-synthesized dimeric alkaloids, the monomeric alkaloids of the plants containing ajmalicine, serpentine, yohimbine, and vindolicine have medicinal values and are used as anti-hypertensive and anti-neuroinflammatory agents, treatments for erectile dysfunction, and the development of antidiabetic therapeutics, respectively [4]. Due to the medicinal uses of the plant alkaloids and, subsequently, their commercial values, methods for extraction and solid-phase chromatographic purification are well-studied and used on large industrial scales [5].

Since 1970, all-liquid phase separation techniques such as *countercurrent chromatography* (CCC) have widely been employed as versatile and practical methodologies for the separation of natural products and also for lab-scale purifications of various alkaloid types [6,7,8,9]. CCC as a comprehensive all-liquid chromatography method is based on fast compound partitioning steps between immiscible biphasic solvent systems using a liquid mobile and a liquid stationary phase [9,10,11,12]. The separation devices are long polytetrafluorethylene tubes configured as coil columns and rotated in rapidly changing planetary gravitational fields.

The immense advantage of CCC methods is the scalability to transform a small laboratory machine application to a larger process scale [13,14,15], once a biphasic solvent system was optimized. Secondly, all metabolites being prone to fractionation are in a liquid solution and could be recovered without sample loss in the absence of chemisorptive effects, which are fundamental prerequisites for bioassay-guided isolation procedures.

The employed *high-performance countercurrent chromatography* (HPCCC) was operated at a maximum velocity of 1600 rpm, inducing approximately 90,000 phase mixing cycles per hour, leading to a separation depending on *partition-ratio factor* values *K_D_* of metabolites such as the targeted indole alkaloids.

In a previous study on *C. roseus* plant material, *centrifugal partition chromatography* (CPC) was performed by Renault et al. [16,17] to isolate pure indole alkaloids (IAs) by using the *pH-zone refinement* technique, initially developed by Yoichiro Ito [9,18]. This approach allows separating the highest crude extract loads on large laboratory or process-scale equipment, eluted by pH-change variations using acid–base gradients and separated by the compound-specific *pK_a_* values of the respective alkaloids [9,18].

In our HPCCC study on *C. roseus*, we evaluated several classical solvent systems and performed the separation solely using the strong polarity modulating activity of the ion-pair reagent trifluoroacetic acid to improve the separation of IA. To choose the suitable solvent system for this application, *liquid chromatography–electrospray ionization–mass spectrometry* (LC-ESI-MS) prediction analysis was performed to detect the semi-quantitative levels of target IA concentrations in the phase layer in so-called classical shake flask experiments [9]. *Partition ratio K_D_* values from 0.5 to 2.5 are recognized to be suitable for a successful separation [19]. This analytical evaluation approach using mass spectrometry enables the compound-specific detection and determination of *K_D_* values of target compounds. Six biphasic solvent system combinations were evaluated.

In general, CCC methodology is an all-liquid separation technique and completely omits the use of expensive solid-phase materials and, lately, their final disposal. Used solvents could be distilled in an azeotropic way and reconstituted to the original phase compositions by adding missing components to be used in the next CCC-separation batch for a sustainable recovery process.

The semi-preparative HPCCC separation of 790 mg crude IA extract using the *elution*/*extrusion* methodology [20] was monitored by *off-line* injections in the sequence of recovered fractions to an *electrospray–ion trap–mass spectrometer* (ESI-IT-MS/MS). This method enabled the location of target IA in the semi-preparative separation and led to a very exact fractionation process, as co-elution effects were visualized by specific selected ion traces. Further clean-up by classical solid-phase chromatography led to six pure IA for 1D/2D *nuclear magnetic resonance spectroscopy* (NMR) for entire structural elucidation.

## 2. Results and Discussion

### 2.1. Characterization of C. roseus Crude Extract by LC-ESI-MS Analysis

The aqueous TFA-acidified crude extract of *Catharanthus roseus* leaves was submitted to liquid–liquid solvent partitioning steps using a classical acid–base alkaloid extraction scheme [9,16,18]. Chloroform extraction removed residual lipophilic impurities from this crude *C. roseus* extract, and a 790 mg fortified sample of indole alkaloid (IA) contents resulted. LC–ESI–MS/MS analysis in the positive ionization mode of the extract of the aerial parts of *C. roseus* detected a complex mixture of minor and higher concentrated IAs (vindoline, catharanthine, cathavolinine, perivine, vindorosine, akuammicine, ajmalicine, and several uncharacterized components).

### 2.2. Solvent System Selection and K_D_ Prediction of Target IAs on HPCCC Using LC-ESI-MS

**Figure 1:**
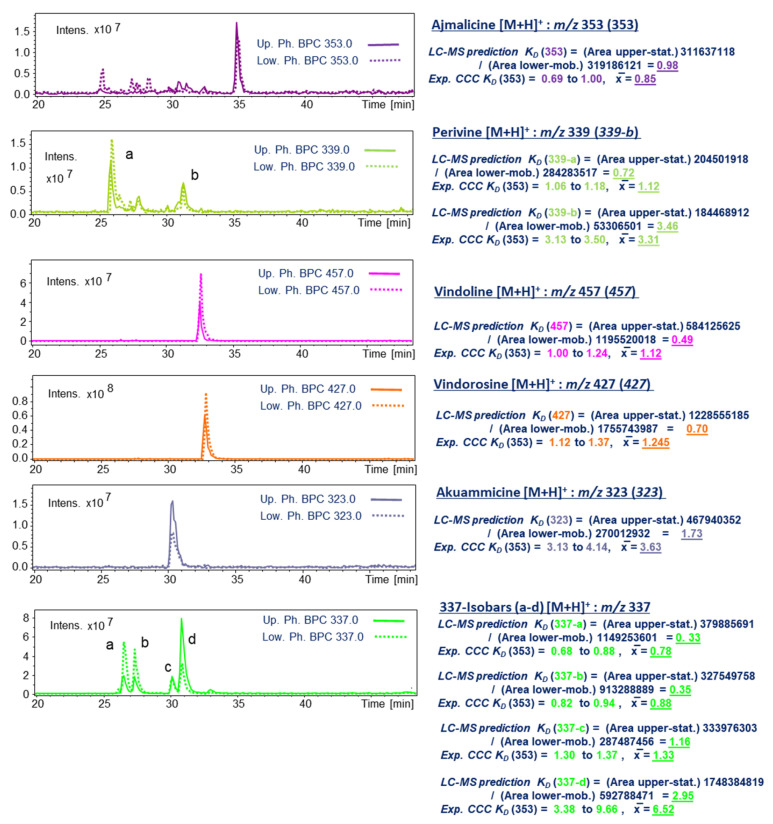
HPCCC solvent system evaluation of *SoSy* 1 (*n*-hexane/*n*-butanol/aqueous 0.5% TFA) by LC-ESI-MS (pos. ion mode) for principal IAs of *C. roseus. Bold line:* compound concentration in upper (stationary) phase; *dashed line:* concentration in lower (mobile) phase. Monitoring of selected single ion traces of ajmalicine (*m/z* 353), perivine isobars **339-a/b** (*m/z* 339), vindoline (*m/z* 457,) vindorosine (*m/z* 427), akuammicine (*m/z* 323), and molecular weight isobars **337-a** to **337-d**.

The fortified *C. roseus* extract in IA content (10 mg) was dissolved for each shake-flask experiment [9] in the six selected biphasic solvent systems (*SoSy*) 1–6 for LC-ESI-MS evaluation of the target concentrations in phase layers. The determined area values from selected ion traces were used to determine the *K_D_* values for prediction of HPCCC (cf. Figure 1). The workflow of this evaluation is described in Section 3.4.

Solvent system (*SoSy*) 2–6 from different solvent system families were seen as not suitable for alkaloid extract (cf. Supplementary Figure S1), as the required *K_D_*-value ranges (0.5–2.5) were not reached, and the IAs were distributed dominantly either to the upper or lower phase layer [9,11,19].

*SoSy1* (*n*-hexane–*n*-butanol–water, 1:1:2, *v*/*v*/*v* and addition of 5.0 mL/L TFA) was interpreted as the only suitable solvent system with *K_D_* values between 0.5 and 2.5 [19] (Figure 1). The bold printed lines displayed the selected LC-ESI-MS ion traces representing in all measurements the respective target compound intensities in the *upper* organic phase layer, and the dashed line resulted in the determined areas in the *lower,* more aqueous phase layer, respectively.

Ajmalicine with the ion trace [M + H]^+^ at *m*/*z* 353 resulted in a *K_D_* value of 0.98; lower concentrated isobars were not taken into account, but the peak distribution of all these signals suggested an earlier elution on HPCCC. As for these minors, the observed area values in the lower (mobile) phase layer appeared to be of higher intensity (using *head-to-tail* operation mode). The conducted HPCCC experiment resulted in a matching *K_D_* range (0.69–1.00) for the recovered preparative peak signal of ajmalicine (cf. Figure 1).

Perivine (**339-a**) ([M + H]^+^; *m*/*z* 339) and one lower concentrated isomer (**339-b**) gave values of 0.72 and 3.46, respectively. Indeed, **339-a** was detected in HPCCC in the *K_D_* 1.06–1.18 range with an *offset* of ∆*K_D_* 0.4 from the LC-MS prediction. Furthermore, **339-b** was detected similar to the prediction in the *K_D_* range 3.13–3.50 (cf. Figure 1).

A single peak for vindoline [M + H]^+^ (*m*/*z* 457) suggested, with *K_D_* 0.49, a more polar elution behavior and therefore earlier elution from the HPCCC device in the ‘*head-to-tail*’ mode. Not in accordance with the LC-ESI-MS prediction, HPCCC delivered **457** much later at *K_D_* 1.00–1.24 (cf. Figure 1).

**Figure 2:**
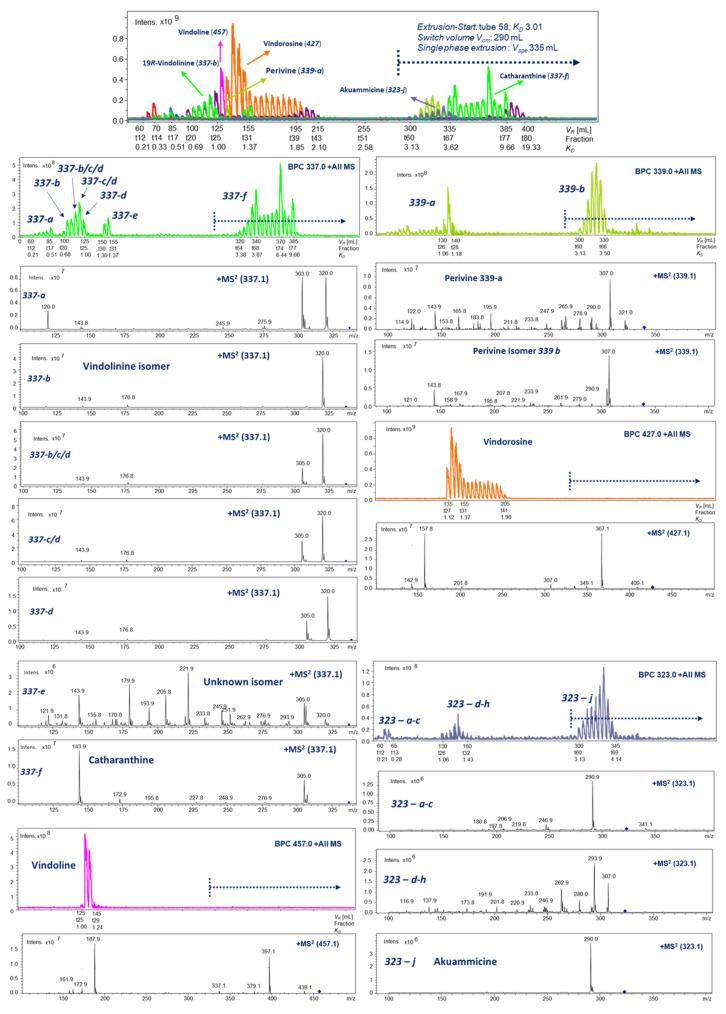
HPCCC *off-line* ESI-MS/MS injection profiling (*m*/*z* 100–2000) in the positive ionization mode of indole alkaloids from *C. roseus*. HPCCC chromatography scale displayed in *K_D_* values, collected tube fractions, and *retention volume V_R_*. The blue doted line in the MS-profile indicate the end of *elution* process and start of the *extrusion*-mode in the HPCCC experiment at switch volume V_CM_ (290 mL). The blue arrows indicate the specific positions of close eluting **337**-isobars in the HPCCC fractions.

The similar retention time as vindoline on C_18_-LC displayed vindorosine (*m*/*z* 427), but the *K_D_* value (0.70) was different and suggested a successful separation. However, in the HPCC experiment, vindorosine eluted in the *K_D_* range of 1.12–1.37, sufficiently separated from vindoline (Figure 2).

The selected ion trace for akuammicine [M + H]^+^ (*m*/*z* 323) at LC-Rt 30.5 min suggested a more polar character, but the *K_D_*-value prediction was 1.73, much higher than for vindoline and vindorosine. This occurrence suggested an unexpectedly higher lipophilicity on HPCCC and led to a very surprisingly high *K_D_* value of 3.13–4.14 in the semi-preparative experiment (recovery from *extrusion* mode) [20] (Figure 1 and Figure 2).

The selected ion trace at *m*/*z* 337 detected four major peaks on C_18_-LC with different retention times potentially related to *19R*-vindolinine and catharanthine. Two isobars (**337-a** and **337-b**) displayed lower *K_D_* values of 0.33 and 0.35 and suggested early elution from HPCCC. The metabolite **337-c** was in the optimal range, and **337-d** with *K_D_* 2.95 was predicted for recovery in the *extrusion* mode. Both 337-isobars *19R*-vidolinine and catharanthine were recovered from HPCCC at *K_D_* 0.68–1.00 (*elution* mode) and 3.38–6.44 (*extrusion* mode), respectively.

Currently, the solvent system prediction by LC-MS is the experimental method of choice, as the *partition ratio values K_D_* of target compounds could be specifically determined by their relative concentrations in the biphasic solvent layers using the respective molecular weight data. This method is not always resulting in absolute matching to the CCC experiment, as the used extract amounts (e.g., 10 mg/2 mL) in the pre-evaluation are not reflecting the real situation of a semi-CCC separation with used concentrations of >500 mg/approx. 100 mL *stationary phase volume* (*V_S_*). Therefore, certain intermolecular effects could be initiated under higher concentrations, such as hydrogen-bond formations, e.g., between carboxylic acid functionalities, and as this sort of dimerization leads to a significant masking of the polar compound character, finally shifting the *retention volumes V_R_* on CCC.

Also, the existing TFA concentration during the CCC experiment and the ion-pair interaction with IAs are difficult to evaluate by predictive experiments. The above-mentioned matrix-background effects might be responsible for the observed *offsets* of LC-MS predicted to the ‘*real*’ CCC-experimental *K_D_* values of some certain IAs of this investigation.

### 2.3. HPCCC Off-Line ESI-MS/MS Injection Profiles

The target monitoring of indole alkaloids (IAs) in *C. roseus* extract by hyphenation of semi-preparative HPCCC and *off-line* ESI-MS/MS injection profiling guided the fractionation process with high accuracy. Unintentional pooling of fractions with potentially separated metabolites will be omitted at an early stage as a direct view of the compositions of fractions is implemented. During the MS profiling, up to 10 precursor ions could be selected in an auto-MS/MS fragmentation process and already provided an extended data set for the characterization of co-eluting compounds. Therefore, tentative structural characterization of minor and potentially novel derivatives is of the greatest benefit in the isolation workflow [21,22,23,24].

However, a full structural characterization by 1D/2D NMR of target IA molecules required two further preparative fining steps by solid-phase column chromatography (cf. Supplementary Figure S10).

Even-numbered quasi-molecular ion signals [M + H]^+^ detected IAs in the positive ionization mode. As monitored by selected ion traces (Figure 2), the wide chromatographic distribution of separated IAs covered the complete *elution* and *extrusion* chapters of the HPCCC experiment [20].

The principal component, vindorosine (**427**), was seen with a rather strong tailing effect over a large *K_D_*-value range (1.12–1.98), whereby vindoline (**457**) co-eluted quite sharply in the range of some fractions (*K_D_* 1.00–1.24). The partial co-elution of vindoline with small interference into the high-intensity peak of vindorosine was not a problematic case. By use of the *off-line* ESI-MS profiling, the fractions in the HPCCC were specifically selected for the two target IAs, and solely pure vindoline and vindorosine were pooled for further clean-up steps and final 1D/2D NMR analysis. The tailing effect of vindorosine could be suspected to be related to a lack of TFA-ion-pair reagent at this stage of HPCCC due to a local high IA concentration.

In a very efficient way, so-called molecular weight isobars of IAs were fractionated, such as isomers of *m*/*z* 337, *m*/*z* 339, or *m*/*z* 323.

In the case of the selected target ion trace *m*/*z* 337, six recovery sections (**337-a**–**337-f**) were defined by a *retention volume V_R_* or *K_D_* value and characterized by respective MS/MS fragmentation data (Figure 2). Vindolinine (**337-b**) and catharanthine (**337-f**) were finally fully elucidated by NMR experiments (cf. Section 2.5.6 and Section 2.5.3). The very different recovery ranges of the pair vindolinine (**337-b**) and catharanthine (**337-f**) were surprising, as both IAs carry as polar functional groups a single methyl-ester function.

The monitoring resulted for *m*/*z* 339 in one early *elution*-mode section (*K_D_* 1.06–1.18) and in late in the *extrusion* (*K_D_* 3.13–3.50), respectively. Moreover, **339-a** was clearly identified as perivine (cf. Section 2.5.4).

Observation of target ions by *off-line* ESI-MS obviously revealed the existing co-elution effects (Figure 2). The recovery sequence of IAs from HPCCC (*head-to-tail* = *reversed-phase* mode) (Table 1) is certainly based on the substitution pattern influenced by functional groups. In Table 1, the sequence of HPCCC elution is given from IA1 to IA6. The presented functional groups do not reflect the experimentally detected order from *elution/extrusion* mode. Therefore, we assumed that specific stereochemical aspects of hindering and restricted access of the deprotonated ion-pair reagent trifluoroacetic acid anion (TFA) to some protonated and positively charged nitrogen positions need to be taken into account. The order of elution might also be influenced by intermolecular hydrogen-bonding effects and masking of polar functionalities (cf. Section 2.2). The free amino-functional group seemed to be of rather low influence to decrease the real lipophilic character of IAs (catharanthine and akuammicine) being recovered from HPCCC in the *extrusion* mode.

The first IA recovered with larger amounts from HPCCC was polar *19R*-vindolinine (**337-b**) (Table 1), carrying one free amino function and one methyl-ester functionality, followed by vindoline (**457**) with lipophilic substitutions (-N-CH_3_ and -CH_2_-CH_3_), but strongly compensated by a polar hydroxy function. Slightly more lipophilic perivine (**339-a**) eluted sharply in the next tube (*K_D_* 1.06–1.18), showing the same substitution pattern as **337-b** but presenting the ethyl-side chain function. Vindorosine (**427**) was the fourth IA in the elution sequence, and surprising was the lipophilic strength of the additional methoxy group compared to **457**. However, akuammicine (**323-j**) with an identical substitution pattern to **337-b** was recovered at high *K_D_* values from the lipophilic *extrusion* section. In catharanthine (**337-f**), a methyl-ester function was exchanged for a less polar carbonyl function. Overall, the free polar amino group seemed to have a low impact on decreasing lipophilicity, as already seen for **323-j**.

**Figure 3:**
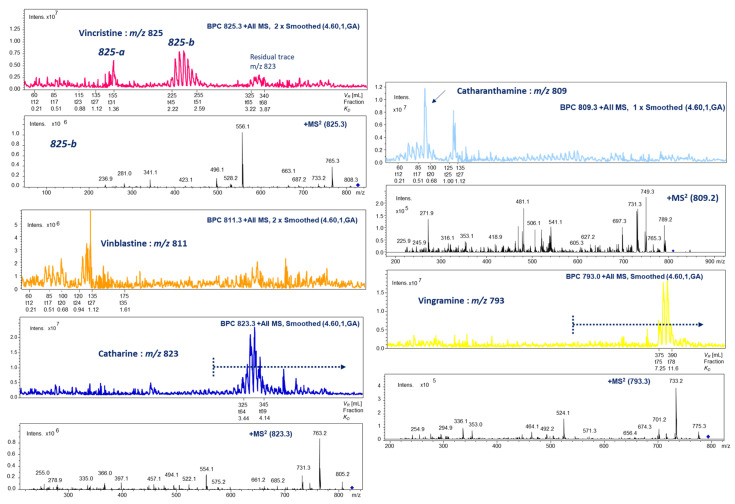
HPCCC *off-line* ESI-MS/MS injection profiling (*m*/*z* 100–2000) of low-concentrated indole alkaloid dimers (positive ionization mode): vincristine, vinblastine (low intensity, no MS/MS), catharine, catharanthamine, and vingramine. The blue doted line indicate the start of the *extrusion*-mode—*switch volume V_CM_* (290 mL) with late detection of IA-dimers *m/z* 823 and *m/z* 793 in the HPCCC experiment.

Minor concentrated dimers of IAs (vincristine: *m*/*z* 825, vinblastine: *m*/*z* 811, catharine: *m*/*z* 823, catharanthamine: *m*/*z* 809, and vingramine: *m*/*z* 793) showing different chromatographic polarities on HPCCC were monitored by *off-line* ESI-MS/MS profiling. The relative concentration in fractions after HPCCC fortification appeared to be very low, assessed by detected ion counts, and finally preparative scale isolations failed. Important cytostatic drugs are the IA dimers vincristine and vinblastine, which are part of modern anti-tumor therapy (Figure 3).

### 2.4. LC-ESI-MS of Selected HPCCC Fractions for Molecular Weight Isobar Monitoring

**Figure 4:**
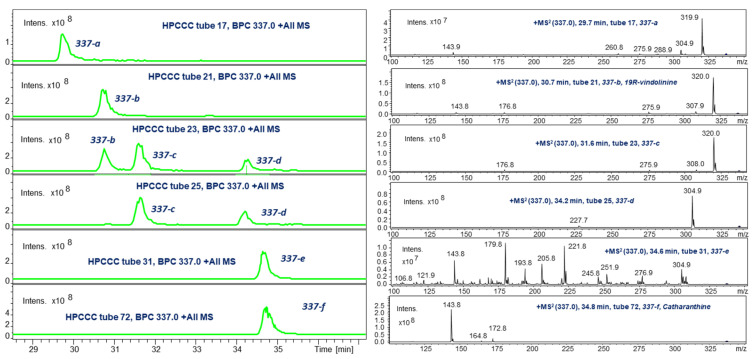
LC-ESI-MS monitoring of *m*/*z* 337 isobars in HPCCC fractions.

**Figure 5:**
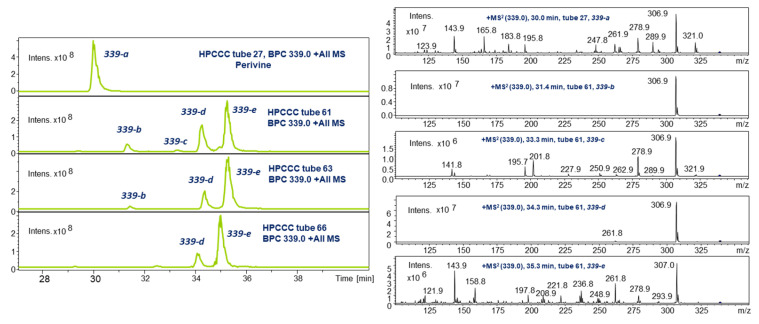
LC-ESI-MS monitoring of *m*/*z* 339 isobars in HPCCC fractions.

**Figure 6:**
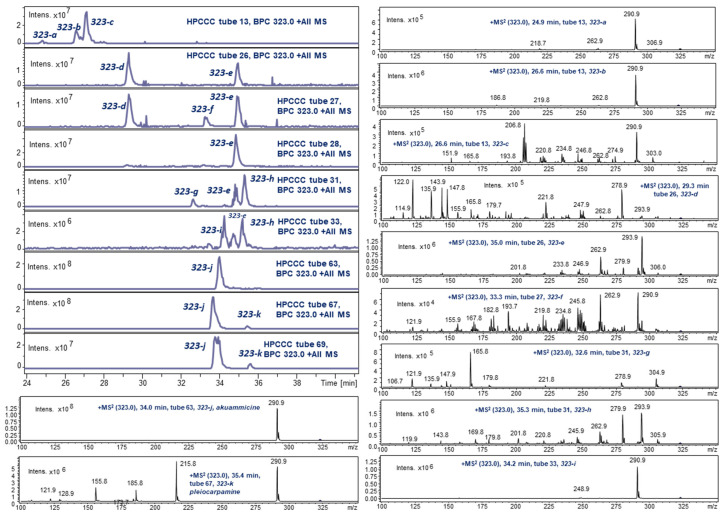
LC-ESI-MS monitoring of *m*/*z* 323 isobars in HPCCC fractions.

A 2D chromatographic approach on HPCCC-separated fractions using C_18_-LC-ESI-MS of some selected fractions revealed a much more complex picture of the existing IA isobars (Figure 4, Figure 5 and Figure 6). This re-chromatographic approach provided additional ESI-MS sensitivity to the detection limits with a deeper view to the diversity of isobars with *m*/*z* 337, *m*/*z* 339, and *m*/*z* 323. These target HPCCC fractions were selected on the data basis of the *off-line* ESI-MS injection profiling experiment (Figure 2).

In the case of *m*/*z* 337, the LC-ESI-MS (Figure 4) detected in the *K_D_* range (0.2–0.5, t17) a single compound (Rt 29.8 min, **337-a**) with *m*/*z* 320 as the major MS/MS fragment. Already t21 in the range (*K_D_* 0.68–1.00) contained the completely separated **337-b** (Rt 30.8 min). The contents of t23 revealed two more isobars, whereby the set **337-a**/**337-b**/**337-c** displayed an absolutely identical MS/MS pattern (Figure 4). Moreover, **337-d** (Rt 34.2 min) resulted in an MS/MS base peak at *m*/*z* 305, whereby the HPCCC *K_D_* range (1.30–1.37, t31) detected pure isobar **337-e** of identical C_18_-LC retention time to **337-f**. This last eluting IA was recovered as one of the most lipophilic compounds in *extrusion* mode. The MS/MS of **337-e** appeared to be very complex and characteristic for this metabolite and seemed, with the ion *m*/*z* 144, to be connected to catharanthine **337-f,** showing this respective base peak (cf. Section 2.5.3).

In the isobar monitoring of *m*/*z* 339 in the *K_D_* range (1.06–1.18, t27) of the *elution* mode (cf. Figure 2), a single peak was detected (30.0 min, **339-a**) with a highly complex MS/MS fragment pattern. The higher abundant and potentially more concentrated recovery sections in the *extrusion* mode (*K_D_* 3.13–3.50) of the ESI-MS injection profile (Figure 2) were detected in tube fraction 61, four isobars (**339-b** to **339-e**) (Figure 5), and showed in the analysis of fraction t66 in the declining slope of the HPCCC peak as the most abundant signal for **339-e**, later NMR elucidated as perivine (cf. Section 2.5.4). Interestingly, isobar LC-ESI-MS/MS spectral data of **339-a** and **339-e** (cf. Figure 5) were quite similar, although the polarity-based recovery sections from HPCCC were completely different. All isobars displayed the base peak signal *m*/*z* 307 in the MS/MS experiment.

The ESI-MS *off-line* injection profile detected three principal sections of different polarities for the selected ion trace *m*/*z* 323 (*K_D_* 0.21–0.28; 1.06–1.43; 3.13–4.14) (Figure 6).

The 2D approach with LC-ESI-MS re-analysis separated and revealed the highest diversity of isobars for *m*/*z* 323 isobars. In the MS/MS, **323-a**, **b**, **c**, **f**, **i**, **j**, and **k** displayed a strong fragment signal for *m*/*z* 291. After preparative column chromatography, purified **323-j** was elucidated by 1D/2D NMR as akuammicine (cf. Section 2.5.5).

### 2.5. HPCCC Separation and 1D/2D NMR Structure Elucidation of Indole Alkaloids (IAs)

#### 2.5.1. Vindorosine (427): (Syn. Demethyoxy-Vindoline, Vindolidine); [M + H]^+^: m/z 427

One of the principal indole alkaloids, vindorosine, was detected by a high signal intensity for the selected ion trace at *m*/*z* 427 in the *off-line* ESI-MS/MS HPCCC experiment (cf. Figure 2) of the fractions in the *elution* mode (*K_D_* 1.12–1.98, fractions t27-t42). Starting at the *K_D_* range of 1.37 (t31), the peak intensities from the MS profile indicated a strong tailing effect in the HPCCC. However, the LC-ESI-MS analysis of the respective fractions containing *m*/*z* 427 proved the existence of solely vindorosine and no isobars over this detected whole HPCCC *elution* range.

**Figure 7:**
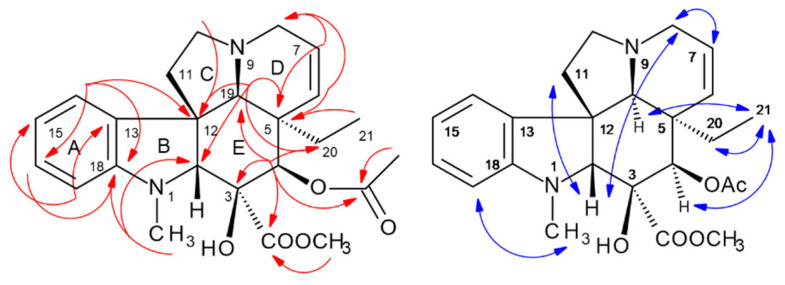
^2,3^*J*-HC long-range correlations (HMBC) (red color), and ^1^H/^1^H-NOESY of vindorosine (**427**) (blue).

As the *off-line* MS injection profile (Figure 2) suggested a high purity of vindorosine, e.g., in fraction t28, 41 mg of it were recovered and directly used for 1D/2D NMR spectroscopic experiments. ^13^C-NMR spectra in CD_3_OD and also in C_6_D_6_ detected twenty-four resonances, including thirteen CH/CH_3_, four CH_2_, and seven quaternary signals (confirmed by DEPT135, phase-edited HSQC) (cf. Supplementary Table S1). In the HMBC, key long-range cross signals (cf. Figure 7, Supplementary NMR Figures S5 and S6) of protons in central structural positions, such as H-2 (*δ* 3.73 ppm), H-4 (*δ* 5.80), H-19 (*δ* 2.62 ppm), H-14 (*δ* 6.71 ppm), and H-17 (*δ* 6.21 ppm), elucidated the fusion and constitution of ring systems (A-E) of vindorosine (cf. Figure 7). The advantage of measuring in C_6_D_6_ was that H-4 (*δ* 5.80 ppm) and H-6 (5.12 ppm) did not overlap as in CD_3_OD (cf. Supplementary Table S1) and enabled a clear HMBC observation. Specific carbon resonances compared to literature data in C_6_D_6_ appeared to be conspicuous (for comparison reasons, the numbering system of Ishikawa et al. [25,26] and displayed ^13^C-*δ*-shift *off*-sets (∆ppm: +/− 1.8–2.8 ppm). However, a NOESY experiment confirmed the relative configurations of chiral centers in the isolated vindorosine, and the chiral centers were in absolute accordance with the methoxy-derivate vindoline (cf. Figure 7).

NOE effect sequences (*seq*.) were observed from the aromatic H-14 (*δ* 7.36 ppm). *Seq. 1* (*upper*): led to H-15 over 16/17 → >N-CH_3_ → H-2 cross-over to H-11a → H-14.

**Figure 8:**
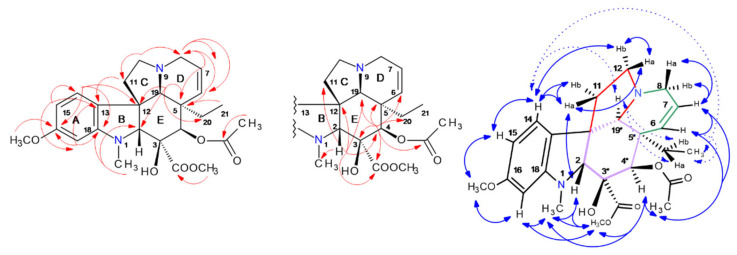
^2,3^*J*-HC long-range correlations (HMBC) (red color), and ^1^H/^1^H-NOESY of vindoline (**457**) (blue) with *upper plane* in bold arrows. Asterix * for chiral centers.

The NOE-*seq. 2*, 3, and 4 were identical to the effects observed for vindoline (cf. below vindoline, Supplementary Table S1, Figure 8). All the observed through-space effects corroborated the known stereochemistry of vindorosine (cf. Supplementary Table S1, Supplementary NMR Figure S5). Potentially, observed *δ*-ppm value *off*sets could be correlated to differing concentrations and pH values of NMR probe solutions.

#### 2.5.2. Vindoline (**457**): (Syn. Methoxy-Vindorosine); [M + H]^+^: m/z 457

Vindoline (**457**) was detected in the *off-line* ESI-MS profiling of HPCCC fractions in a rather narrow elution window (t25–t27, *K_D_* 1.00–1.24) (cf. Figure 2) using the selected ion trace for [M + H]^+^ at *m*/*z* 457.

The ESI-MS/MS from the MS-profile experiment displayed two strong fragment ions at *m*/*z* 397 and 188 (cf. Figure 2), consistent with analysis of a commercial reference and literature data [27].

Vindoline (**457**) is a methoxy derivative of the IA vindorosine (**427**) [4] (Figure 8), and for better comparison, the numbering system of Ishikawa [25] was used for **IA-427**. **IA-457** displayed in the *head-to-tail* mode a lower *retention volume* than **IA-427**, explained by its approximate more polar character. The part of co-elution in t27 with vindorosine (Figure 2) was not problematic, as most of **IA-427** was eluted in later fractions. Although a quite low *selectivity factor* value was determined (α 1.38) from the HPCCC experiment, this chromatography situation was not problematic, as the major concentrated IA-427 with strong tailing was the second eluting metabolite. Retention time values from LC-ESI-MS indicated that vindoline (Rt 36.5 min) and vindorosine (Rt 36.8 min) would be a highly problematic IA pair to be separated by preparative C_18_-LC.

HPCCC fractions t26–27 were cleaned by preparative C_18_-HPLC to remove poly-disperse background material, and then **IA-457** was used for 1D/2D NMR (cf. Supplementary NMR Figure S9). ^13^C-/DEPT135-NMR detected 25 carbons (four CH_2_, eight CH, five CH_3_, and eight quaternary resonances) [28]. The combination of HSQC and HMBC (Supplementary Table S1, Figure 8) unambiguously determined the constitution of vindoline (**457**). Singlet proton resonances H-2 (δ 3.72 ppm), H-19 (δ 3.58), and H-4 (δ 5.43) were valuable ‘bridge points’ with indicative ^2,3^*J*-HC long-range signals for elucidation of ring-system connections (Figure 8). Comparing the data of **427** and vindoline (**457**), stronger ^13^C-NMR *δ*-shift differences were solely seen for the aromatic ring due to the methoxy-substitution, and smaller shifts for C-2 and 12 supported the consistency of all steric centers in both IAs.

The analysis of all observed NOESY correlation signals confirmed the stereochemical centers (C-3/4/5/19) with their orientations known from X-ray analysis of the vindoline system in the IA dimer vincristine (*syn*: leurocristine) [29].

Vindoline NOE effects were presented for visualization in an *upper* and a *lower* molecular plane (Figure 8). In this approach, the starting point for observation of *NOE effect sequences* (*seq*.) was the aromatic H-14 (*δ* 7.21 ppm). *Seq. 1* (*upper*) (bold blue arrows): led to H-15 → aromatic -OCH_3_ → >N-CH_3_ → H-2 crossing over the indole to H-11a → H-14. *Seq. 2* branched at H-2 → -COOCH_3_ → H-4 → -COCH_3_ → H-6 → H-7 → H-8a/b. *Seq. 3* connected H-11a to H-12a/b and back to H-14. In *seq. 4*, the *lower* plane (dashed blue) was seen from H-14 → H-19 → ethyl protons H-20a/b connecting to *seq. 2* and directly from H-14 to 21-CH_3_ due to conformational orientation (cf. Supplementary Table S1, Figure 8).

In the *off-line* ESI-MS/MS profile of HPCCC fractions (Figure 2), as well as in the LC-ESI-MS/MS analysis of the individual CCC fractions, solely one compound was detected, indicating the complete absence of any *m*/*z* 457 isobaric metabolites. The ESI-MS/MS data of vindoline (**457**) with the most potent fragment ions at *m*/*z* 188 and 397 were in perfect accordance with Kumar et al. [30].

#### 2.5.3. Catharanthine (**337-f**): [M + H]^+^ m/z 337

**Figure 9:**
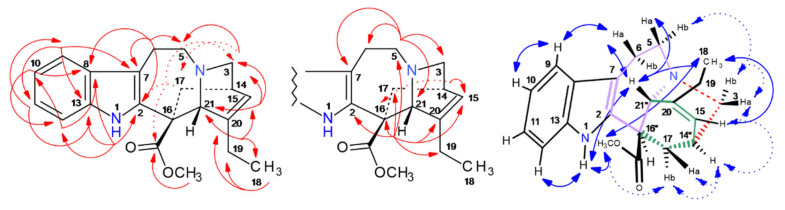
^2,3^*J*-HC long-range correlations (HMBC) (red color) and ^1^H/^1^H-NOESY of catharanthine (**337-f**) (blue) with *upper plane* in bold arrows. Asterix * for chiral centers.

Catharanthine (**337-f**)—a major concentrated alkaloid with a C16-C21 bond (resulting in the displayed bridged rigid ring system of a type II indole system (iboga alkaloid) (cf. Figure 9) [5]. **IA-337-f** was detected in the *off-line* ESI-MS experiment with [M + H]^+^ at *m*/*z* 337 in the *extrusion* process of HPCCC (t64–78, *K_D_* 3.38–11.6). The identity, the occurrence of **337-f** as a single metabolite, and the absence of respective isobars were proven by LC-C18-ESI-MS analysis and MS/MS fragmentation pattern showing the principal fragment ions *m*/*z* 305 and 144 with intensities according to literature [3].

The lipophilicity of **337-f** is due to missing polar substitutions (hydroxyl function), much higher compared to vindoline and vindorosine. Interestingly, akuammicine (fractions t60–69) appeared to be less lipophilic than catharanthine, although the amount of polarity-influencing groups seemed to be equal.

Catharanthine was solely purified by HPCCC, and 23 mg were directly used from t74 for 1D/2D NMR experiments, whereby ^13^C/DEPT135-NMR detected 21 carbon atoms (five CH_2_, seven CH, two CH_3_, and seven quaternary C).

The constitution of **337-f** was clarified by HSQC, HMBC, COSY, and NOESY (cf. Supplementary Table S1, Figure 9). Indicative ^2,3^*J*-HC long-range correlations in the HMBC, such as from H-5/6/9/15/21/14/15/16, determined the bicyclic system (Figure 9, Supplementary NMR Figure S2). The experiment in CDCl_3_ with strong correlation signals from proton >NH-1 provided helpful information for structural assignment in the HMBC and NOESY experiments. A key correlation position for observation of a large set of ^2,3^*J*-HC cross-signals was proton-signal *δ* 4.88 ppm at the C-21 chiral center.

Catharanthine contains three stereochemical centers (C-14/16/21), whereby *14S* is characteristic of this iboga-type alkaloid [5]. The ring system connections and the stereochemical arrangements (Figure 9) were determined by NOESY cross signals. NOE effects were seen in two molecular planes (*upper* and *lower*).

Also in this case of **337-f**, NOE correlation sequences for stereochemical analysis were detected, e.g., seen from aromatic H-9 (*δ* 7.49 ppm) to H-6a/6b → H-5a → H-21 methylester CH_3_-22, and NH-1 → CH_3_-18 characterizing the *upper* molecular field (bold arrows). NOE observations for the *lower* plane equally start from H-9 → H-6b → H-3b → 3a → H-14 → H-15, and then back to the *upper* plane (CH_2_-19 and CH_3_-18), closing up to the NOE *bridgehead* H-21 (*δ* 4.88 ppm). (cf. Supplementary NMR Figure S2, Figure 9). Signal overlappings in the ^1^H-NMR of H-5b (*δ* 3.43) and 6a (*δ* 3.41 ppm) prevented the precise analysis of NOESY and COSY correlations. As confirmed by NOESY, isolated catharanthine is a rigid tricyclic indole alkaloid from the *14S* series (whereby most of the iboga alkaloids belong to *14R*) [5].

A more extensive set of ‘*337 isobars*’ was detected in the *elution* range of HPCCC (Figure 2, cf. selected ion trace *m*/*z* 337 and respective MS/MS data compilation)—one of these metabolites was isolated and identified as *19R*-vindolinine (**337-b**) (cf. Section 2.5.6). All IA (**337-a 337-e**) compounds in the fractions t13 to t23 displayed a much more polar chromatographic elution behavior than catharanthine (**337-f**). However, on analytical C18-HPLC, all fractions t24/25 and t30/31 displayed similar retention times compared with catharanthine. Therefore, *head-to-tail* mode-HPCCC (*reversed phase* mode) and LC-C18 displayed complementary separation characteristics. These 337 isobars would have barely been fractioned solely by the use of a preparative C_18_-HPLC chromatographic separation.

The MS/MS fragmentation of isobars **337-a**–**337-d** was very similar; only the unknown IA **337-e** and catharanthine displayed differences in the pattern (cf. Figure 2 and Figure 4, selected ion trace *m*/*z* 337).

#### 2.5.4. Perivine (**339-a**): [M + H]^+^ m/z 339

Perivine (**339-a**) was traced by the selected single ion trace at *m*/*z* 339 in the HPCCC/ESI-MS *off-line* profile (Figure 2) in a narrow recovery range (fractions t26–28, *K_D_* 1.06–1.18). The principal compound, vindorosine (**427**), co-eluted. In consecutive steps, perivine was cleaned by C_18_-HPLC and SiO_2_-column chromatography from the most concentrated target fraction, t27.

**Figure 10:**
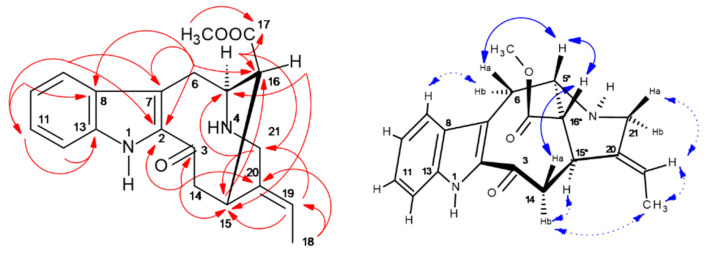
^2,3^*J*-HC long-range correlations (HMBC) (red color) and ^1^H/^1^H-NOESY of perivine (**339-a**) (blue) with *upper plane* in bold arrows. Asterix * for chiral centers.

^13^C/DEPT135-NMR detected 20 carbons consisting of three CH_2_, eight CH, two CH_3_, and seven quaternary resonances. HSQC, HMBC, and COSY (cf. Supplementary Table S1) unambiguously determined the constitution of **339-a**. ^2,3^*J*-HC correlations in the HMBC from H-5/6/14/15/16 elucidated the carbon resonances in the bicyclic system (cf. Supplementary NMR Figure S4, Figure 10).

The EI-MS of **339-a** with [M]^+^ at *m*/*z* 338 displayed the base peak ion *m*/*z* 166 and lower intensity ions at *m*/*z* 130, 172, and 279, and these ions were in good accordance with the literature [2]. HR-ESI-MS (pos. mode) resulted in [M + H]^+^ at *m*/*z* 339.17032 ([C_20_H_22_O_3_N_2_ + H]^+^, *calc.* 339.17319, −∆*m*/*z* 0.003 ppm), and indicative fragment ions *m*/*z* 124 (C_7_H_10_O_1_N_1_), 166 (C_9_H_12_O_2_N_1_), 196 (C_10_H_14_O_3_N_1_), 279 (C_18_H_19_O_1_N_2_), and 307 (C_19_H_19_O_2_N_2_) were investigated for element sum formulas [31], and MS/MS data already had been derived from *off-line* ESI-MS/MS low-resolution profile (cf. Figure 2) [2].

Perivine contains three stereochemical centers (cf. Figure 10), which had been elucidated by observed NOESY cross signals. Protons located in the ‘*upper*’ molecular plane (Figure 10, cf. Supplementary NMR Figure S4) were seen by consecutive NOE effects due to close spatial distance H-6a (*δ* 3.76 ppm) → H-5 (*δ* 2.90 ppm) → H-14a (*δ* 3.55 ppm), and on the ‘*lower*’ plane with the NOE-*seq*.: H-21a (*δ* 3.76 ppm) → H-19 (*δ* 5.81 ppm) → CH_3_-18 (*δ* 1.81 ppm) → H-14b (*δ* 2.68 ppm) → H-15 (*δ* 3.88 ppm) (Figure 10). In the HPCCC extrusion section (*K_D_* 3.14–3.50), one further 339-isobar was detected, but preparative isolation failed (cf. Figure 2).

#### 2.5.5. Akuammicine (**323-j**): ([M + H]^+^: m/z 323

Akuammicine was detected in the *off-line* ESI-MS/MS profile by the selected single ion trace [M + H]^+^ at *m*/*z* 323 during HPCCC-*extrusion* (fractions t60–69, *K_D_* 3.13–4.14) (Figure 2).

The major fractions from the HPCCC (t60–62; *K_D_* 3.13–4.14) were used for chromatographic clean-up (preparative C_18_-HPLC and SiO_2_-CC).

During short-time storage of the purified fraction in the solvent mixture of SiO_2_-CC, color-less crystals of akuammicine formed and were submitted to X-ray analysis in previous work by Yahyazadeh et al. [32]. Results of constitution and stereochemical properties were in absolute accordance with the published formula [5].

**Figure 11:**
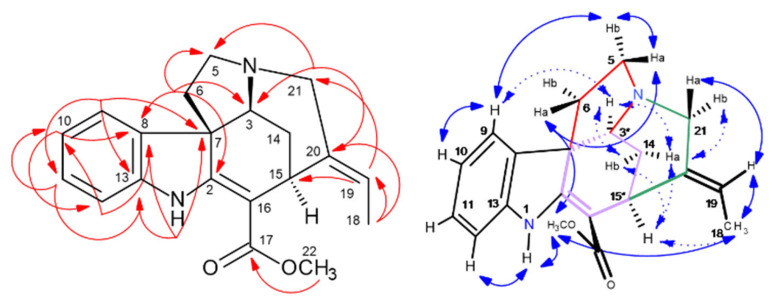
^2,3^*J*-HC long-range correlations (HMBC) (red color), and ^1^H/^1^H-NOESY of akuammicine (**323-j**) (blue) with *upper plane* in bold arrows. Asterix * for chiral centers.

Purified akuammicine (1.5 mg) was used for 1D/2D NMR analysis (cf. Supplementary Table S1 and Supplementary NMR Figure S3). ^13^C-NMR detected 20 carbons (four CH_2_, seven CH, two CH_3_, and seven quaternary resonances). HSQC, HMBC, and COSY (cf. Supplementary Table S1, Figure 11) unambiguously determined the constitution of **323-j**. Akuammicine contains three stereochemical carbon positions (C-3/7/15) (Figure 11), and these findings were confirmed by observed NOESY cross signals for the through-space proton connectivity. *Upper* (bold blue arrows) and *lower* (dashed arrows) molecular planes are displayed in Figure 11. The consecutive correlation sequences of NOE*-seq.* starting from aromatic H-9 (*δ* 7.39 ppm) to H-5b/5a → H-6a → methylester CH_3_-22 → NH-1 → H-12 defined the *upper* molecular field (bold arrows). NOE observations for the *lower* plane equally start from H-9 → H-3 → H-14a/b → H-15 → CH_3_-18. The CH_3_-18 function is a kind of bridgehead connecting over CH_3_-22 to the described *upper* layer and also a second *upper* region (H-19 and H-21a) (cf. Supplementary Table S1, Figure 11). A large number of molecular weight 323-isomers with strongly increased polarity compared to **323-j** were seen in the *elution* areas t12–13 (*K_D_* 0.21–0.28) and t26–32 (*K_D_* 1.06–1.43). Especially, the similar MS/MS fragmentation pattern of **323-j** to **323-a** and **323-b** was of interest, suggesting a molecular coherence (cf. Figure 2 and Figure 6). Principal IA 323 isobars could be strictamine, cochrovicine, 12-methoxy-vincamine, pericyclivine, and pleiocarpamine.

#### 2.5.6. *19R*-Vindolinine (**337-b**): [M + H]^+^, m/z 337

Vindolinine [33] was detected in the *off-line* ESI-MS/MS profiling of HPCCC fractions (t20–25, *K_D_* 0.68–1.00) by the single ion trace at *m*/*z* 337 at low retention volumes *V_R_* and *K_D_* values (0.68–1.00) (Figure 2) [29]; therefore, it acted as a quite polar compound in the reversed-phase operation mode (*head-to-tail* compared to the molecular weight isobar catharanthine recovered in the *extrusion* mode (cf. Section 2.5.3). In this fractionation section, several isobars with *m*/*z* 337 co-eluted and were later specifically detected by LC-ESI-MS/MS analysis (Figure 4). Results from the HPCCC/ESI-MS/MS profile already visualized that the fragment spectral data (base peak ions *m*/*z* 320, 305) were different from catharanthine (**337-f**) and indicated similar data to vindolinine or the *19S*-epimer [34]. For cleanup, HPCCC fractions t23–t25 were selected based on ESI-MS profile data for preparative C_18_-HPLC and SiO_2_-CC and subsequent 1D/2D NMR analysis.

**Figure 12:**
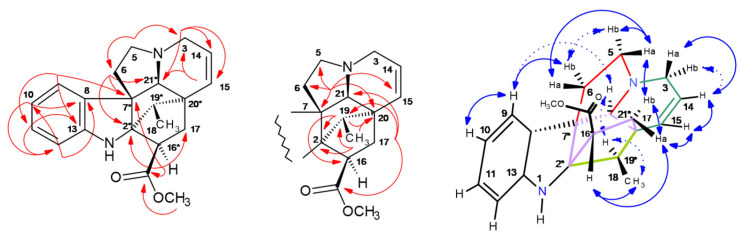
^2,3^*J*-HC long-range correlations (HMBC) (red color) and ^1^H/^1^H-NOESY of *19R*-vindolinine (**337-b**) (blue) with *upper plane* in bold arrows. Asterix * for chiral centers.

The ^13^C-/DEPT135-NMR detected 21 carbon atoms (four CH_2_, nine CH, two CH_3_, and six quaternary C). HSQC, HMBC, COSY, and NOESY experiments in CD_3_OD solvent resulted in structure **337-b** (cf. Supplementary Table S1, Figure 12) and full chemical shift assignments. ^2,3^*J*-HC long-range correlations in the benzene ring deduced the indole system, and further cross-signals H-3/5/6/15/21 determined the hexa–hydro-indolizine partial structure (cf. Supplementary NMR Figure S7, Figure 12). The bicyclic skeleton bridge from C-2 to C-20 was seen by protons H-19 (*δ* 2.16 ppm) and 18-CH_3_ (*δ* 1.11 ppm) to C-2 (*δ* 82.8 ppm), further C-20 (*δ* 45.8 ppm). The singlet signal H-21 (*δ* 3.82 ppm) was in a bridgehead position, seeing the connectivities to C-5/6/7/15/20.

Vindolinine, containing six chiral centers (C-2, 7, 16, 19, 20, and 21), was described before by four diastereomers (*19R*, *19S*, *16-epi*-*19R*, and *16-epi-19S*) [35]. For comparison of stereochemical properties, vindolinine was re-measured in CDCl_3_ (cf. Supplementary NMR Figure S8), whereby data for *19R*-vindolinine matched more precisely.

*19R*-vindolinine stereochemistry was investigated (cf. Supplementary Table S1) by NOESY correlations. Also, in this particular case, indicative NOE effects were seen in two molecular planes (*upper* and *lower,* as displayed in Figure 12).

Consecutive NOE-*seq*. were seen from aromatic H-9 (*δ* 7.18 ppm) to H-6b → H-6a → H-17b → H-17a (characterized *upper* molecular field—bold arrows). Observation of correlations from H-17a (*δ* 2.06 ppm) to chiral H-16 and 18-CH_3_ confirmed the *19R*-stereochemistry, as this methyl group would not be detectable by a NOE in a *19S*-configuration of the methyl function in the bicyclic, and also the *16-epi-*form was excluded as all protons (H-17a, H-16, and 18-CH_3_) were in very close spatial proximity. H-17a and 18-CH_3_ were detected from H-15 → H-14 → H-3a/b. The double bond proton H-15 (*δ* 6.47 ppm) was characterized by the sequence in the *lower* molecular field to H-19 → H-21 → H-9. The NOESY results completely corroborated the NMR assignments for *19R*-vindolinine (**337-b**).

## 3. Materials and Methods

### 3.1. Plant Materials

The seedlings of *Catharanthus roseus* var. Nirvana Pink Blush were obtained from the ornamental plant grower ‘Gärtnerei Volk GmbH Pflanzenhandel’ (Braunschweig, Germany). The plant seedlings were planted in equal 0.5 L pots containing 330 g using a mixture of standard garden soil and sand (2:1) and grown outside from June to July 2015.

### 3.2. Reagents

For alkaloid extraction, double distilled water and trifluoroacetic acid (TFA) (Sigma-Aldrich Chemie GmbH, Deisenhofen, Germany) were used. Solvent systems for HPCCC separations were composed of HPLC-grade solvents, ultra-clean laboratory water (NANO pure Diamond Analytical, pore size 0.2 µm, Wilhelm Werner GmbH, Leverkusen, Germany), *n*-hexane (LC–MS-grade, Fisher Scientific, Loughborough, UK), *n*-butanol, chloroform, and NaOH (Carl Roth Chemicals, Karlsruhe, Germany).

Acetonitrile for LC-MS analysis and for the ESI-MS/MS *off-line* injection profile with *make-up* solvent mixture was mass-spectrometry grade (Honeywell Speciality Chemicals Seelze GmbH, Seelze, Germany), and propionic acid (Sigma-Aldrich Chemie GmbH, Deisenhofen, Germany) for the elimination of strong TFA ion signal suppression in mass spectrometry [36].

Solvent systems for preparative HPLC separations were composed of HPLC-grade solvents, ultra-clean laboratory water (Wilhelm Werner GmbH, Leverkusen, Germany), MeOH (LC–MS-grade, Fisher Scientific, Loughborough, UK), and TFA as an ion-pair reagent for HPCCC (Sigma-Aldrich Chemie GmbH, Deisenhofen, Germany). For semi-preparative silica gel column chromatography separation, dichloromethane, methanol, and *n*-hexane (LC–MS-grade, Fisher Scientific, Loughborough, UK) and ammonia solution (25%) (HPLC LiChropur, Merck, Darmstadt, Germany) were employed.

Solvents for NMR spectroscopy were CD_3_OD (99.95% D), CDCl_3_ (99.95% D), and C_6_D_6_ (99.95%) from Deutero GmbH (Kastellaun, Germany) and internal standard TMS (Sigma-Aldrich, Deisenhofen, Germany).

### 3.3. Preparation of Alkaloid-Enriched Extracts from the Aerial Plant of C. roseus

Aerial plant parts were harvested, shock-frozen in liquid nitrogen, and stored at −20 °C. After gentle lyophilization by freeze-drying, the dried tissue material was powdered by a bead mill instrument (Mixer Mill MM, Retsch, Hann, Germany) at a vibrational frequency of 25 Hz for 1 min. Furthermore, 125 g of dried powder was immersed in 2 L of water adjusted to pH 2 by TFA, homogenized by a T-25 digital Ultra-Turrax (IKA, Staufen i. Breisgau, Germany) at maximum speed (25,000 rpm for 10 min), and shaken overnight for extraction. Plant particles were centrifuged off (30 min, 8000 rpm) (Hermle Labortechnik GmbH, Wehingen, Germany). The acidic extract was lyophilized and yielded 23 g of dried extract that was re-dissolved in 1 L of chloroform. A 1 L solution of NaOH (100 mM, pH = 12.8) was added, and the mixture was vigorously mixed for alkaloid extraction. The phases were centrifuged (8000 rpm for 15 min), and the chloroform phase was dried by immersing the flask of chloroform phase in liquid nitrogen and freeze-drying overnight at −85 °C/1.0 mbar (model: Christ Beta 2–8 LD plus, Martin Christ Gefriertrocknungsanlagen GmbH, Osterode i. Harz, Germany) in −60 °C overnight for indole alkaloid (IA) recovery and resulted in 790 mg of an IA=fortified sample.

### 3.4. Solvent System Selection by Prediction of Partition Ratio K_D_ Values in the Biphasic HPCCC Solvent System Phase Layers by LC-ESI-MS

The suitable biphasic HPCCC solvent system for the separation of *C. roseus* alkaloids was selected based on *partition-ratio factor K_D_* determination using selected single ion traces of targeted indole alkaloids (IAs) to assess the specific distributions in the biphasic phase layers, routinely measured by LC-ESI-MS analysis of aliquot volumes taken from the shake-flask experiments with the tested solvent systems [9] (cf. Figure 1). Exemplified, integration of one target selected ion trace delivers area counts (A) for the *upper*- and *lower*-layer concentrations, respectively. These are used for the *K_D_*-value equation (*head-to-tail* operation mode: *K_D_* = Area (*upper* layer)/Area (*lower* layer)). In general, *K_D_* values of target metabolites should range between 0.5 and 2.5 [11,19].

The procedure of solvent systems evaluation was performed in small shake flask experiments of the respective chosen HPCCC solvent systems by peak area integrations from LC-ESI-MS (cf. above).

For this *K_D_*-prediction purpose, 10 mg of the dried *Catharanthus* crude extract was added to the biphasic solvent systems and investigated in the shake-flask assays [5]. These systems consisted of three to four solvent components, with solvent system *SoSy1* (*n*-hexane–*n*-butanol–water with 1:1:2, *v*/*v*/*v* and addition of 5.0 mL/L TFA), *SoSy2* (*n*-hexane–*n*-butanol–water 1:1:2, *v*/*v*/*v* and some droplets of ammonia solution 25%), *SoSy3* (*n*-hexane–methanol–ethanol–water 6:5:4:5, *v*/*v*/*v*/*v*), *SoSy4* (*n*-hexane–ethyl acetate–methanol–water 3:7:5:5 *v*/*v*/*v*/*v*), *SoSy5* (*n*-butanol–acetonitrile–water 4:1:5 *v*/*v*/*v*), and *SoSy6* (*n*-hexane–ethanol–water 6:5:1 *v*/*v*/*v*). The most suitable solvent system was *SoSy1*, based on the determined target compound partition ratio values (cf. Figure 1, cf. Supplementary Figure S1).

### 3.5. Semi-Preparative Elution–Extrusion HPCCC and Peripheral Devices

Alkaloids from *C. roseus* were separated on a high-performance multilayer coil planet *J*-type HPCCC centrifuge (Spectrum, Dynamic Extractions, Gwent, UK) with two self-balanced coil bobbins and a *ß*-value range of 0.52–0.86 (equation: *ß* = coil radius r/revolution radius R) [19]. Two semi-preparative tube column systems (62.5 mL, 1.6 mm bore size i.d.) made of polytetrafluoroethylene (PTFE) were connected in series to yield a total coil column volume *V_C_* of 125 mL. The separation was performed in the *head-to-tail* (*reversed phase*) mode with the denser aqueous phase layer as the mobile phase using the evaluated solvent system *SoSy1* (water–*n*-hexane–*n*-butanol 2:1:1, *v*/*v*/*v* with an addition of TFA 5.0 mL/L as the ion-pair reagent TFA) applying the *elution–extrusion* approach [20]. The *head-end* of the coil configuration was located at the periphery of the coils. The separation was performed at the machine’s maximum velocity of 1600 rpm (g-force level 243). The liquid cooling thermostat (RC6, Lauda Dr. Wobser GmbH & Co. KG, Lauda-Königshofen, Germany) connected to the HPCCC kept the operation temperature constantly at 25 °C using. After equilibration of the different solvent volumes in a 2.5 L separatory funnel, the two-phase layers were separated shortly before use. These freshly prepared solvent layers were pumped for the separation with a preparative LC pump (solvent delivery system, K-501, Knauer Wissenschaftliche Geräte GmbH, Berlin, Germany). The hydrodynamic equilibrium with the respective *stationary phase retention—S_f_*—was determined at 1600 rpm and delivery of mobile phase at a flow rate of 5.0 mL/min.

For the calculation of *S_f_*, the volume of *mobile phase take-up*—*V_M_*—was determined (50 mL), which is equivalent to a *stationary phase volume*—vs. (75 mL) at the starting time of the HPCCC experiment, resulting in an *S_f_* value of 60.0%. These values were corrected by the external volumes of the periphery tubings (7 mL) and led to a corrected *S_f_* value of 65.6%.

The crude fortified IA crude sample (cf. alkaloid extract Section 3.3.) was injected after reaching the hydrodynamic equilibrium of the HPCCC columns.

The fortified indole alkaloid extract (790 mg) sample was dissolved in 2.5 mL aliquot volumes of the two-phase layers of *SoSy1*, filtered over a Chromafil Xtra GF-100/25 fiberglass membrane disk filter (1 µm pore size, 25 mm i.d., Macherey & Nagel, Düren, Germany), and injected through a 5.0 mL sample loop to the separation column by a manual low-pressure sample injection valve (Rheodyne, Cotati, CA, USA).

UV single-wavelength detection was conducted at λ 254 nm with a K-2501 UV detector (Knauer Wissenschaftliche Geräte GmbH, Berlin, Germany). The fraction collector SuperFrac with type B racks (Pharmacia, Uppsala, Sweden) collected fractions during the *elution and extrusion* process in intervals of 1 min.

The *elution* mode of the HPCCC experiment was run until tube fraction 58 (Figure 2), equivalent to a *switching volume V_CM_* of 290 mL, and the respective *K_D_* value (3.01) to start the *extrusion* mode until fraction t93. The chromatographic scale was converted to a *K_D_*-based axis as described [22,23,24].

Aliquots of the collected HPCCC fractions of every fraction from *elution* and *extrusion* were directly filled, in the sequence of collection, into HPLC vials as preparation for the *off-line* metabolite elution profiling experiment. Furthermore, 100 μL of each fraction was transferred and diluted with 1.4 mL of methanol (LC-MS grade) and used for sequential ESI-MS/MS injections (cf. Section 3.6.1 and Section 3.6.2).

### 3.6. Off-Line ESI-MS/MS Injection Analysis for Semi-Preparative HPCCC Indole-Alkaloid Profiling

#### 3.6.1. General Procedures

For target-guided detection monitoring of indole alkaloids separated by a semi-preparative HPCCC experiment, aliquot volumes from the recovered fractions from the *elution* and *extrusion* modes were injected *off-line* in the sequence of recovery to an ESI-MS/MS-IT mass spectrometer (HCT-Ultra ETD II, Bruker Daltonics, Bremen, Germany).

This approach projected the semi-preparative HPCCC separation by a mass-spectrometric profile using single ion traces of selected molecular weights (ESI positive ionization mode) and guided the accurate fractionation process of the indole alkaloids.

A programmable external autosampler system (AS-2000A, Merck-Hitachi, Tokyo, Japan) was used for the *off-line* ESI-MS profiling. The injected fractions were delivered to the MS device by a 1.5 m long capillary (peak tubing, 1/16 in. × 0.030 in.) by a classical binary HPLC pump (G1312A, 1100 Series, Agilent, Waldbronn, Germany) using a *makeup* solvent mixture (flow rate: 0.5 mL/min, H_2_O/ACN—50:50, *v*/*v*, and 1.0% propionic acid additive). Less volatile propionic acid was used instead of formic acid to compensate for the strong signal-quenching effects of residual TFA from the injected HPCCC samples. This polar *makeup* solvent was evaluated for effective transfer of indole alkaloids from the autosampler to the ESI-MS device to result in clear signal shapes with low width and a regular Gaussian bell shape (Figure 2). Intensity evaluations were performed by fast pre-injection experiments of selected fractions in order to determine correct injection volumes. Every fraction from the HPCCC runs (*elution* and *extrusion* mode) was sequentially injected, and data were collected in a single data file. The interval for re-occurring injections was 2 min. Every observed injection signal corresponded to one injected vial from the HPCCC experiments displaying the full molecular weight information of ionized compounds. Mass values of selected ion traces were annotated with different colors for better visual determination. Over the detected signals of one single mass, a so-called hull curve could be constructed to give an equivalent view to a full *on-line* HPCCC-ESI-MS hyphenation experiment. In total, for monitoring of *elution* and *extrusion* modes, 83 fractions were injected in the sequence of recovery.

#### 3.6.2. ESI-MS/MS Parameter Settings

##### Off-Line ESI-MS/MS

The best sensitivity for indole alkaloid detection was seen for positive ionization mode, resulting solely in [M + H]^+^ quasi-molecular ion species. The scan range was set from *m*/*z* 100 to 2000, and rapid ‘Ultra’ mode (scan rate 26,000 *m*/*z* per sec.) was chosen. Drying gas nitrogen (flow rate 10.0 L/min, 320 °C), and nebulizer pressure was set to 60 psi.

Ionization voltage HV capillary was setto −3500 V; HV end plate was offset to −500 V; trap drive was set to 55.7; octopole RF amplitude was set to 187.1 Vpp; lens 2 was set to −60.0 V; Cap Ex was set to +115.0 V; max. accumulation time was set to 200 ms; averages were at 3 spectra; trap drive level at 100%; target mass range at *m*/*z* 500; compound stability at 80%; Smart ICC target at 100,000; and the functions of ICC charge control and the smart parameter settings were activated.

For the generation of auto-MS/MS data, the 7 most intense ion precursor signals were selected for MS/MS fragmentation at an amplitude of 1.0 V.

##### LC-ESI-MS/MS

For HPCCC solvent system evaluation and semi-quantitative assessment of target distribution in the phase layers, LC-ESI-MS/MS was used in the positive ESI-MS ionization mode. This method was also used for the analysis of specific HPCCC fractions to monitor the distribution of various unidentified isobars, e.g., [M + H]^+^ *m*/*z* 339, *m*/*z* 337, and *m*/*z* 323 (cf. Section 2.4).

LC-gradient conditions were as follows: A C_18_-HPLC column, ProntoSil C18Aq column (5 μm, 250 × 2.0 mm, Knauer, Berlin, Germany) with a flow rate of 0.25 mL/min, was used. Solvent A: nanopure water/formic acid 98:2 (*v*/*v*); solvent B: acetonitrile/formic acid 98:2 (*v*/*v*). Gradient conditions were as follows: 0 min (1% B), 20 min (20% B), 35 min (50% B), 45 min (100% B), 55 min (100% B), 60 min (1% B), and 65 min (1% B). ESI-MS-MS parameter settings were as follows: the drying gas of choice was nitrogen (flow 11.0 L/min, 350 °C) and the nebulizer pressure was set to 60 psi. ESI-MS/MS ionization parameters (pos. mode) were as follows: capillary at -3500 V, end plate offset at −500 V, capillary exit at +121 V, target mass range at *m*/*z* 500, compound stability at 100%, trap drive level at 100%, ICC target at 100,000, max. accumulation time at 200 ms, ICC charge control on, scan range at *m*/*z* 100–2000, threshold auto MS/MS at 10,000, and MS/MS at fragmentation amplitude at 1.0 V.

### 3.7. Drying of HPCCC Fractions for Preparative Clean-Up

Subsequent to the sampling process for the *off-line* injection ESI-MS/MS profile, all recovered HPCCC fractions from the *elution* and *extrusion* processes were gently dried with a SpeedVac Plus concentrator equipped with a rotor for the used fraction collector tubes (SC210A and refrigerated vapor trap RVT 400, Thermo Savant, Holbrook, NY, USA).

### 3.8. Calculation of K_D_ Scale of HPCCC Experiment

The HPCCC time-dependent *elution–extrusion* scale from the ESI-MS injection profile (cf. Figure 2) was converted by the calculated *elution* or *retention volumes V_R_* into the respective *partition ratio* values *K_D_*. The conversion of CCC separation times into a *K_D_*-based scale delivers solvent-system-specific constants to compare experimental results generated on different CCC devices, such as during *scale-up* studies. Accurate calculation of appropriate *K_D_* values of the specifically detected metabolites of the HPCCC separation and *stationary phase retention S_F_* was corrected by the machine’s *extra column volumes V_Ext_* (e.g., periphery tubings). The *K_D_* calculation workflow was described in detail by Tran et al. [23] and Grecco et al. [22].

### 3.9. Preparative RP18-HPLC

The selected HPCCC fractions were pooled on behalf of results from the off-line ESI-MS/MS profiling and TLC results and then re-chromatographed by preparative RP18-HPLC (Wellchrom K-1001, Knauer Gerätebau Berlin, Germany) using a C18 column (Prontosil C18Aq, 25 × 250 mm) and an isocratic flow rate of 4.5 mL/min (acetonitrile/water, 60:40 with 1% TFA). Alkaloids were monitored using a UV detector (Wellchrom K-2600, Knauer Gerätebau, Berlin, Germany) at λ 254, 280, and 300 nm (cf. separation scheme Supplementary Figure S10).

### 3.10. Open-Column (CC-SiO_2_) Separation and TLC Analysis

The amber-colored HPLC fractions were finally purified by SiO_2_ column chromatography (Merck, Darmstadt, Germany) using ethyl acetate/*n*-hexane/ethanol/25% aqueous ammonia (100/5/5/3) to yield pure alkaloids, detected by thin-layer chromatography (TLC) (SiO_2_ 60 F254, Merck, Darmstadt, Germany) using this solvent system and visualization by alkaloid-sensitive Dragendorff spray reagent (cf. separation scheme in Supplementary Figure S10).

### 3.11. NMR

1D (^1^H, ^13^C, and DEPT135) and 2D NMR spectroscopy (HSQC phase-edited, HMBC, ^1^H/^1^H-COSY, and ^1^H/^1^H-NOESY) were recorded on a 300 MHz spectrometer (FT 300, Bruker Biospin, Rheinstetten, Germany) at ambient temperature (295K).

Chemical shifts in the *^1^H* were referenced to TMS (*δ*: 0.00 ppm), and the *^13^C* to the respective solvent signals (cf. Supplementary Table S1, Supplementary Figure S1). Samples were measured in CD_3_OD, CDCl_3_, and C_6_D_6_ (cf. Supplementary Figures S2–S9).

## 4. Conclusions

In the present study, on indole alkaloid recovery from a *Catharanthus roseus* crude extract, all-liquid countercurrent chromatography (HPCCC) separation was applied and combined with mass-spectrometric guided *off-line* ESI-MS/MS target profiling. This approach was an effective and powerful hyphenation to fractionate and trace IAs in a semi-preparative process of 790 mg of fortified extract and finally led to six purified compounds, i.e., (*19R*)-vindolinine, akuammicine, perivine, catharanthine, vindoline, and vindorosine. These recovered IAs were fully elucidated and confirmed by 1D/2D NMR experiments.

The principal aim of sensitive ESI-MS/MS monitoring was to generate a molecular weight profile of IAs and visualize the chromatography on the basis of mass values covering the complete HPCCC separation process for the sections of *elution* and *extrusion* modes. Therefore, unintentional mixing of already purified metabolites by false pooling of fractions was prevented, and exact ranges of chromatographic co-elution regions were determined using selected single ion traces and were expressed by *partition-ratio* values. Target fractions were clearly identified, as well as unknown or very minor concentrated isobaric metabolites. The diverse distributed recovery ranges on HPCCC of molecular weight isobars, e.g., *m*/*z* 337, such as the pair—*19R*-vindolinine and catharanthine—were clearly traced. We propose that a further increase in the TFA ion-pair reagent concentration in the biphasic solvent system will positively influence the HPCCC resolution of target IAs in this study and might reduce the observed tailing effect of vindorosine.

The HPCCC separation appeared to be more efficient than preparative C_18_-HPLC, as this would have been problematic, as already analytical C_18_-LC retention time differences were rather small. Isobars of *m*/*z* 323 were nicely fractionated, and one major compound was cleaned to result in akuammicine. Also, the IA pair, vindoline (**457**) and vindorosine (**427**), were pre-fractionated by HPCCC, and this process compensated for difficulties of low retention time differences on preparative C_18_-HPLC. Chromatographic key values, such as compound-specific and also biphasic solvent-dependent *partition ratio K_D_* values, were determined and could be used as valuable separation parameters for future scale-up or optimization of the whole IA recovery process. The complexity of IAs in the *C. roseus* extract was enormously high; two further downstream purification steps were required and led to the pure and NMR-suitable samples.

Overall, the complementary separation characteristics of the all-liquid HPCCC technique and classic column chromatography in their combination resulted in an efficient workflow for IA purification. In our case, the mass-spectrometric profiling was conducted on semi-preparative HPCCC scale but could easily be used to work in a process of larger laboratory CCC or centrifugal partition chromatography (CPC) recovery scales [22]. Also, IA dimers of *Catharanthus* with strong anti-tumor activity, such as vincristine, were detected by the *off-line* ESI-MS monitoring process in respective HPCCC fractions. However, as very minor concentrated compound fractions resulted, a further cleanup to recover amounts (mg) for NMR failed. The respective *K_D_* values were determined and could be used directly for high-load preparative recovery CCC studies, although this would require larger IA concentrations to perform a reasonable *scale-up* for recovery of vincristine and vinblastine, still being a very important part of the portfolio of treatments of modern anti-tumor therapy. Overall, the monomeric IA precursors currently being used for the semi-synthesis of vincristine and vinblastine could be effectively recovered by all-liquid HPCCC methods.

## Figures and Tables

**Table 1:** IA-elution sequence (IA1 to IA6) on HPCCC (*head-to-tail* mode) based on functional groups (specific formulas of IAs cf. Section 2.5).

**1**	*19R*-Vindolinine (**337-b**)	-NH	-COOCH_3_				
**2**	Vindoline (**457**)	-N-CH_3_	-COOCH_3_	-OH	-OAc	-CH_2_-CH_3_	
**3**	Perivine (**339-a**)	-NH	-COOCH_3_			-CH_2_-CH_3_	
**4**	Vindorosine (**427**)	-N-CH_3_	-COOCH_3_	-OH	-OAc	-CH_2_-CH_3_	-O-CH_3_
**5**	Akuammicine (**323-j**)	-NH	-COOCH_3_				
**6**	Catharanthine (**337-f**)	-NH	-CO	-OH	-OAc	-CH_2_-CH_3_	

## Data Availability

Original data files are available and access to them can be requested.

## Supplementary Materials

The following supporting information can be downloaded at: https://www.mdpi.com/article/10.3390/molecules30102115/s1. Figure S1: Solvent system evaluation SoSy 2–6. Figures S2–S9 (NMR S2–S9): 1D/2D NMR original spectral data. Figure S10: Chromatography separation scheme of IAs. Table S1: Compiled 1D/2D NMR data of recovered and elucidated IAs.

## Abbreviations

HPCCC	High-performance countercurrent chromatography
CPC	Centrifugal partition chromatography
TLC	Thin-layer chromatography
LC-ESI-MS	Liquid chromatography–electrospray–mass-spectrometry
IAs	Indole alkaloids
NMR	Nuclear magnetic resonance spectroscopy
DEPT135	Distortionless enhancement by polarization transfer
HSQC	Hetero single-quantum transfer
HMBC	Hetero multiple-bond correlation
COSY	Correlation spectroscopy
NOESY	Nuclear Overhauser-effect spectroscopy
