# Supplementary material for: Electrospray–Mass Spectrometry-Guided Targeted Isolation of Indole Alkaloids from Leaves of Catharanthus roseus by Using High-Performance Countercurrent Chromatography
Source: Molecules. 2025 May 9;30(10):2115. doi: 10.3390/molecules30102115 (PMC12113773; doi:10.3390/molecules30102115)
Supplement: Supplementary file 1 [file molecules-30-02115-s001.zip › Supplement Figure S1.pdf]

Supplement Figure S1 : Solvent System evaluation by LC-ESI-MS SoSy2 – SoSy6

Solvent system 2: *n*-hexane / *n*-butanol / aqueous NH_3 (25%) some droplets = 1 : 1 : 2

Compound distribution was not suitable : indole alkaloids were located in the upper more organic stationary phase, solely akuammicine displayed a suitable K_D 2.19.

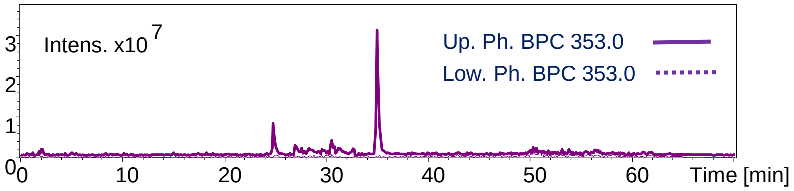

Ajmalicine [M+H]⁺ : *m/z* 353

K_D not in the range of 0.5 – 5.0

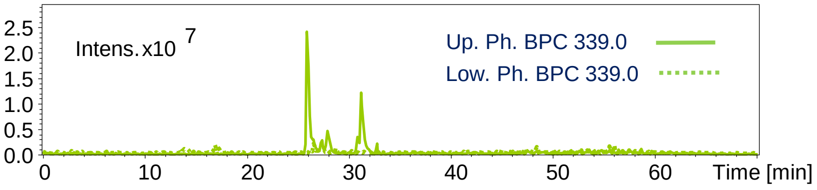

Perivine [M+H]⁺ : *m/z* 339

K_D not in the range of 0.5 – 5.0

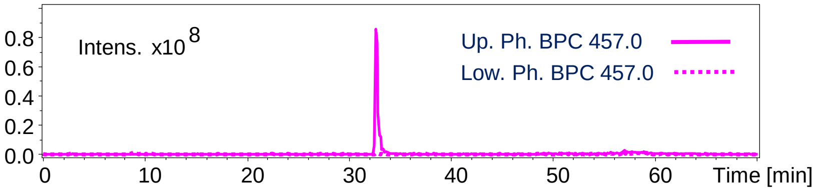

Vindoline [M+H]⁺ : *m/z* 457

K_D not in the range of 0.5 – 5.0

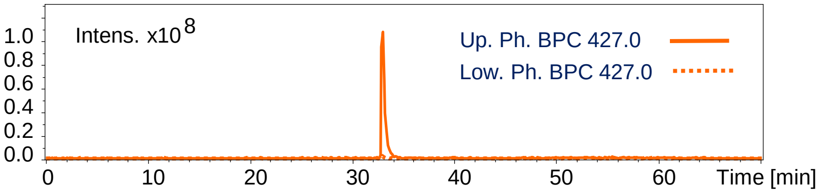

Vindorisine [M+H]⁺ : *m/z* 427

K_D not in the range of 0.5 – 5.0

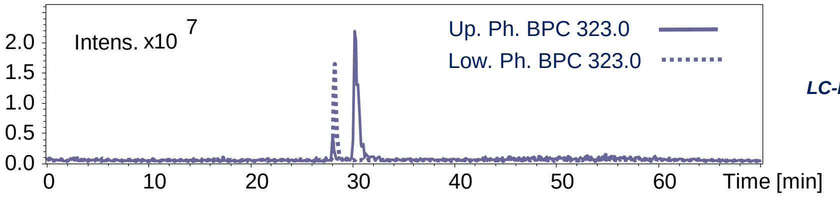

Akuammicine [M+H]⁺ : *m/z* 323

LC-MS prediction K_D **(323) = (Area upper-stat.) 655233995 / (Area lower-mob.) 299252777 = 2.19**

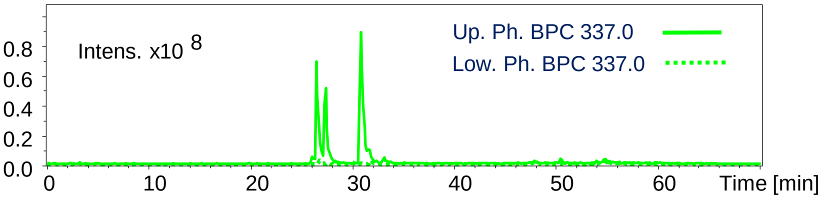

337-Isobars [M+H]⁺ : *m/z* 337

K_D not in the range of 0.5 – 5.0

Solvent system 3: *n*-hexane / methanol / ethanol / water = 6 : 5 : 4 : 5

Distribution was not suitable : all indole alkaloids were located in the lower more aqueous mobile phase.

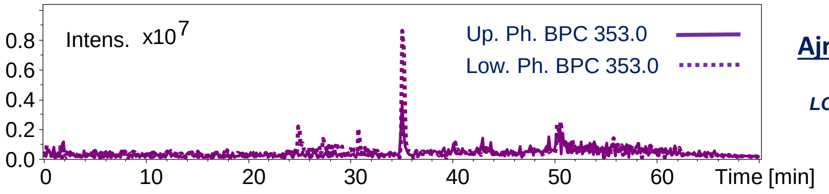

Ajmalicine $[M+H]^+$: *m/z* 353

LC-MS prediction K_D **(353) = (Area upper-stat.) 60247963 / (Area lower-mob.) 163050207 = 0.37**

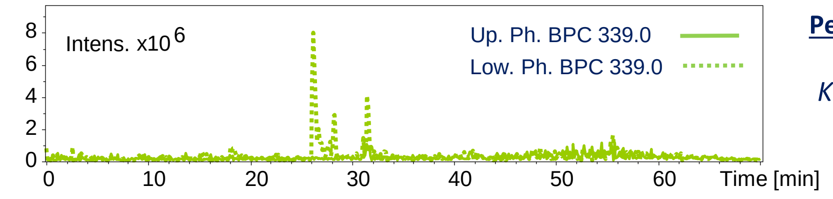

Perivine $[M+H]^+$: *m/z* 339

K_D not in the range of 0.5 – 5.0

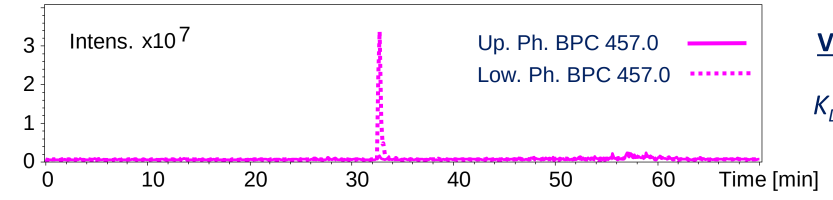

Vindoline $[M+H]^+$: *m/z* 457

K_D not in the range of 0.5 – 5.0

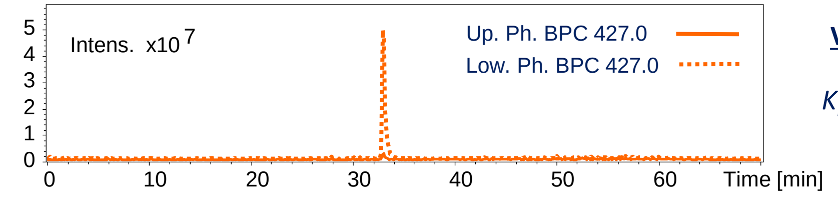

Vindorisine $[M+H]^+$: *m/z* 427

K_D not in the range of 0.5 – 5.0

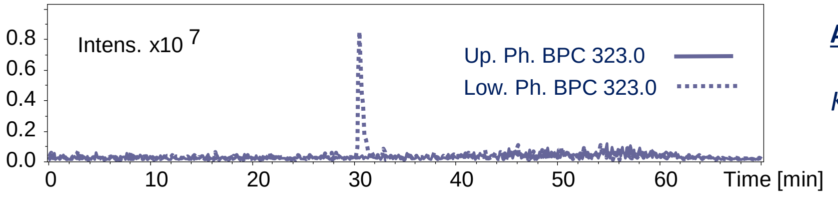

Akuammicine $[M+H]^+$: *m/z* 323

K_D not in the range of 0.5 – 5.0

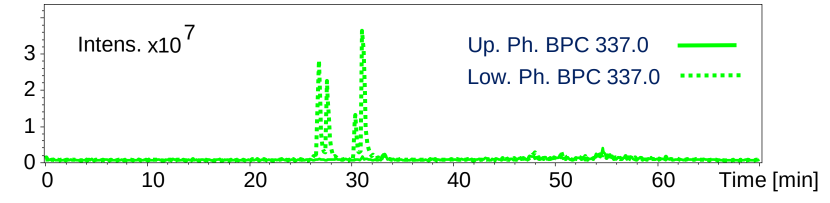

337-Isobars $[M+H]^+$: *m/z* 337

K_D not in the range of 0.5 – 5.0

Solvent system 4: *n*-hexane / methanol / ethanol / water = 3 : 7 : 5 : 5

Distribution was not suitable : most alkaloids were located in the lower mobile more aqueous phase with K_D approx. 0.5.

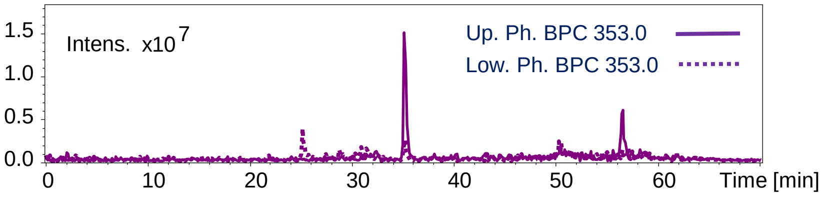

Ajmalicine [M+H]⁺ : *m/z* 353

K_D not in the range of 0.5 – 5.0

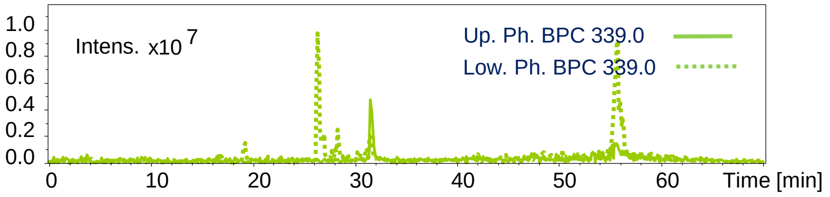

Perivine [M+H]⁺ : *m/z* 339

K_D not in the range of 0.5 – 5.0

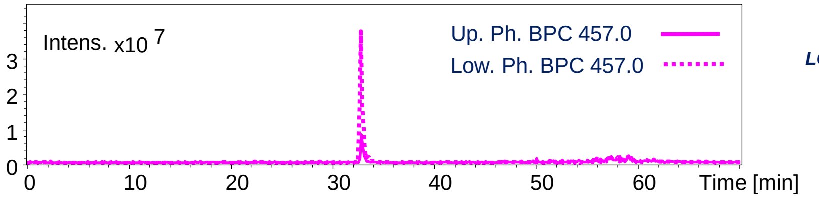

Vindoline [M+H]⁺ : *m/z* 457

LC-MS prediction K_D **(457) = (Area upper-stat.) 130552434 / (Area lower-mob.) 694241870 = 0.19**

K_D not in the range of 0.5 – 5.0

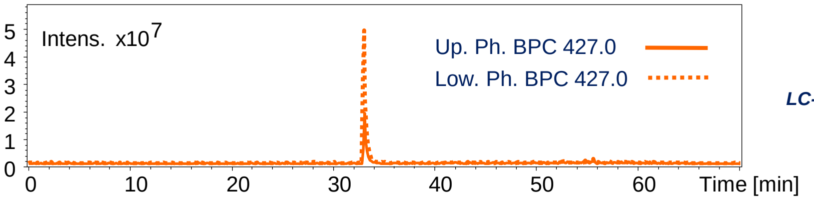

Vindorisine [M+H]⁺ : *m/z* 427

LC-MS prediction K_D **(427) = (Area upper-stat.) 333508477 / (Area lower-mob.) 1055663444 = 0.31**

K_D not in the range of 0.5 – 5.0

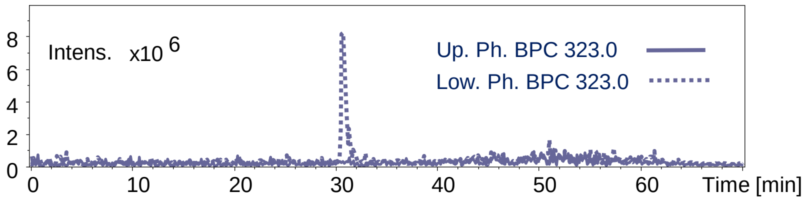

Akuammicine [M+H]⁺ : *m/z* 323

K_D not in the range of 0.5 – 5.0

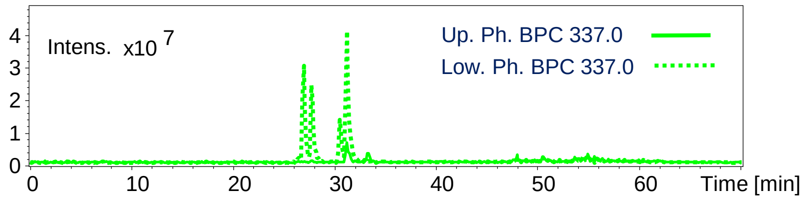

337-Isobars [M+H]⁺ : *m/z* 337

K_D not in the range of 0.5 – 5.0

Solvent system 5: *n*-butanol / acetonitrile / water   4 : 1 : 5

Distribution was not suitable : the more lipophil indole alkaloids were in the upper stationary phase and the more polar in the lower mobile phase.
Vindoline (457) and Vindorosine (427) displayed good K_D-values around ~1 but the other alkaloids would be separated more by single step liquid liquid partioning steps.

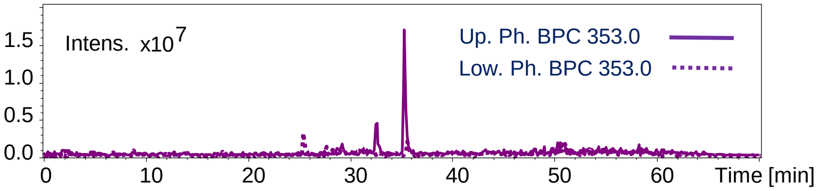

Ajmalicine [M+H]⁺ : *m/z* 353

K_D not in the range of 0.5 – 5.0

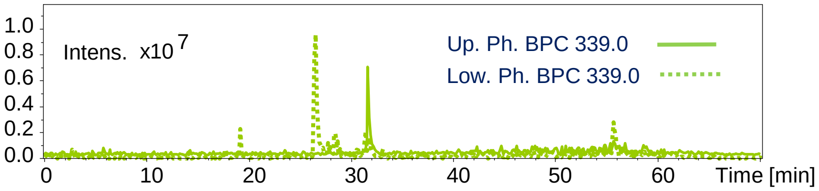

Perivine [M+H]⁺ : *m/z* 339

K_D not in the range of 0.5 – 5.0

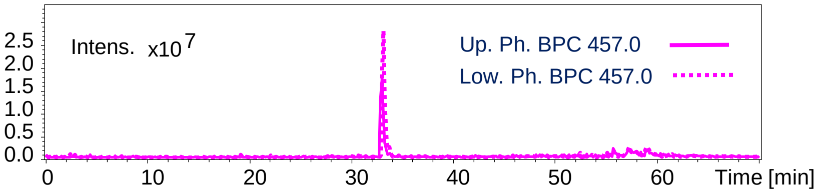

Vindoline [M+H]⁺ : *m/z* 457

LC-MS prediction K_D **(457) = (Area upper-stat.) 535025891 / (Area lower-mob.) 694241870 = 0.77**

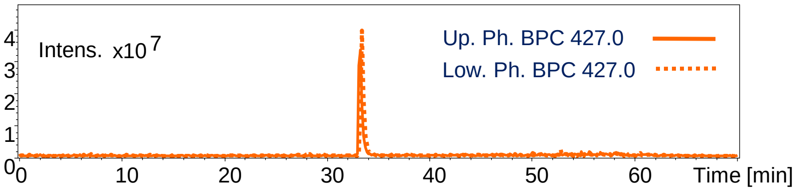

Vindorisine [M+H]⁺ : *m/z* 427

LC-MS prediction K_D **(427) = (Area upper-stat.) 743888029 / (Area lower-mob.) 752157768 = 0.99**

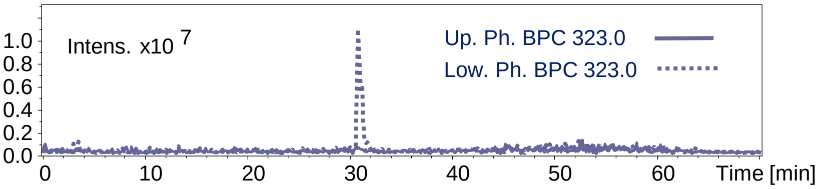

Akuammicine [M+H]⁺ : *m/z* 323

K_D not in the range of 0.5 – 5.0

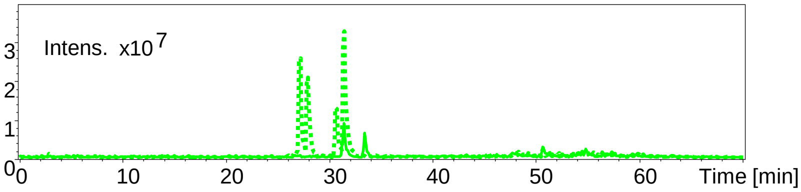

337-Isobars [M+H]⁺ : *m/z* 337

K_D not in the range of 0.5 – 5.0

Solvent system 6 : *n*-hexane / ethanol / H_2O 6 : 5 : 1

Distribution was not suitable : the more lipophil indole alkaloids were in the upper stationary phase and the more polar in the lower mobile phase. Vindoline (457) and vindorosine (457) displayed quite large but acceptable K_D-value ranges 2.7 – 3.7

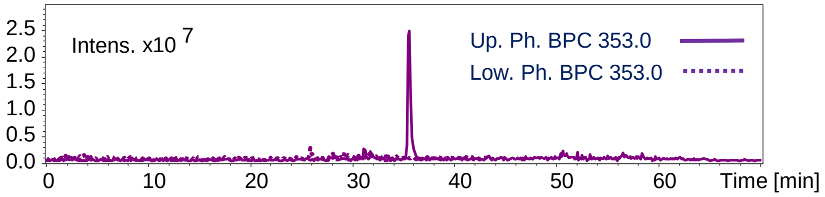

Ajmalicine $[M+H]^+$: *m/z* 353

K_D not in the range of 0.5 – 5.0

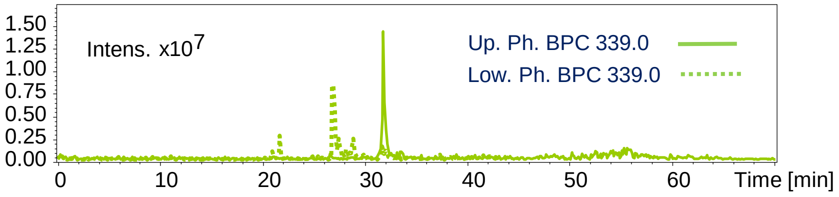

Perivine $[M+H]^+$: *m/z* 339

K_D not in the range of 0.5 – 5.0

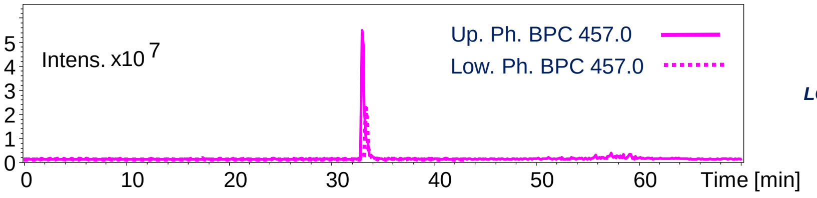

Vindoline $[M+H]^+$: *m/z* 457 (*457*)

***LC-MS prediction K_D* (457) = (Area upper-stat.) 1035329089 / (Area lower-mob.) 381436162 = 2.71**

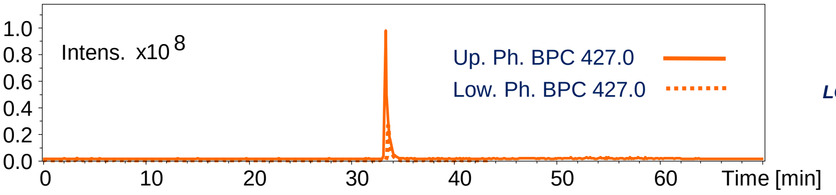

Vindorisine $[M+H]^+$: *m/z* 427

***LC-MS prediction K_D* (427) = (Area upper-stat.) 1828363776 / (Area lower-mob.) 497915151 = 3.67**

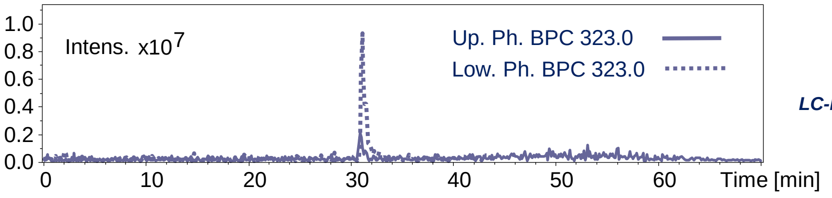

Akuammicine $[M+H]^+$: *m/z* 323

***LC-MS prediction K_D* (323) = (Area upper-stat.) 59167726 / (Area lower-mob.) 497915151 = 0.12**

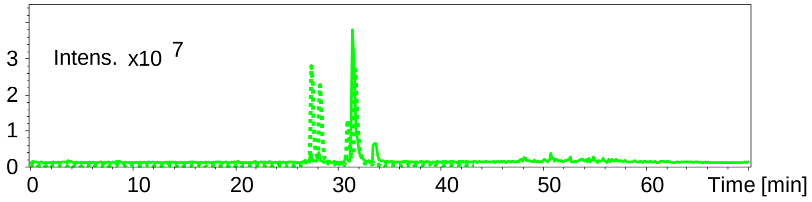

337-Isobars $[M+H]^+$: *m/z* 337

K_D not in the range of 0.5 – 5.0
