# Supplementary material for: Electrospray–Mass Spectrometry-Guided Targeted Isolation of Indole Alkaloids from Leaves of Catharanthus roseus by Using High-Performance Countercurrent Chromatography
Source: Molecules. 2025 May 9;30(10):2115. doi: 10.3390/molecules30102115 (PMC12113773; doi:10.3390/molecules30102115)

Supplement NMR Figures S2-S9, pages 3 –135:
1D/2D-NMR spectra of indol alkaloids from Catharanthus roseus **(^{1}H: 300 MHz; ^{13}C: 75 MHz)**

Electrospray-Mass-Spectrometry Guided Recovery Profiling of Indole Alkaloids from Leaves of *Catharanthus roseus* by High-Performance Countercurrent Chromatography

Mahdi Yahyazadeh [1,2], Dirk Selmar [2], and Gerold Jerz [3,*]

1 Research Institute of Forests and Rangelands, Agricultural Research, Education and Extension Organization (AREEO),

Tehran, Iran.; m.yahyazadeh-balalami@tu-bs.de,

2 Institute of Plant Biology, TU Braunschweig, Mendelssohnstr. 4, 38106 Braunschweig, Germany; d.selmar@tu-bs.de

3 Institut für Lebensmittelchemie, Technische Universität Braunschweig, Schleinitz-Str. 20, 38106 Braunschweig, Germany

* Corresponding author: g.jerz@tu-braunschweig.de (G.J.)

Supplement NMR Figures S1-S8 pages 3 –135:
1D/2D-NMR spectra of indol alkaloids from Catharanthus roseus **(^{1}H: 300 MHz; ^{13}C: 75 MHz)**

pp. 3 - 21, NMR-S2 : *1D/2D-NMR of* Catharanthine (*337-f*): (NMR-ID GJ243) in **$CDCl_3$** calib. to TMS (^{1}H) and to solvent signal 77.26 (^{13}C), δ [ppm], *J* [Hz]. Reference data: Wenkert et al. 1976 in $CDCl_3$ Helv Chim Ac

pp. 22 – 40, NMR-S3 : *1D/2D-NMR of* Akuammicine (*323-j*) (NMR-ID GJ408) in **$CDCl_3$** calib. to TMS (^{1}H) δ 0.00 [ppm], and to solvent signal (^{13}C) δ 77.26 [ppm], *J* [Hz]. Reference data: Kuehne et al J Org Chem 1994.

pp. 41 – 57, NMR-S4 : *1D/2D-NMR of* Perivine (*339-e*): (NMR-ID GJ318) in **CD_3OD** calib. to TMS (^{1}H) δ 0.00 [ppm], and solvent signal δ 49.0 [ppm] (^{13}C), *J* [Hz].

pp. 58 – 78, NMR-S5 : *1D/2D-NMR of* Vindorosine (*427*) (syn. demethyoxy-vindoline, vindolidine)**:** (NMR-ID GJ238) in **CD_3OD** calib. to TMS (^{1}H) δ 0.00 [ppm], and δ 49.0 [ppm] (^{13}C), *J* [Hz].

pp. 79 – 91 , NMR-S6 : *1D/2D-NMR of* Vindorosine (*427*) (syn. demethyoxy-vindoline, vindolidine)**:** (NMR-ID GJ338) in **C_6D_6** calib. TMS (^{1}H) δ 0.00 [ppm], and solvent signal δ 128.5 [ppm] (^{13}C), *J* [Hz].

pp. 92 – 112, NMR-S7 : *1D/2D-NMR of 19R*-Vindolinine (*337-b*): (NMR-ID GJ317+GJ320) in **CD_3OD** calib. TMS (^{1}H) δ 0.00 [ppm], and δ 49.0 [ppm] (^{13}C), *J* [Hz]:

pp. 113 – 116, NMR-S8 : *1D/2D-NMR of 19R*-Vindolinine (*337-b*): (NMR-ID GJ434) in **$CDCl_3$** calib. to TMS (^{1}H) δ 0.00 [ppm], and to solvent signal (^{13}C), δ 77.26 [ppm], *J* [Hz]. Reference data Att-ur-Rahman 1983 Z. Naturforsch.

pp. 117 – 135, NMR-S9 : *1D/2D-NMR of* Vindoline (457): (NMR-ID GJ319+GJ275) in **CD_3OD** calib. TMS (^{1}H) δ 0.00 [ppm], and δ 49.0 [ppm] (^{13}C), *J* [Hz].

Figure NMR-S2

^{1}H NMR – Catharanthine (*337-f*) in $CDCl_3$ (300 MHz)

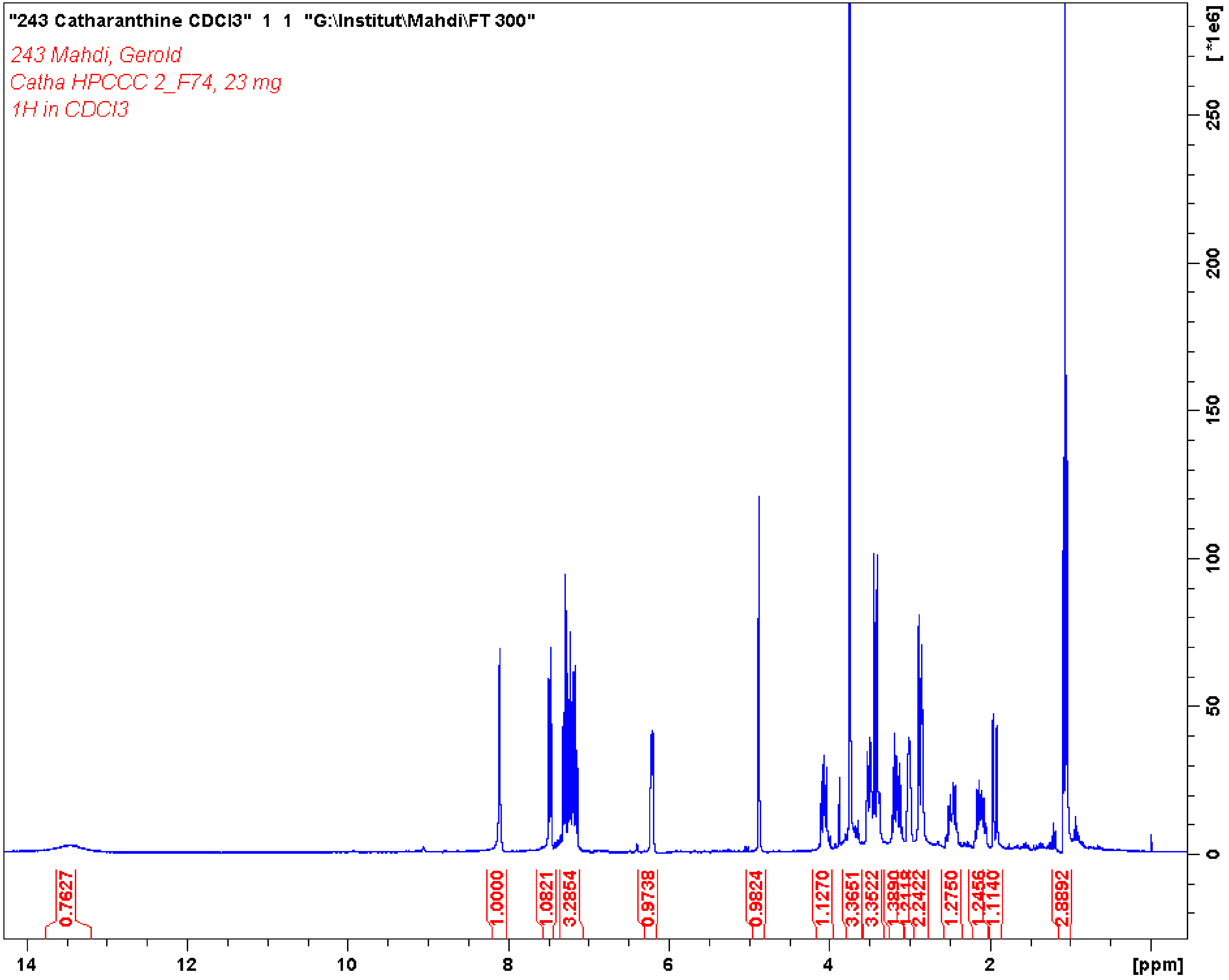

Figure NMR-S2

^{1}H NMR – Catharanthine (*337-f*) in $CDCl_3$ (300 MHz)

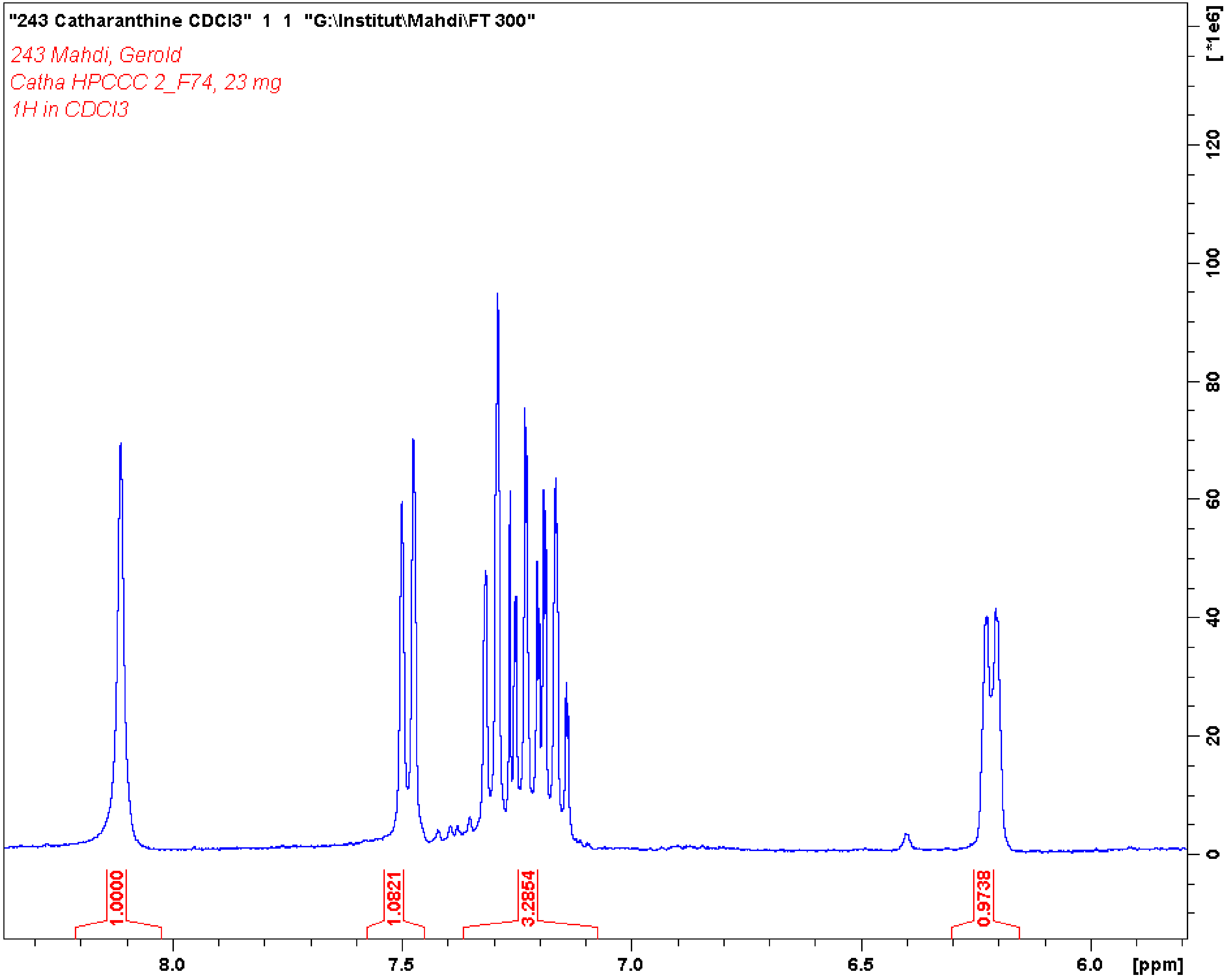

Figure NMR-S2

^{1}H NMR – Catharanthine (*337-f*) in $CDCl_3$ (300 MHz)

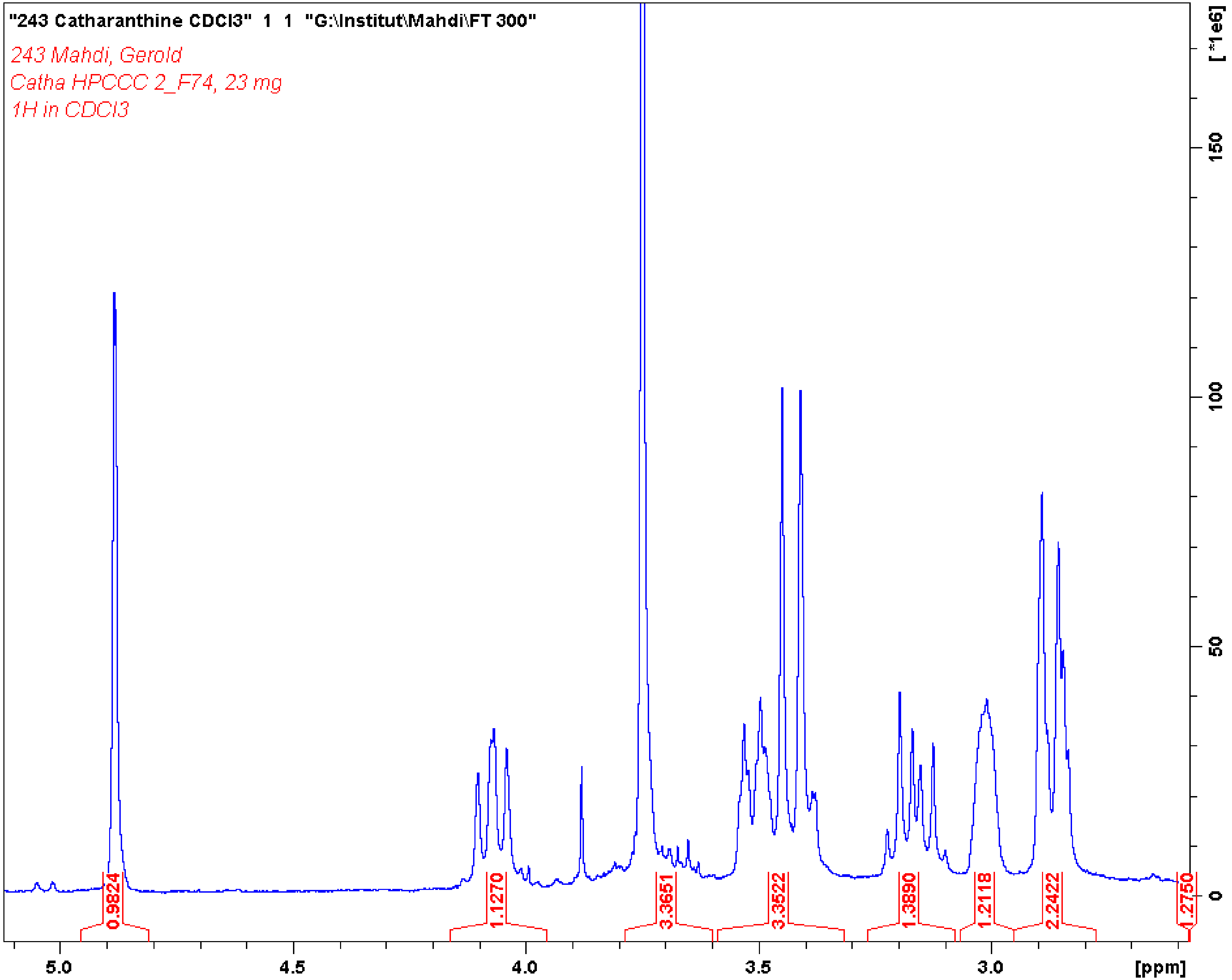

Figure NMR-S2

^{1}H NMR – Catharanthine (*337-f*) in $CDCl_3$ (300 MHz)

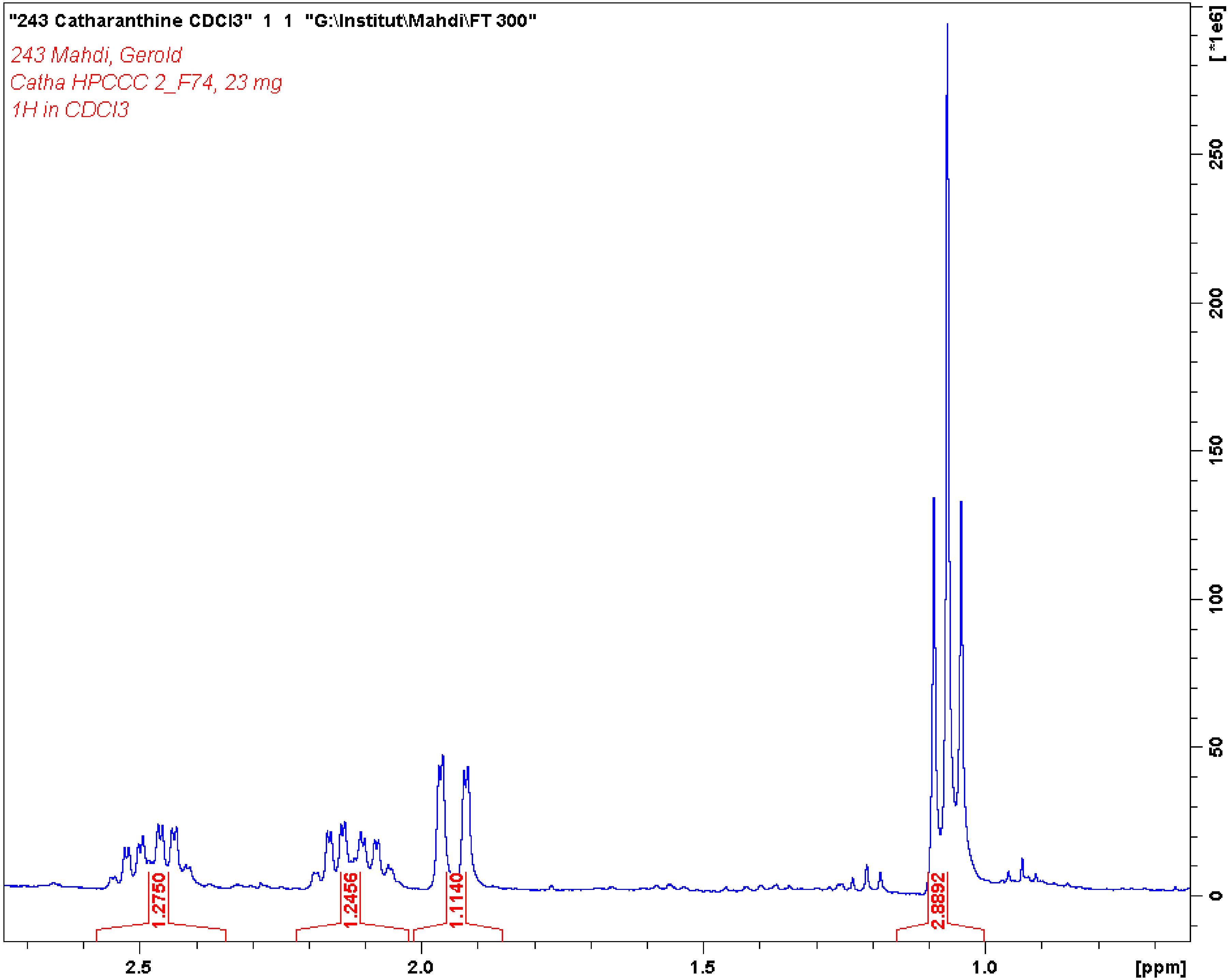

Figure NMR-S2

^{13}C NMR – Catharanthine (*337-f*) in $CDCl_3$ (75 MHz)

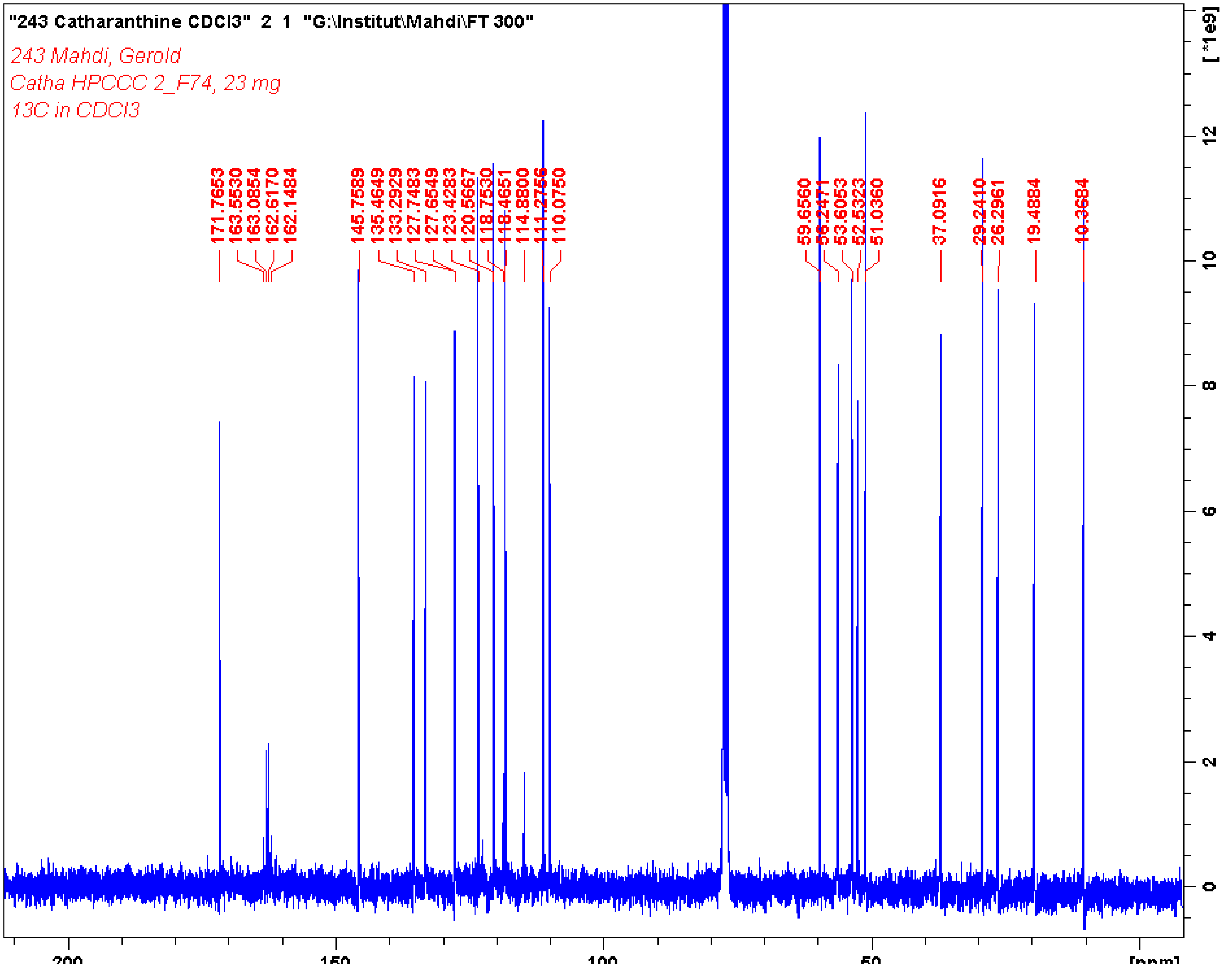

Figure NMR-S2

^{13}C NMR – Catharanthine (*337-f*) in $CDCl_3$ (75 MHz)

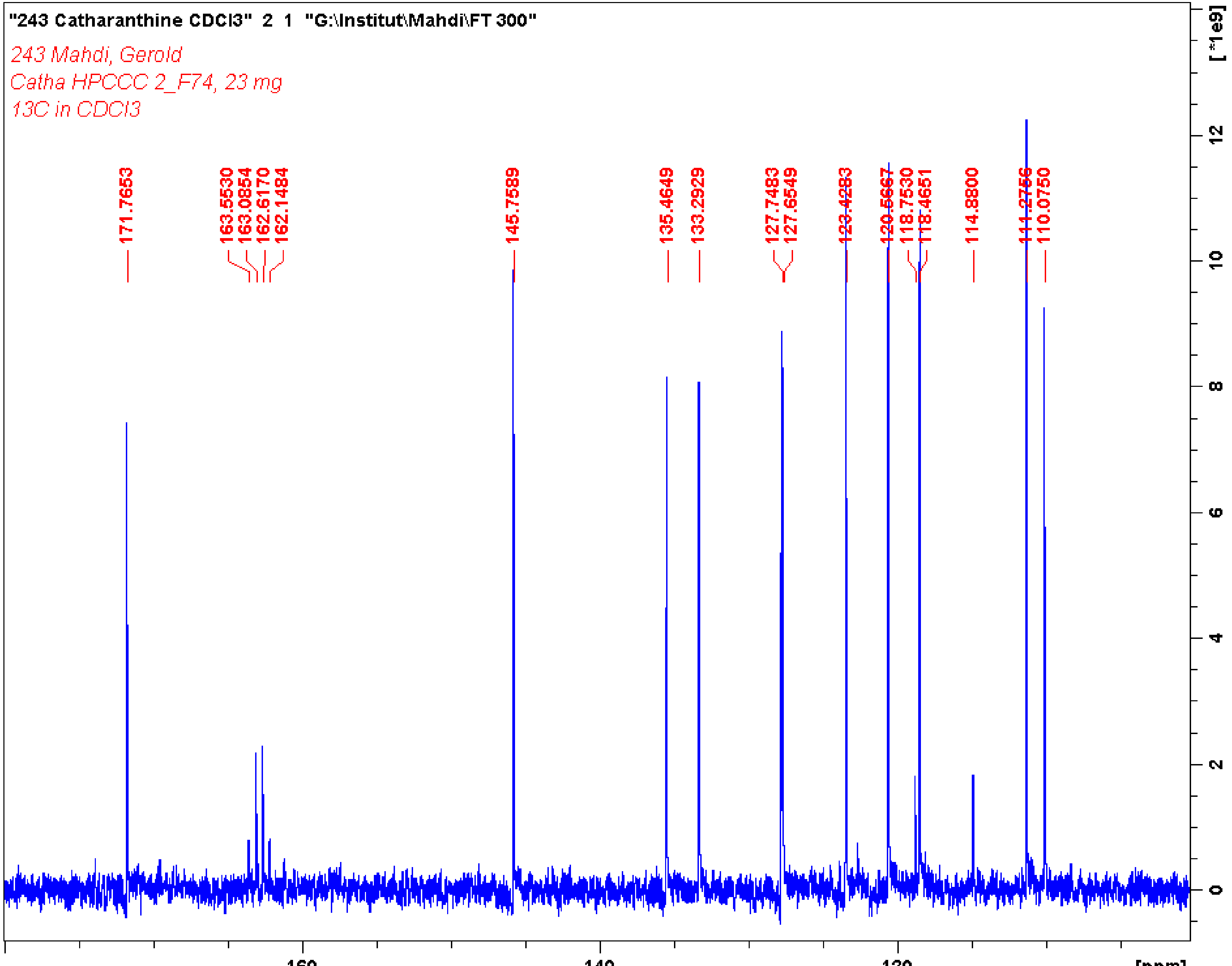

Figure NMR-S2

^{13}C NMR – Catharanthine (*337-f*) in $CDCl_3$ (75 MHz)

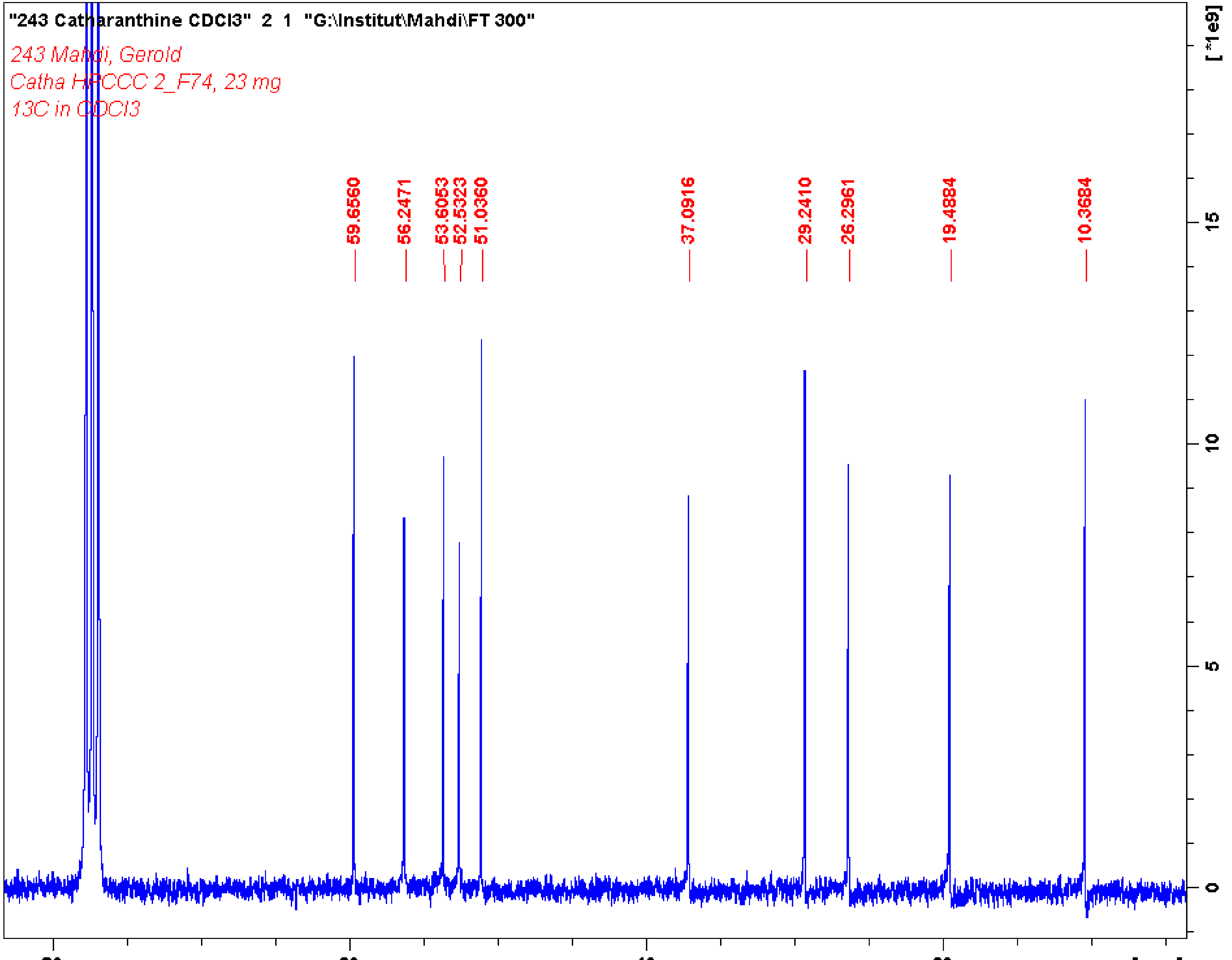

Figure NMR-S2

DEPT135 NMR
– Catharanthine (*337-f*)
in $CDCl_3$
(75 MHz)

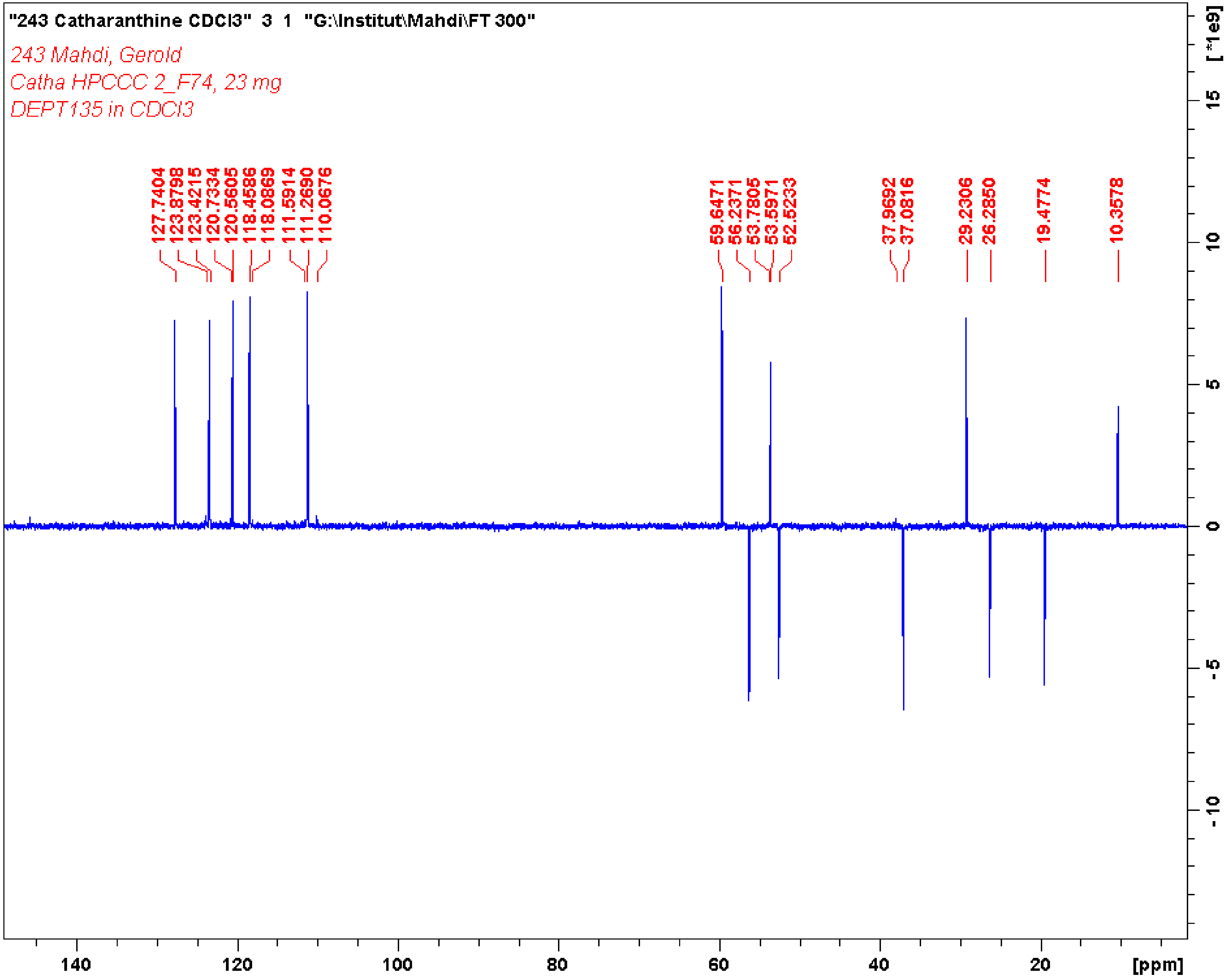

Figure NMR-S2

$^{1}H/^{1}H$-COSY – Catharanthine (*337-f*) in $CDCl_3$

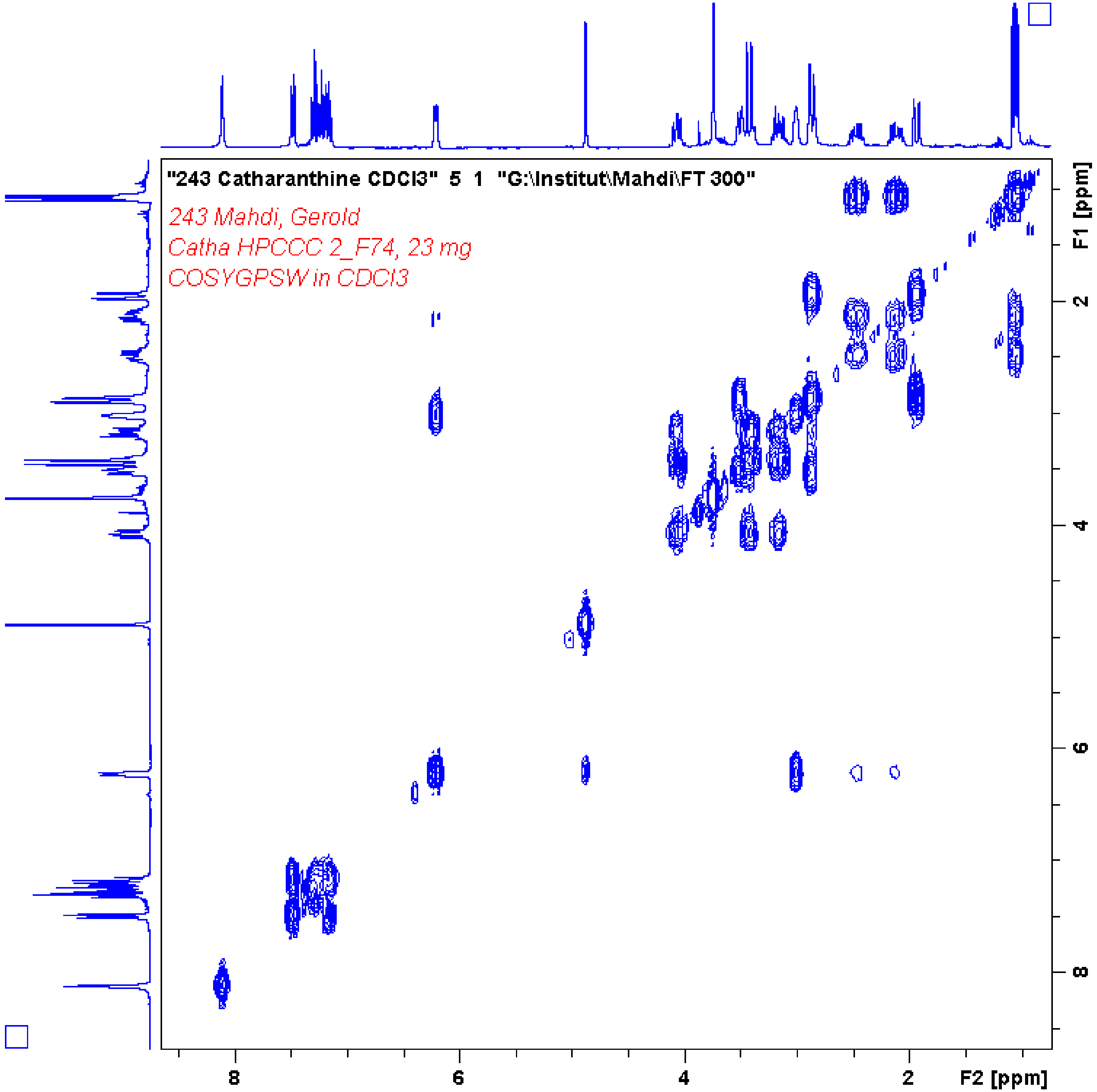

Figure NMR-S2

$^1H/^1H$-COSY – Catharanthine (*337-f*) in $CDCl_3$

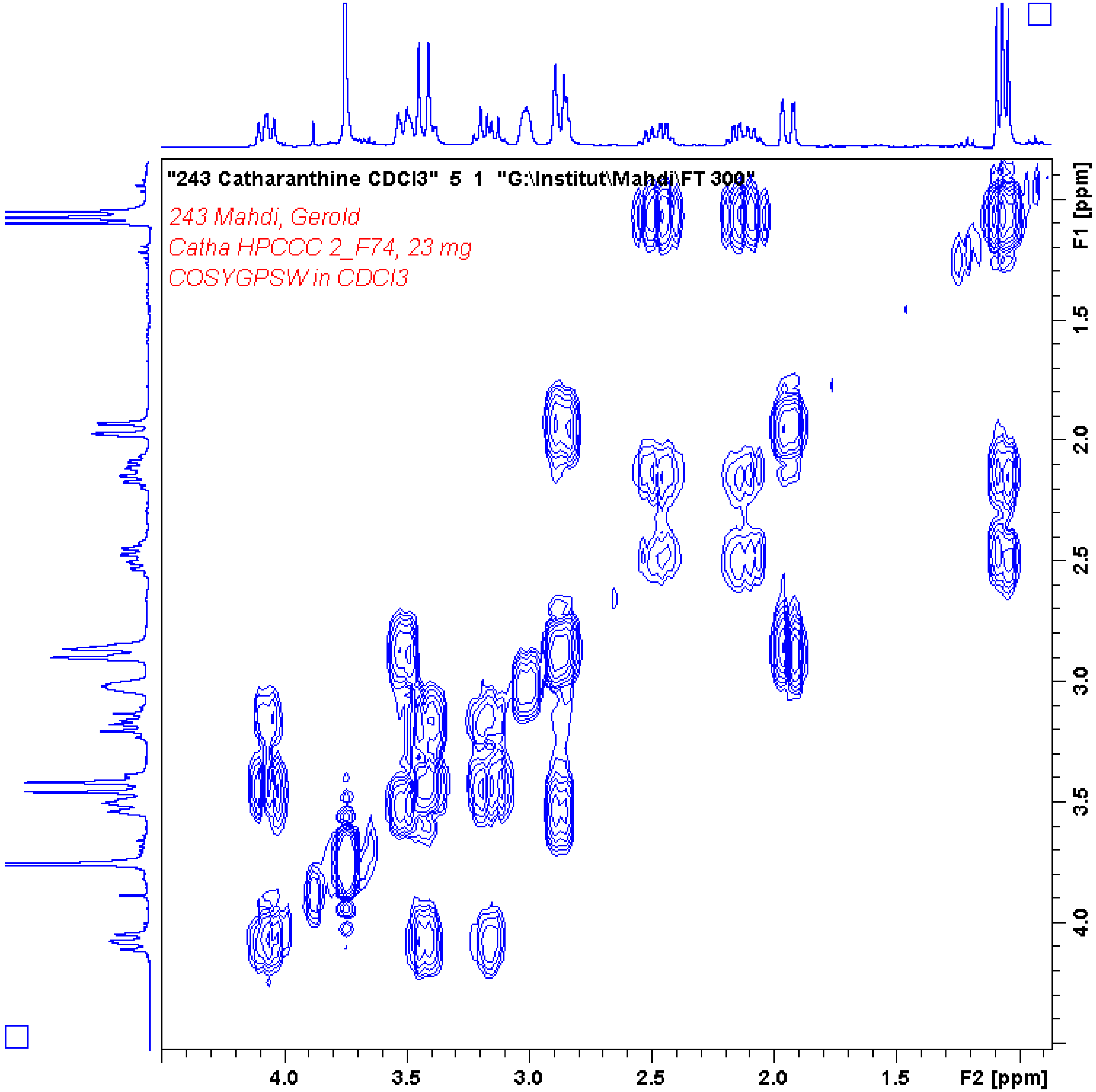

Figure NMR-S2

HSQC phase edited ^{1}J-HC

Catharanthine (*337-f*) in $CDCl_3$

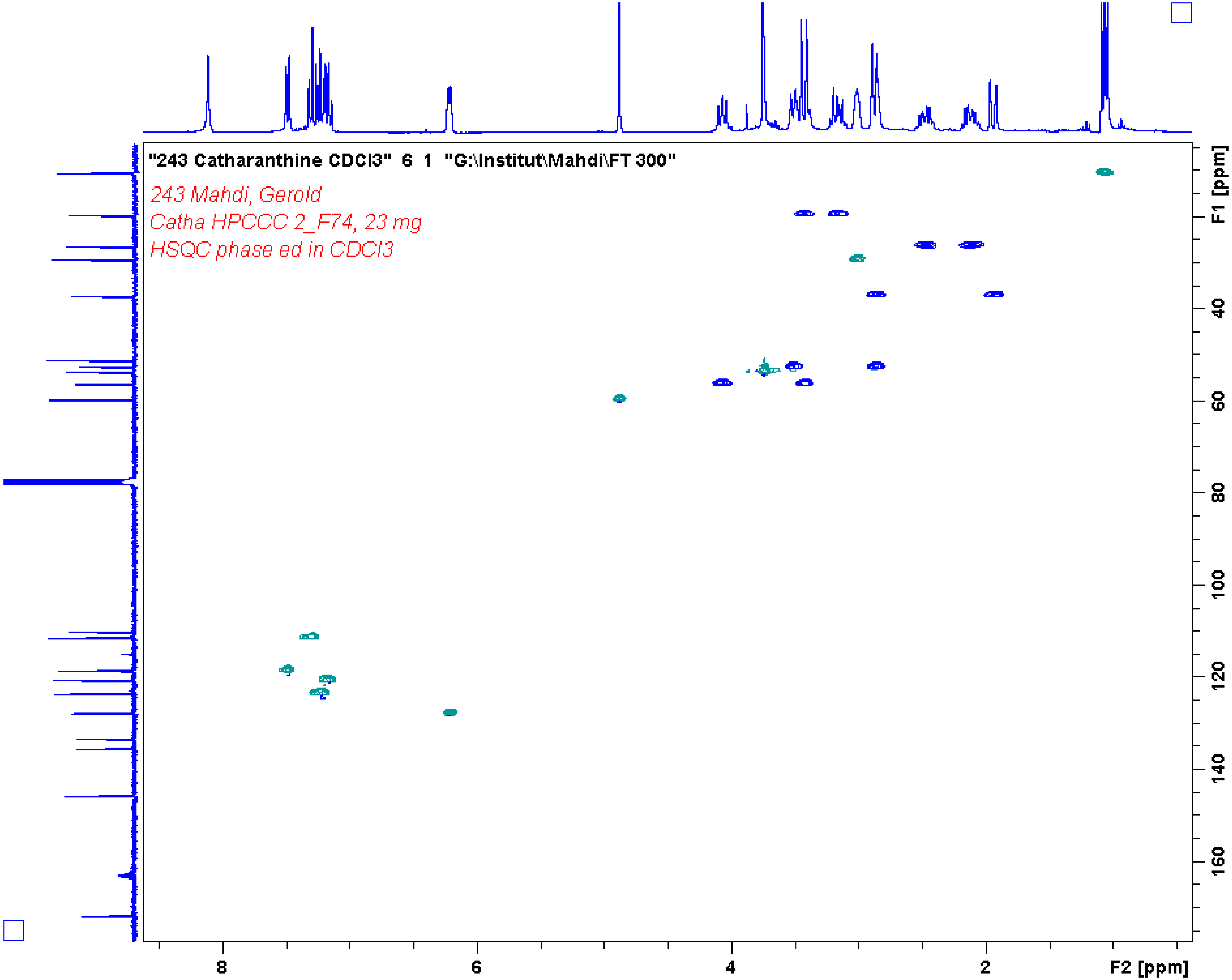

Figure NMR-S2

HSQC phase edited ¹J-HC

Catharanthine (*337-f*) in $CDCl_3$

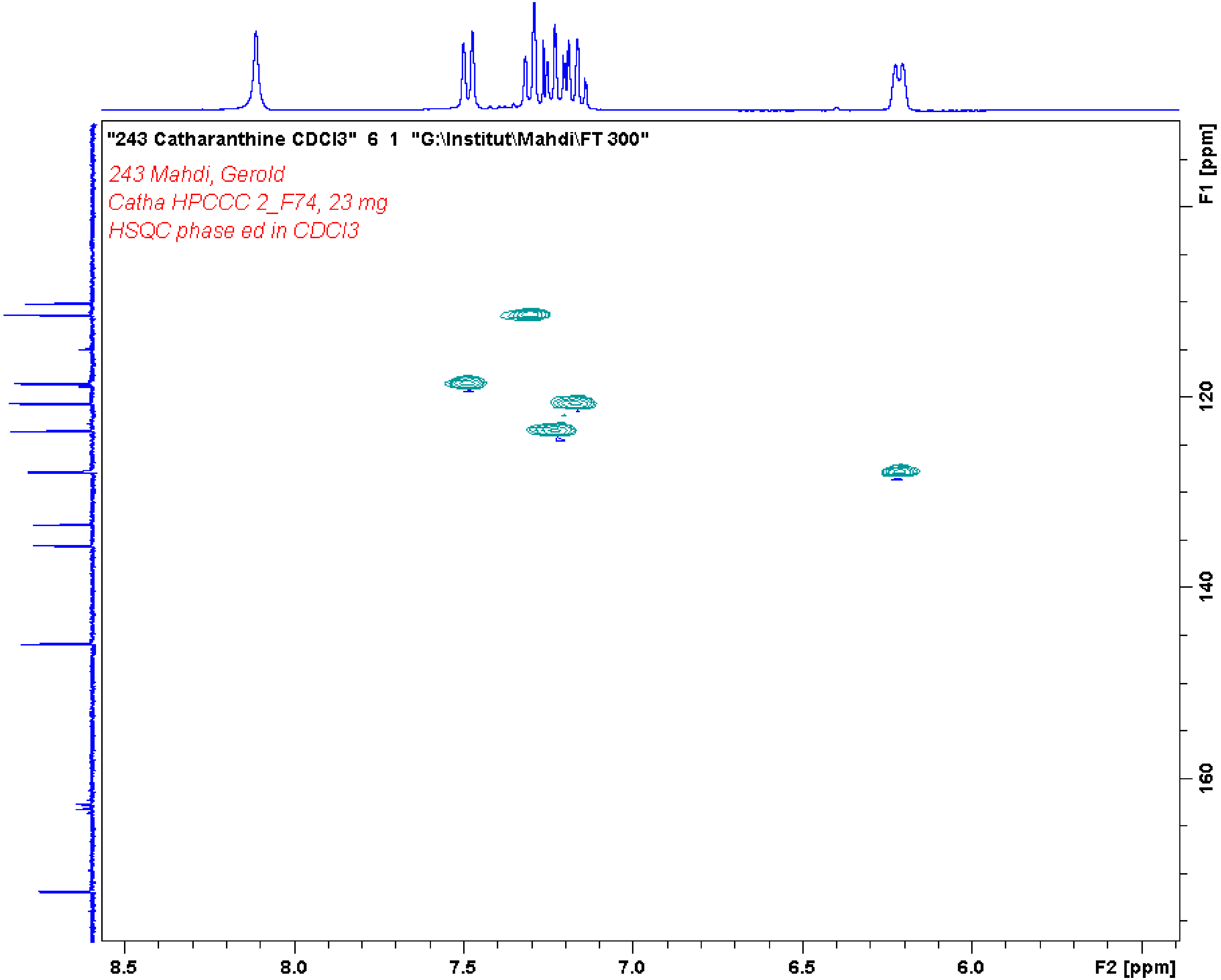

<u>**Figure NMR-S2**</u>

HSQC phase edited [1]*J-HC*

Catharanthine (*337-f*)
in $CDCl_3$

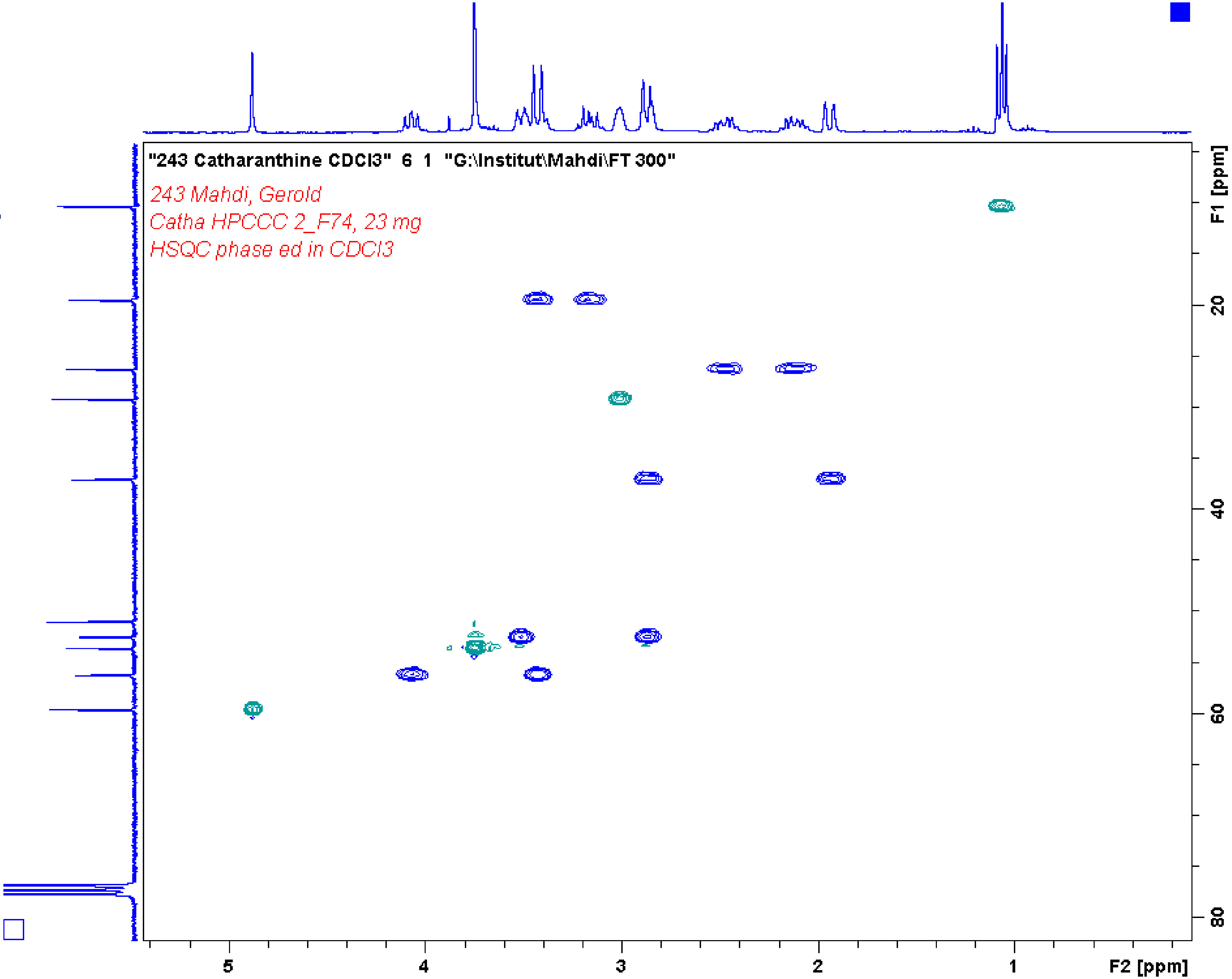

Figure NMR-S2

HMBC - long-range $^{2,3}J$-HC
Catharanthine (*337-f*)
in $CDCl_3$

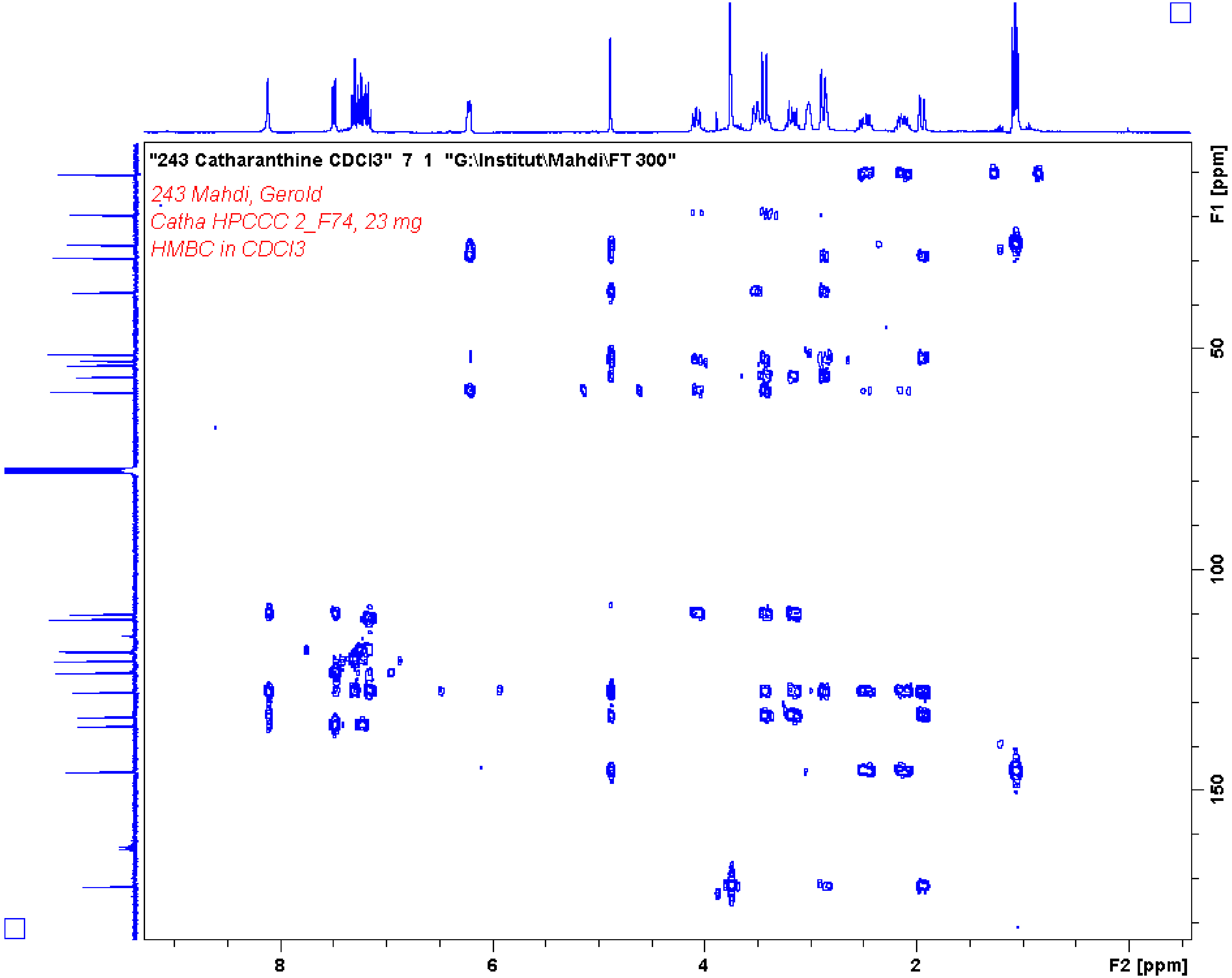

Figure NMR-S2

HMBC - long-range $^{2,3}J$-H(

Catharanthine (*337-f*)

in $CDCl_3$

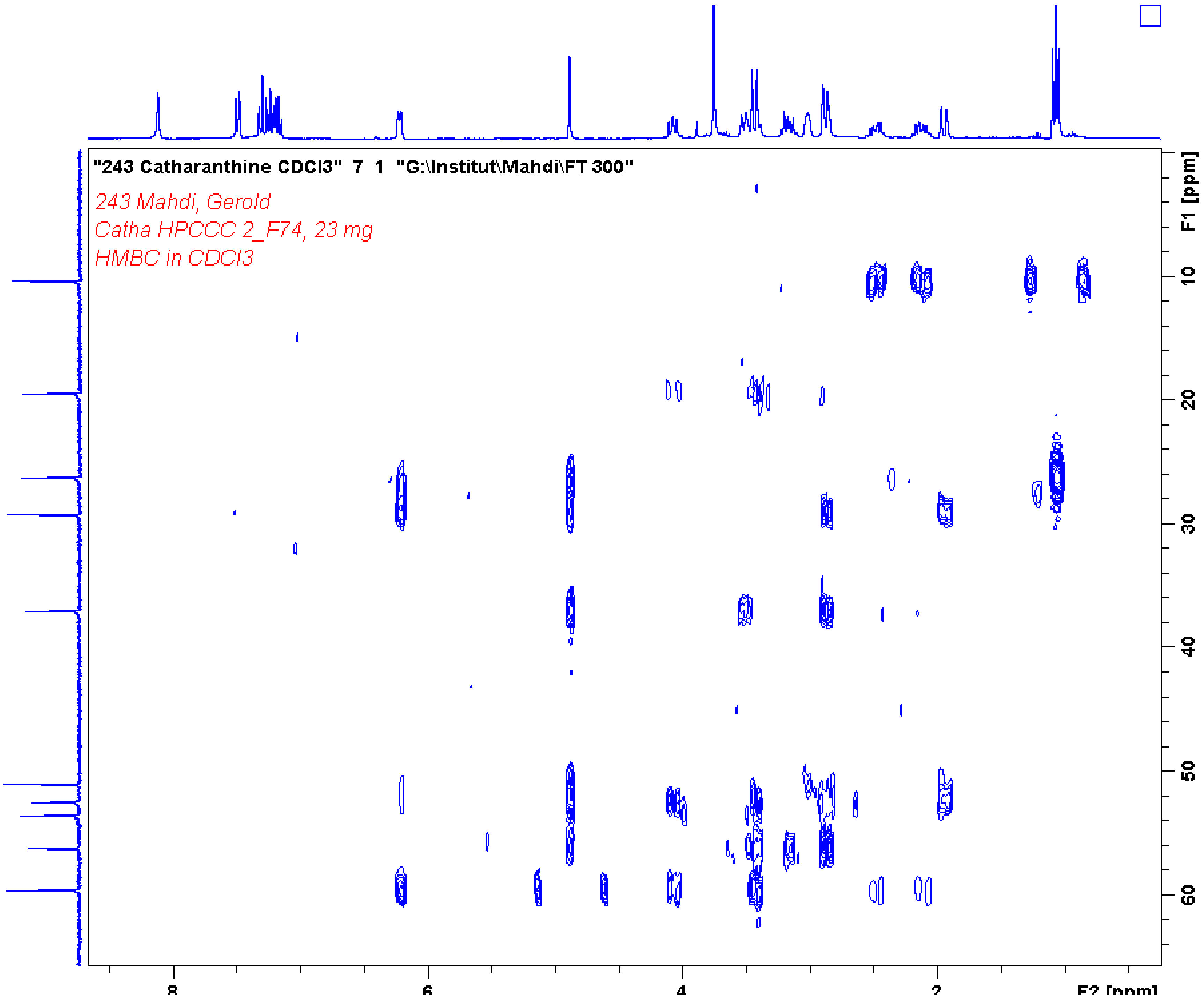

Figure NMR-S2

HMBC - long-range ^{2,3}*J-HC*
Catharanthine (*337-f*)
in $CDCl_3$

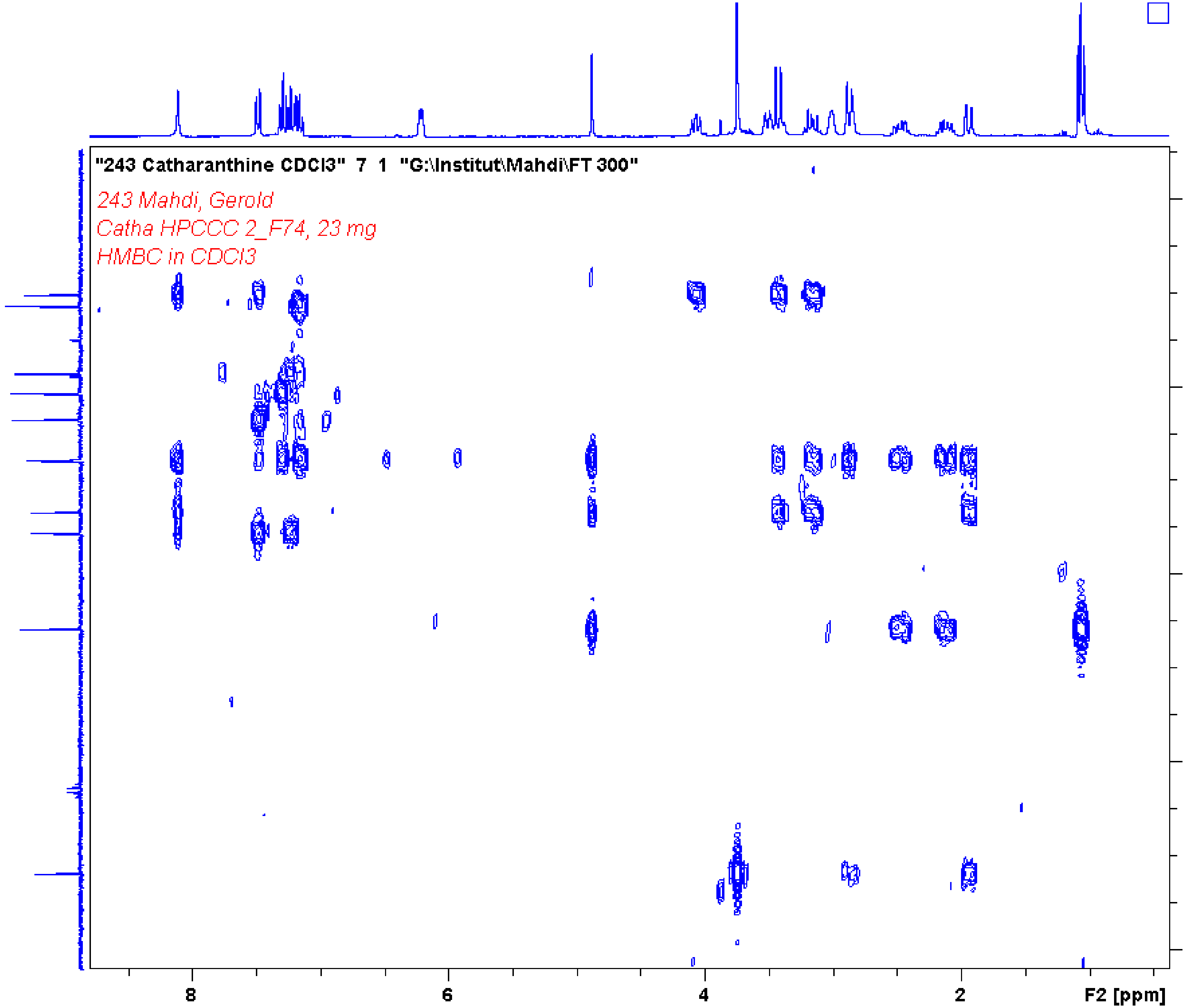

Figure NMR-S2

HMBC - long-range $^{2,3}J$-*HC*
Catharanthine (*337-f*)
in $CDCl_3$

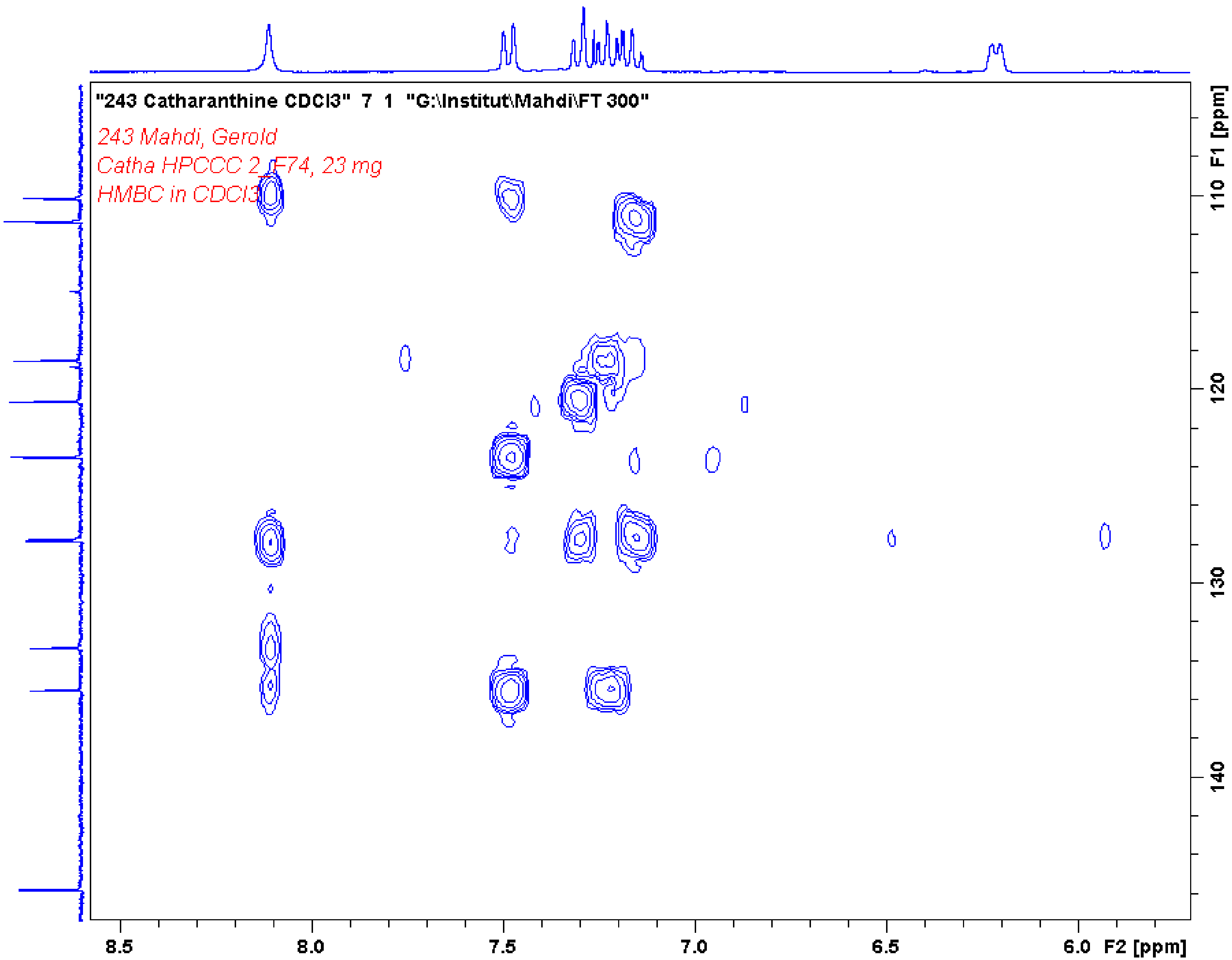

Figure NMR-S2

$^{1}H/^{1}H$-NOESY – Catharanthine (*337-f*) in $CDCl_3$

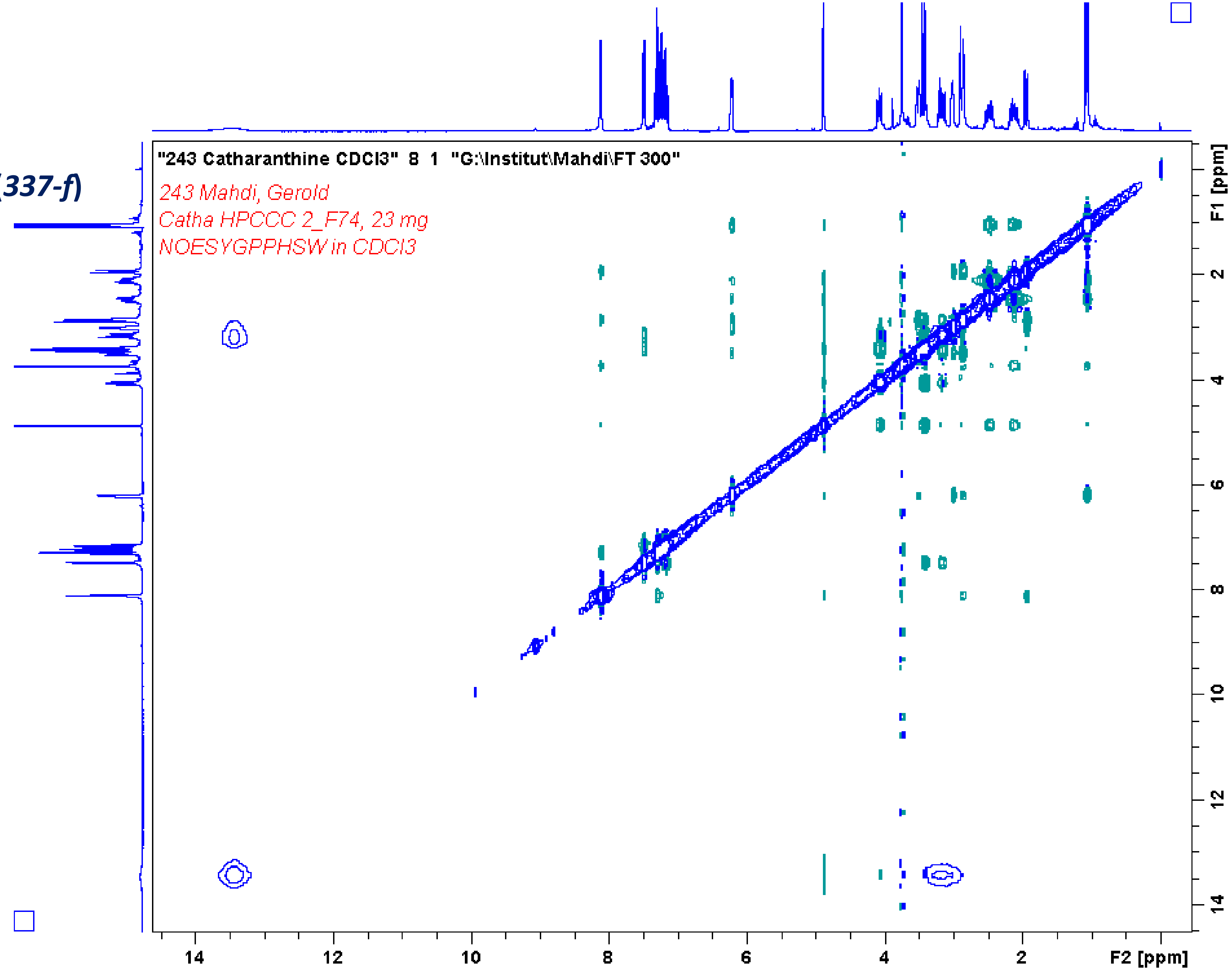

Figure NMR-S2

$^1H/^1H$-NOESY – Catharanthine (*337-f*) in $CDCl_3$

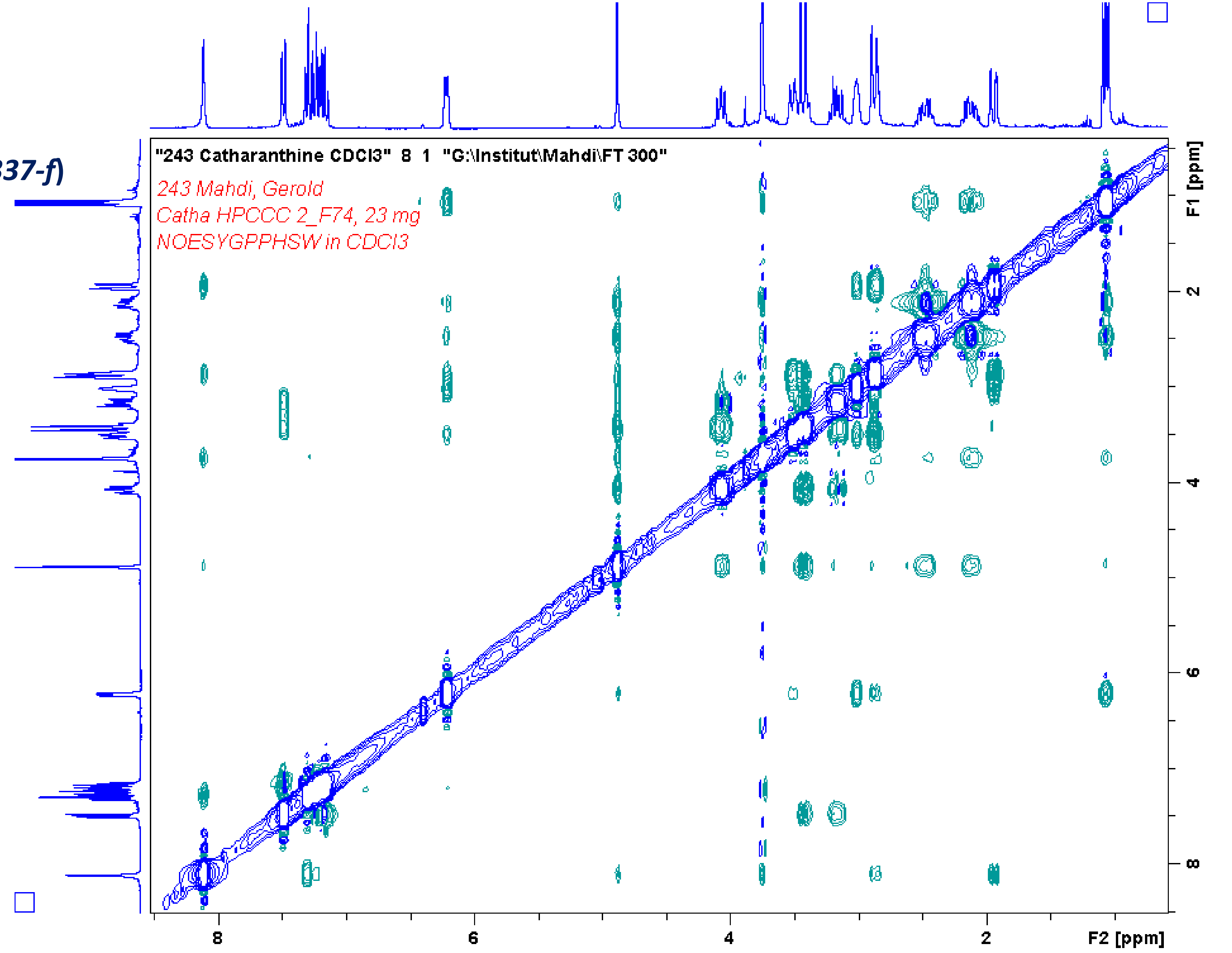

Figure NMR-S3

^{1}H NMR – Akuammicine (*323-j*) in $CDCl_3$ (300 MHz)

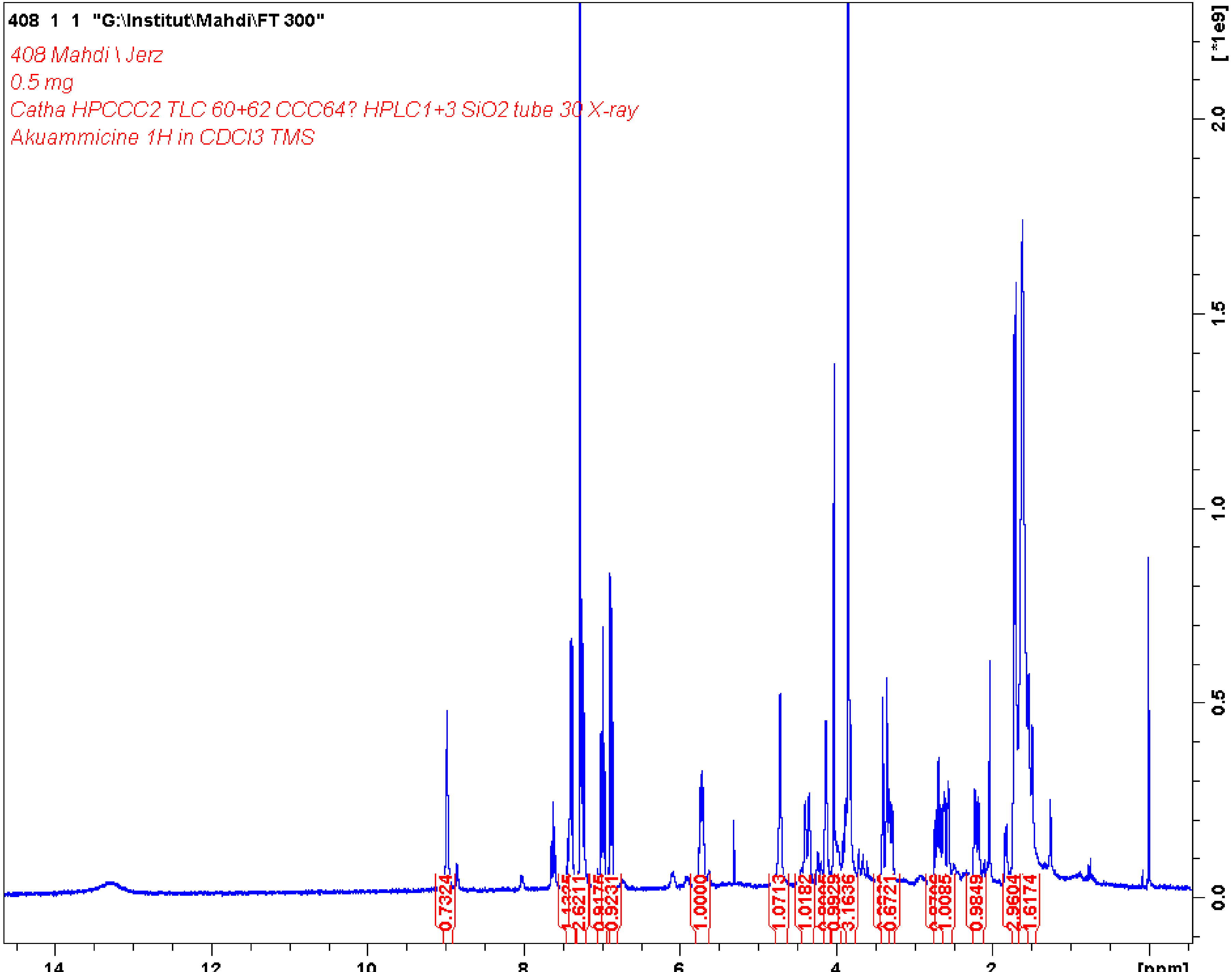

<u>**Figure NMR-S3**</u>

^{1}H NMR – Akuammicine (*323-j*)
in $CDCl_3$
(300 MHz)

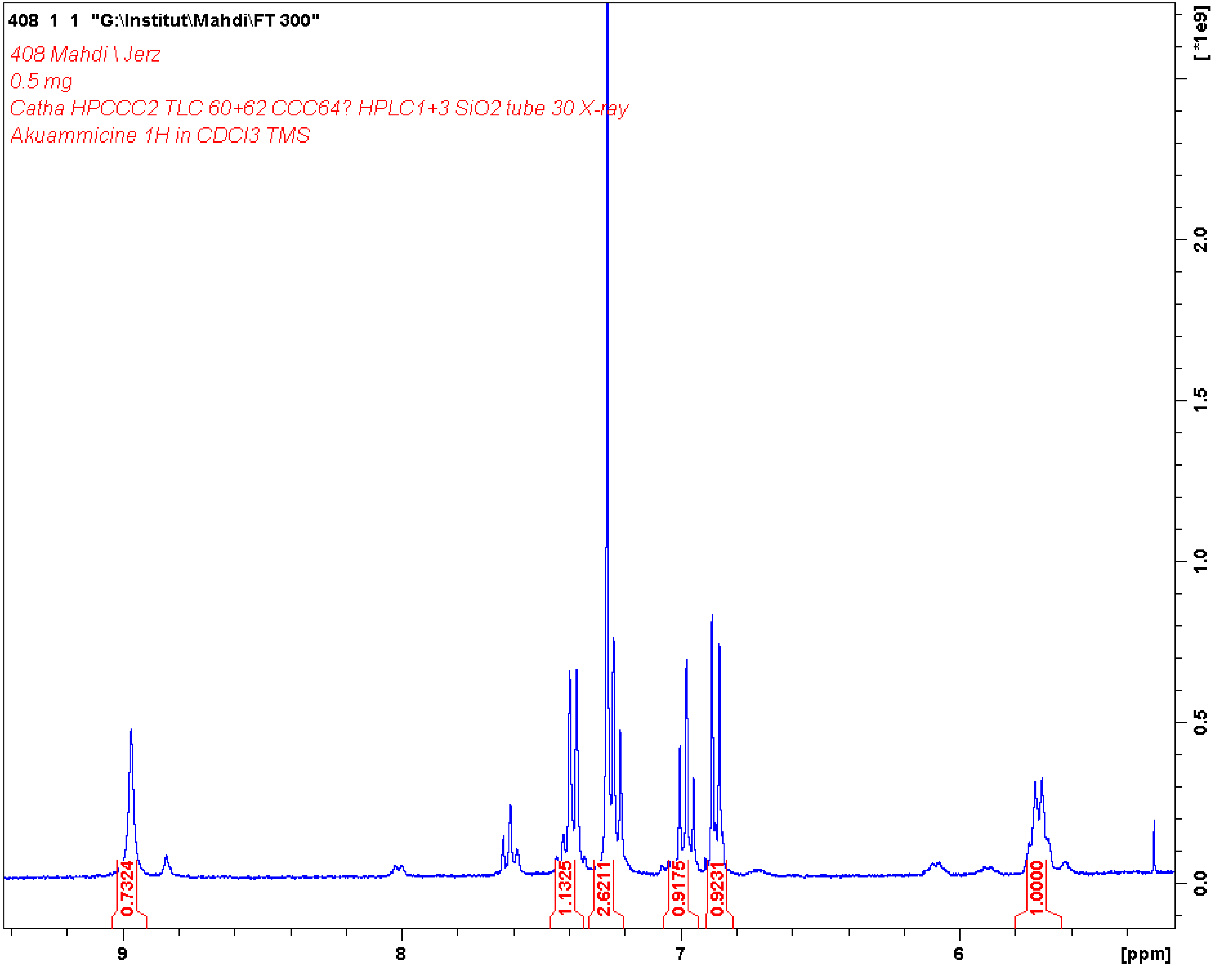

Figure NMR-S3

^{1}H NMR – Akuammicine (*323-j*) in $CDCl_3$ (300 MHz)

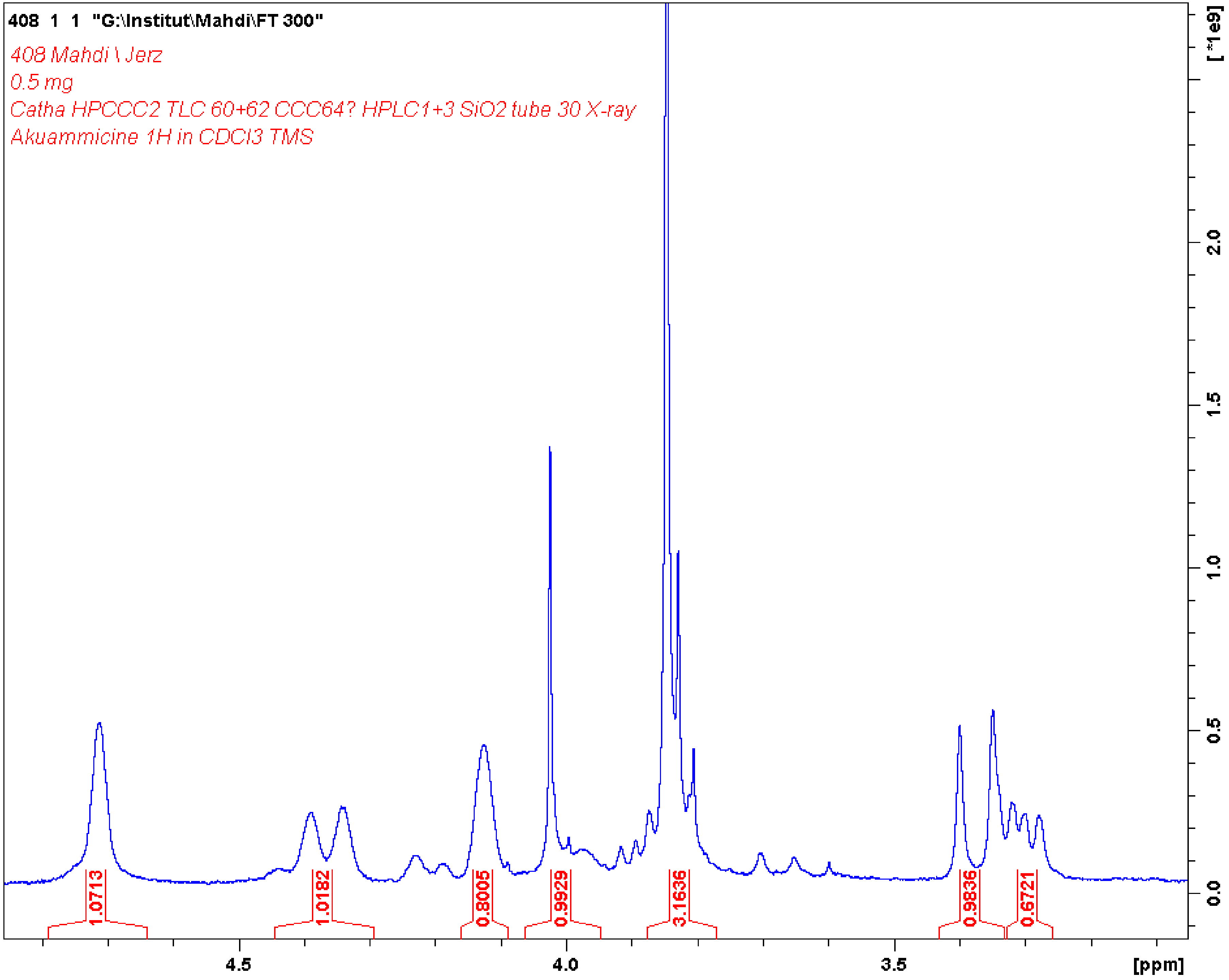

Figure NMR-S3

^{1}H NMR – Akuammicine (*323-j*)
in $CDCl_3$
(300 MHz)

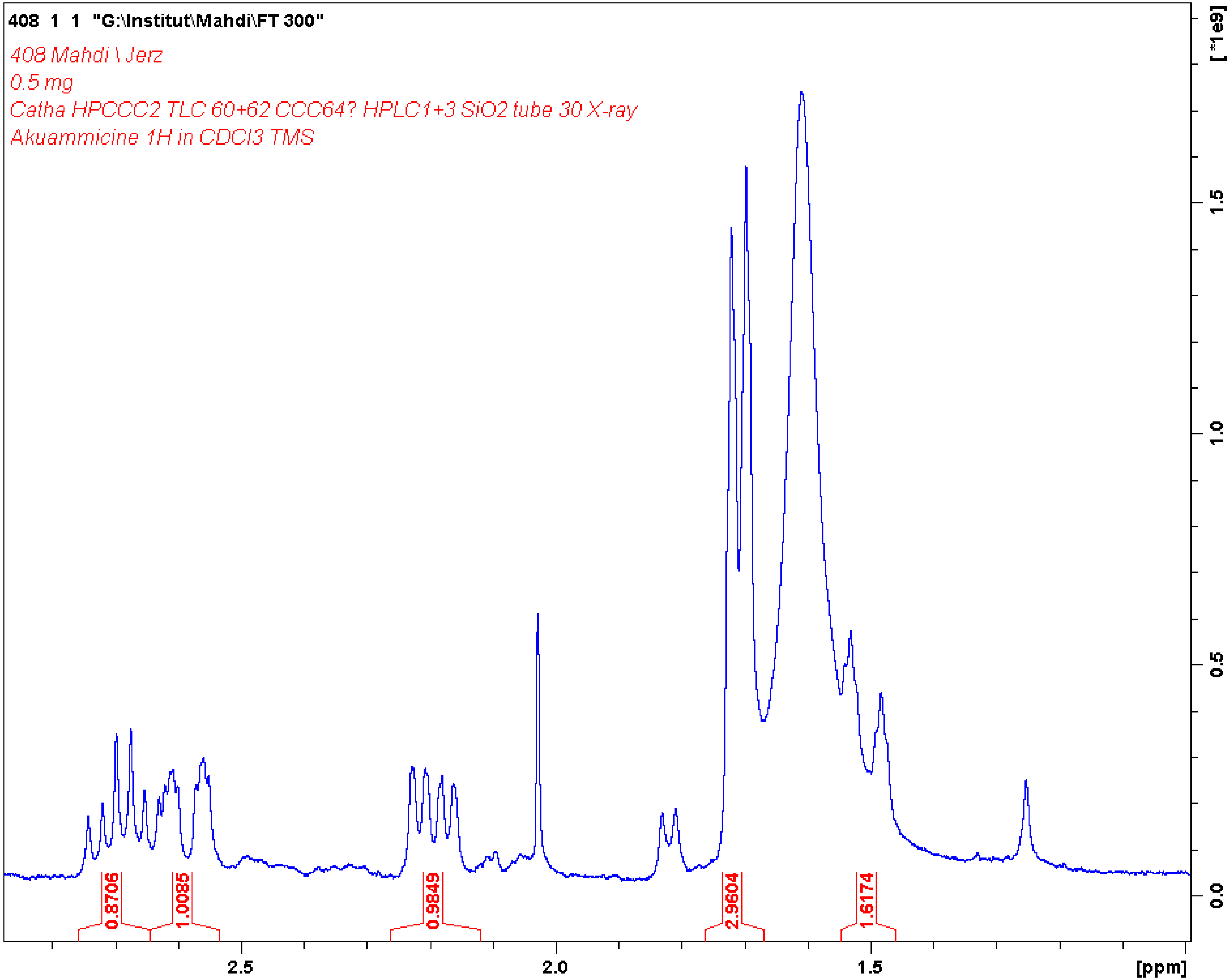

Figure NMR-S3

^{13}C NMR – Akuammicine (*323-j*) in $CDCl_3$ (75 MHz)

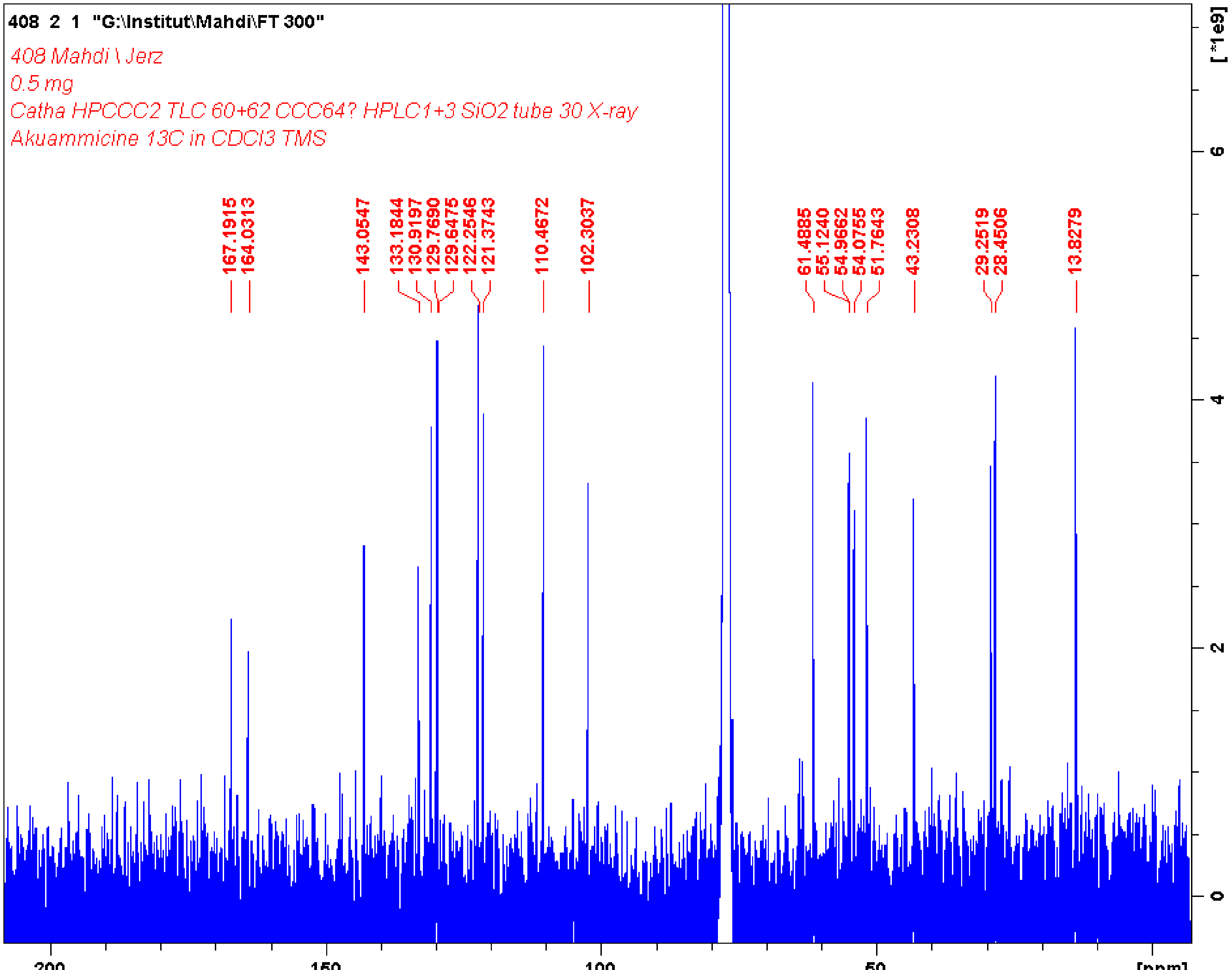

Figure NMR-S3

^{13}C NMR – Akuammicine (*323-j*) in $CDCl_3$ (75 MHz)

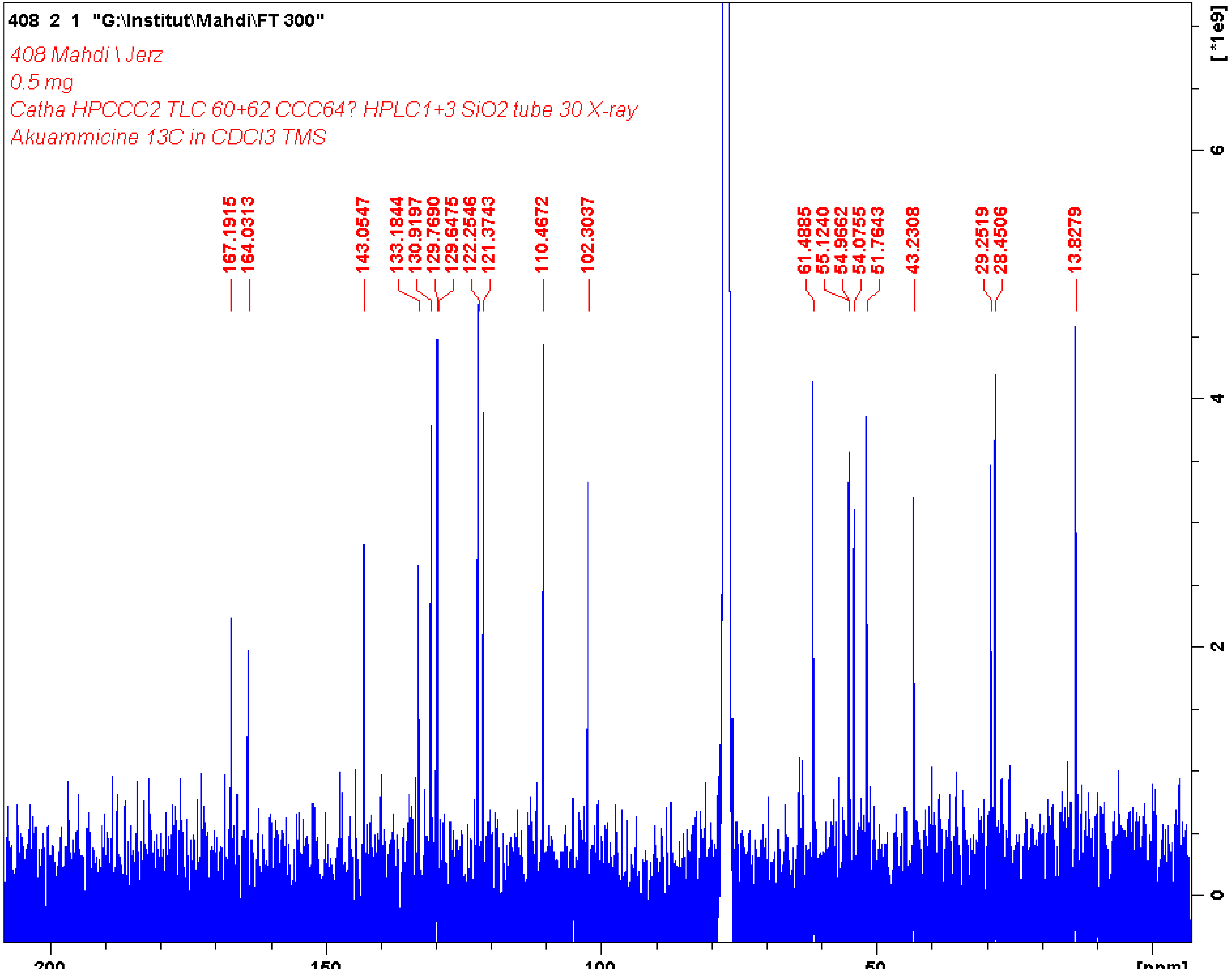

Figure NMR-S3

DEPT135 – Akuammicine (*323-j*) in $CDCl_3$ (75 MHz)

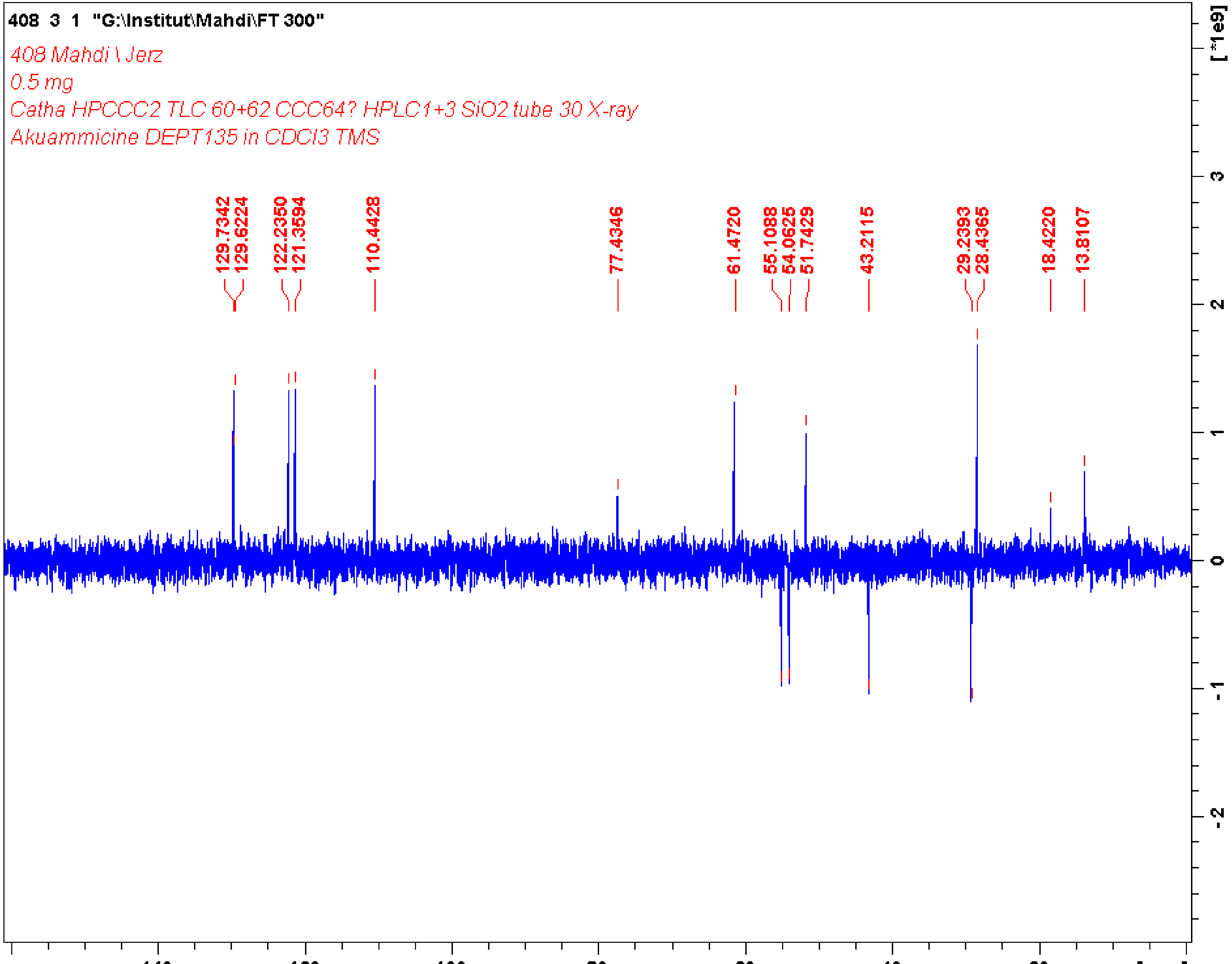

Figure NMR-S3

$^{1}H/^{1}H$-COSY
– Akuammicine (*323-j*)
in $CDCl_3$

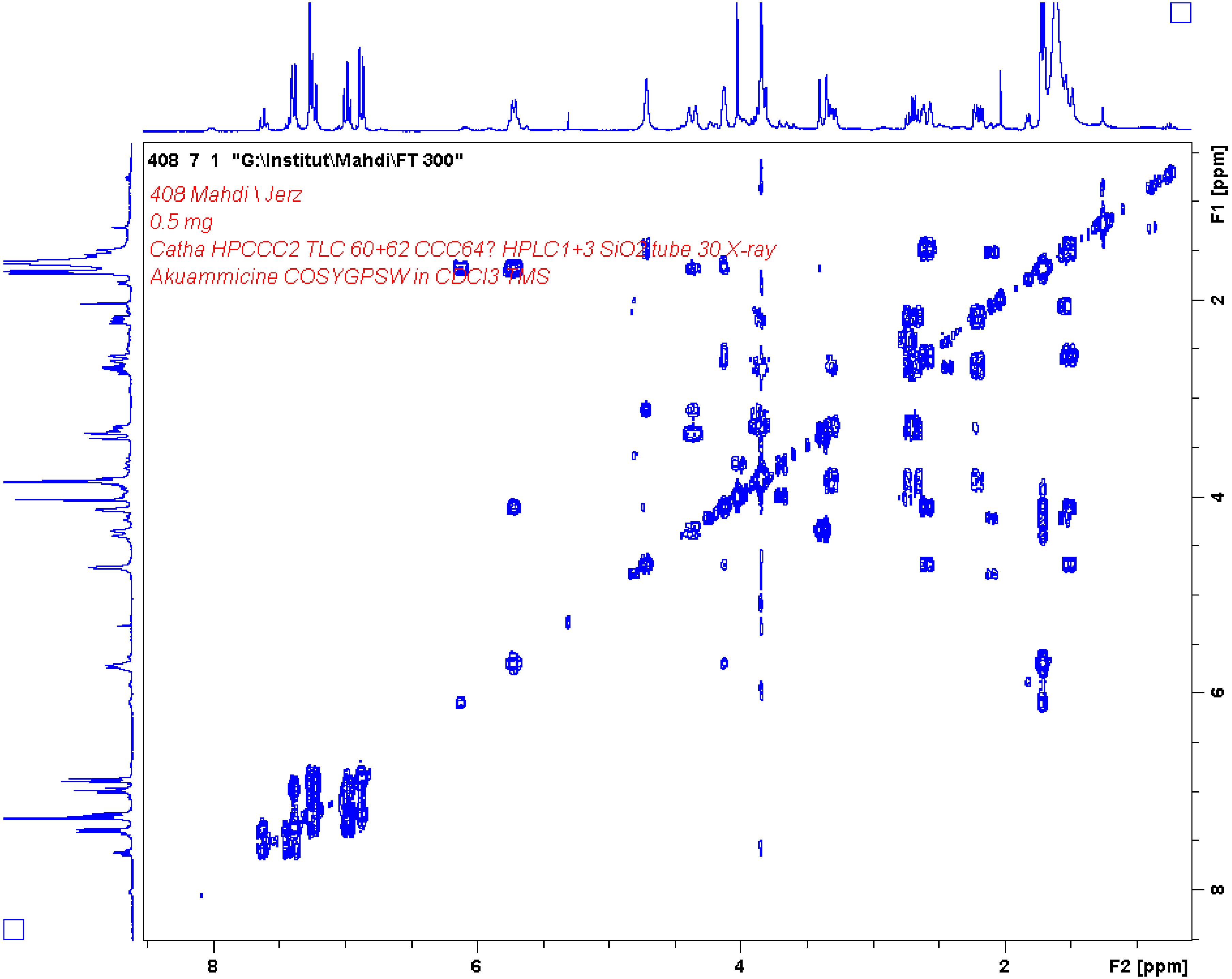

Figure NMR-S3

$^1H/^1H$-COSY – Akuammicine (*323-j*) in $CDCl_3$

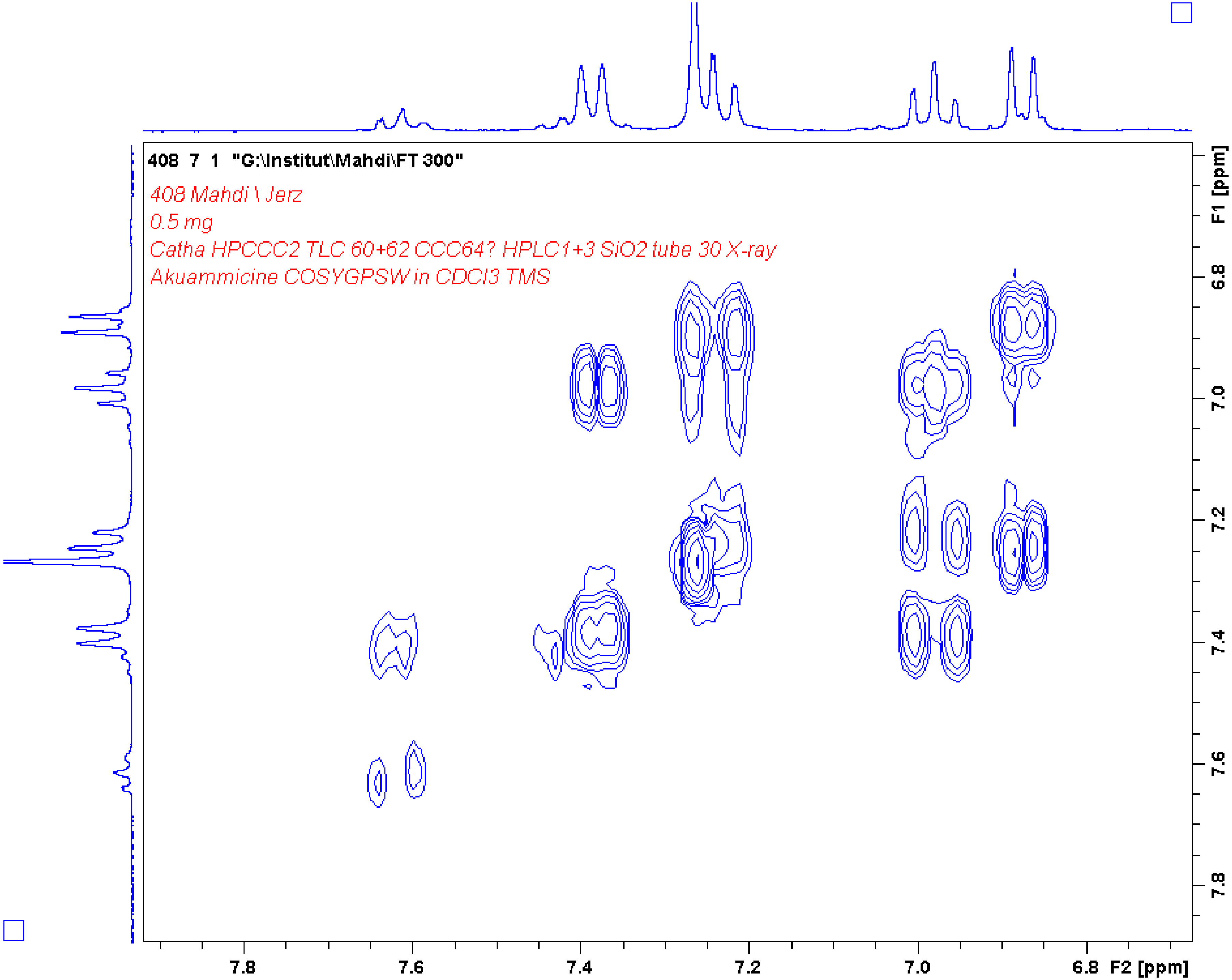

Figure NMR-S3

$^1H/^1H$-COSY
– Akuammicine (*323-j*)
in $CDCl_3$

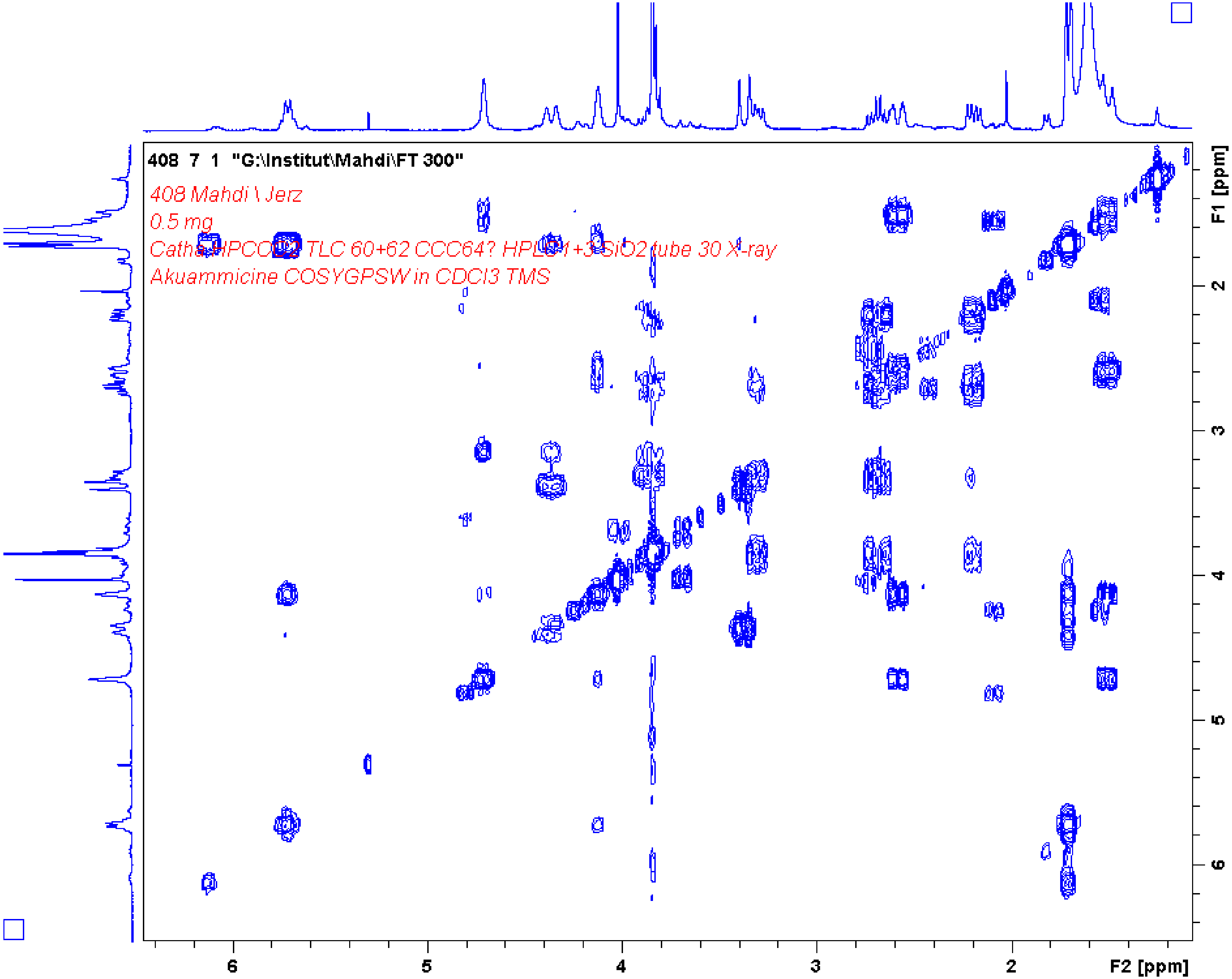

Figure NMR-S3

HSQC phase edited ^{1}J-HC

Akuammicine (*323-j*) in $CDCl_3$

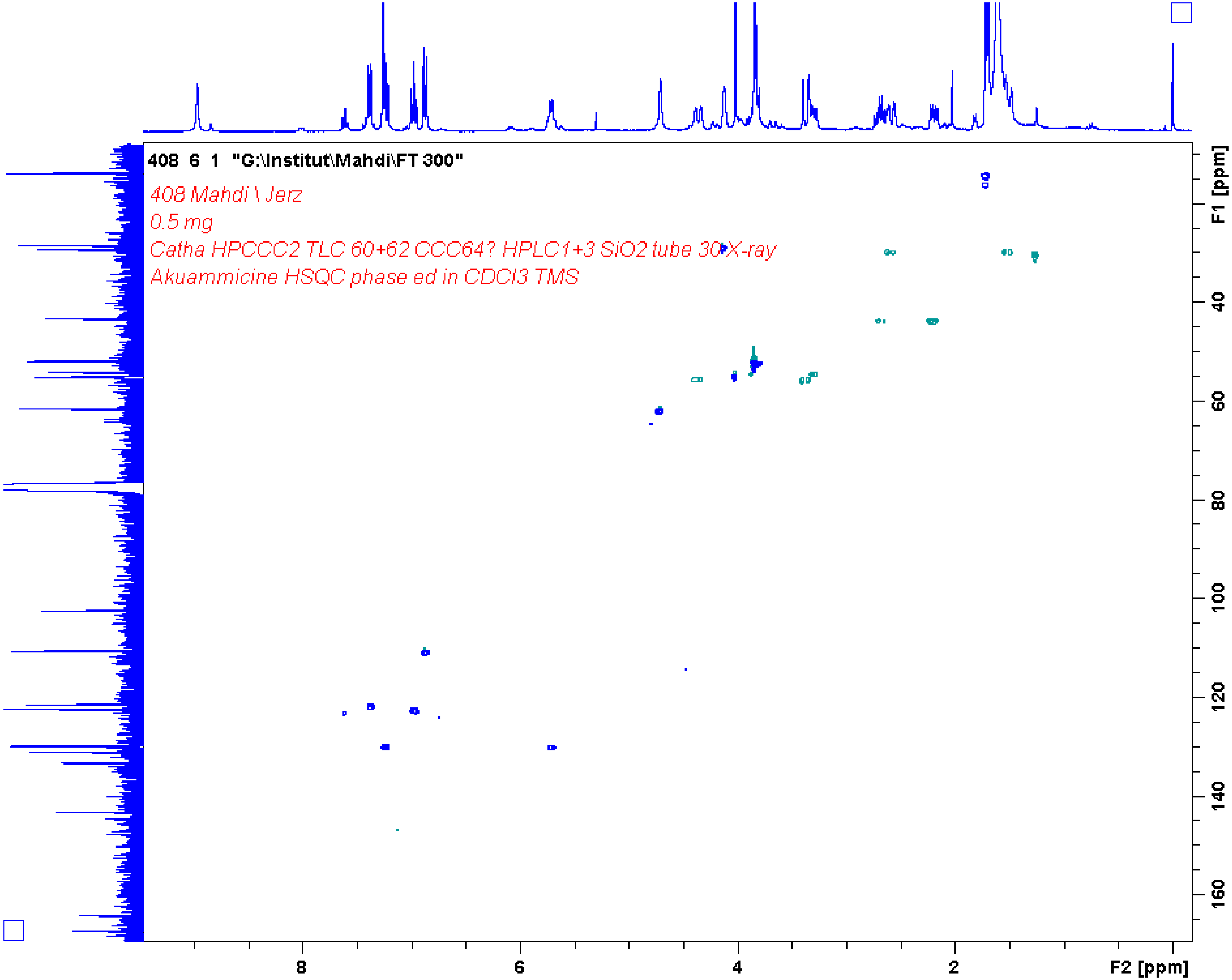

Figure NMR-S3

HSQC phase edited ^{1}J-HC

Akuammicine (*323-j*)
in $CDCl_3$

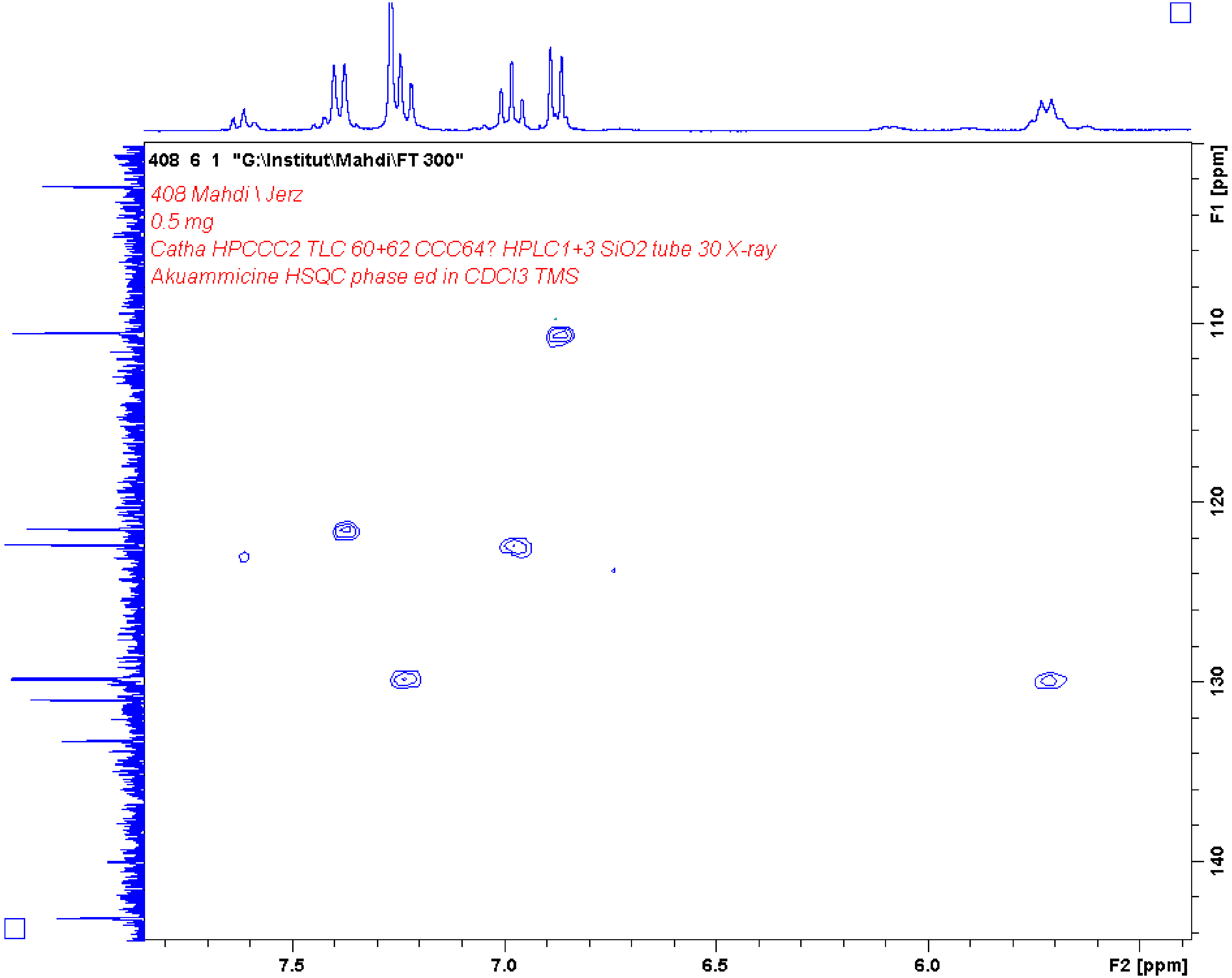

Figure NMR-S3

HSQC phase edited ^{1}J-HC

Akuammicine (*323-j*)
in $CDCl_3$

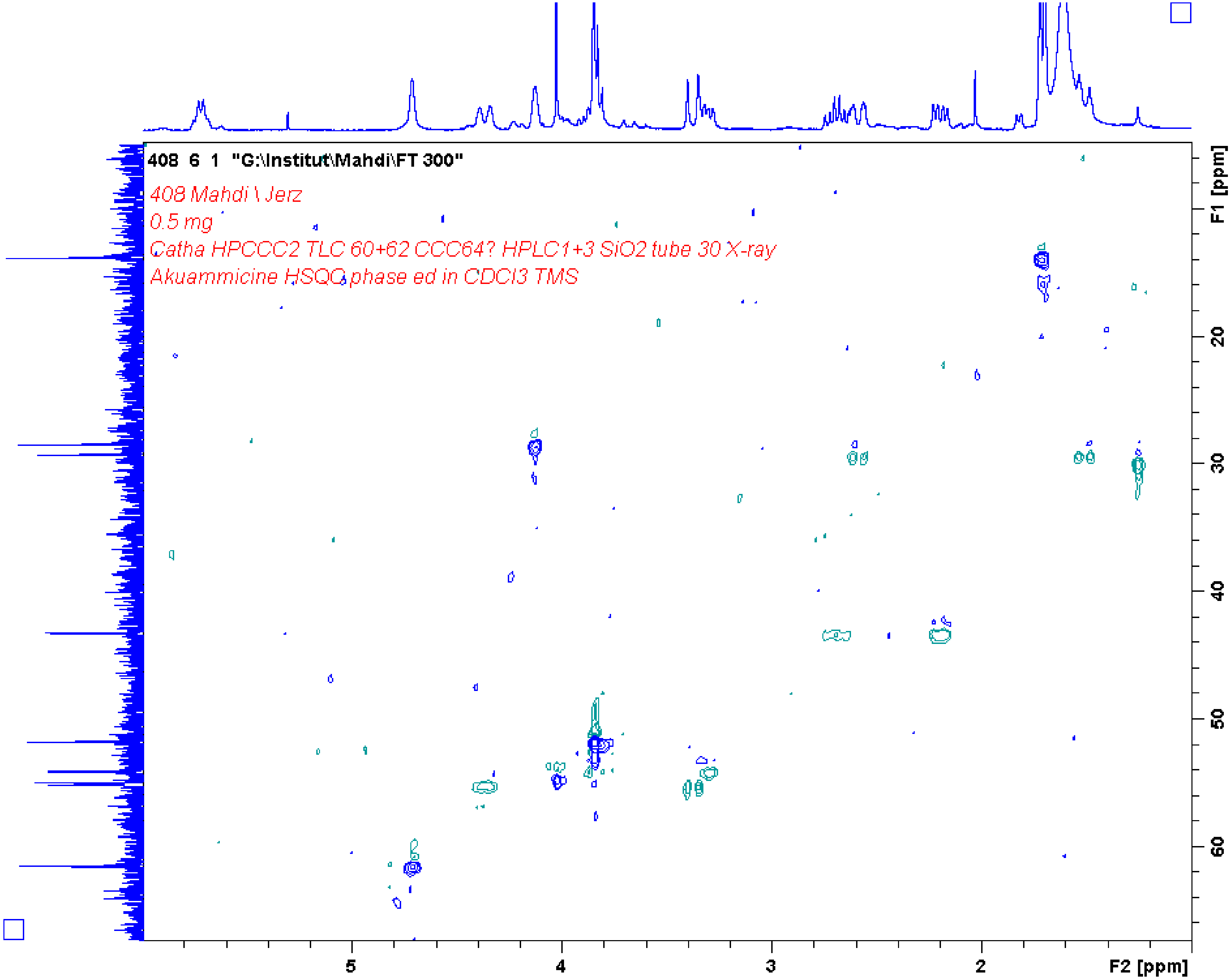

Figure NMR-S3

HMBC, long-range $^{2,3}J$-HC

Akuammicine (*323-j*) in $CDCl_3$

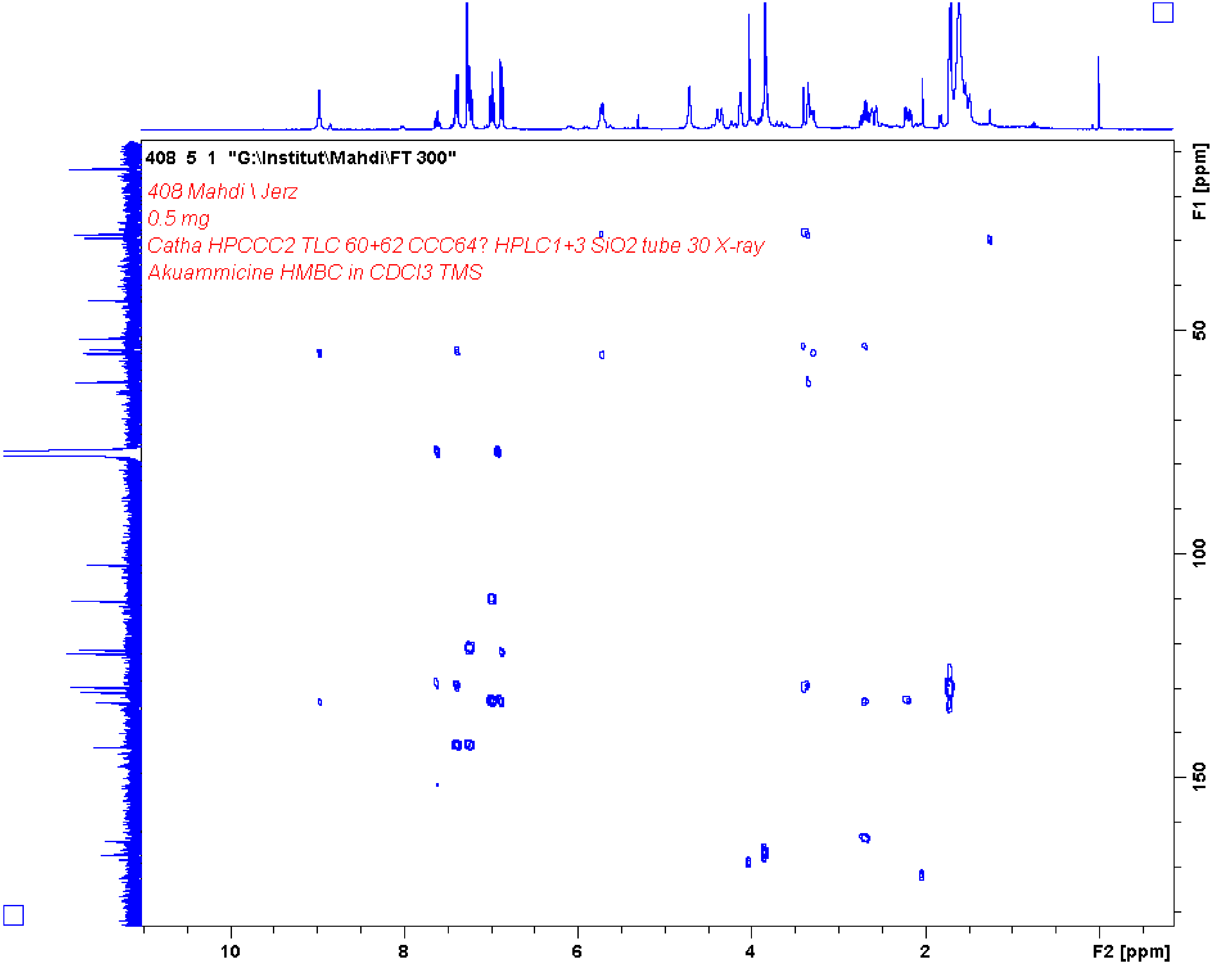

Figure NMR-S3

HMBC, long-range $^{2,3}J$-HC

Akuammicine (*323-j*) in $CDCl_3$

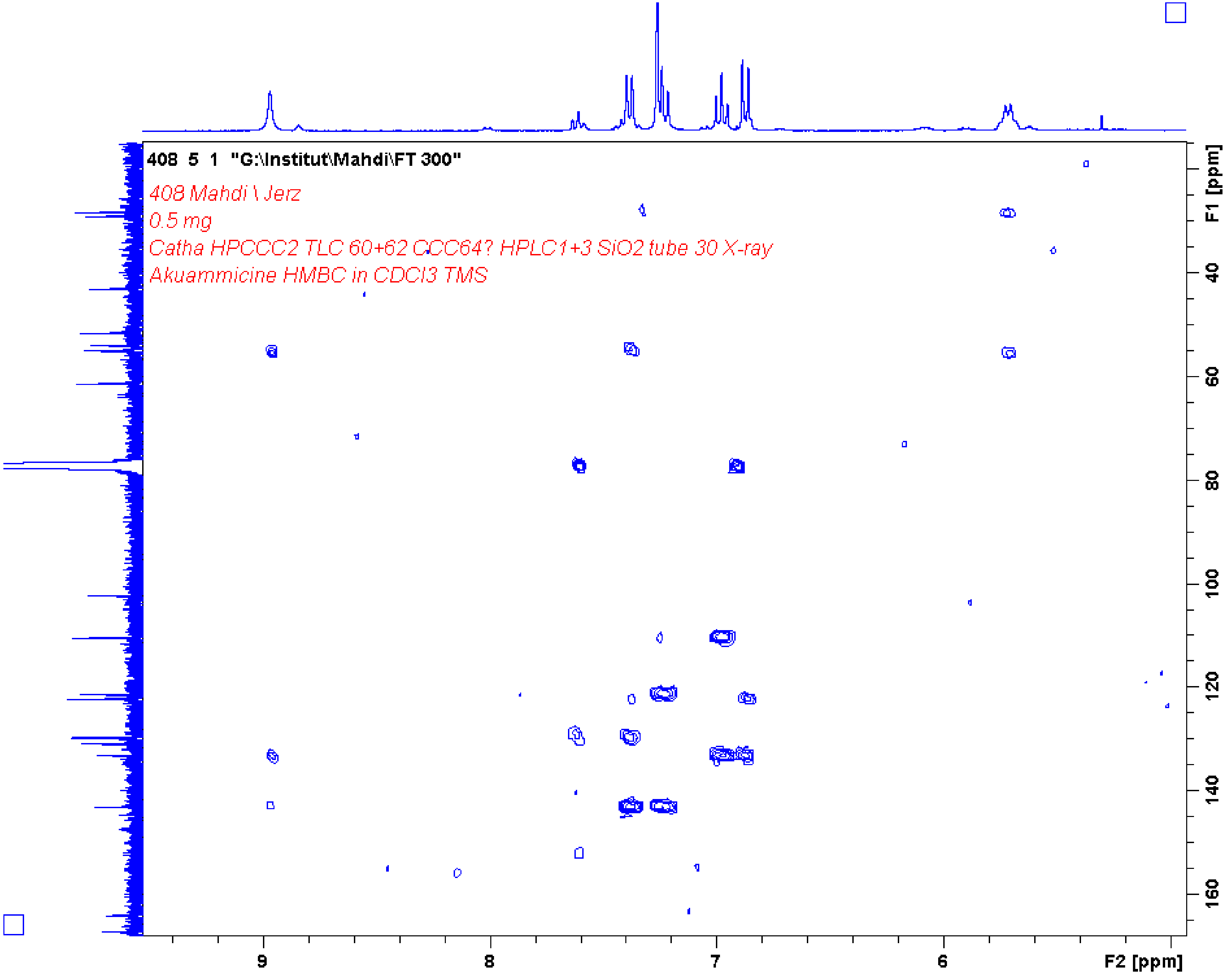

Figure NMR-S3

HMBC, long-range $^{2,3}J$-HC

Akuammicine (*323-j*)
in $CDCl_3$

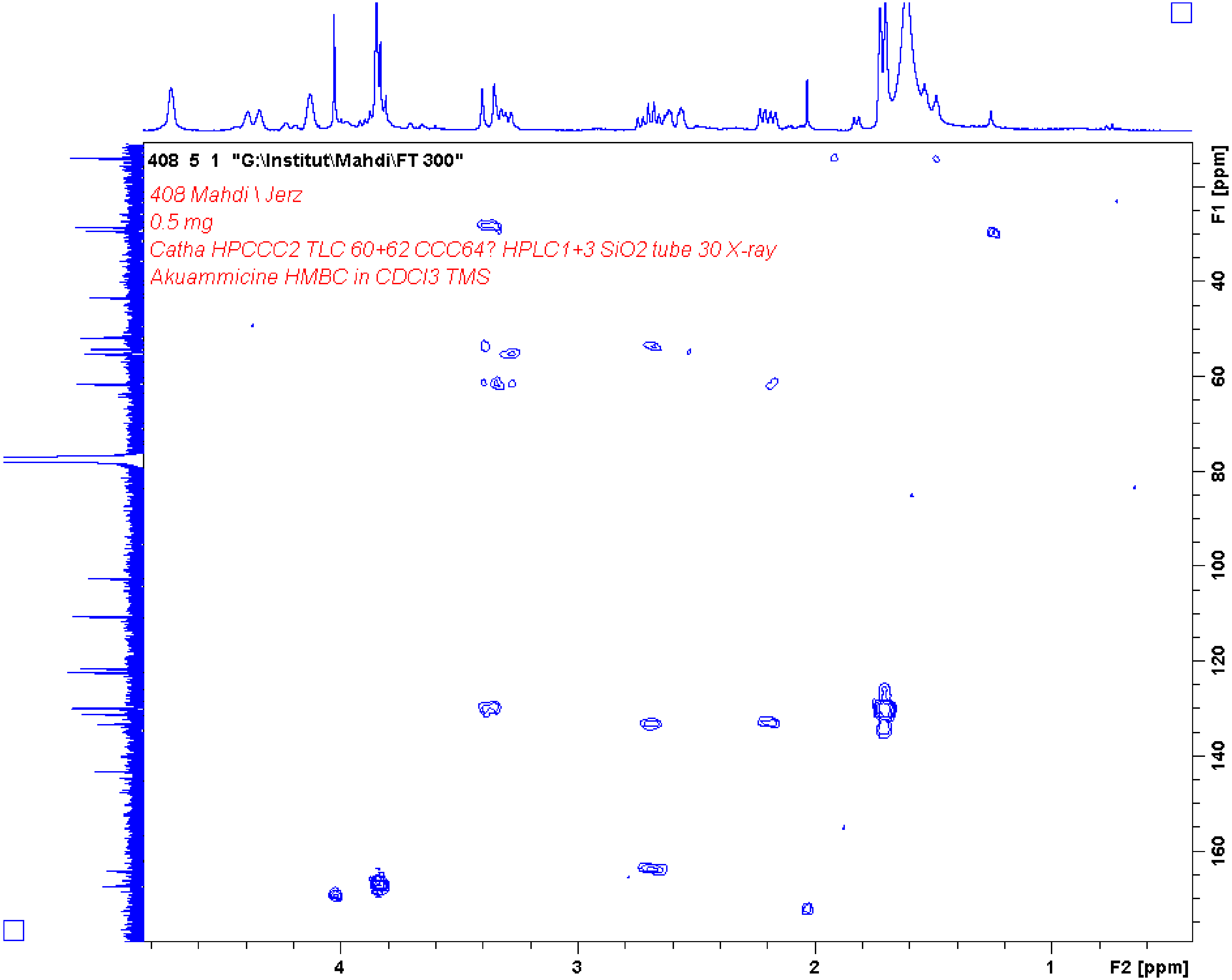

Figure NMR-S3

$^1H/^1H$-NOESY
– Akuammicine (*323-j*)
in $CDCl_3$

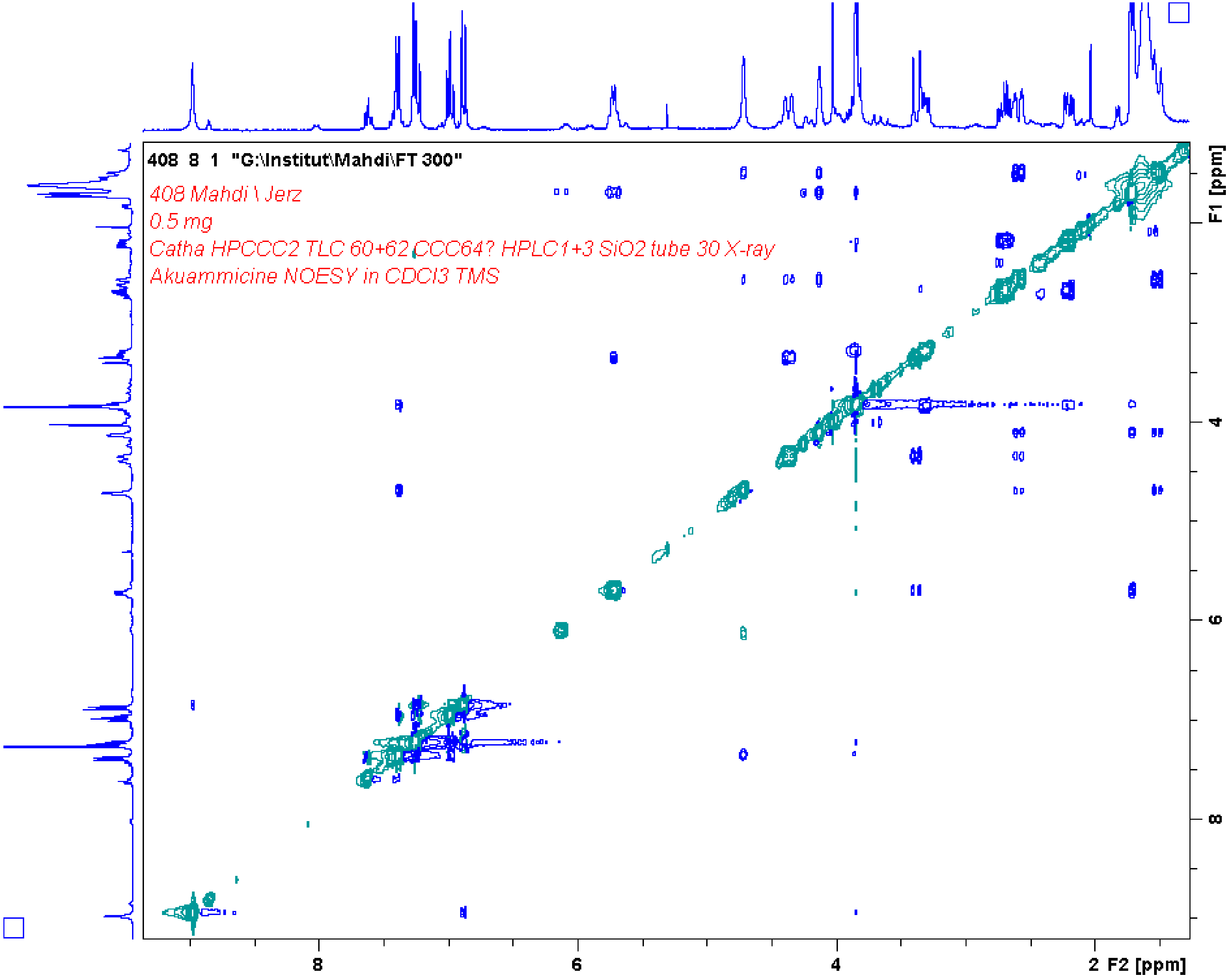

Figure NMR-S3

$^1H/^1H$-NOESY
– Akuammicine (*323-j*)
in $CDCl_3$

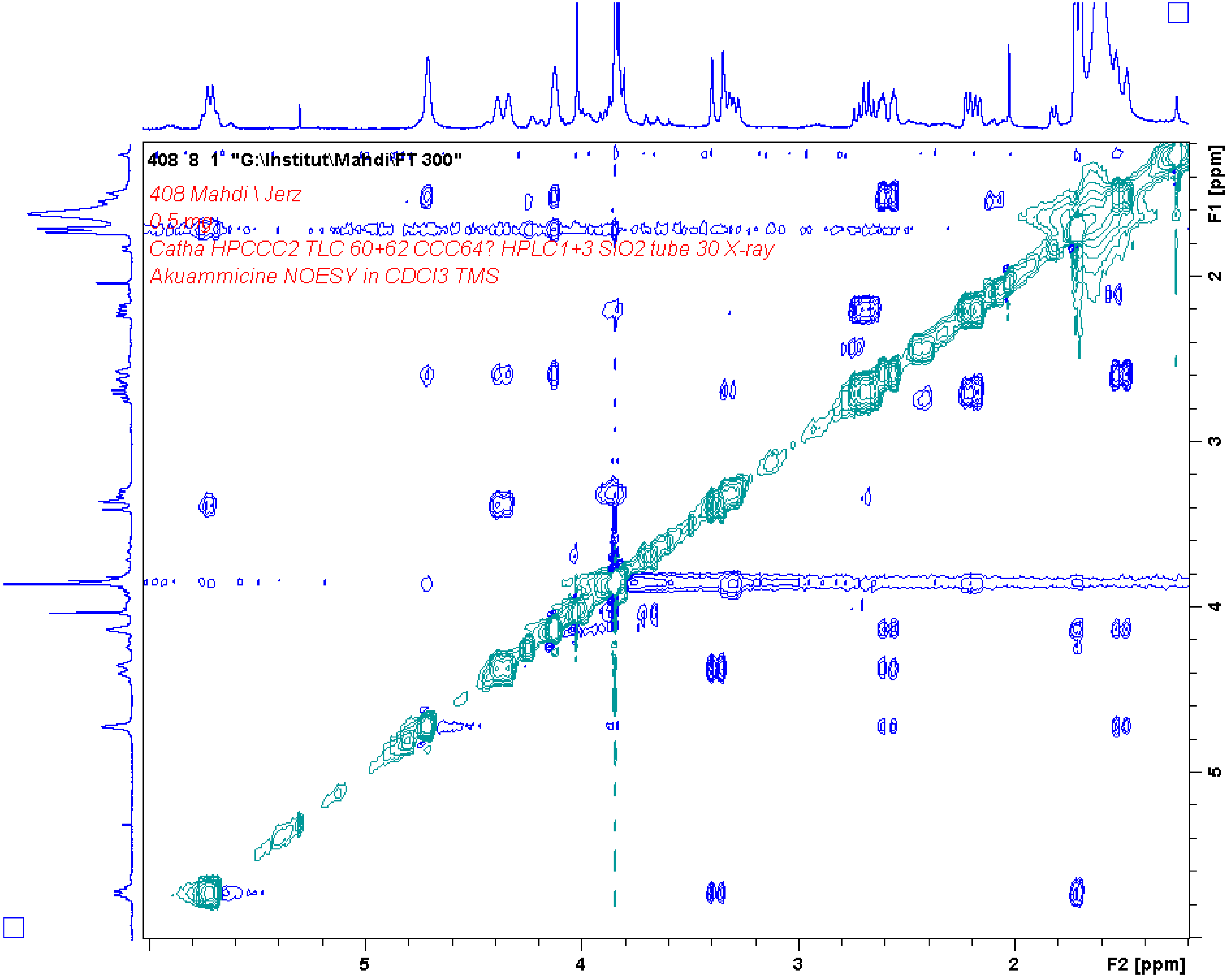

Figure NMR-S3

$^1H/^1H$-NOESY – Akuammicine (*323-j*) in $CDCl_3$

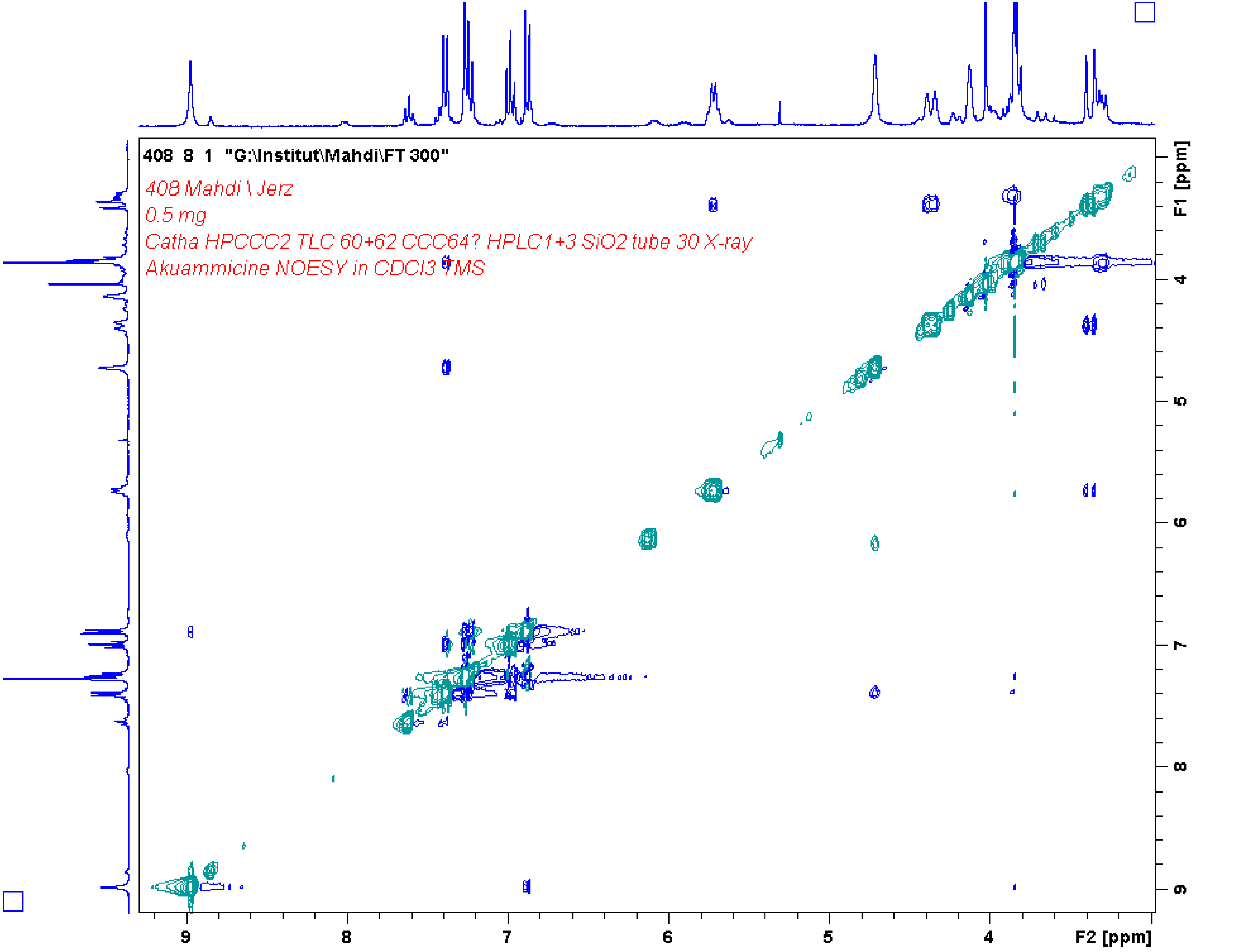

Figure NMR-S4

^{1}H NMR – Perivine (*329-a*)
in CD_3OD
(300 MHz)

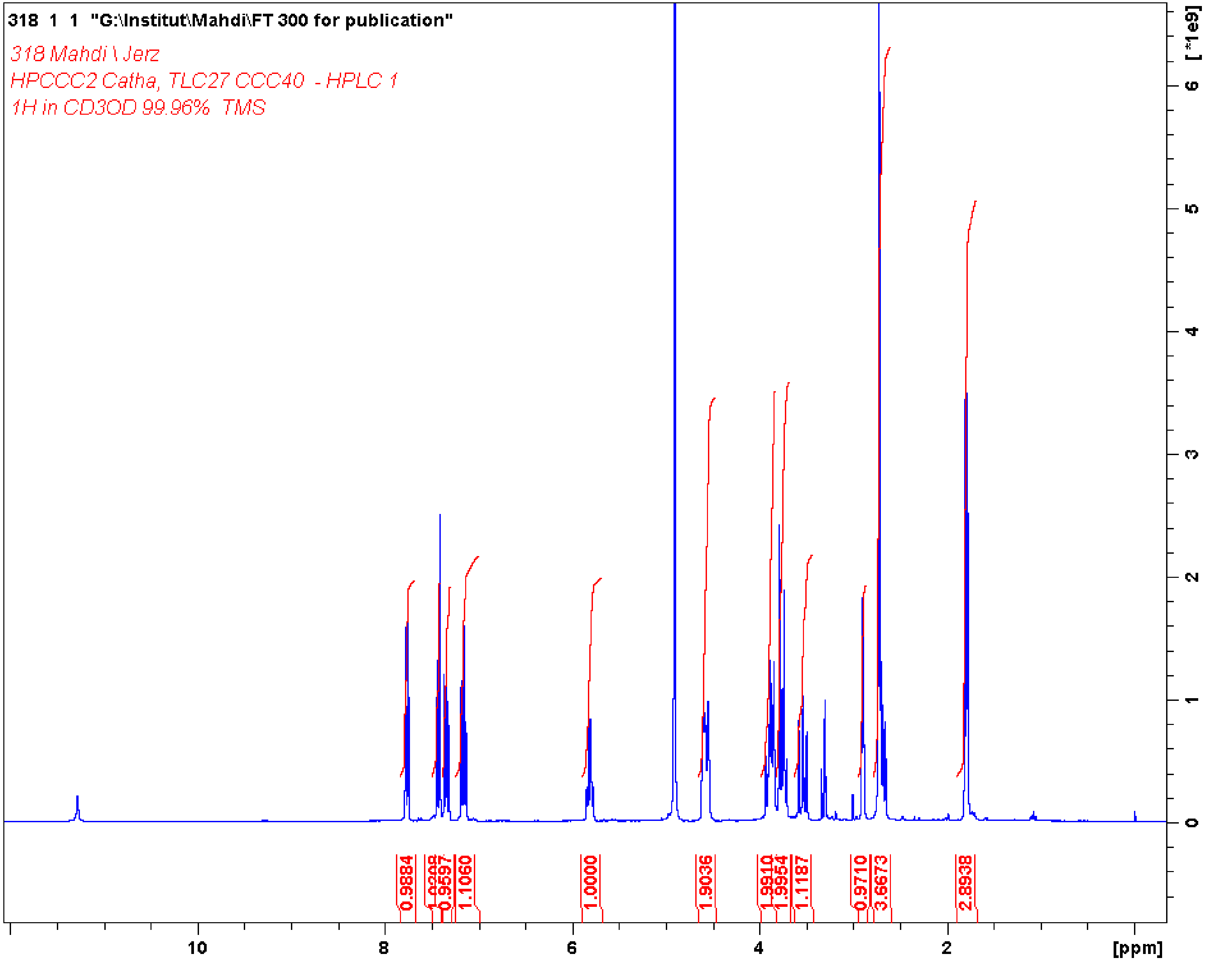

Figure NMR-S4

^{1}H NMR – Perivine (*329-a*)
in CD_3OD
(300 MHz)

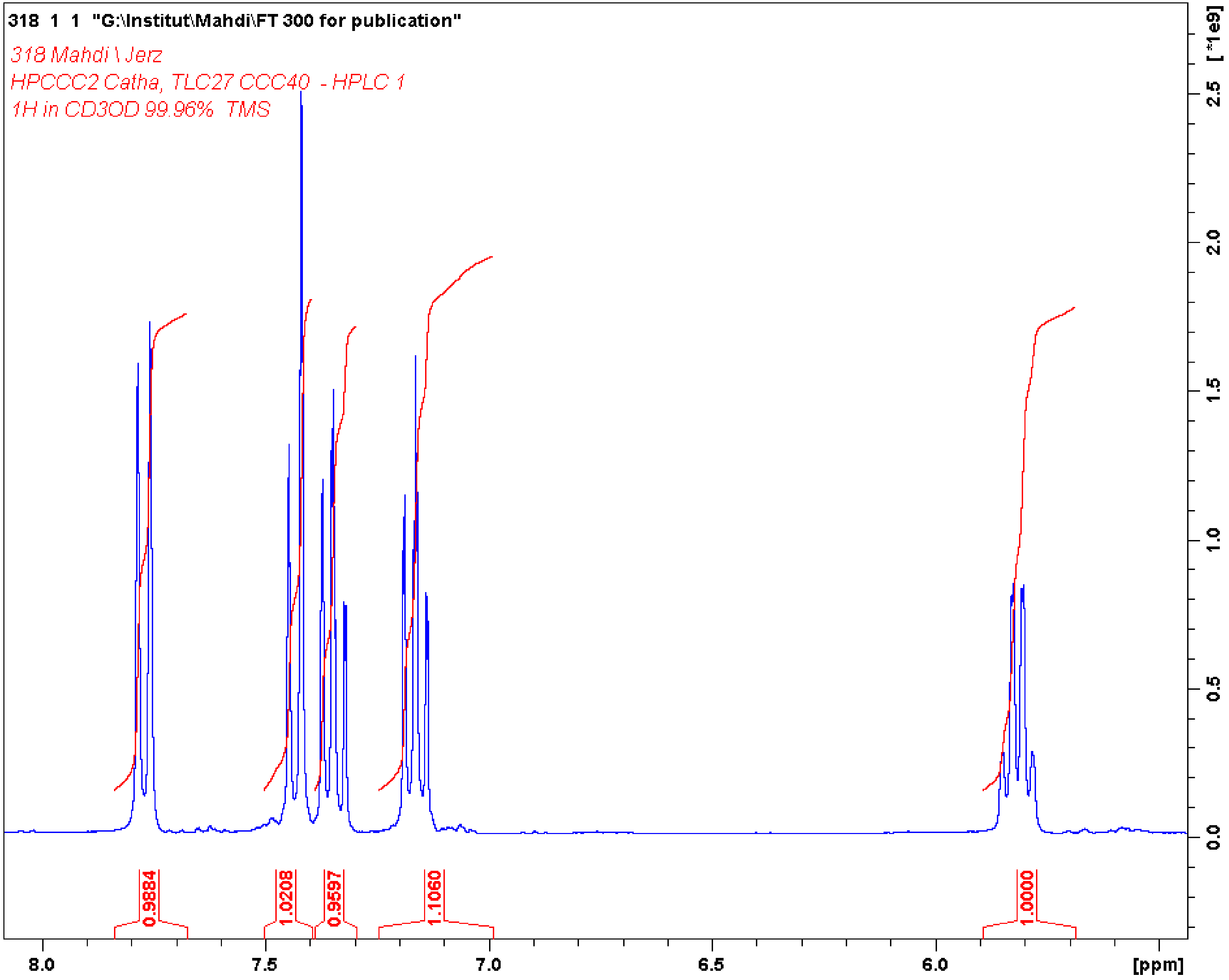

Figure NMR-S4

^{1}H NMR – Perivine (*329-a*) in CD_3OD (300 MHz)

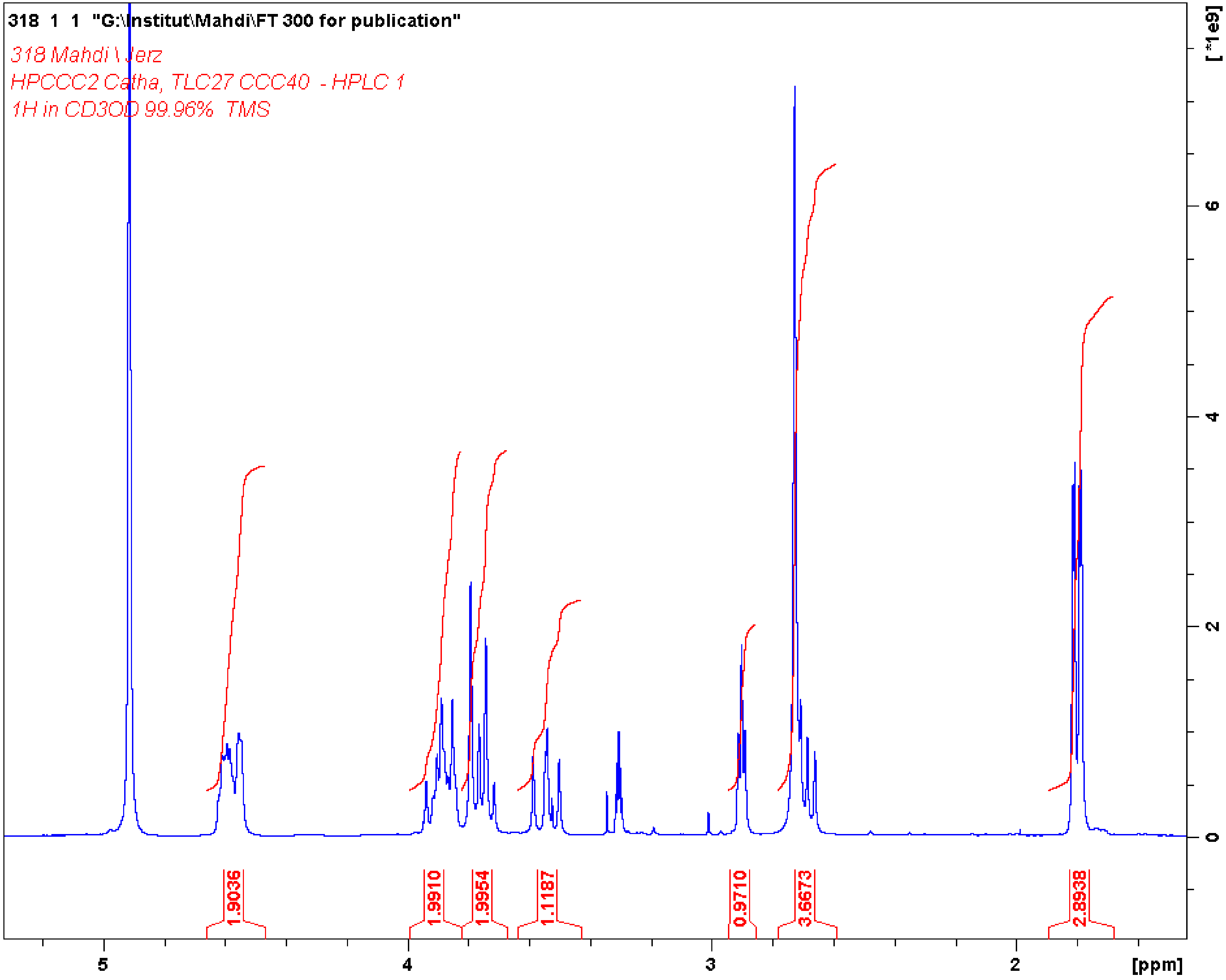

Figure NMR-S4

^{13}C NMR – Perivine (*329-a*)
in CD_3OD
(75 MHz)

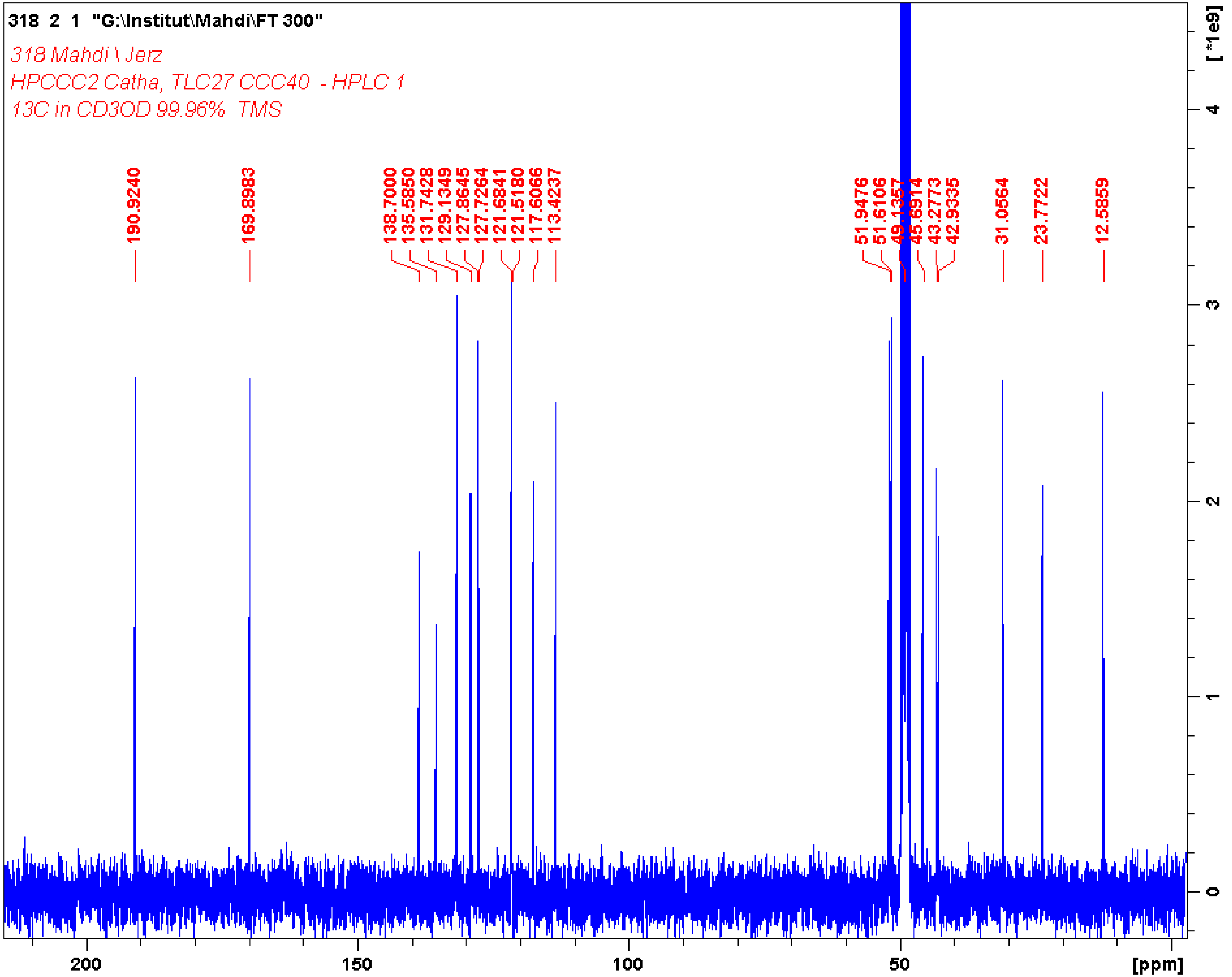

Figure NMR-S4

^{13}C NMR – Perivine (*329-a*)
in CD_3OD
(75 MHz)

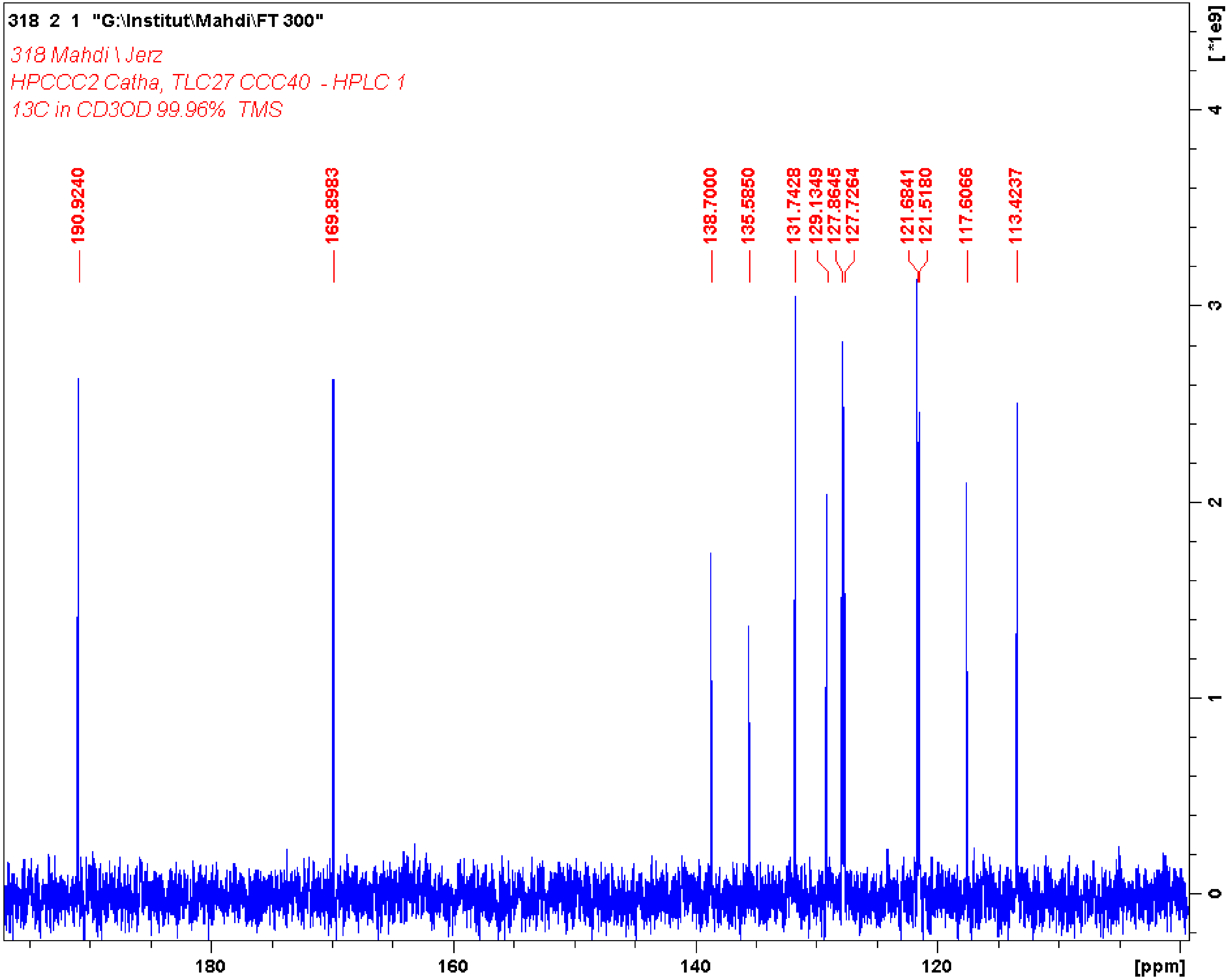

Figure NMR-S4

^{13}C NMR – Perivine (*329-a*)
in CD_3OD
(75 MHz)

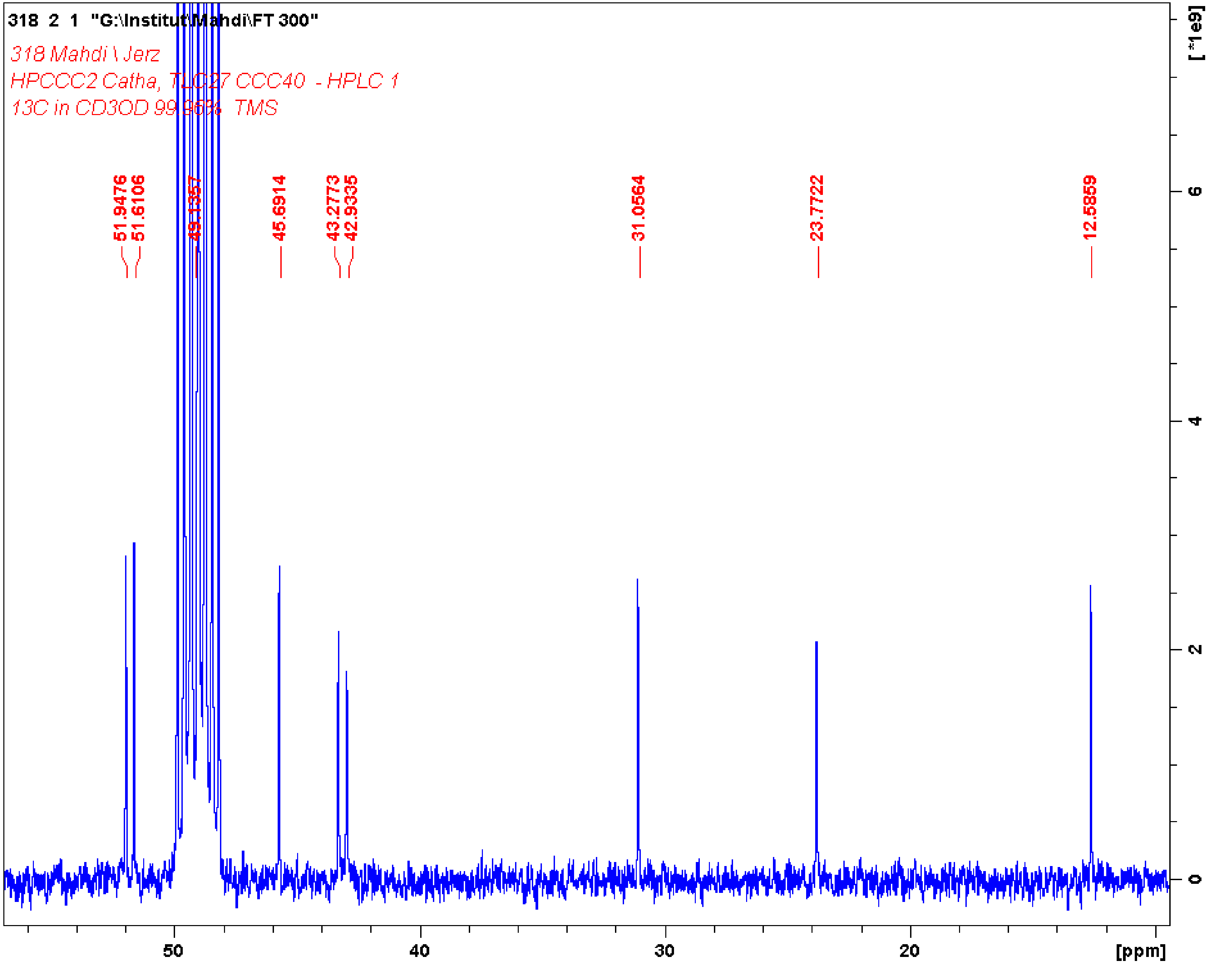

Figure NMR-S4

DEPT-135 NMR – Perivine (*329-a*) in CD_3OD (75 MHz)

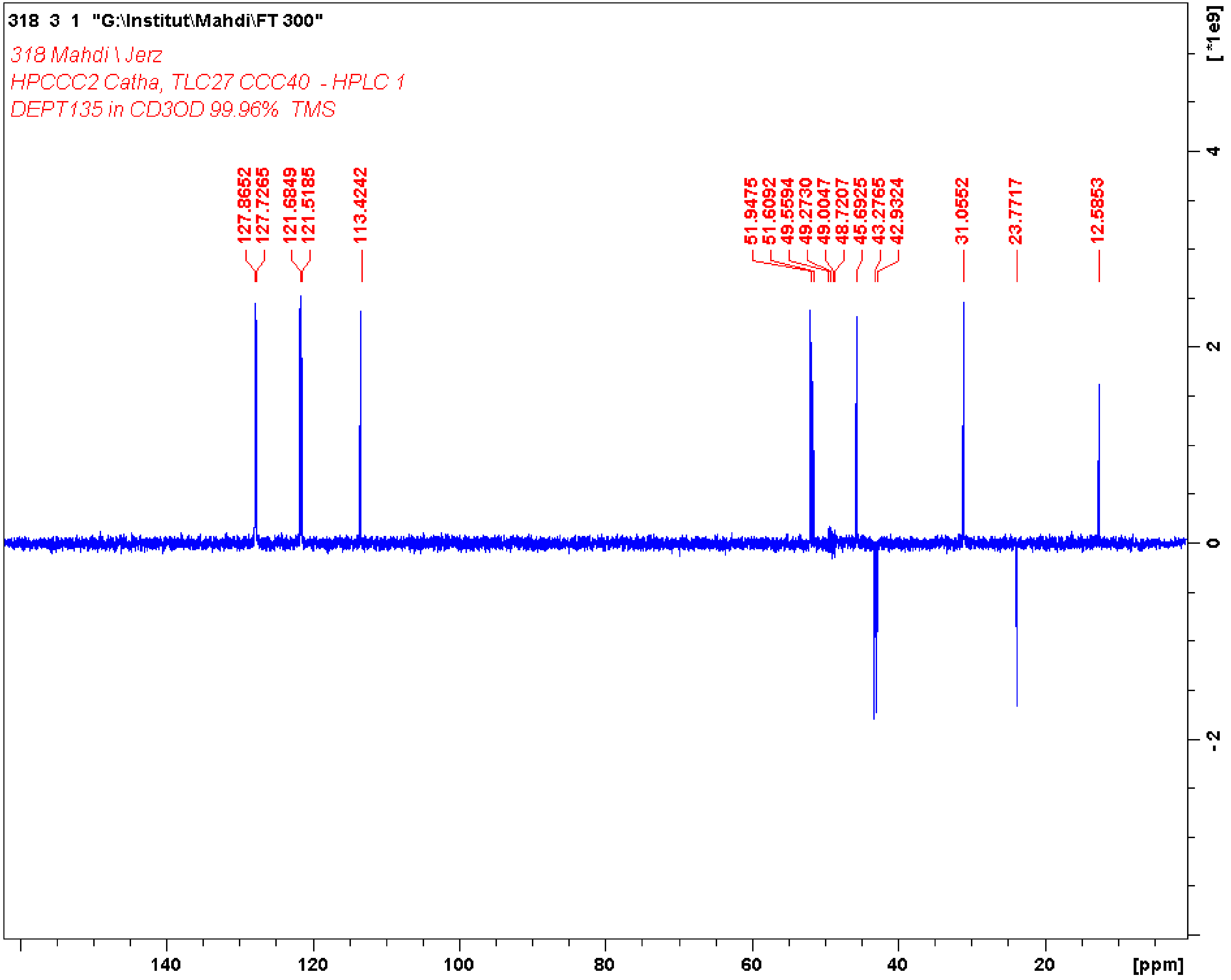

Figure NMR-S4

DEPT-135 NMR – Perivine (*329-a*)
in CD_3OD
(75 MHz)

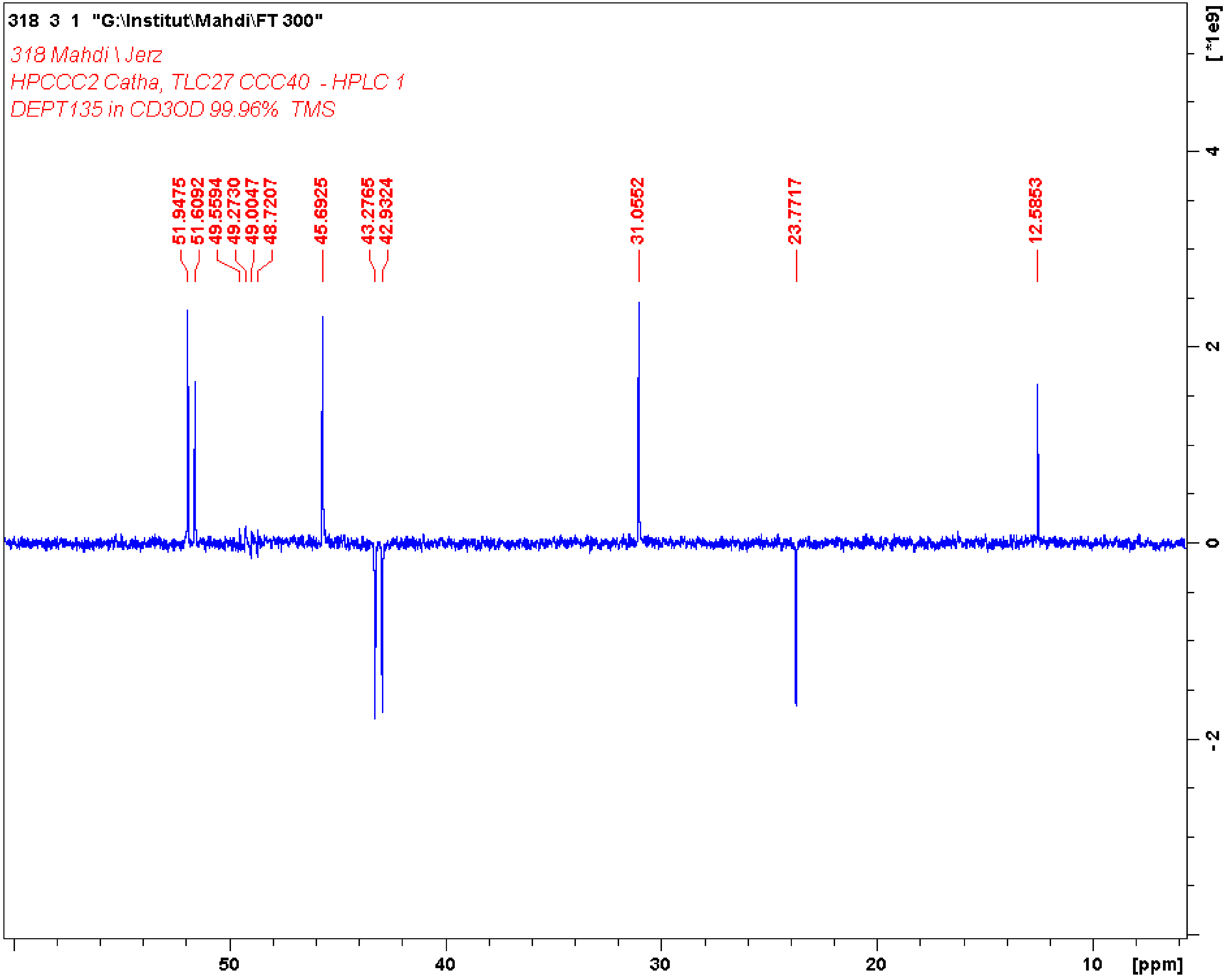

Figure NMR-S4

$^1H/^1H$-COSY
Perivine (*329-a*)
in CD_3OD

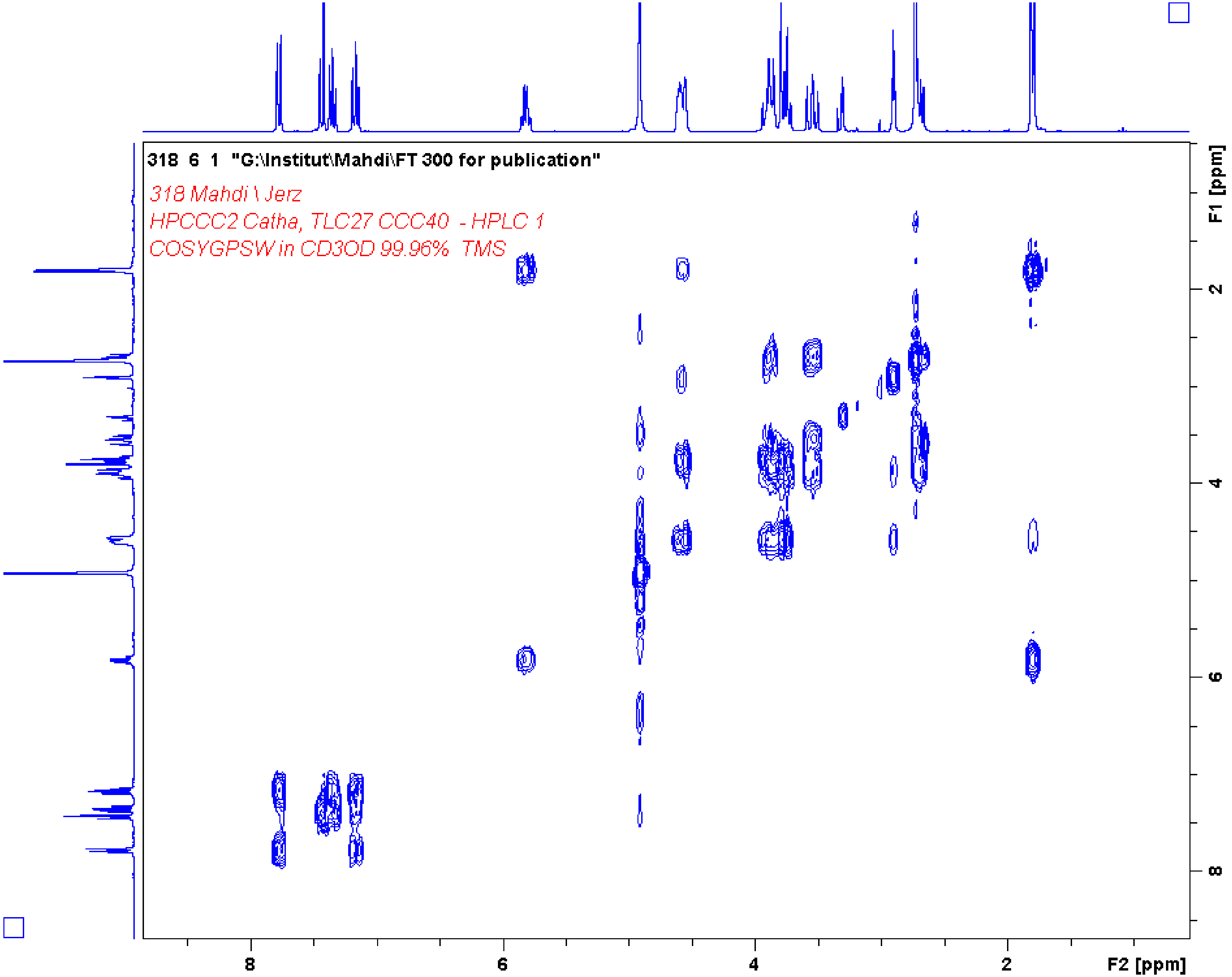

Figure NMR-S4

HSQC phase edited ^{1}J-*HC*

Perivine (*329-a*)
in CD_3OD

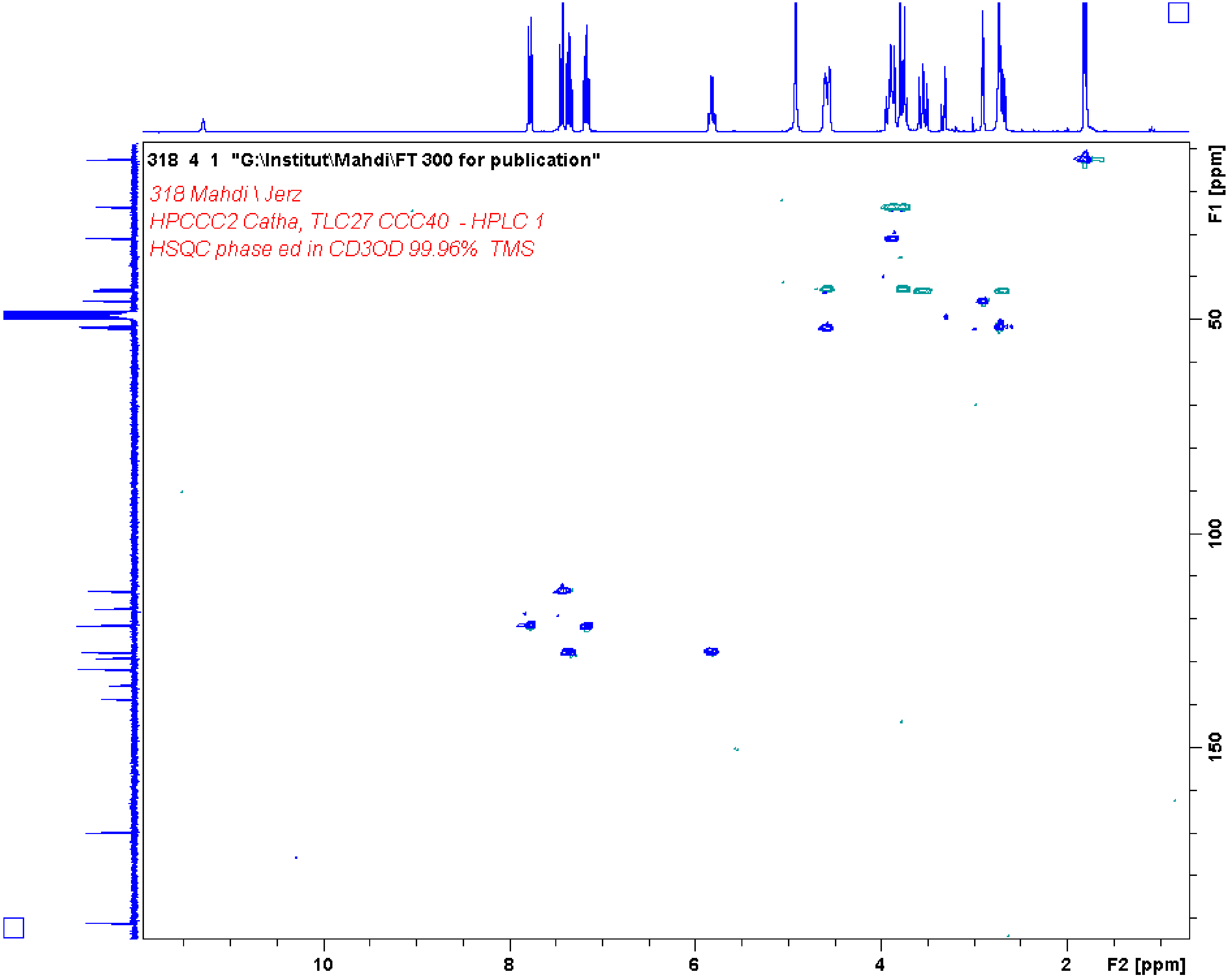

Figure NMR-S4

HSQC phase edited *¹J-HC*

Perivine (*329-a*)
in CD_3OD

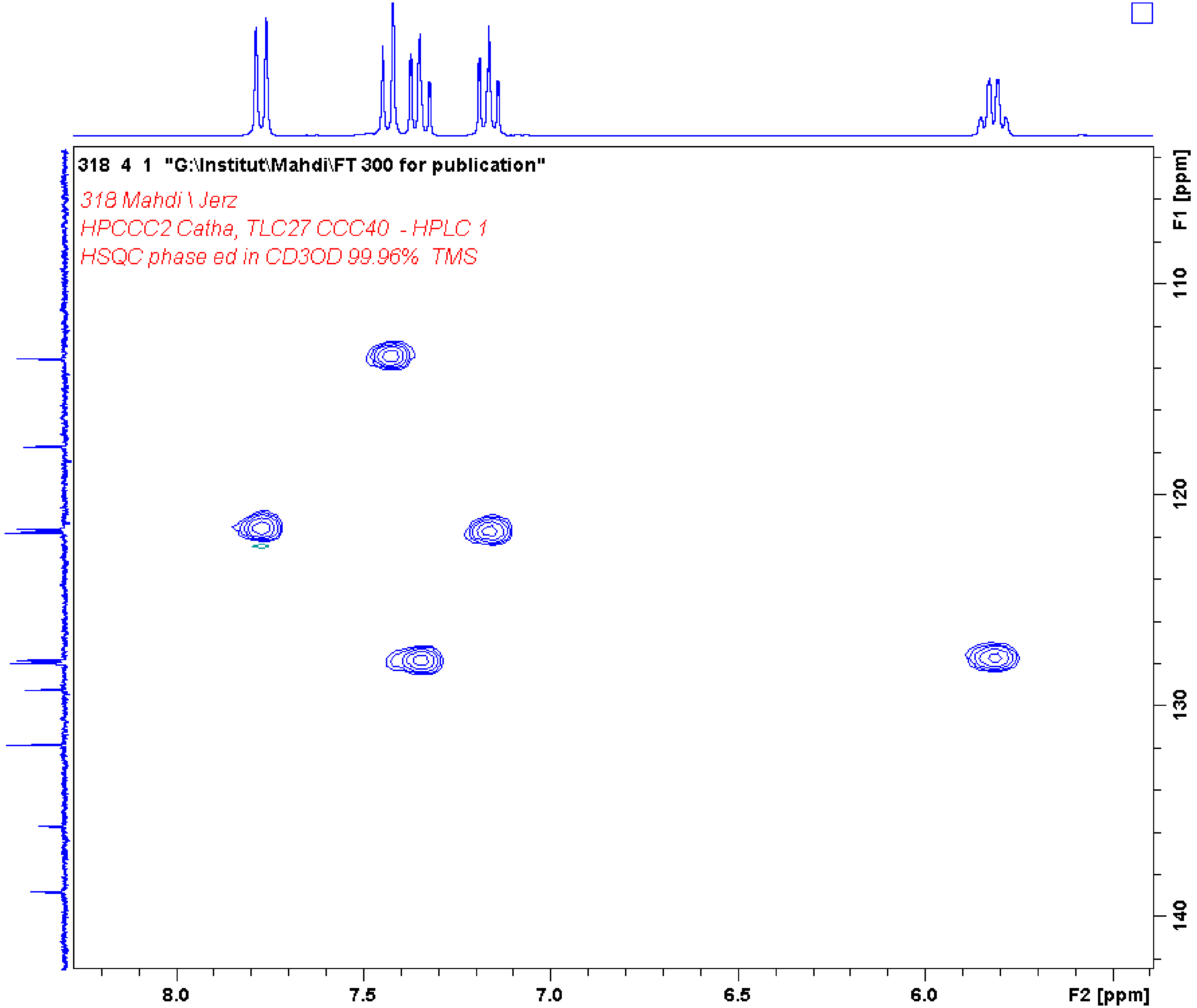

Figure NMR-S4

HSQC phase edited ^{1}J-HC

Perivine (*329-a*)
in CD_3OD

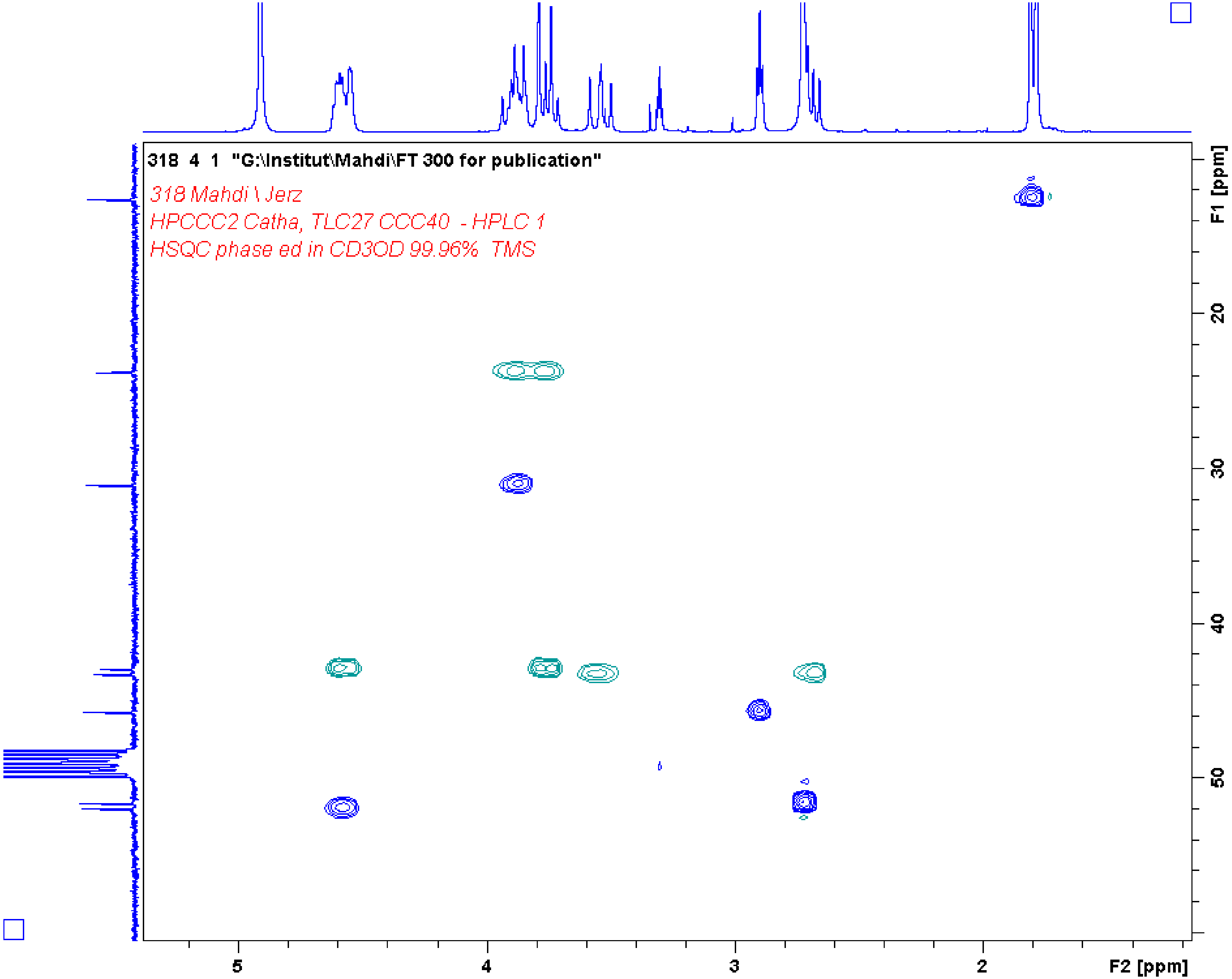

Figure NMR-S4

HMBC, long-range ^{2,3}*J-HC*

Perivine (*329-a*)
in CD_3OD

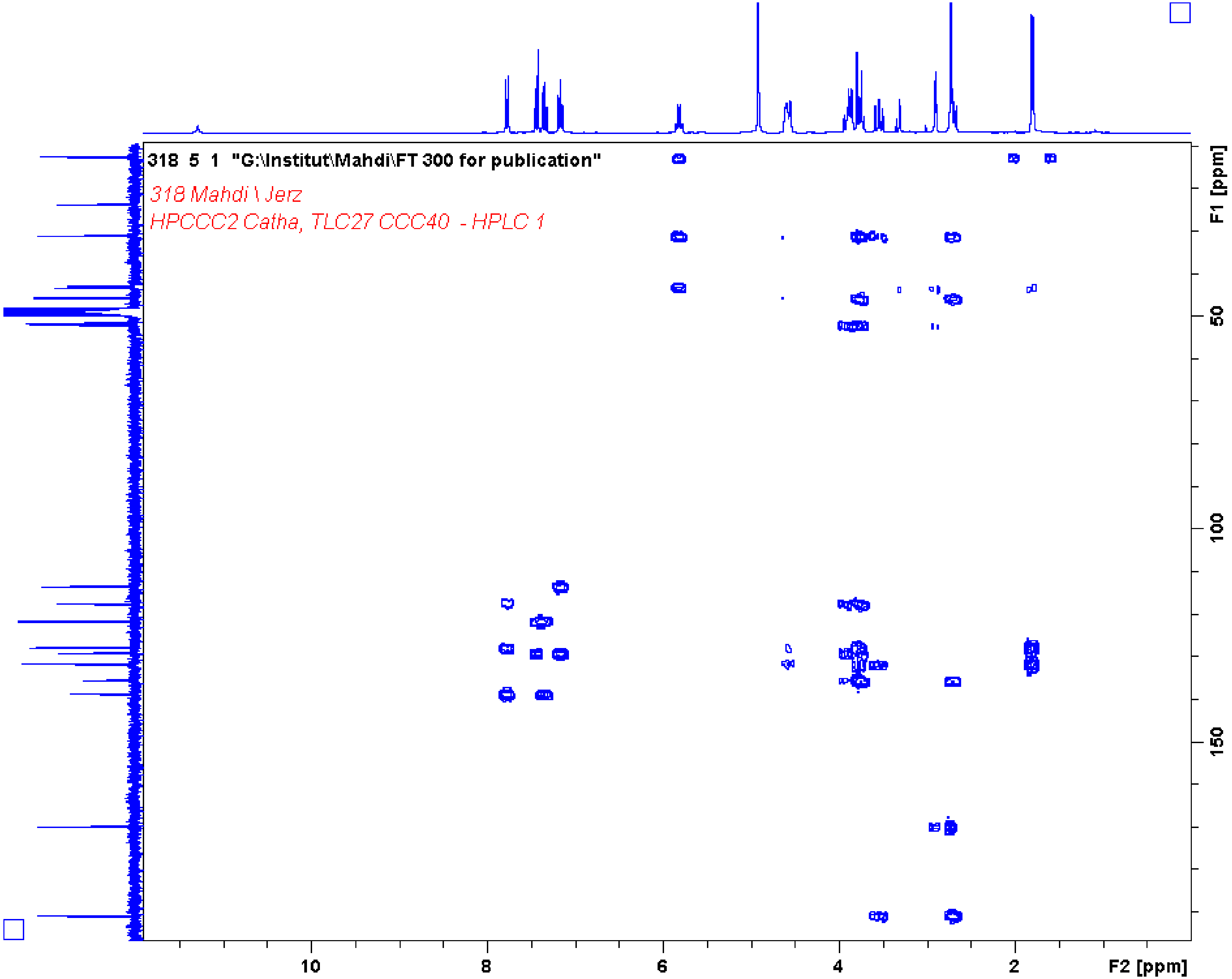

Figure NMR-S4

HMBC, long-range $^{2,3}J$-HC

Perivine (*329-a*) in CD_3OD

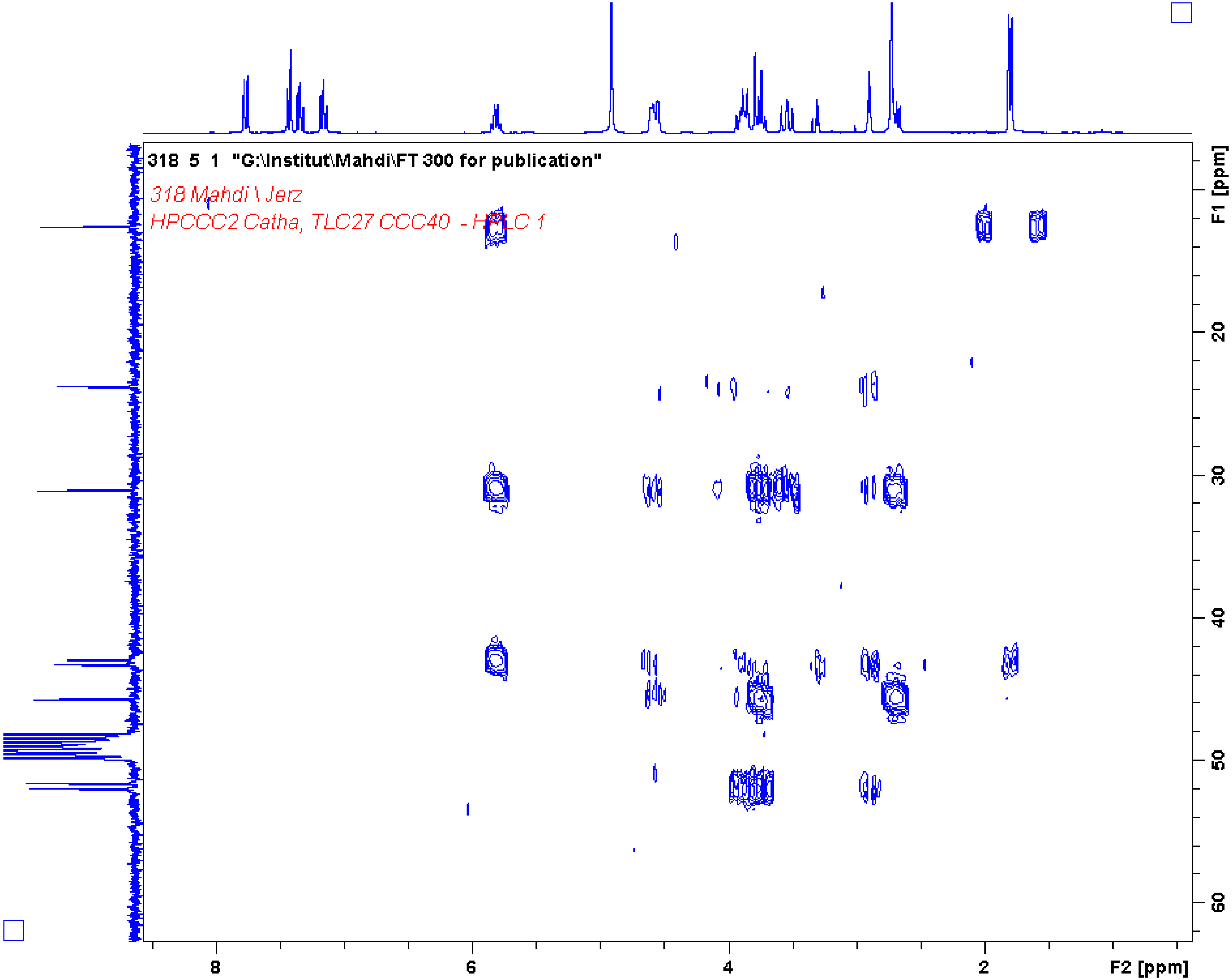

Figure NMR-S4

HMBC, long-range $^{2,3}J$-*HC*

Perivine (*329-a*)
in CD_3OD

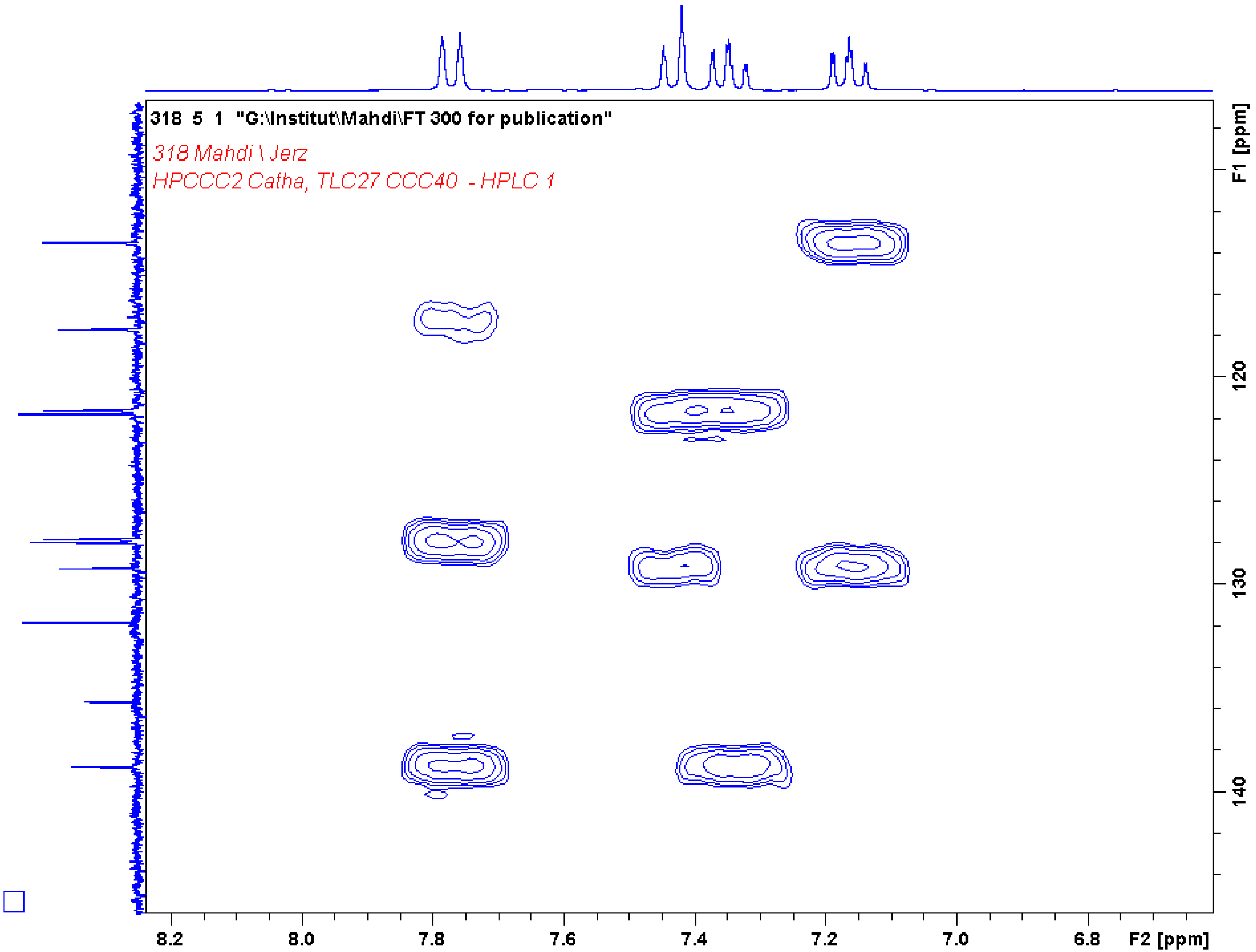

Figure NMR-S4

HMBC, long-range ^{2,3}*J-HC*

Perivine (*329-a*)
in CD_3OD

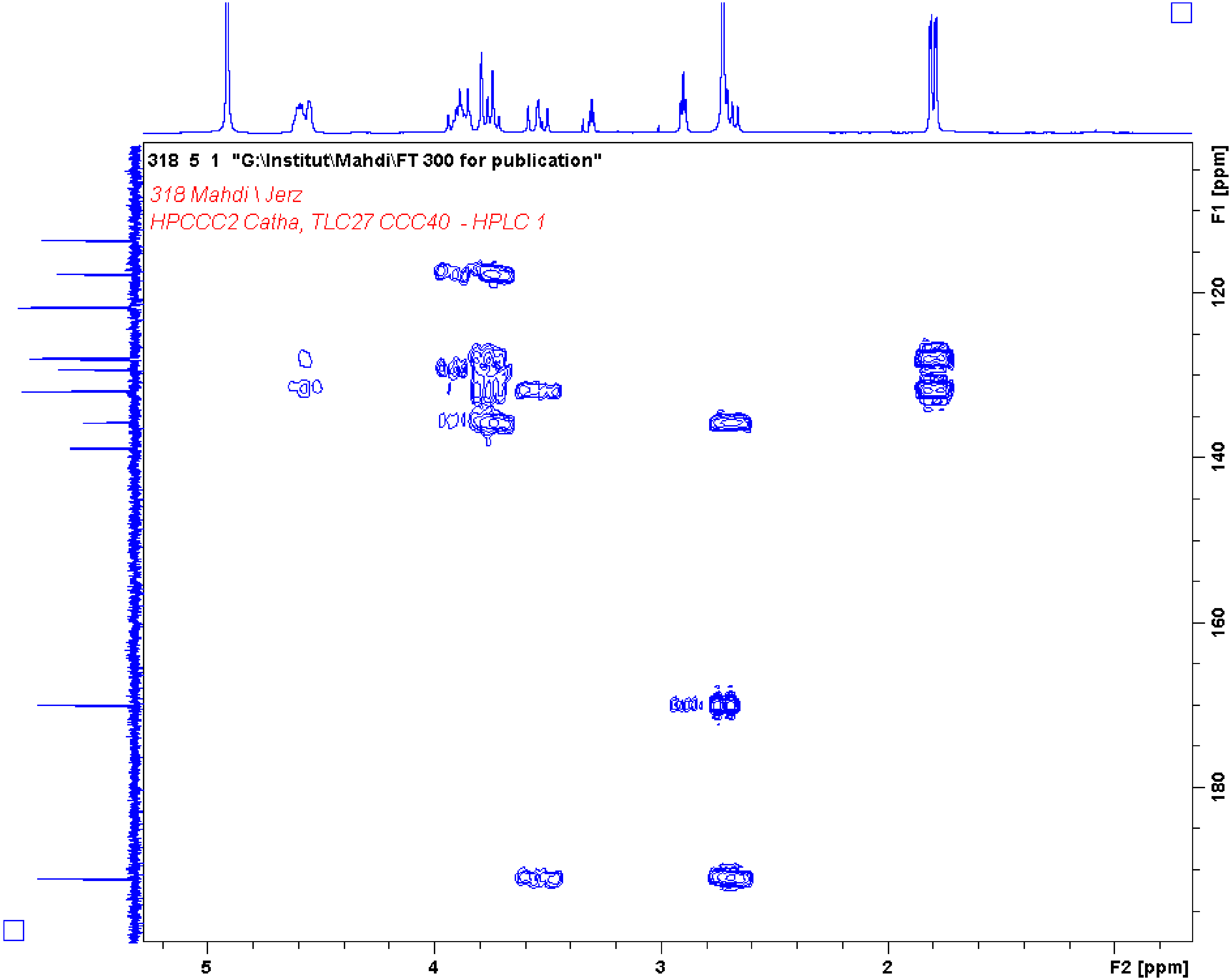

Figure NMR-S4

^1H/^1H-NOESY
Perivine (*329-a*)
in CD_3OD

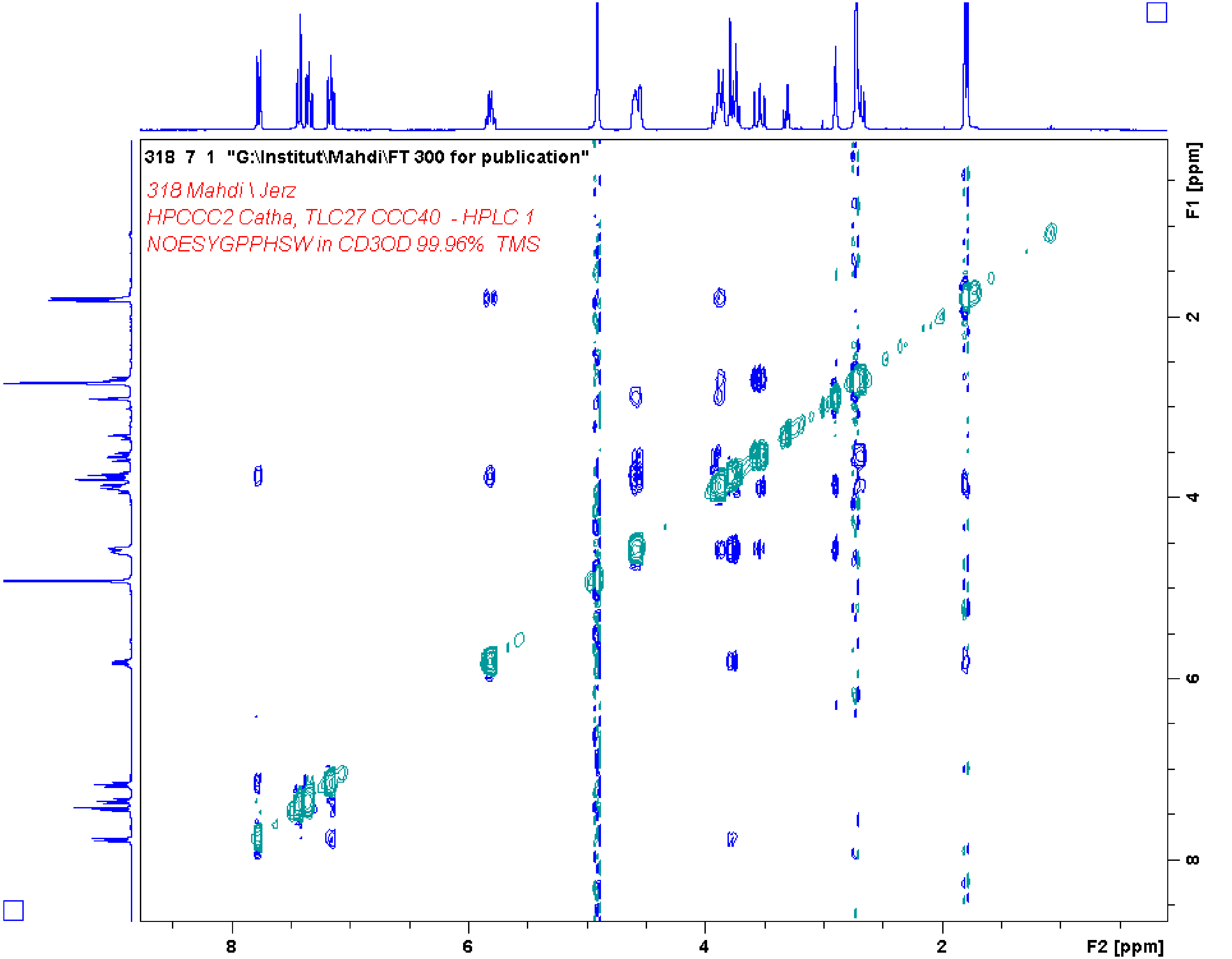

Figure NMR-S5

^{1}H NMR – Vindorosine (*427*) in CD_3OD (300 MHz)

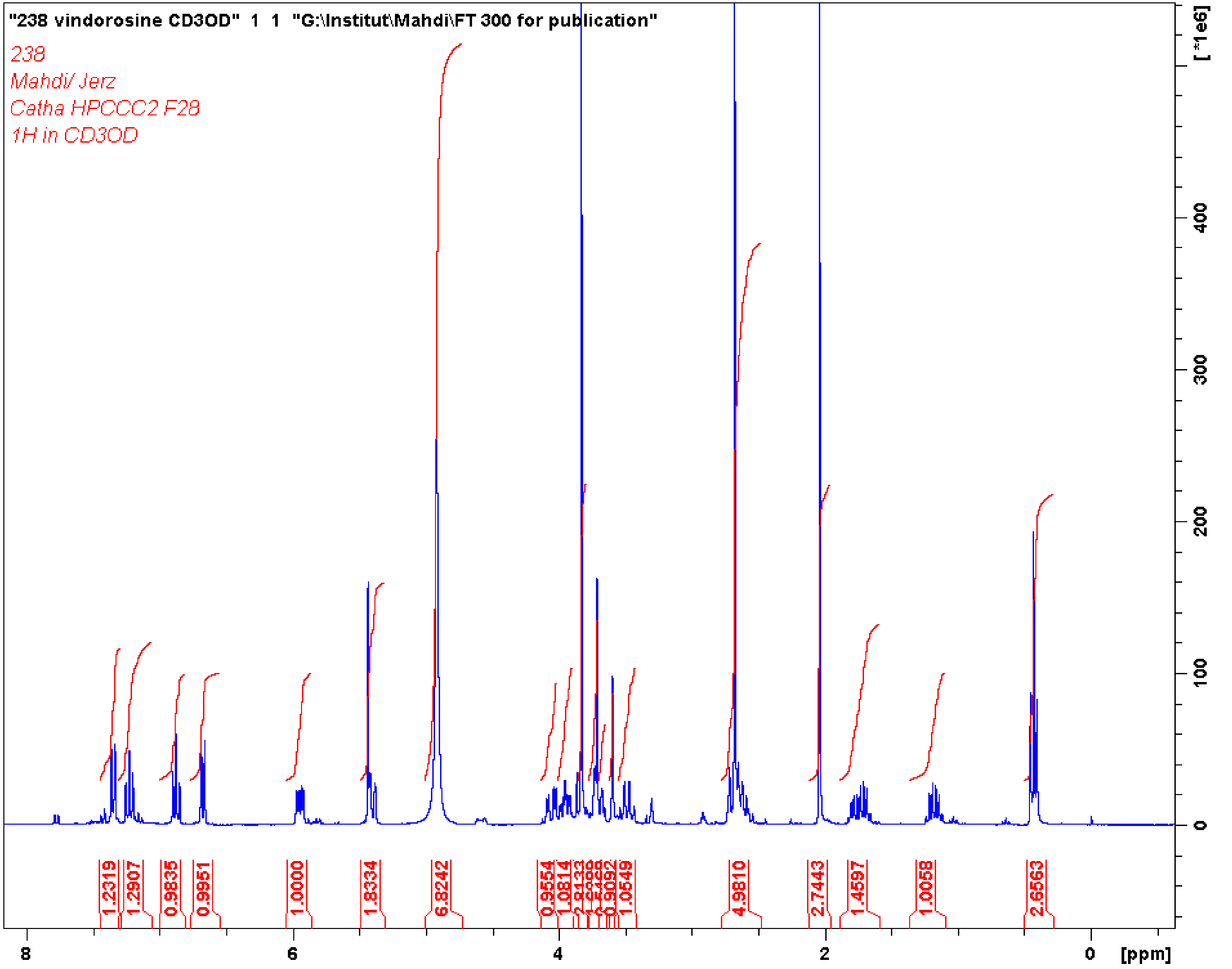

Figure NMR-S5

^{1}H NMR – Vindorosine (*427*) in CD_3OD (300 MHz)

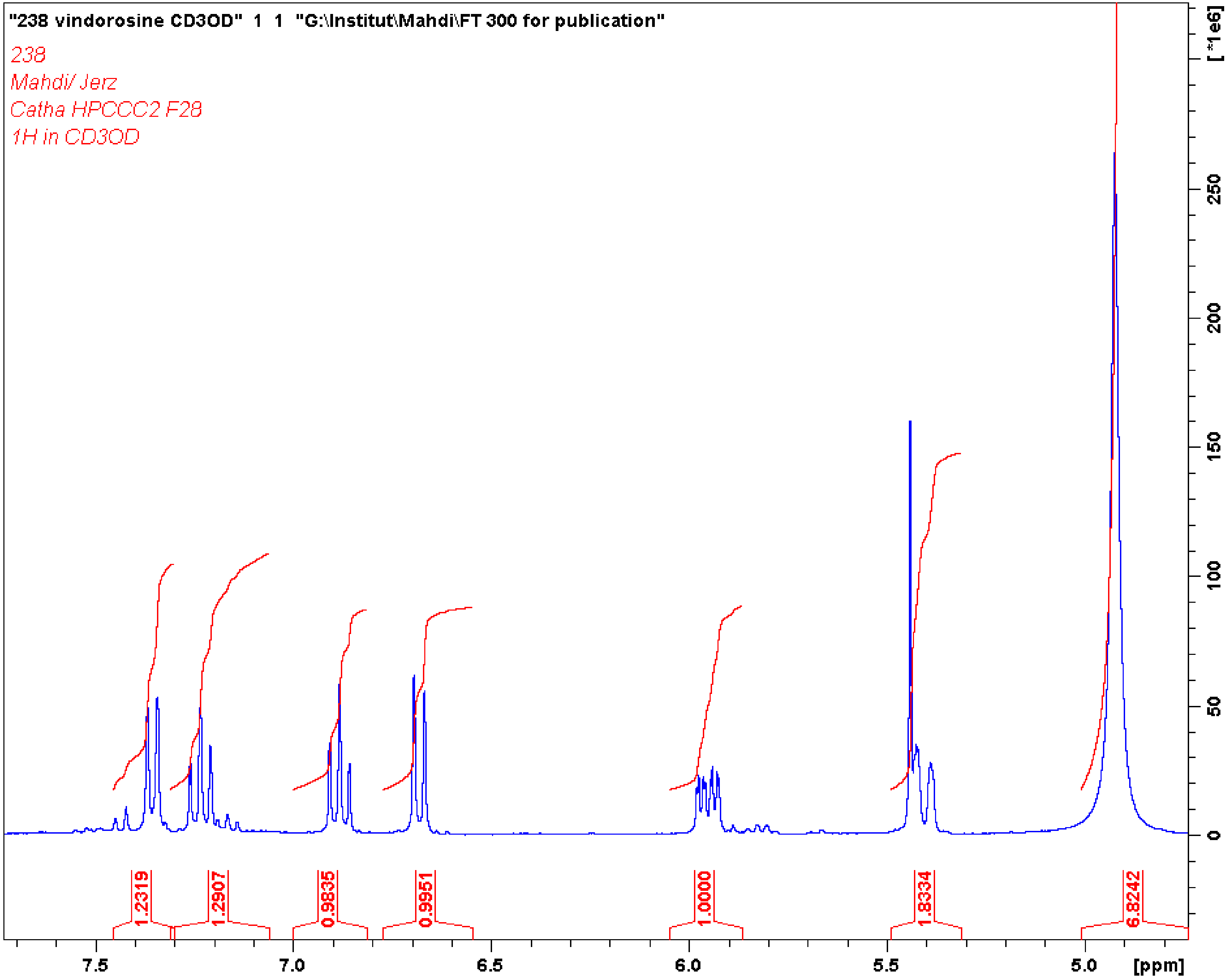

Figure NMR-S5

^{1}H NMR – Vindorosine (*427*) in CD_3OD (300 MHz)

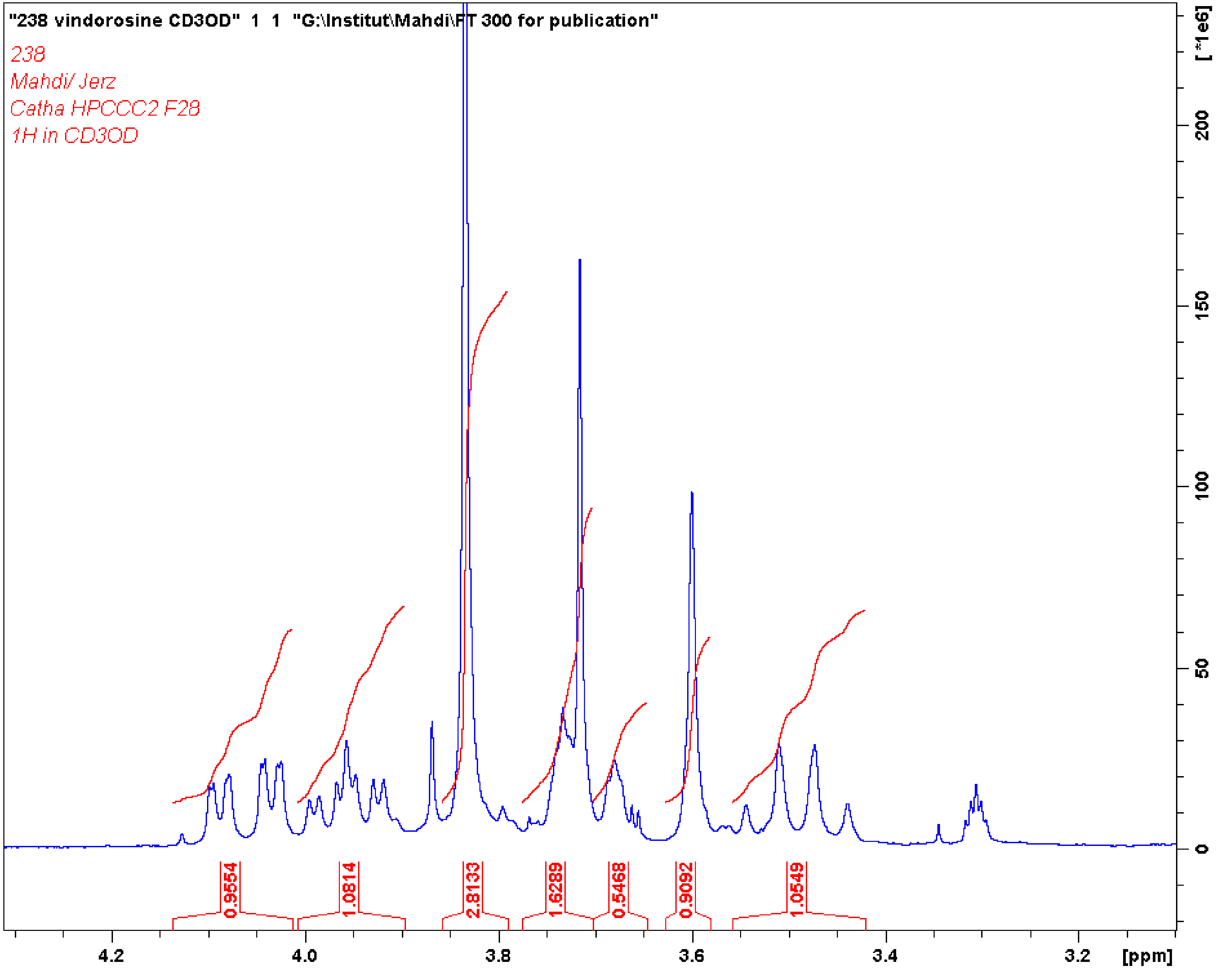

Figure NMR-S5

^{1}H NMR – Vindorosine (*427*) in CD_3OD (300 MHz)

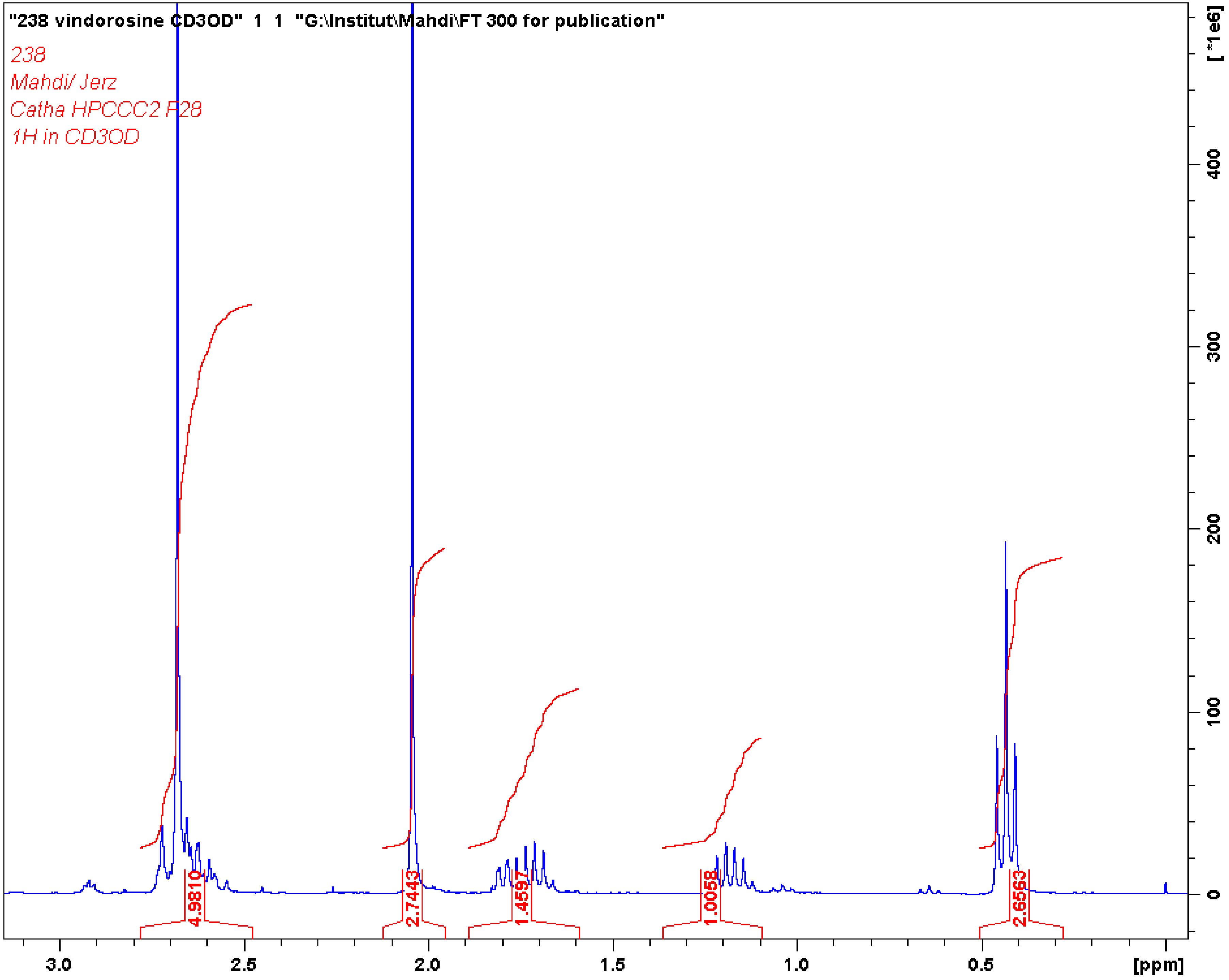

Figure NMR-S5

^{13}C NMR – Vindorosine (*427*) in CD_3OD (75 MHz)

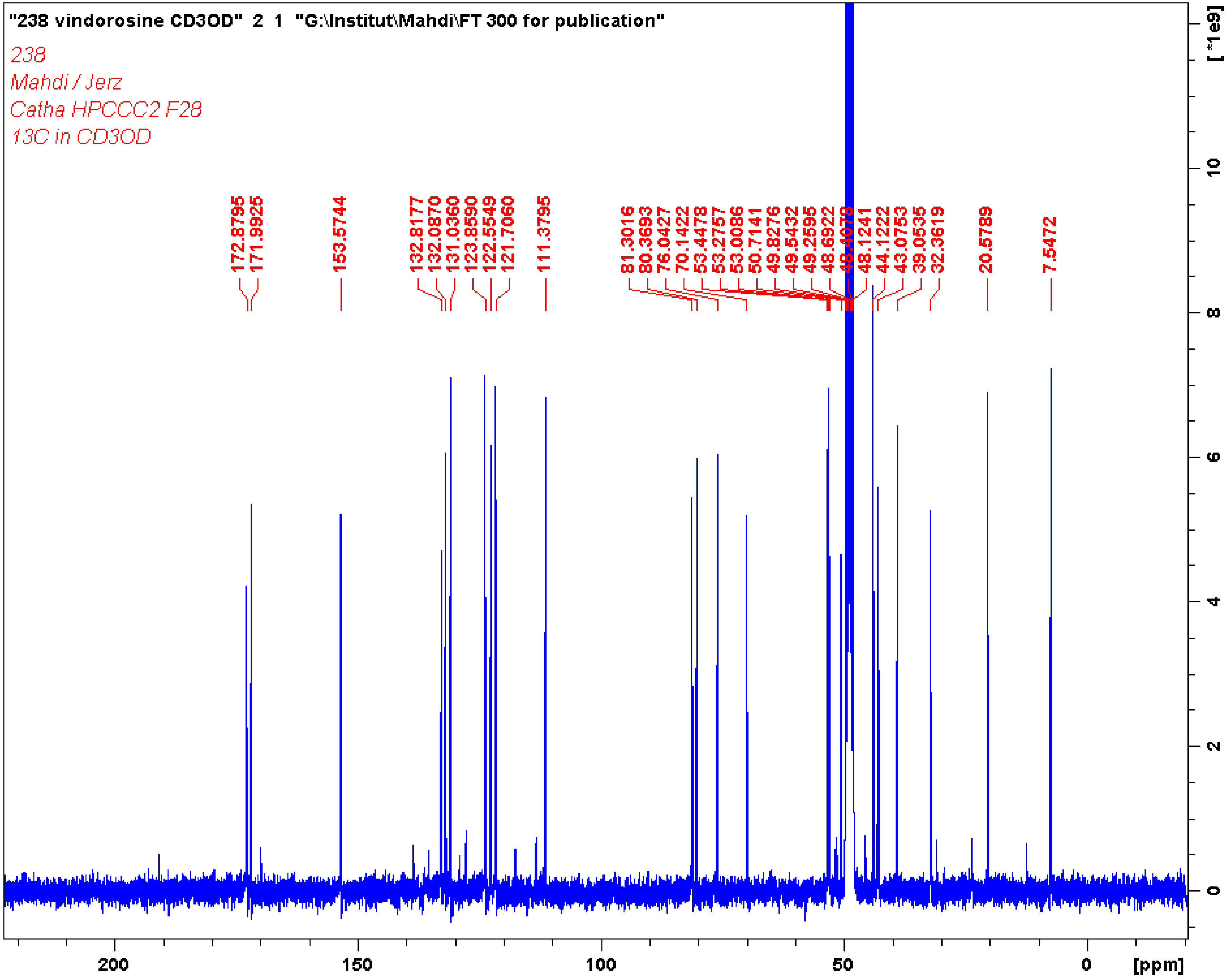

Figure NMR-S5

^{13}C NMR – Vindorosine (*427*) in CD_3OD (75 MHz)

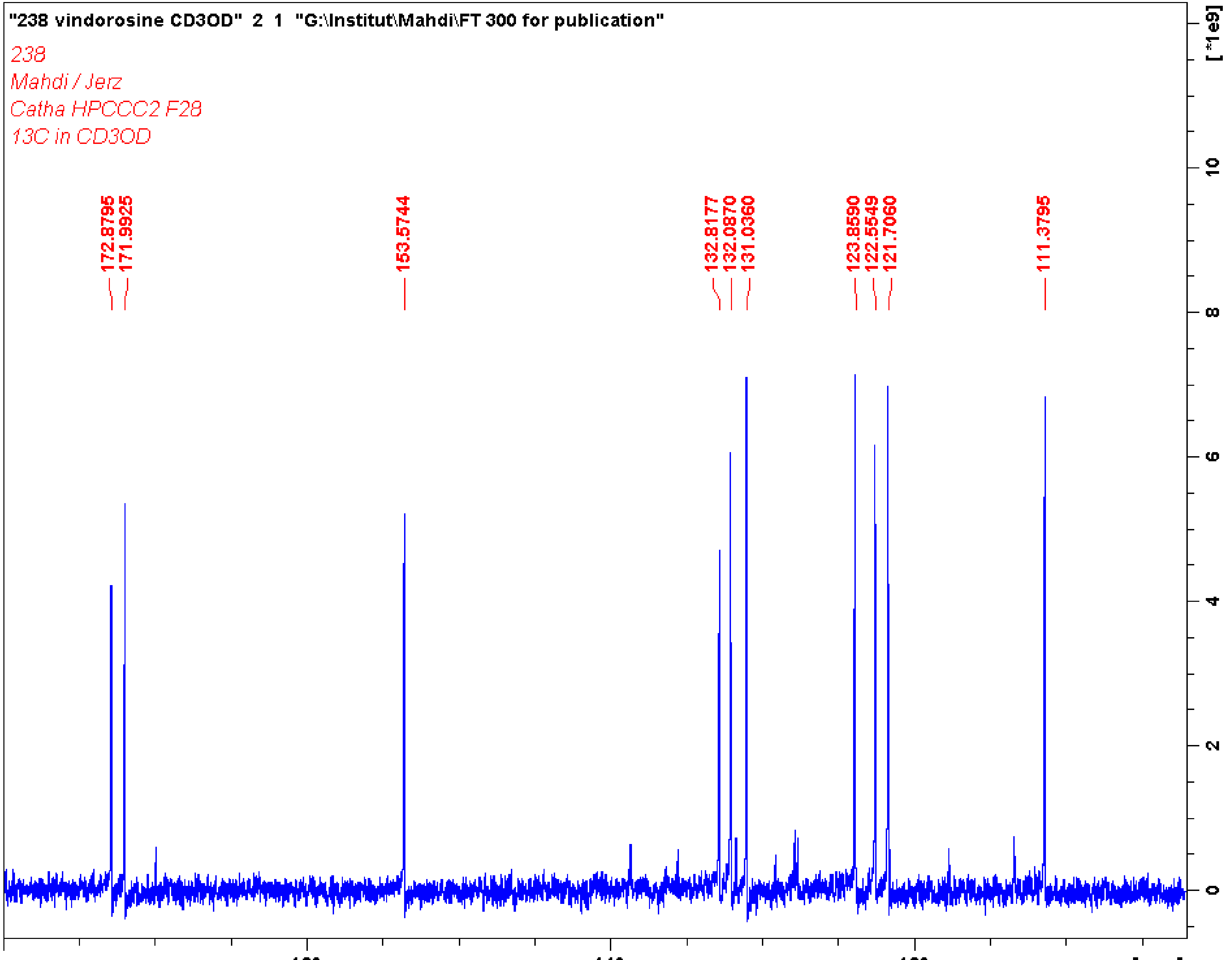

Figure NMR-S5

^{13}C NMR – Vindorosine (*427*) in CD_3OD (75 MHz)

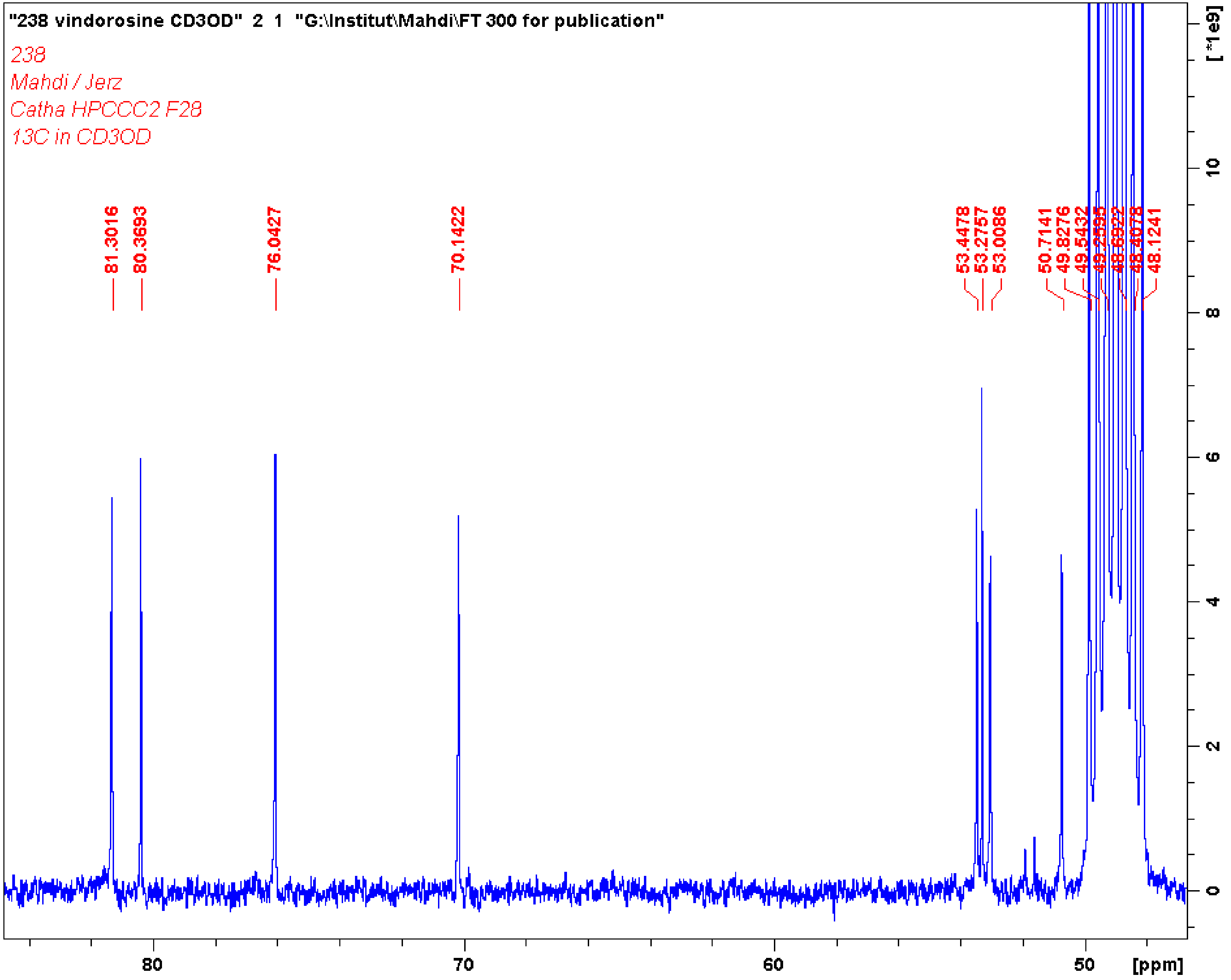

Figure NMR-S5

^{13}C NMR – Vindorosine (*427*)
in CD_3OD
(75 MHz)

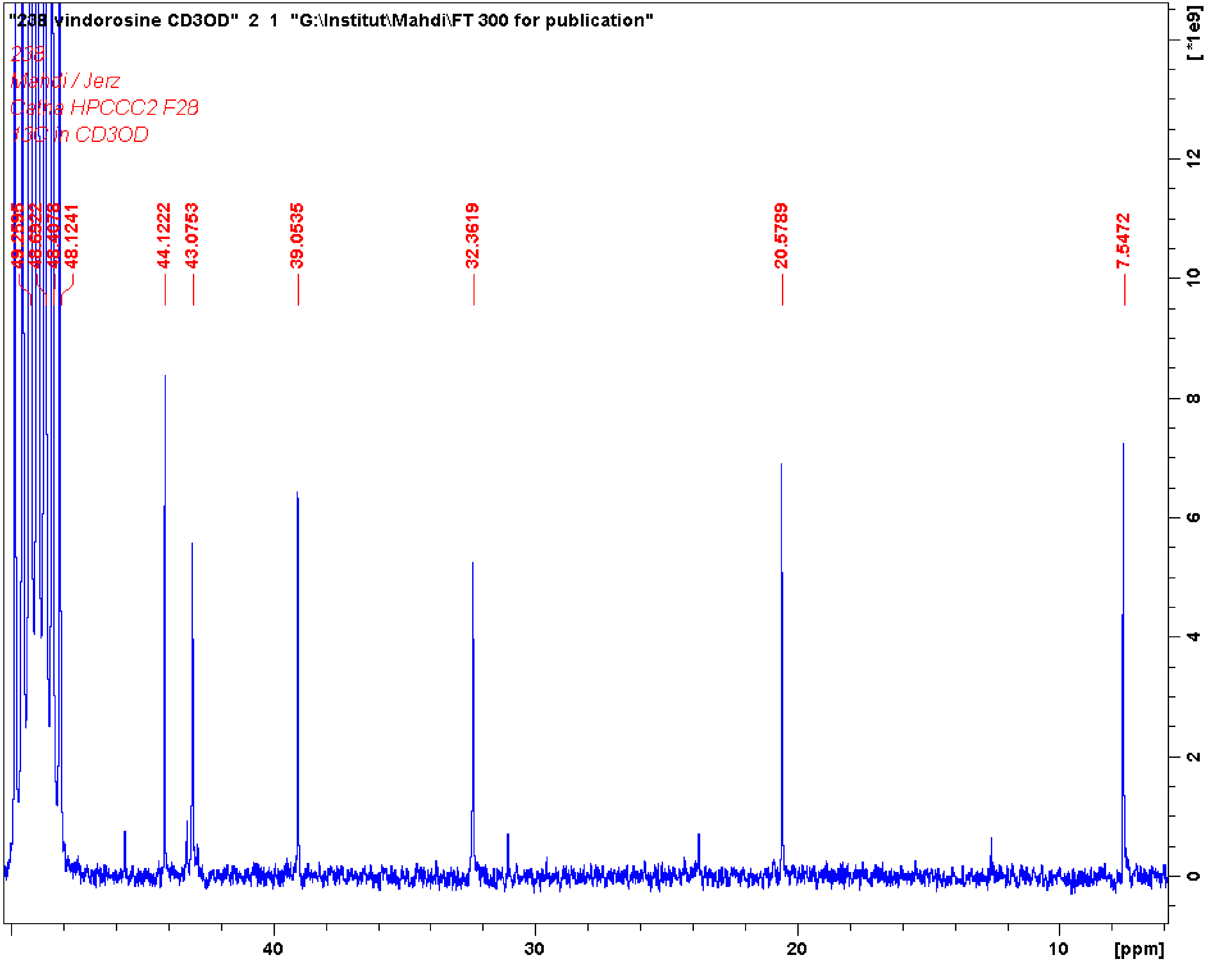

Figure NMR-S5

**DEPT 135 -NMR – Vindorosine (*427*
in CD_3OD
(75 MHz)**

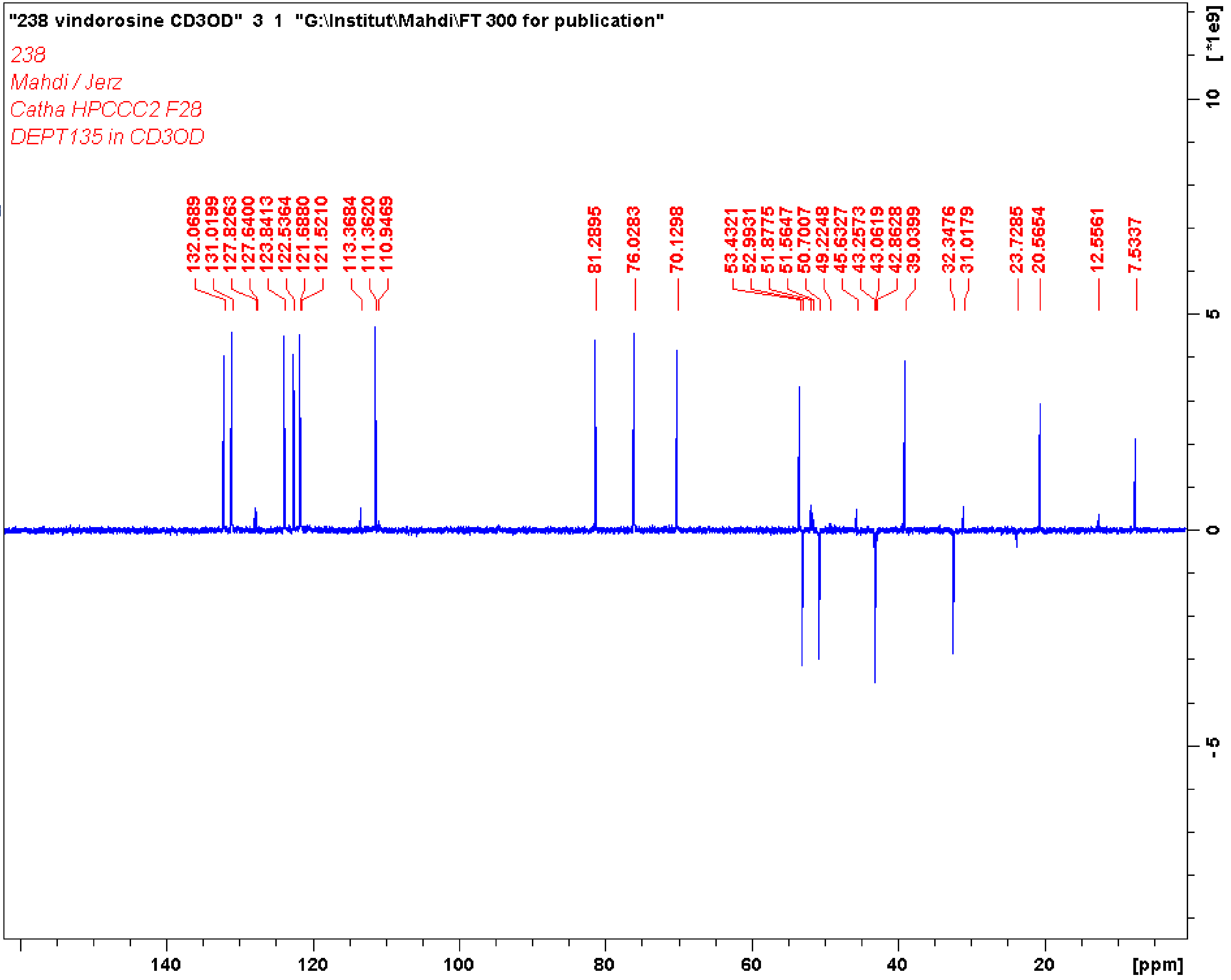

Figure NMR-S5

DEPT 135 -NMR
Vindorosine (*427*)
in CD_3OD
(75 MHz)

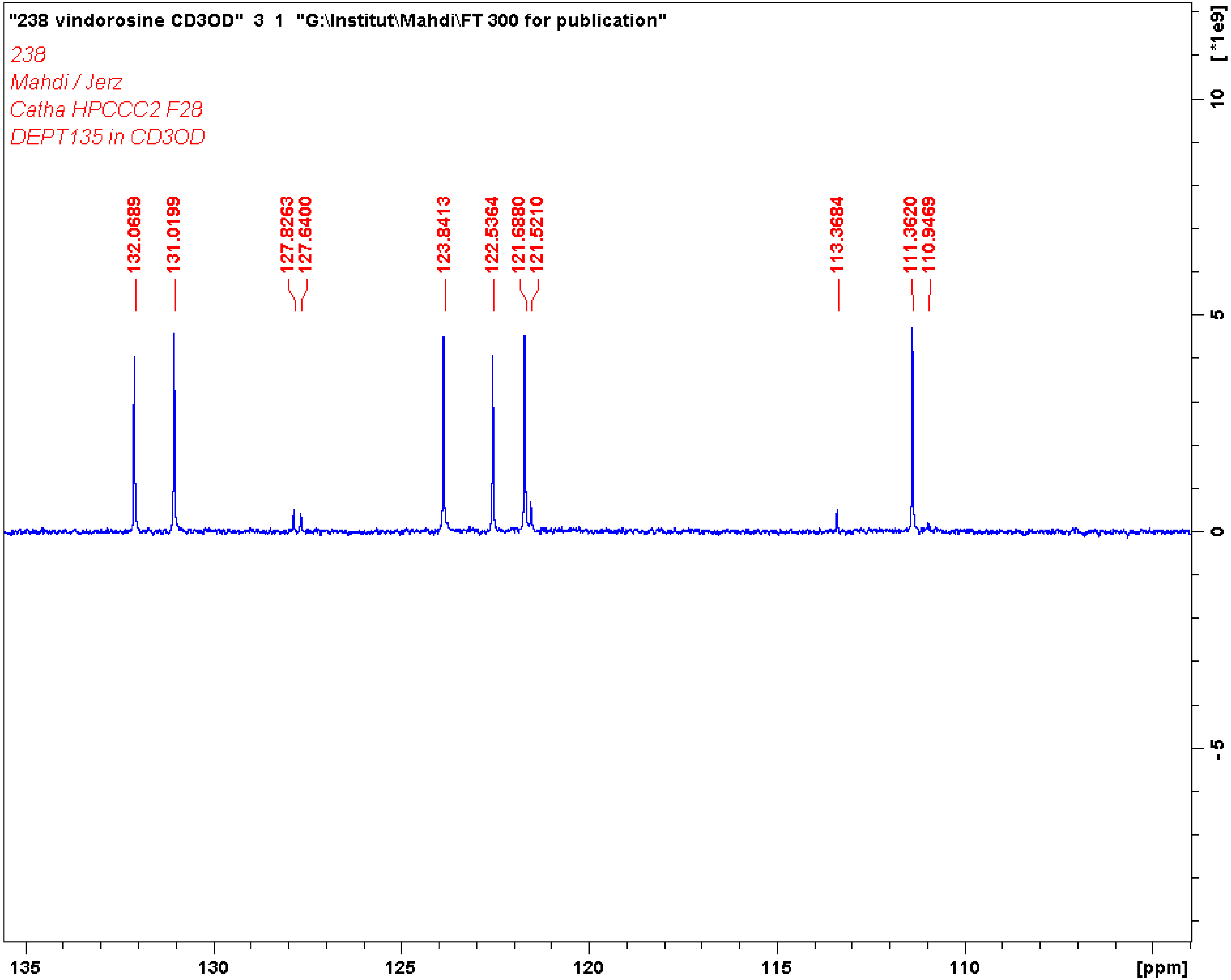

Figure NMR-S5

DEPT 135 -NMR
Vindorosine (*427*)
in CD_3OD
(75 MHz)

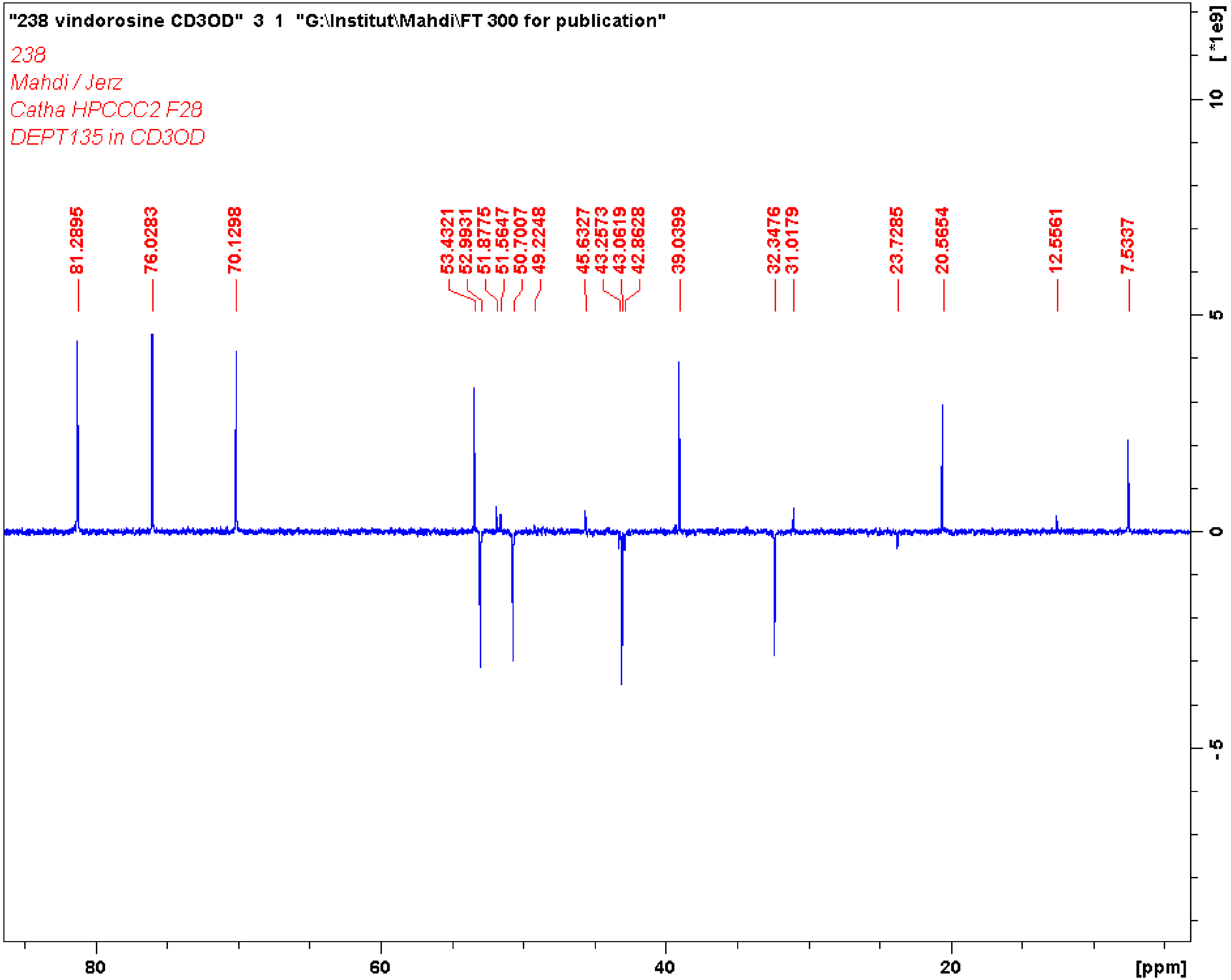

Figure NMR-S5

HSQC phase edited ^{1}J-HC
Vindorosine (*427*) in CD_3OD

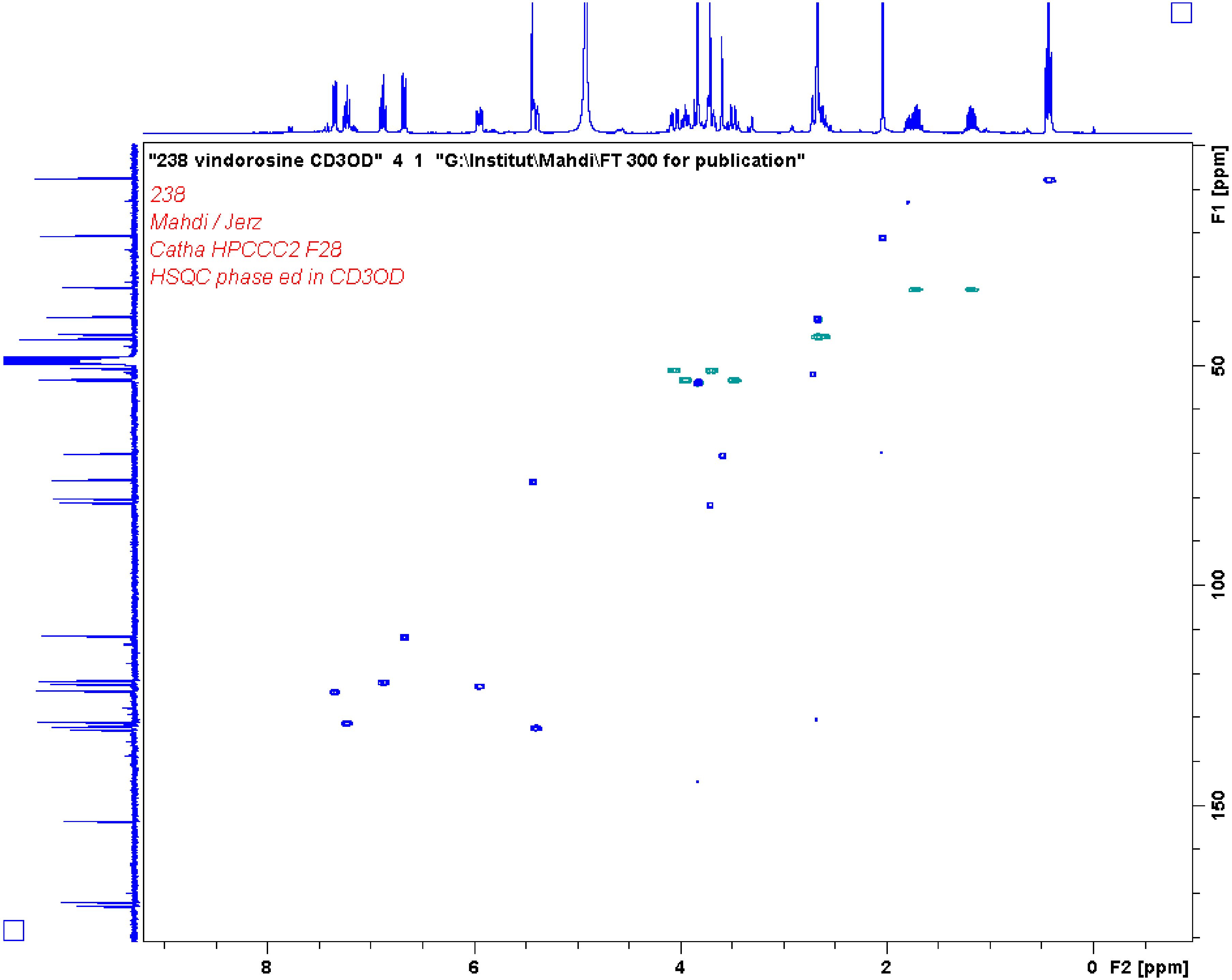

Figure NMR-S5

HSQC phase edited ^{1}J-HC
Vindorosine (*427*) in CD_3OD

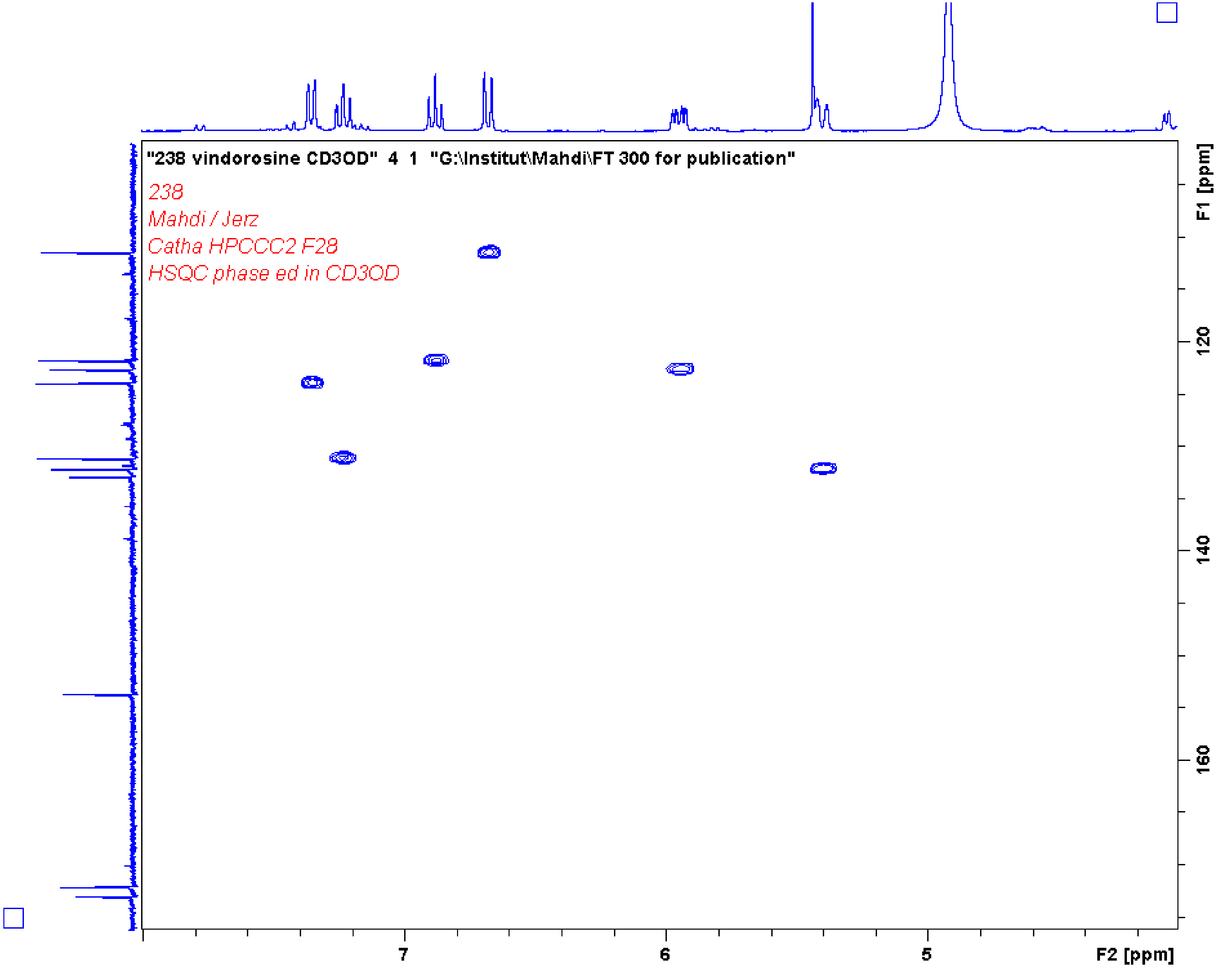

Figure NMR-S5

HSQC phase edited ^{1}J-HC Vindorosine (*427*) in CD_3OD

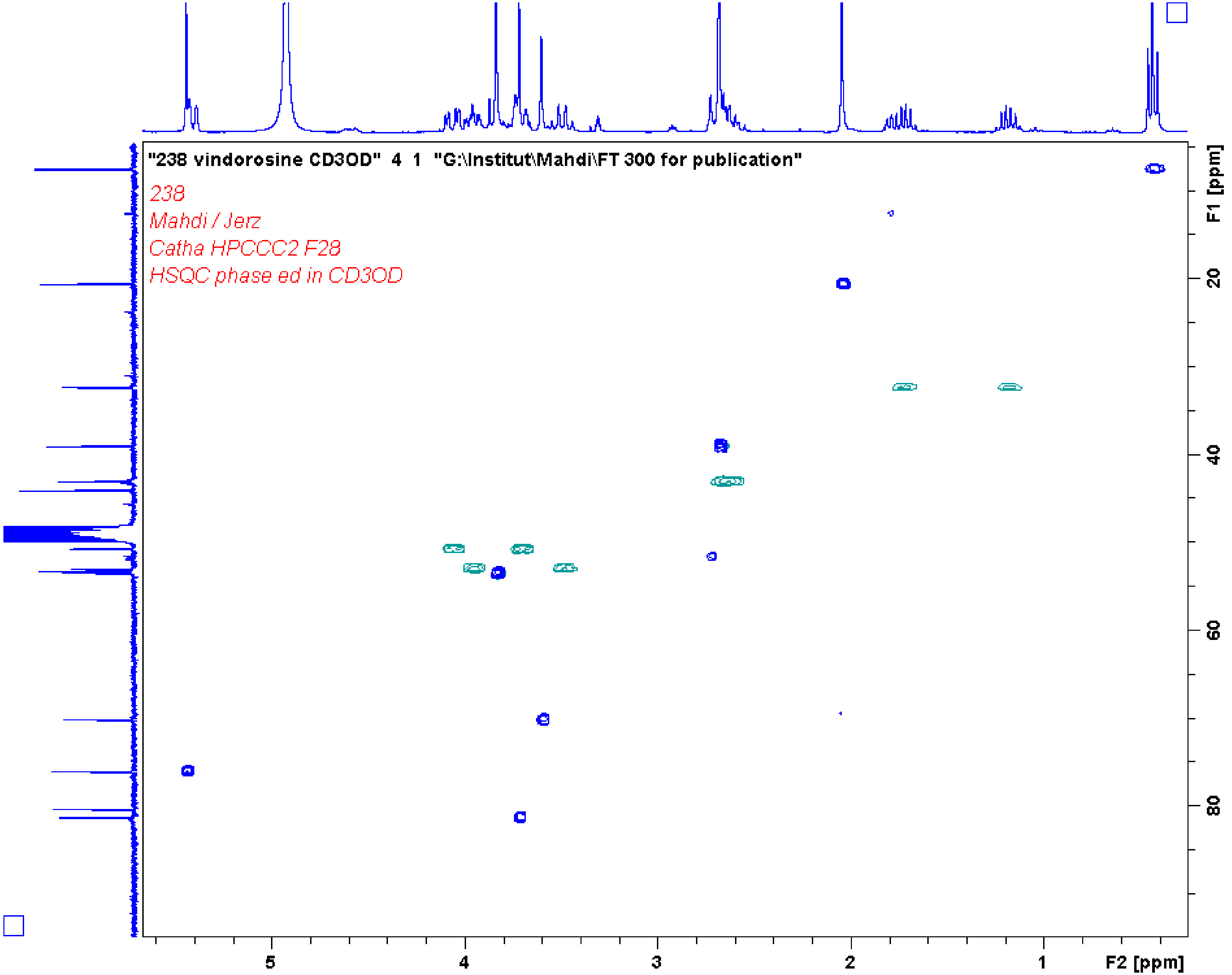

Figure NMR-S5

HMBC, long-range ^{2,3}*J-HC*

Vindorosine (*427*) in CD_3OD

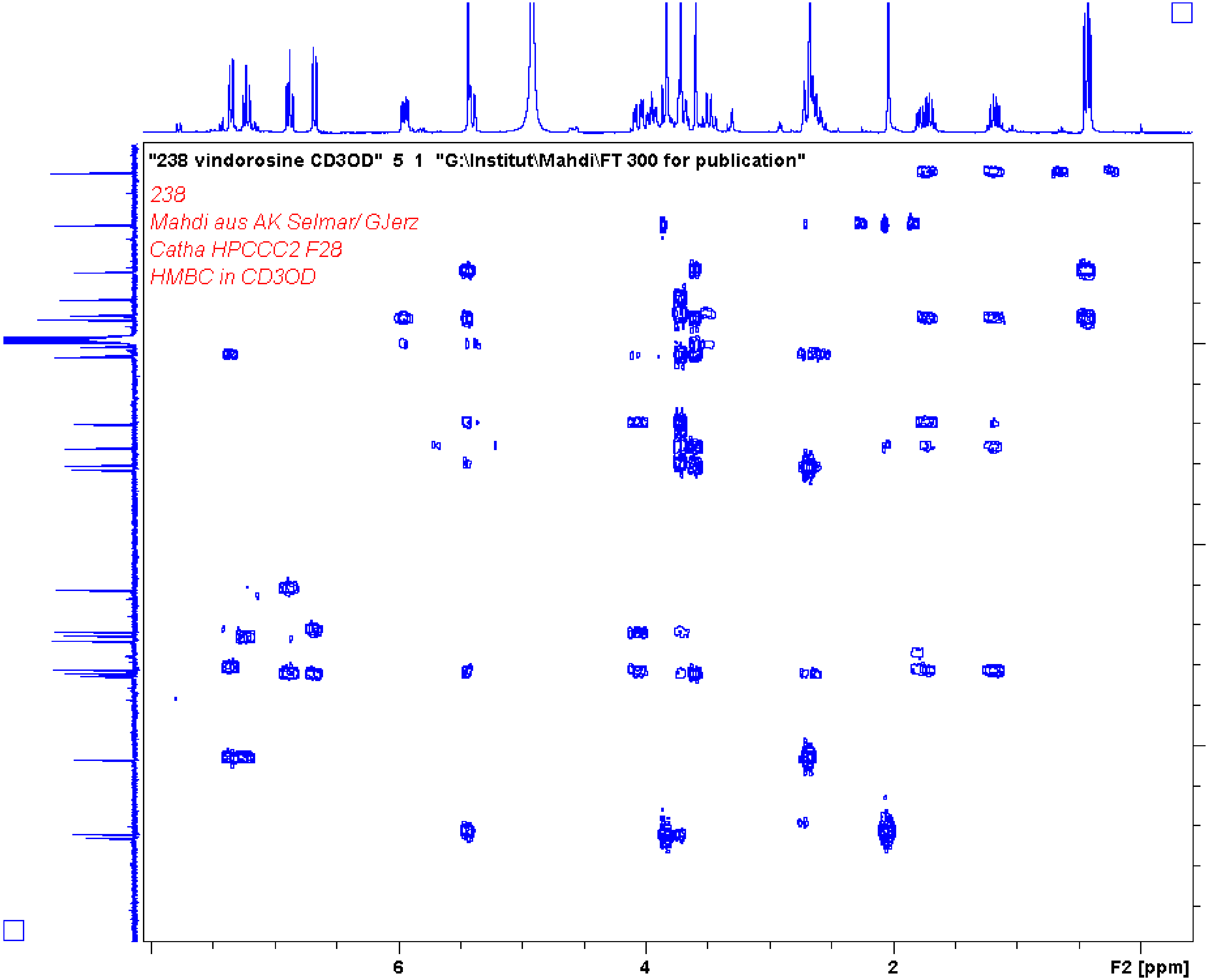

Figure NMR-S5

HMBC, long-range $^{2,3}J$-HC

Vindorosine (*427*) in CD_3OD

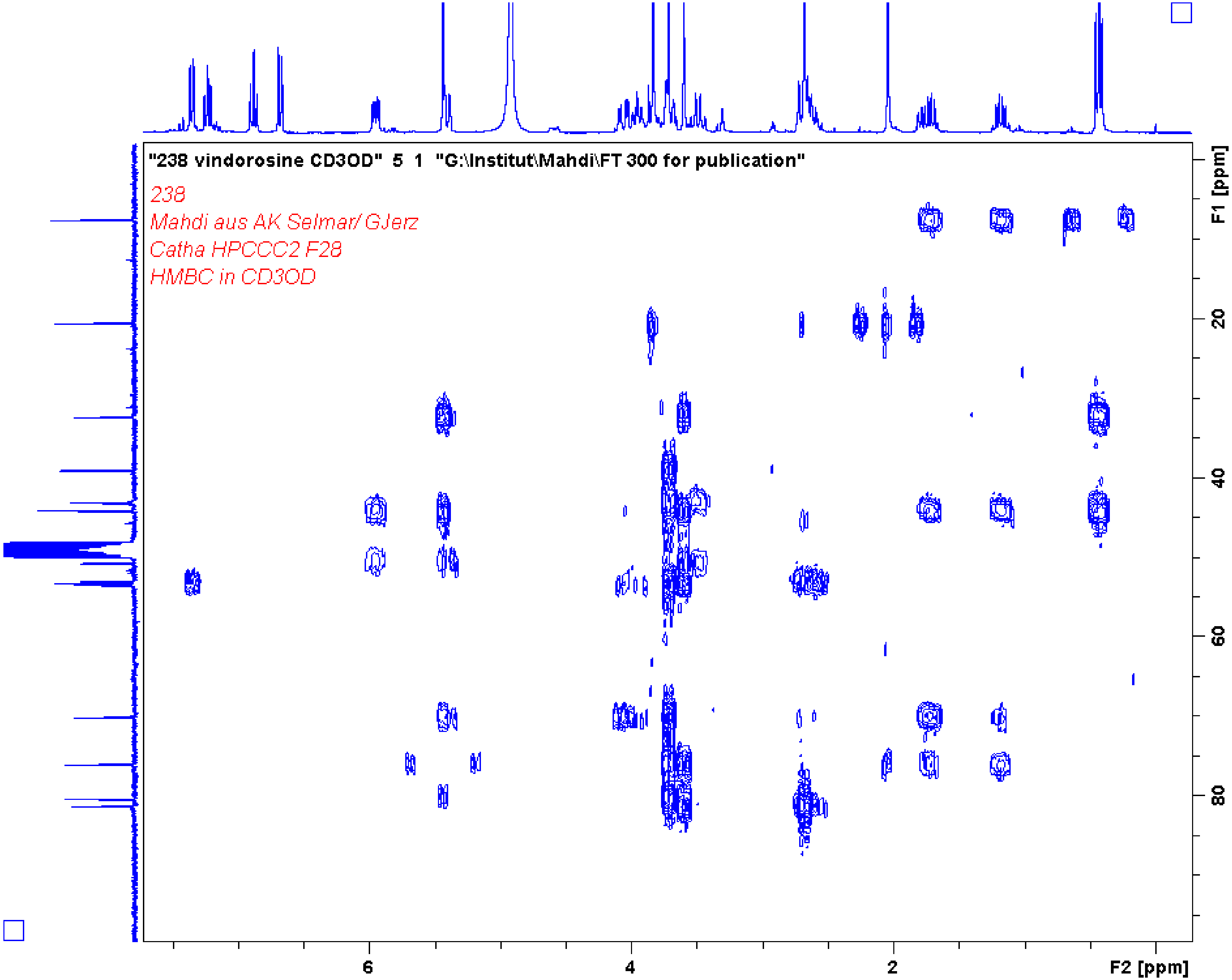

Figure NMR-S5

HMBC, long-range $^{2,3}J$-HC

Vindorosine (*427*) in CD_3OD

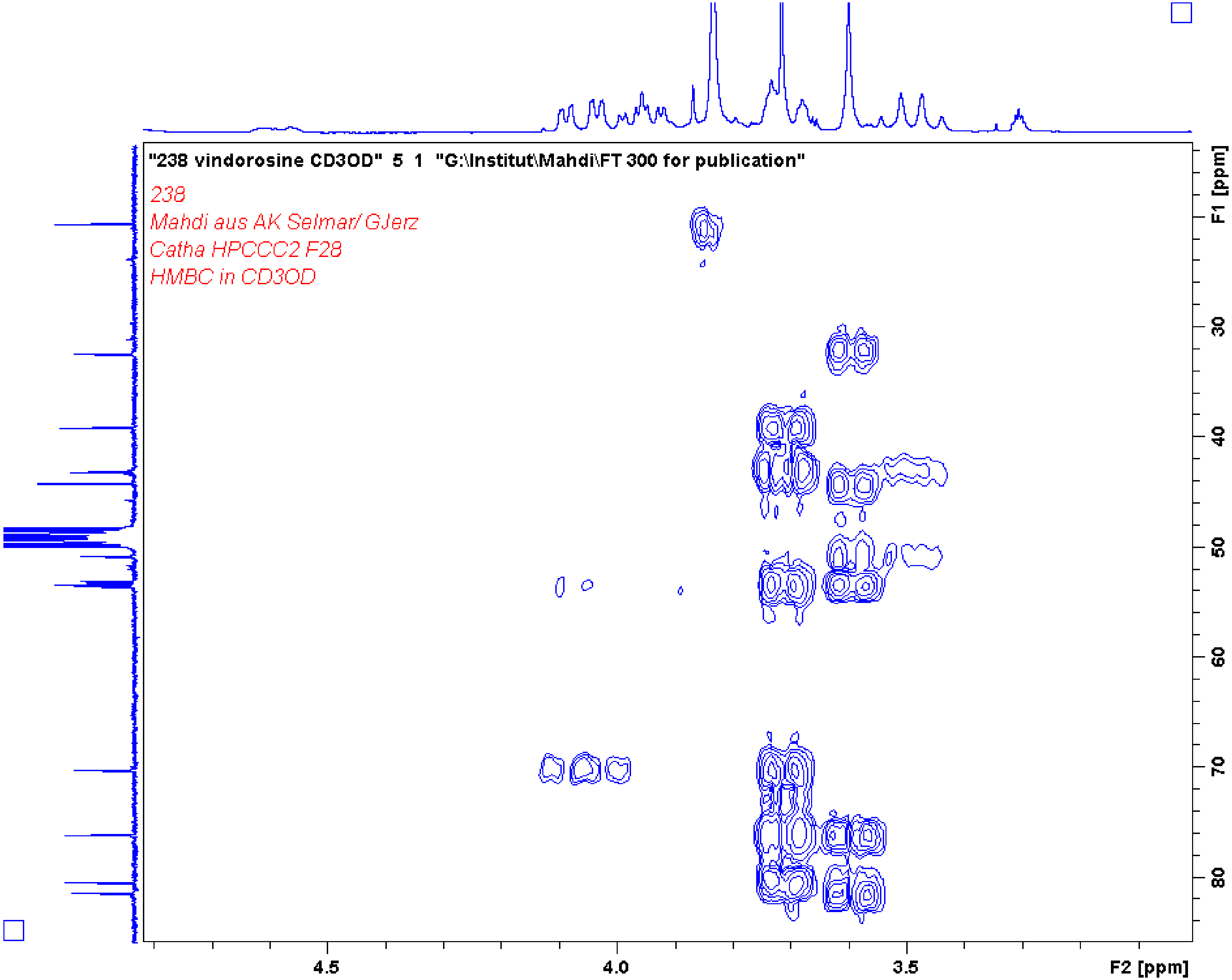

Figure NMR-S5

HMBC, long-range $^{2,3}J$-*HC*

Vindorosine (*427*) in CD_3OD

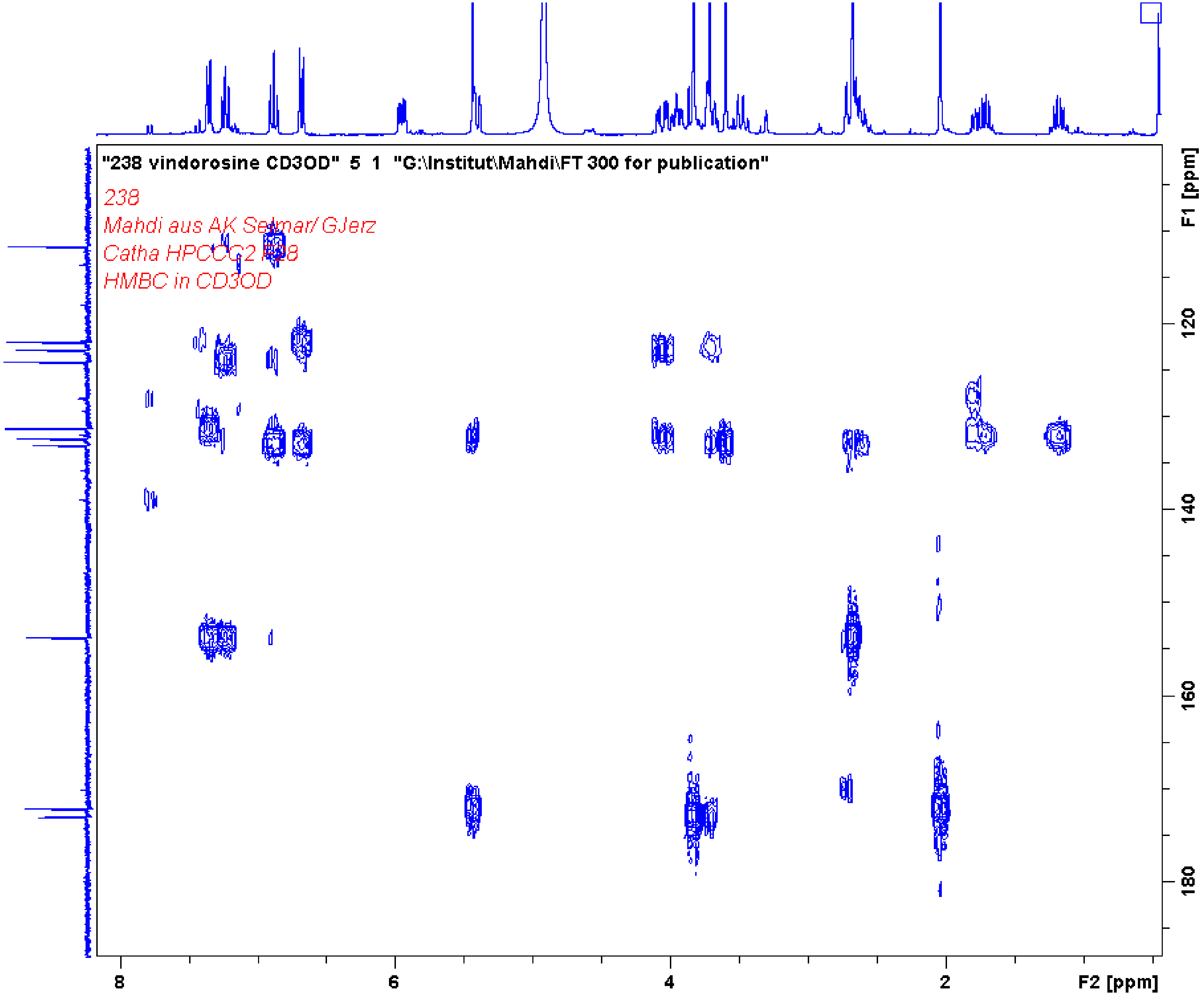

Figure NMR-S5

$^{1}H/^{1}H$-NOESY
Vindorosine (*427*) in CD_3OD

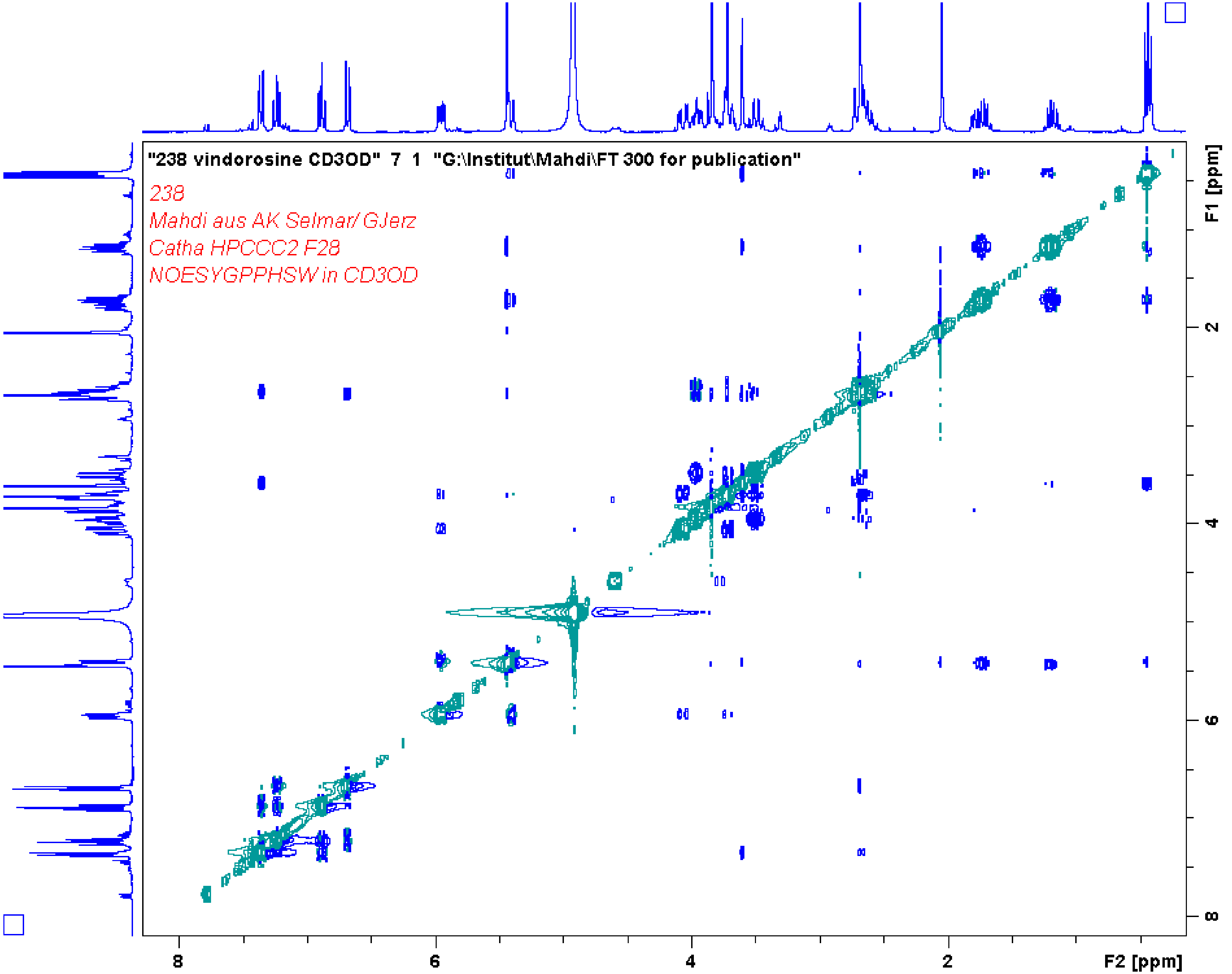

Figure NMR-S5

^1H/^1H-NOESY
Vindorosine (*427*) in CD_3OD

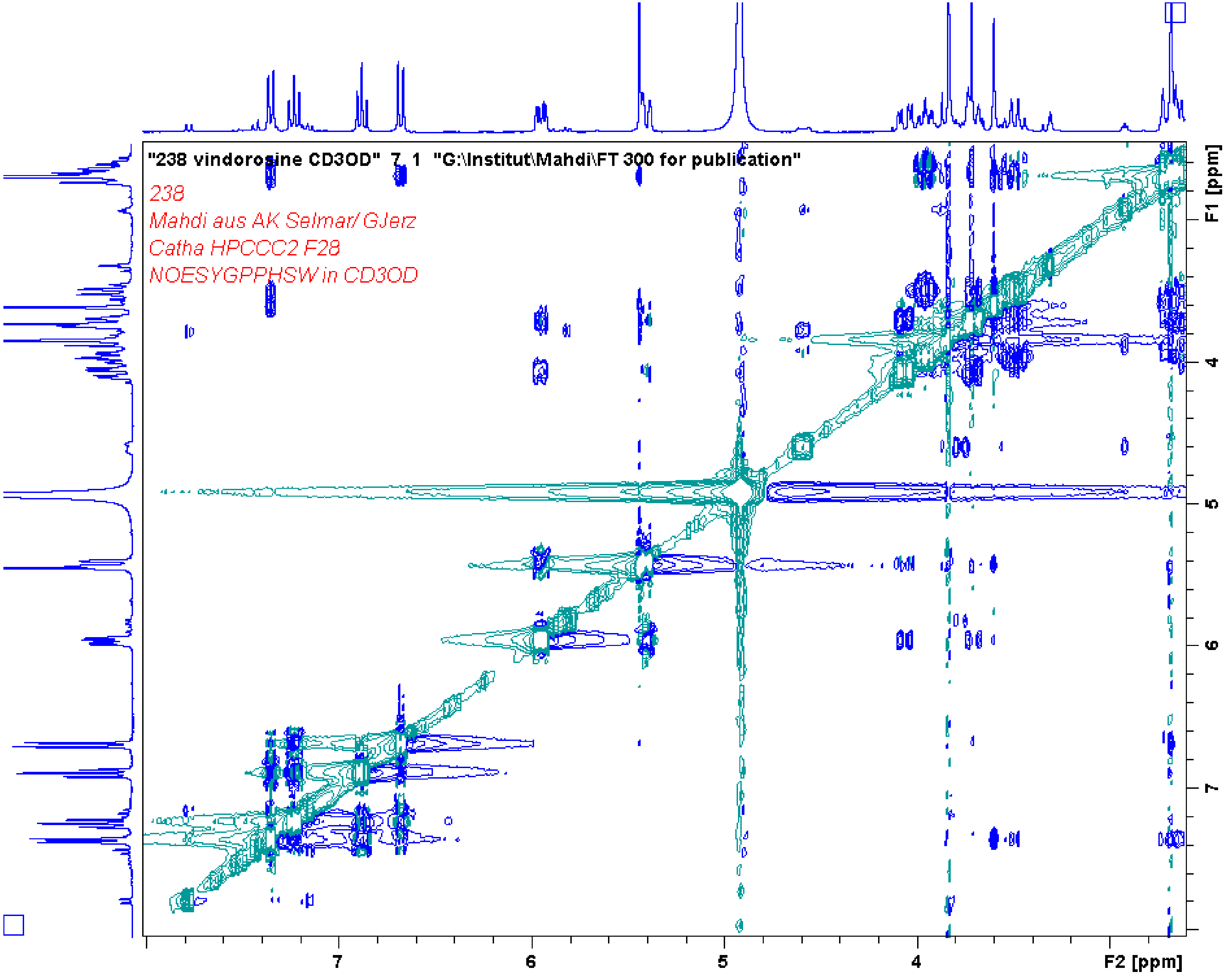

Figure NMR-S5

$^{1}H/^{1}H$-NOESY
Vindorosine (*427*) in CD_3OD

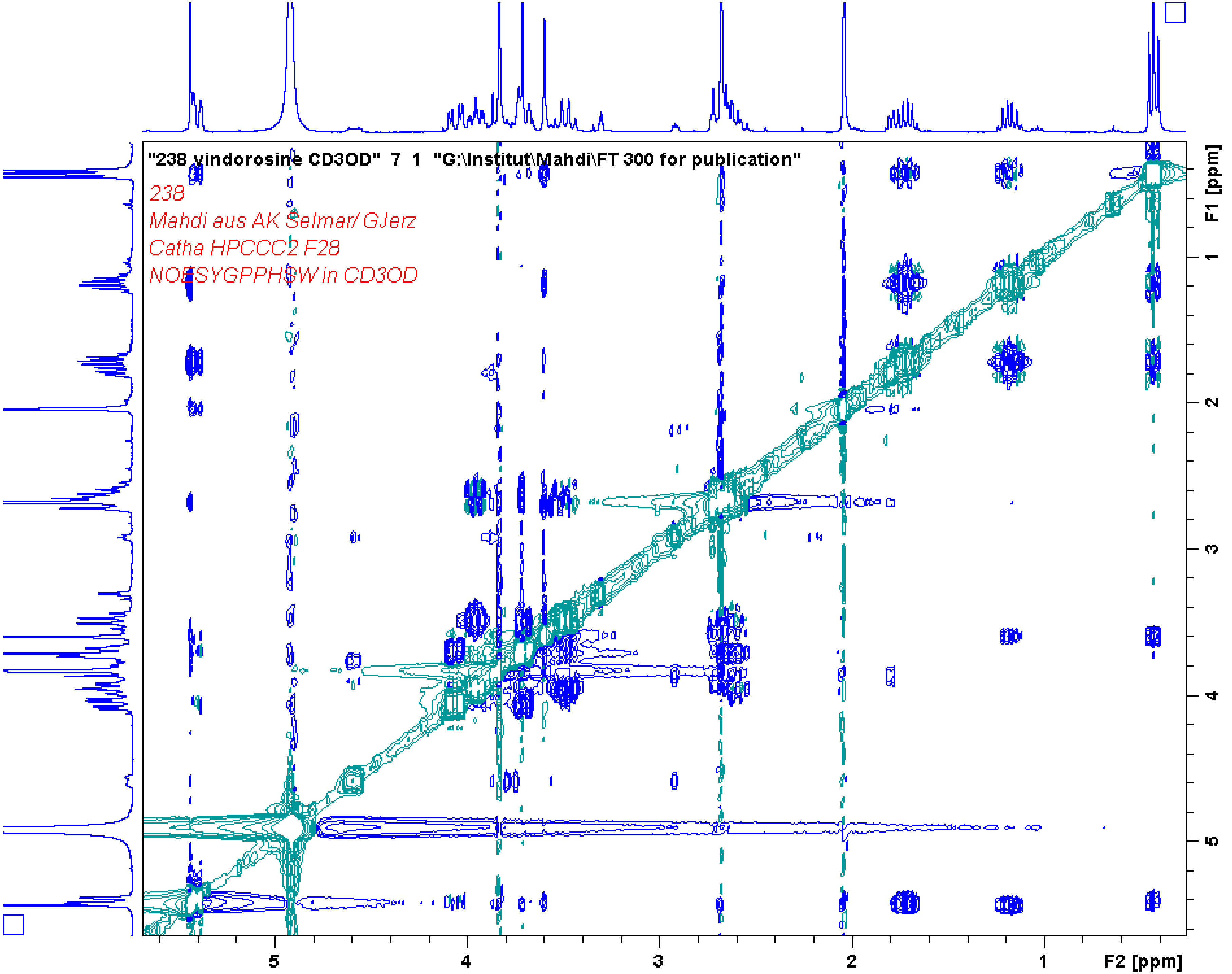

Figure NMR-S6

¹H NMR – Vindorosine (*427*) in C_6D_6 (300 MHz)

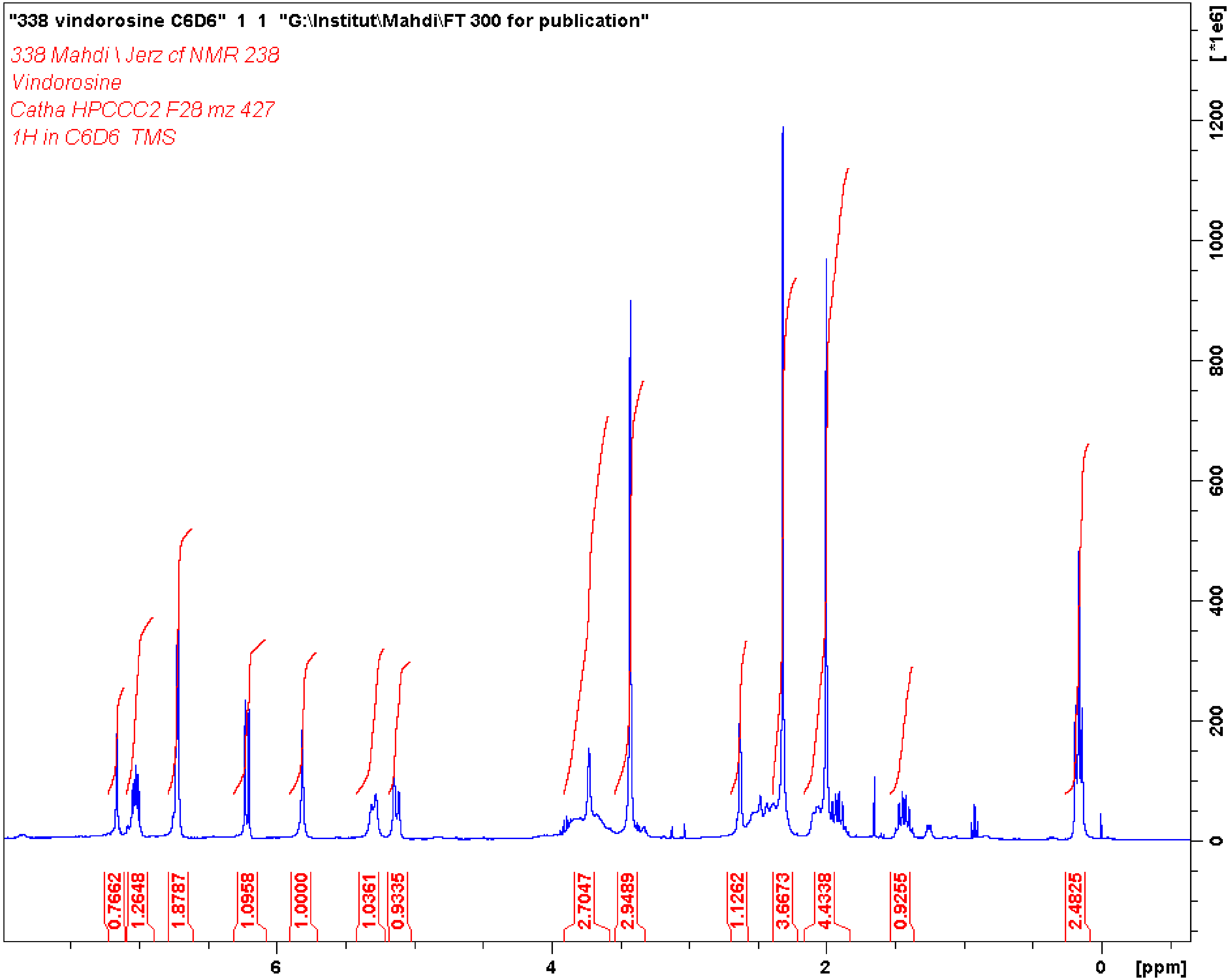

Figure NMR-S6

^{1}H NMR – Vindorosine (*427*) in C_6D_6 (300 MHz)

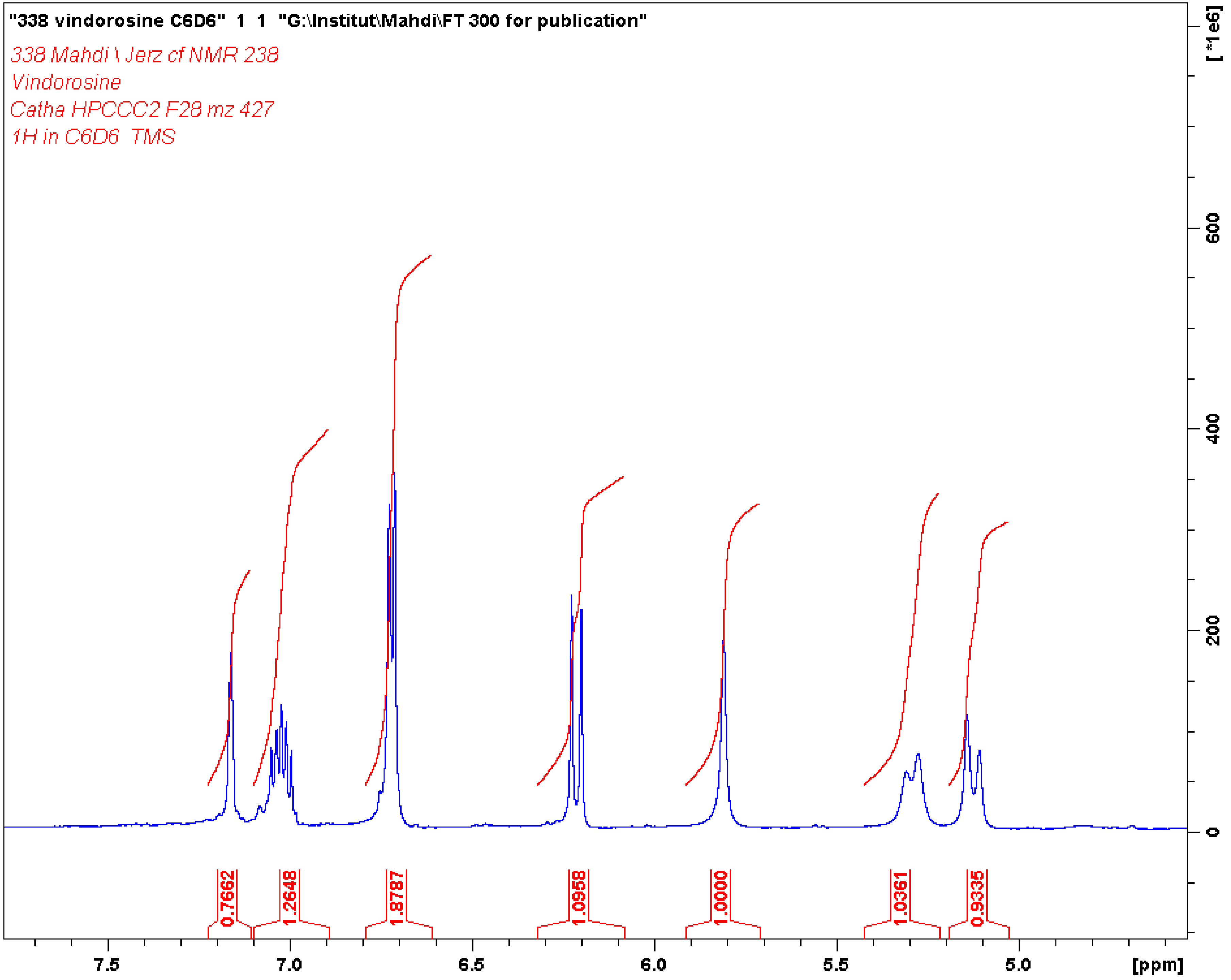

Figure NMR-S6

^{13}C NMR – Vindorosine (*427*) in C_6D_6 (75 MHz)

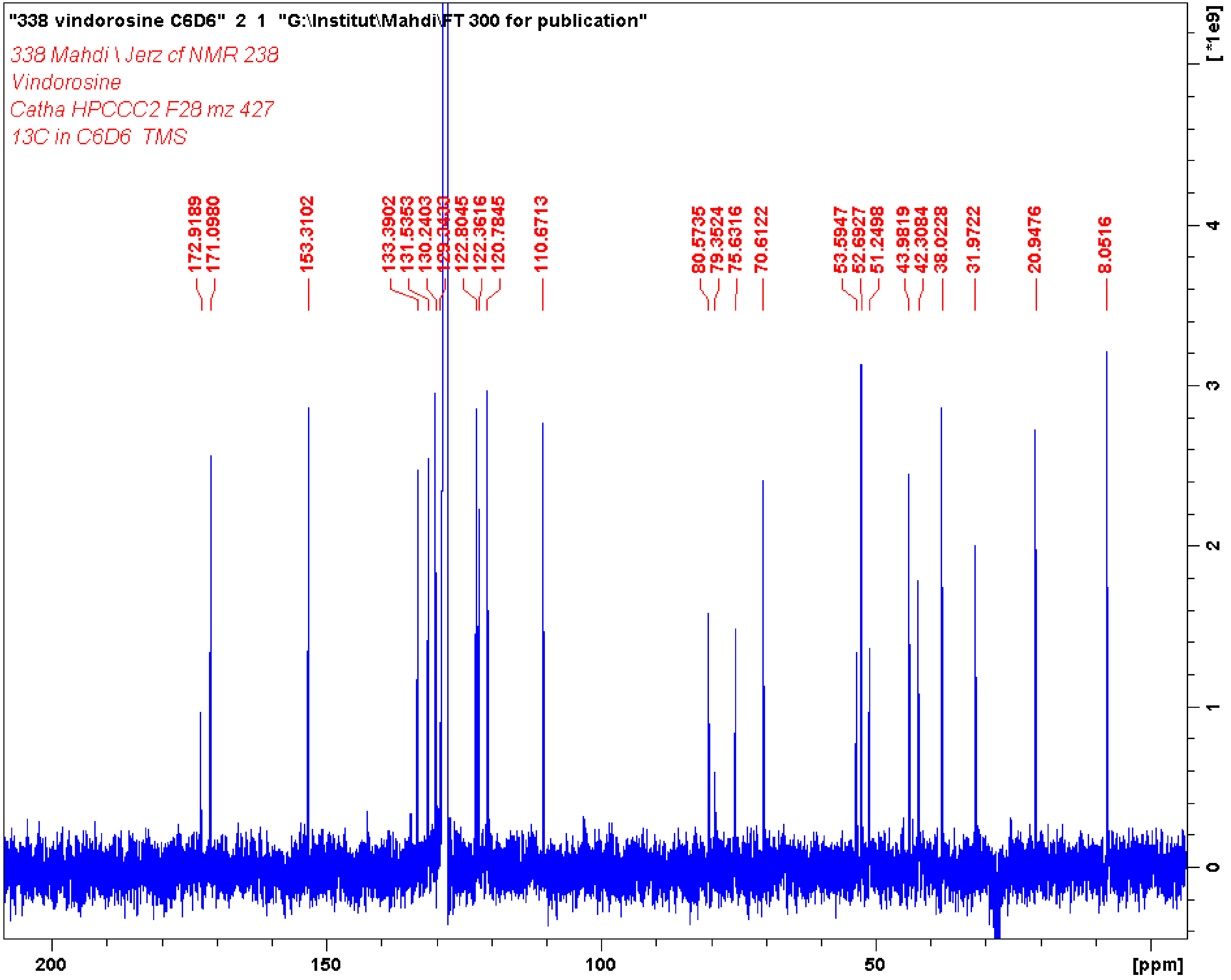

Figure NMR-S6

^{13}C NMR – Vindorosine (*427*) in C_6D_6 (75 MHz)

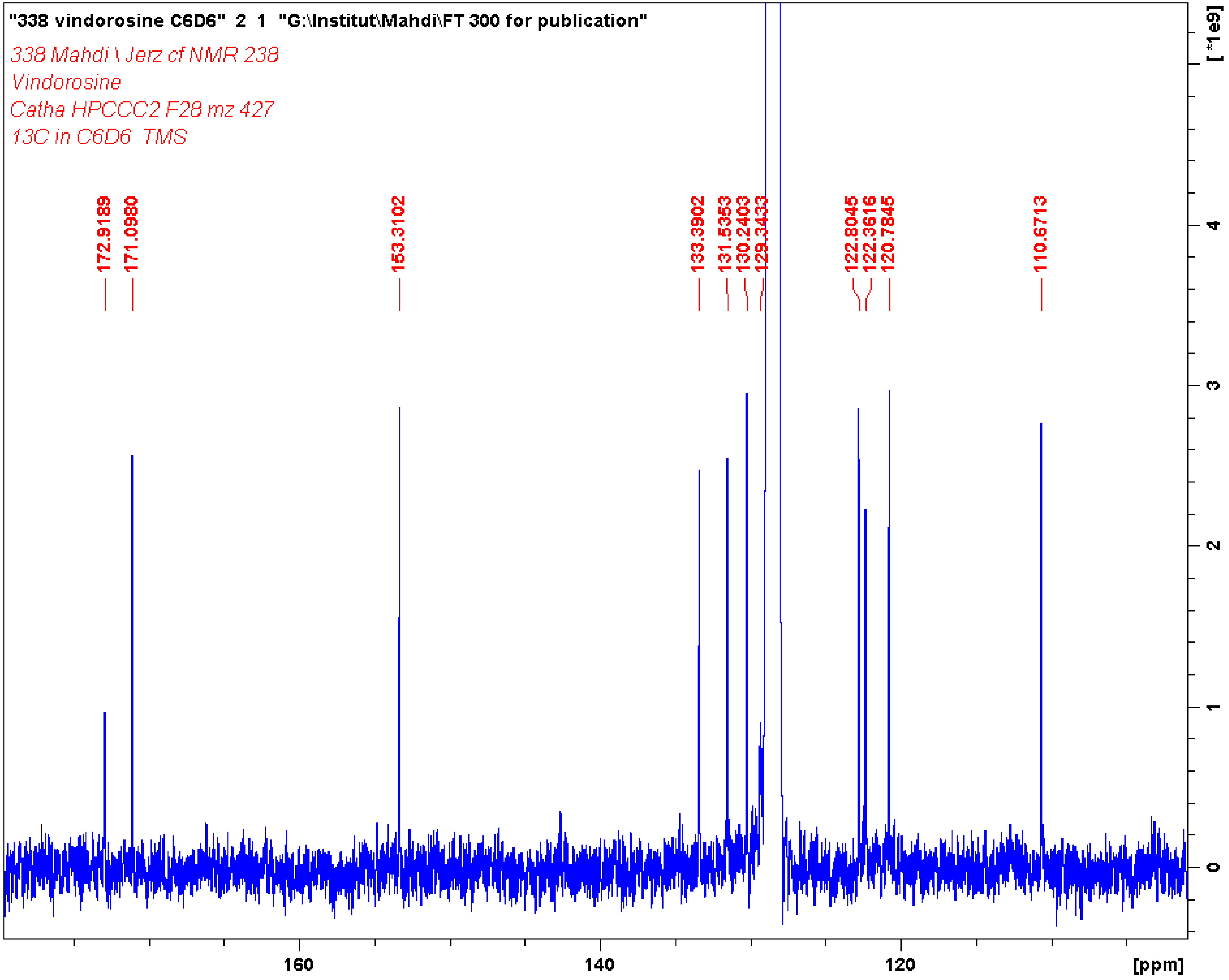

Figure NMR-S6

^{13}C NMR – Vindorosine (*427*) in C_6D_6 (75 MHz)

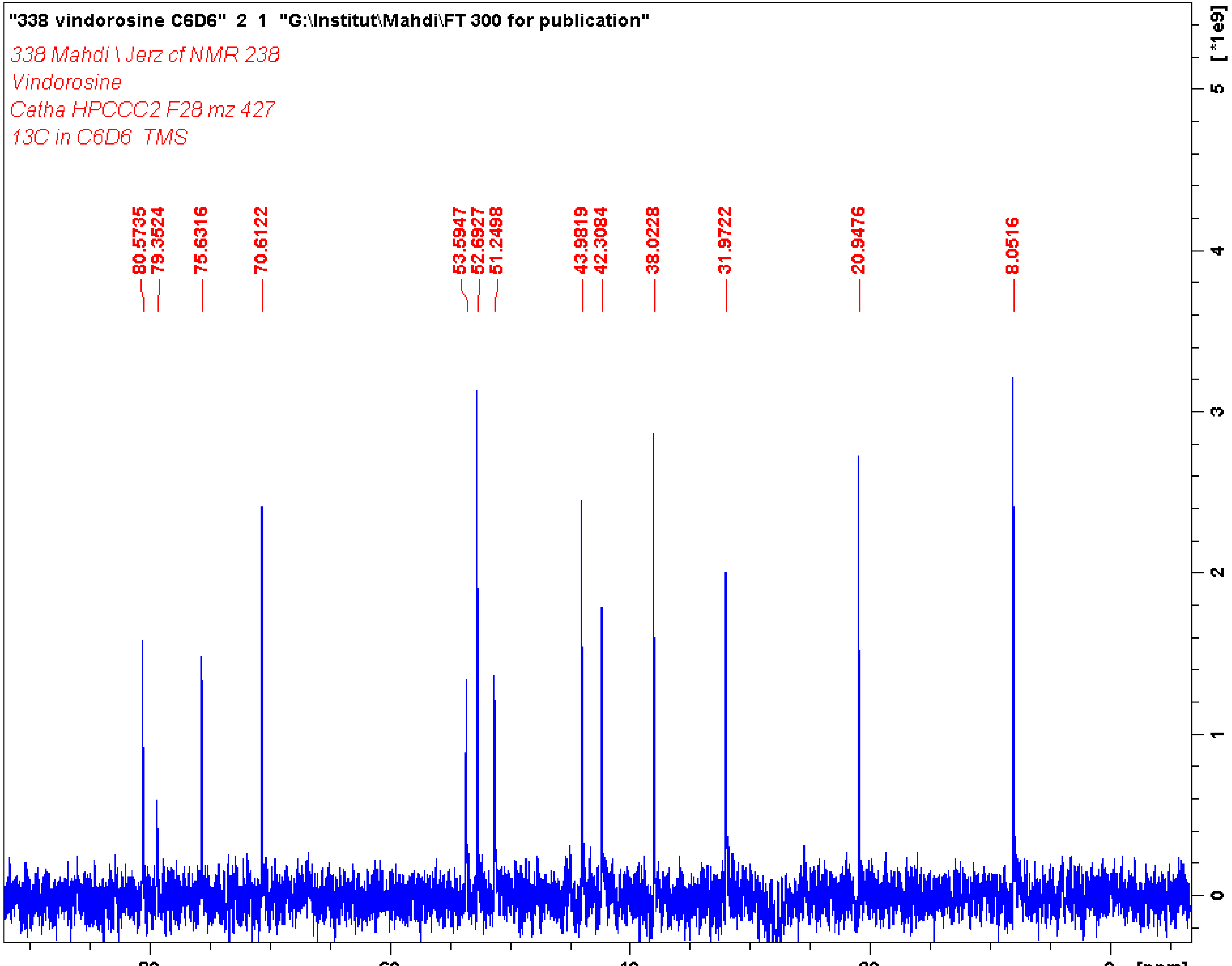

Figure NMR-S6

DEPT 135 NMR – Vindorosine (*427*) in C_6D_6

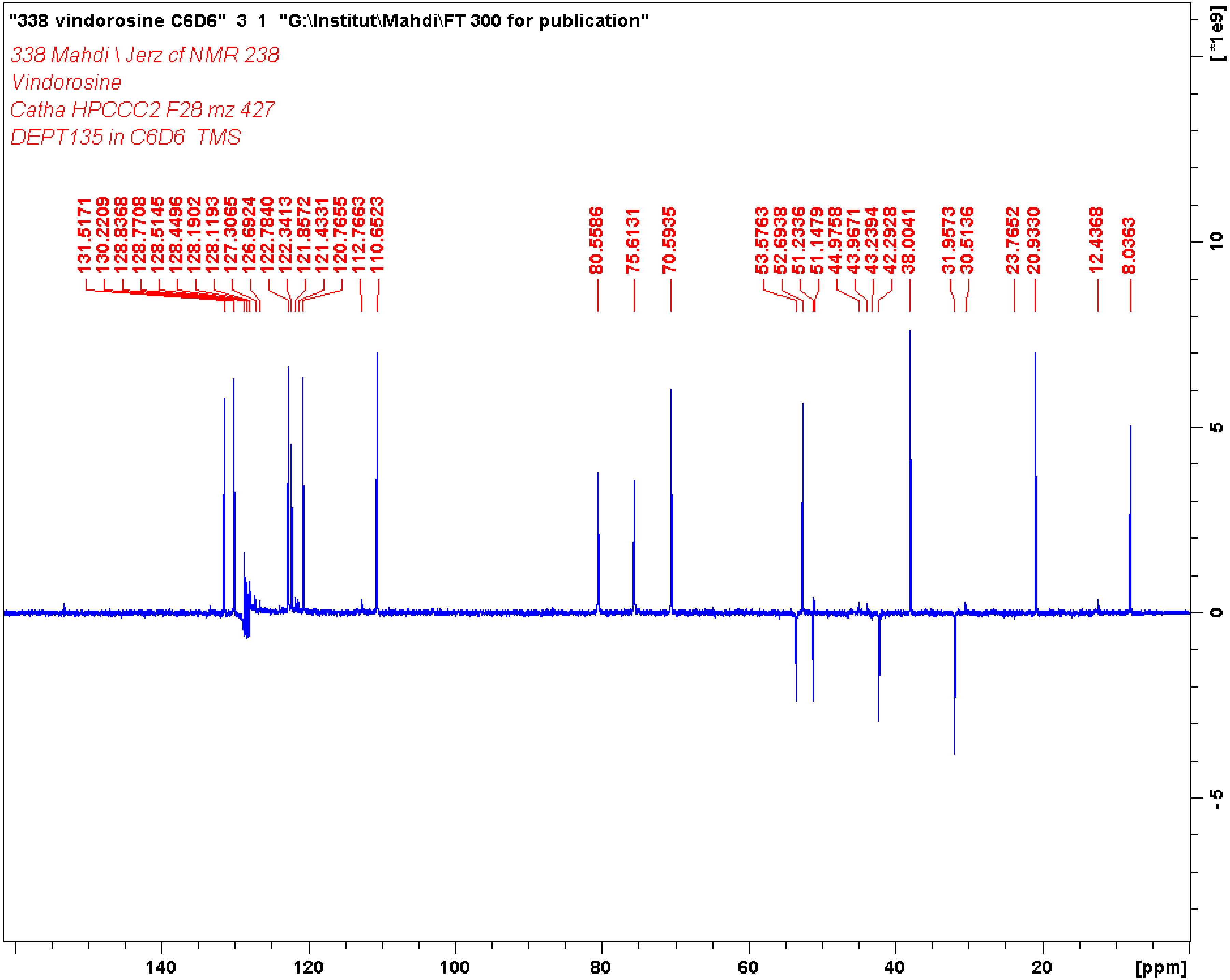

Figure NMR-S6

DEPT 135 NMR – Vindorosine (*427*) in C_6D_6

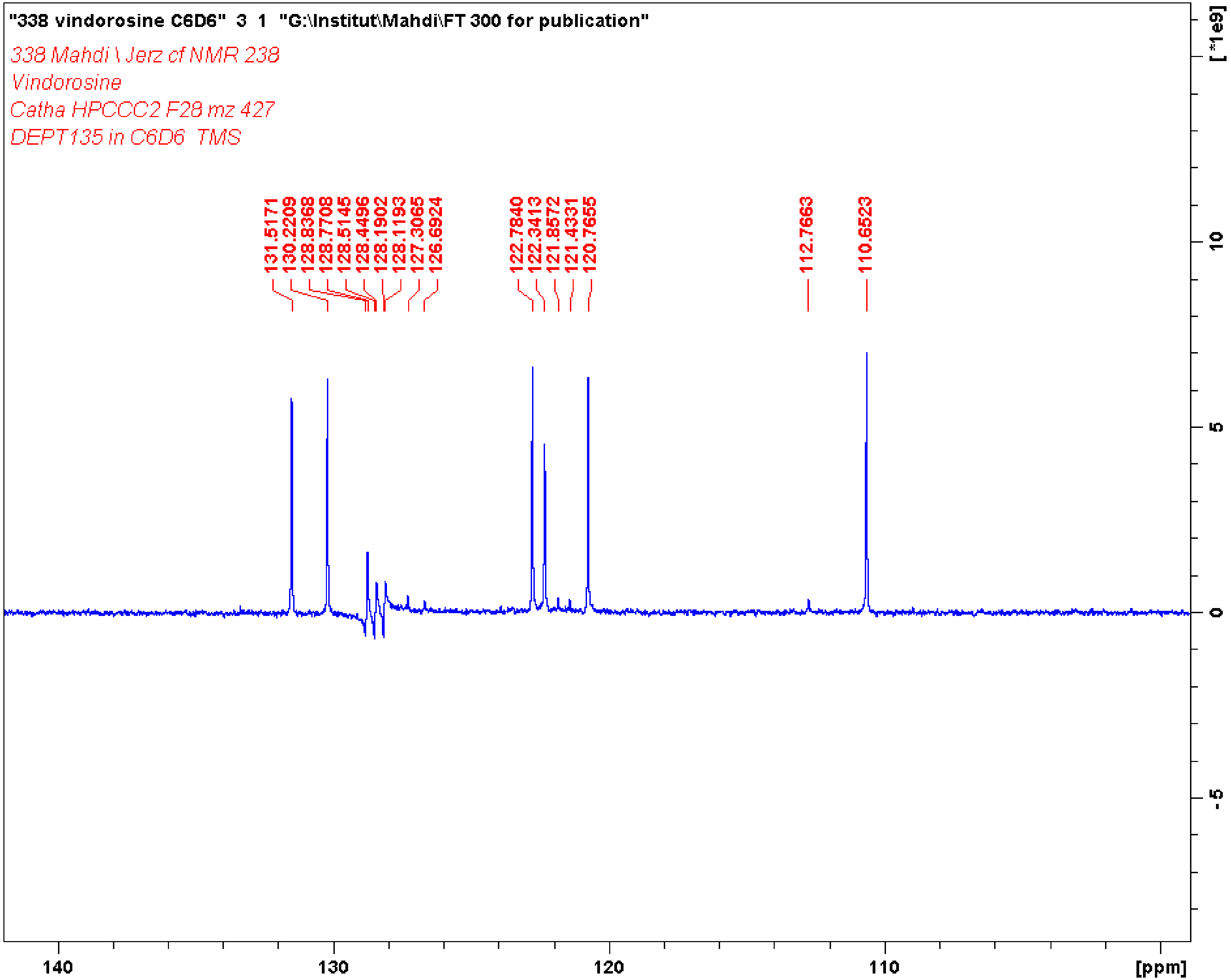

Figure NMR-S6

HSQC phase edited ^1J-HC
Vindorosine (*427*)
in C_6D_6

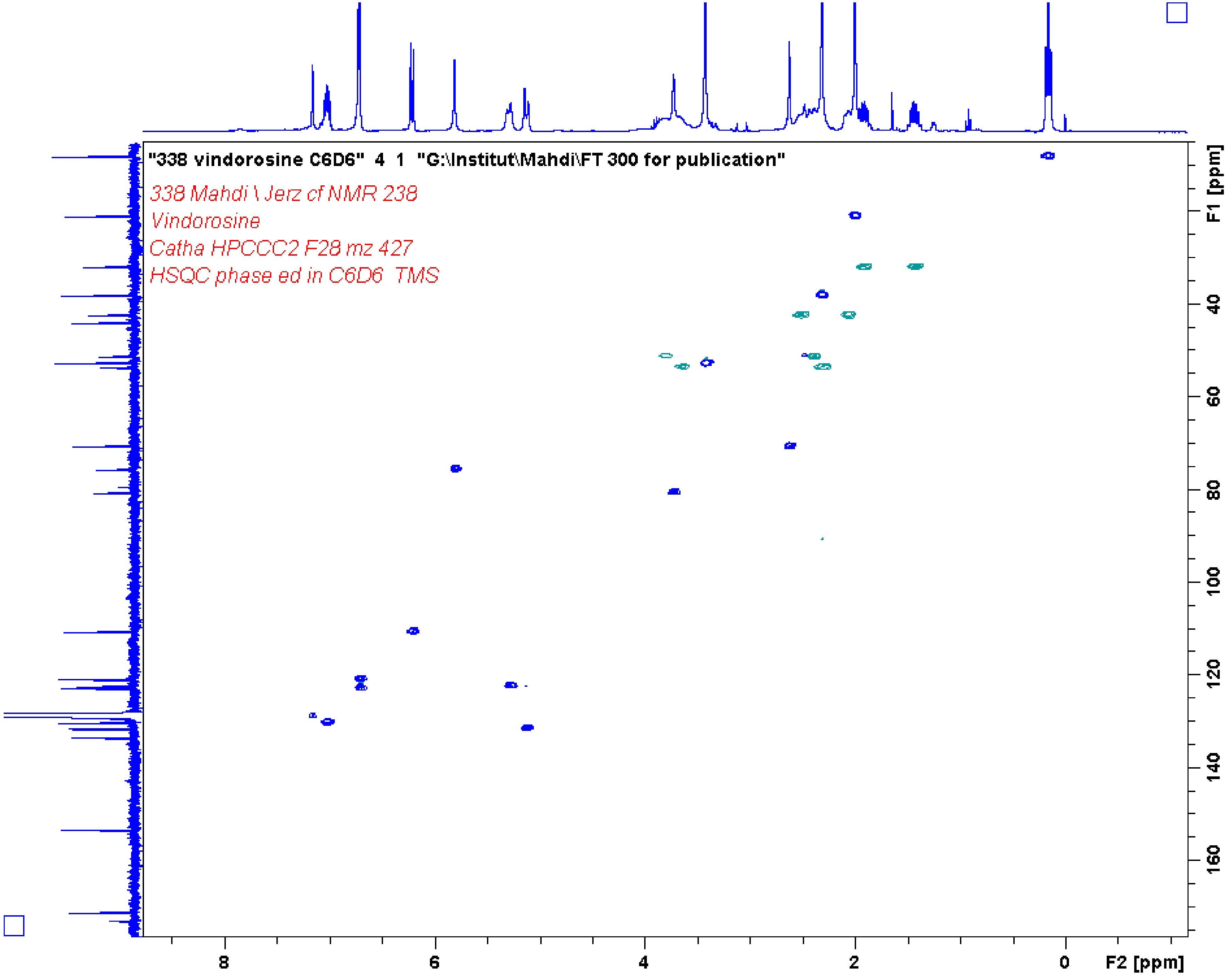

Figure NMR-S6

HSQC phase edited ^{1}J-HC
Vindorosine (*427*)
in C_6D_6

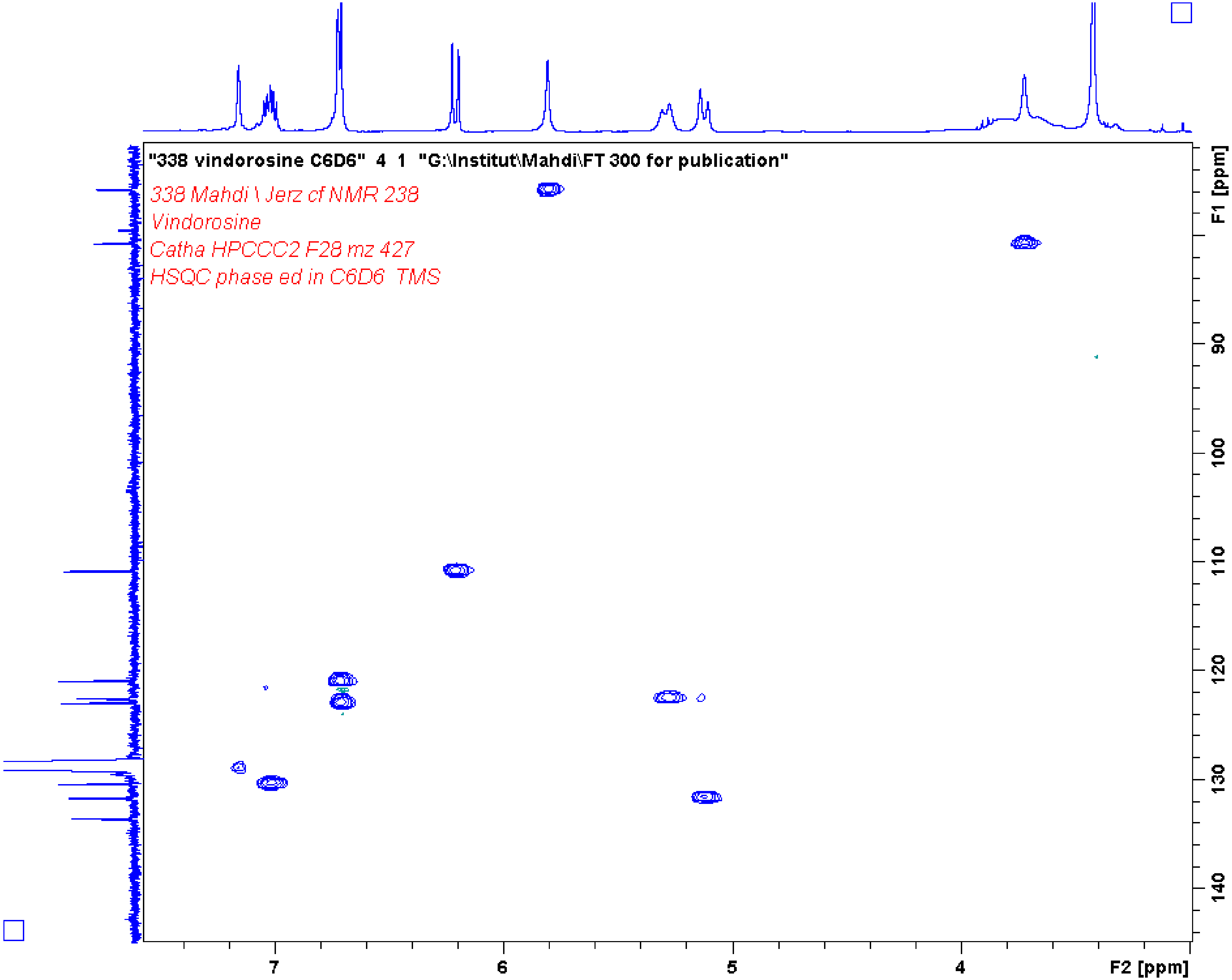

Figure NMR-S6

HSQC phase edited [1]*J-HC*
Vindorosine (*427*)
in C_6D_6

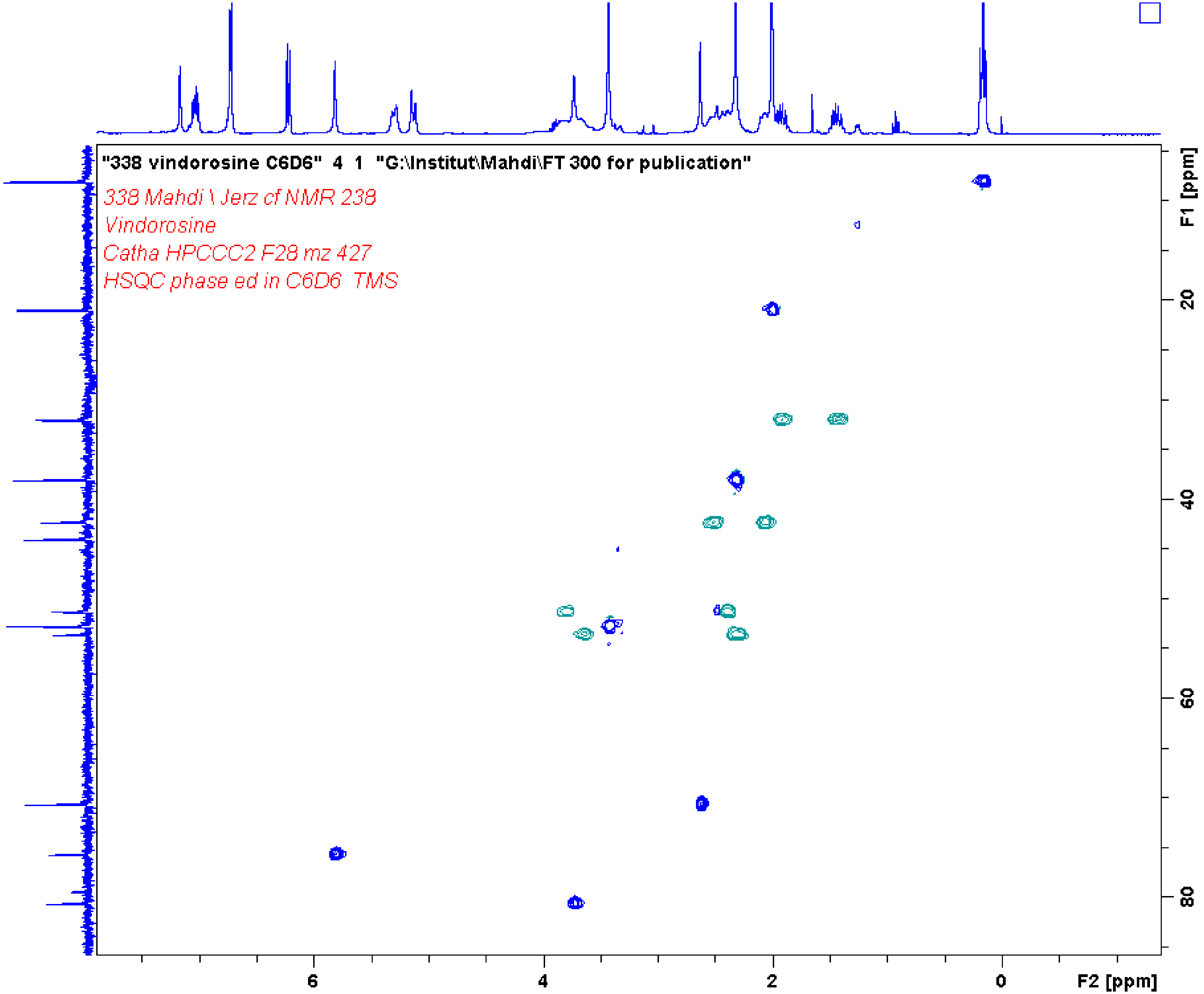

Figure NMR-S6

HMBC, long-range $^{2,3}J$-*HC*

Vindorosine (*427*) in C_6D_6

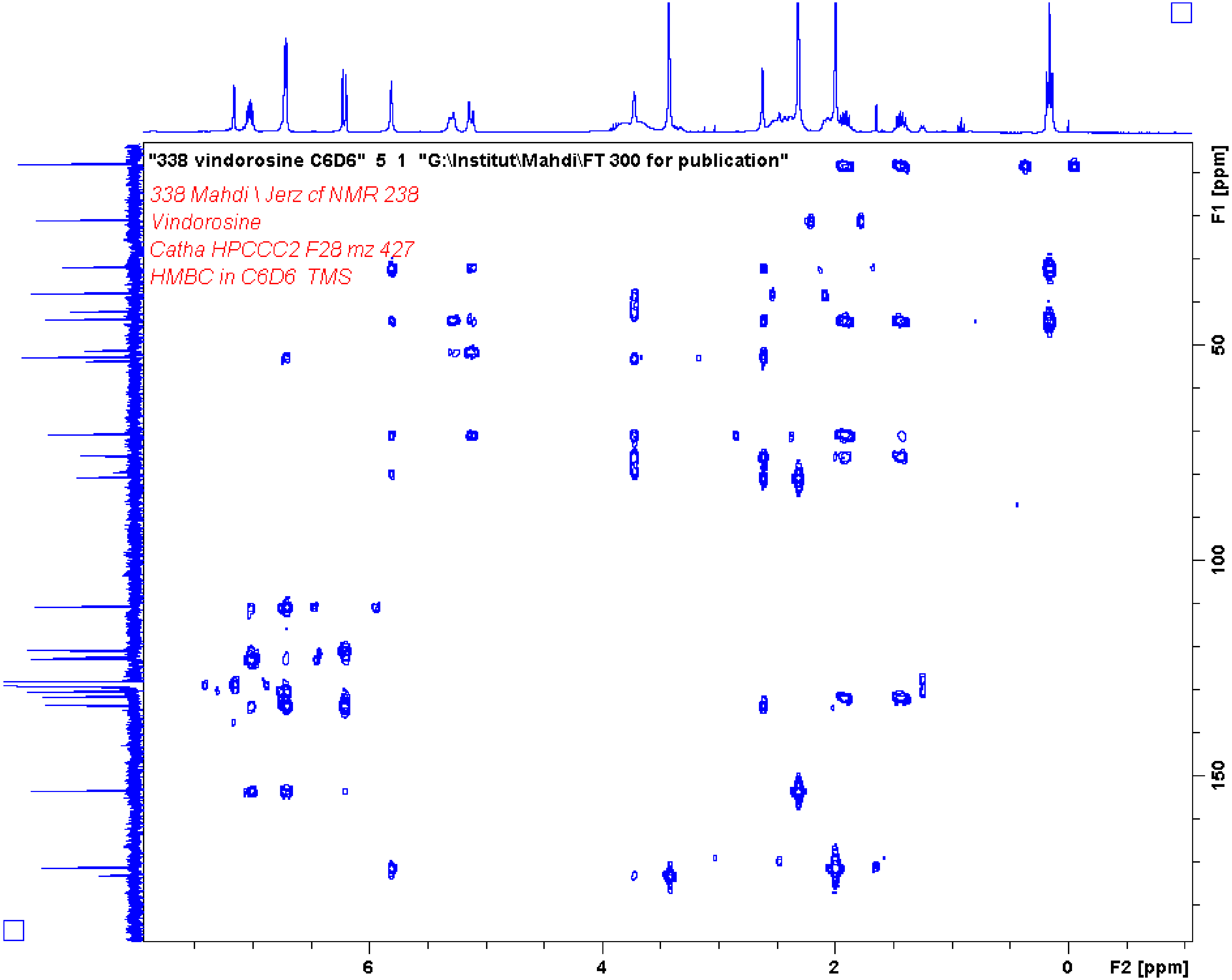

Figure NMR-S6

HMBC, long-range $^{2,3}J$-HC

Vindorosine (*427*) in C_6D_6

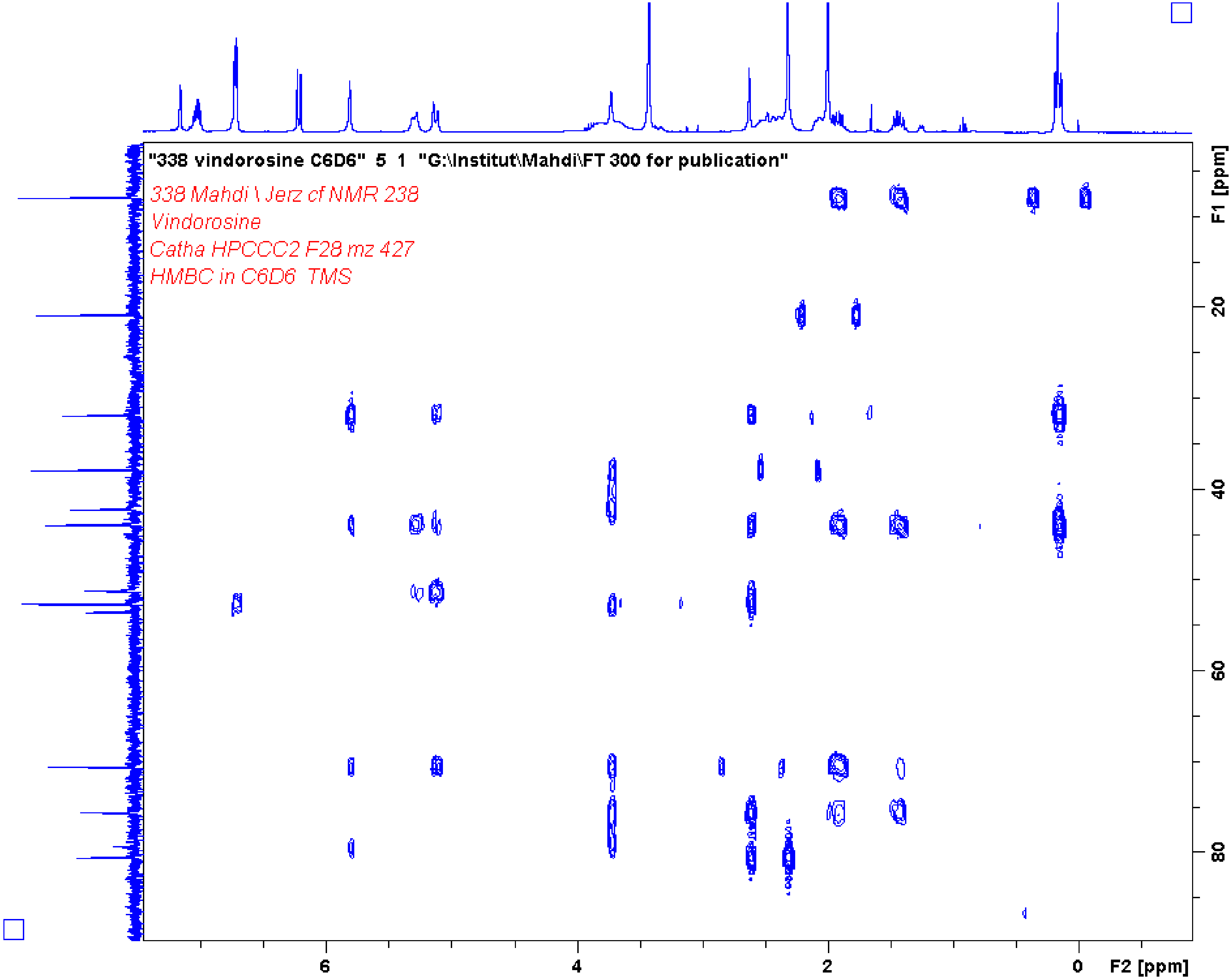

Figure NMR-S6

HMBC, long-range $^{2,3}J$-HC

Vindorosine (*427*) in C_6D_6

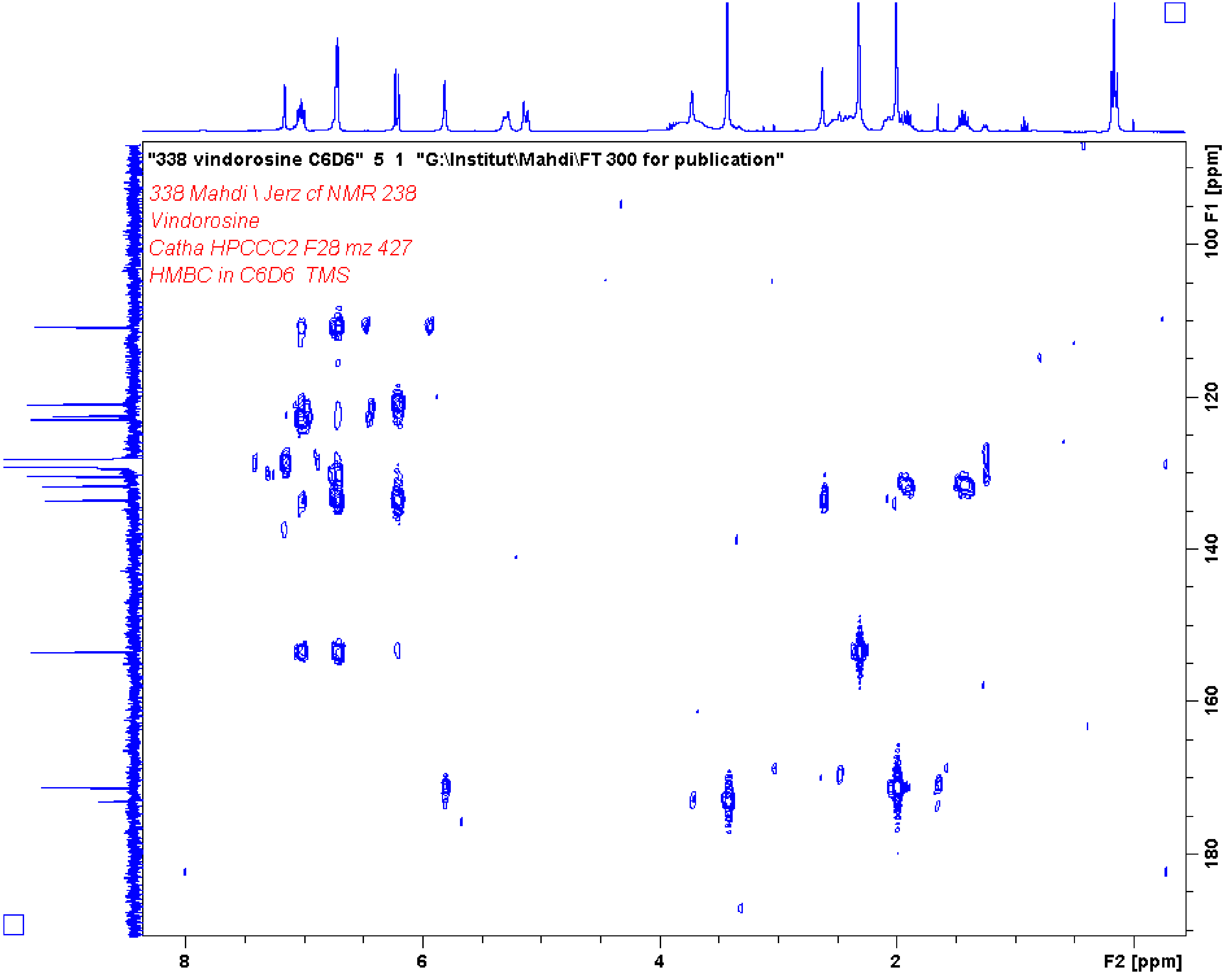

Figure NMR-S7

¹H NMR – Vindolinine (*337-b*) in CD_3OD (300 MHz)

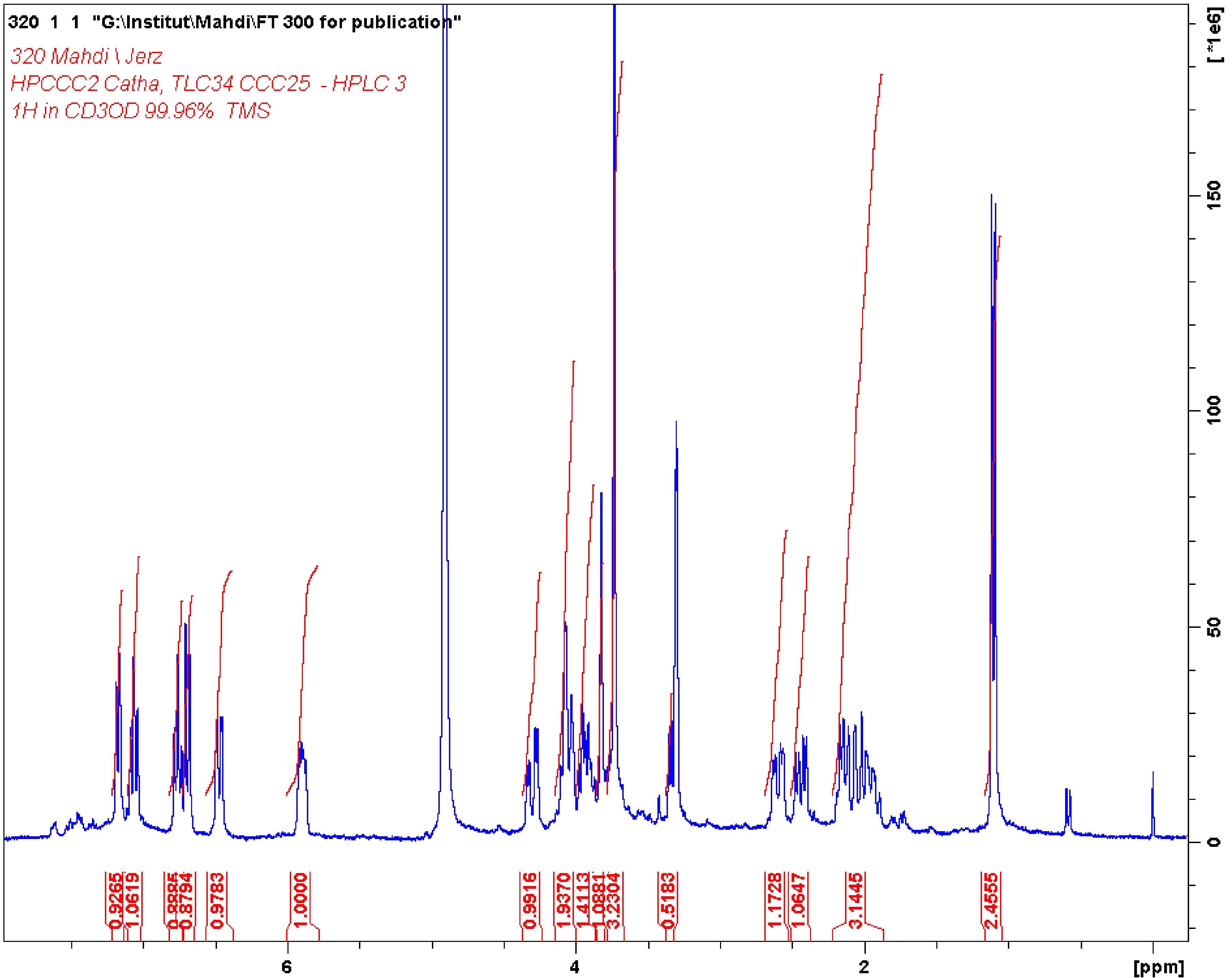

Figure NMR-S7

^{1}H NMR – Vindolinine (*337-b*)
in CD_3OD
(300 MHz)

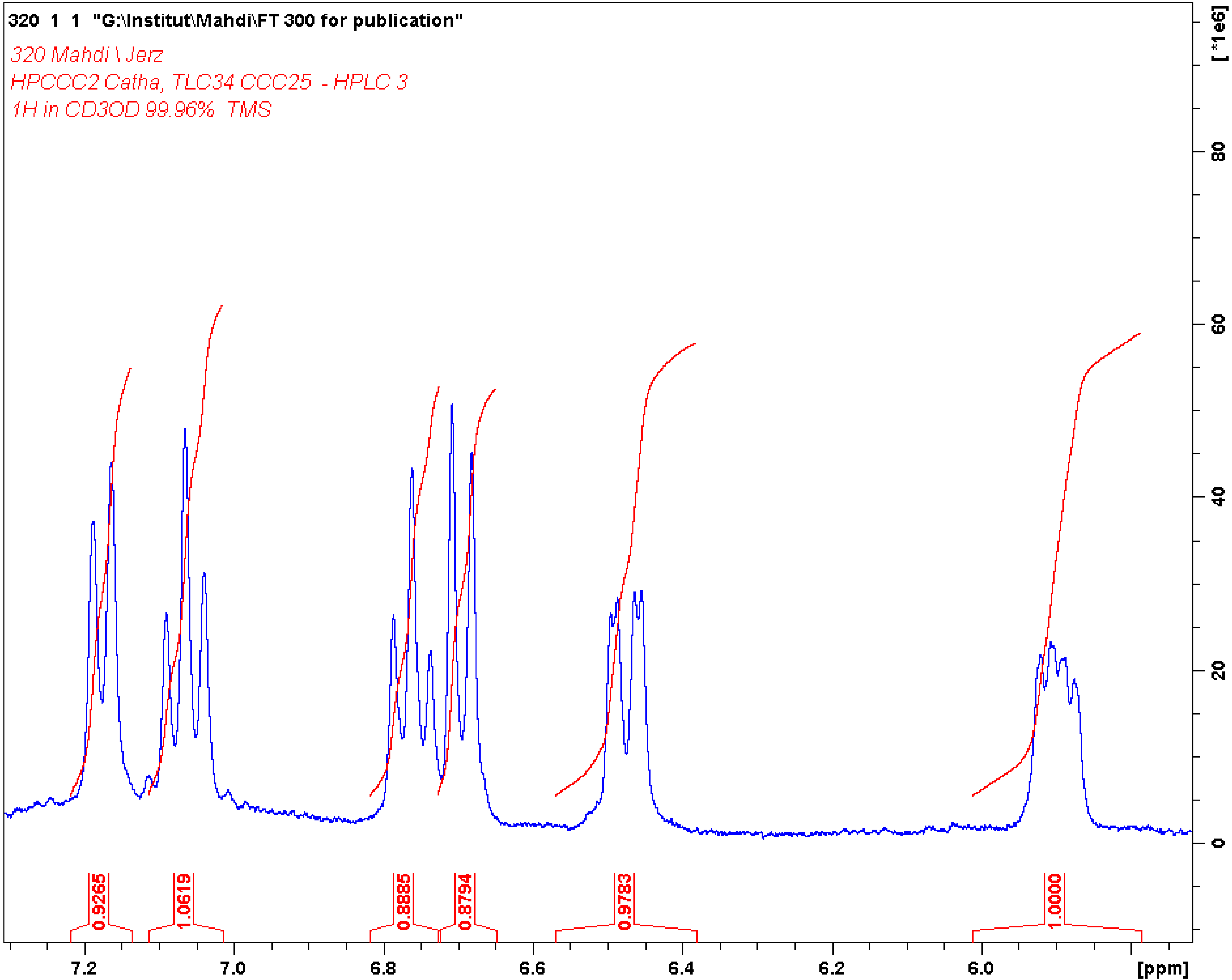

Figure NMR-S7

^{1}H NMR – Vindolinine (*337-b*)
in CD_3OD
(300 MHz)

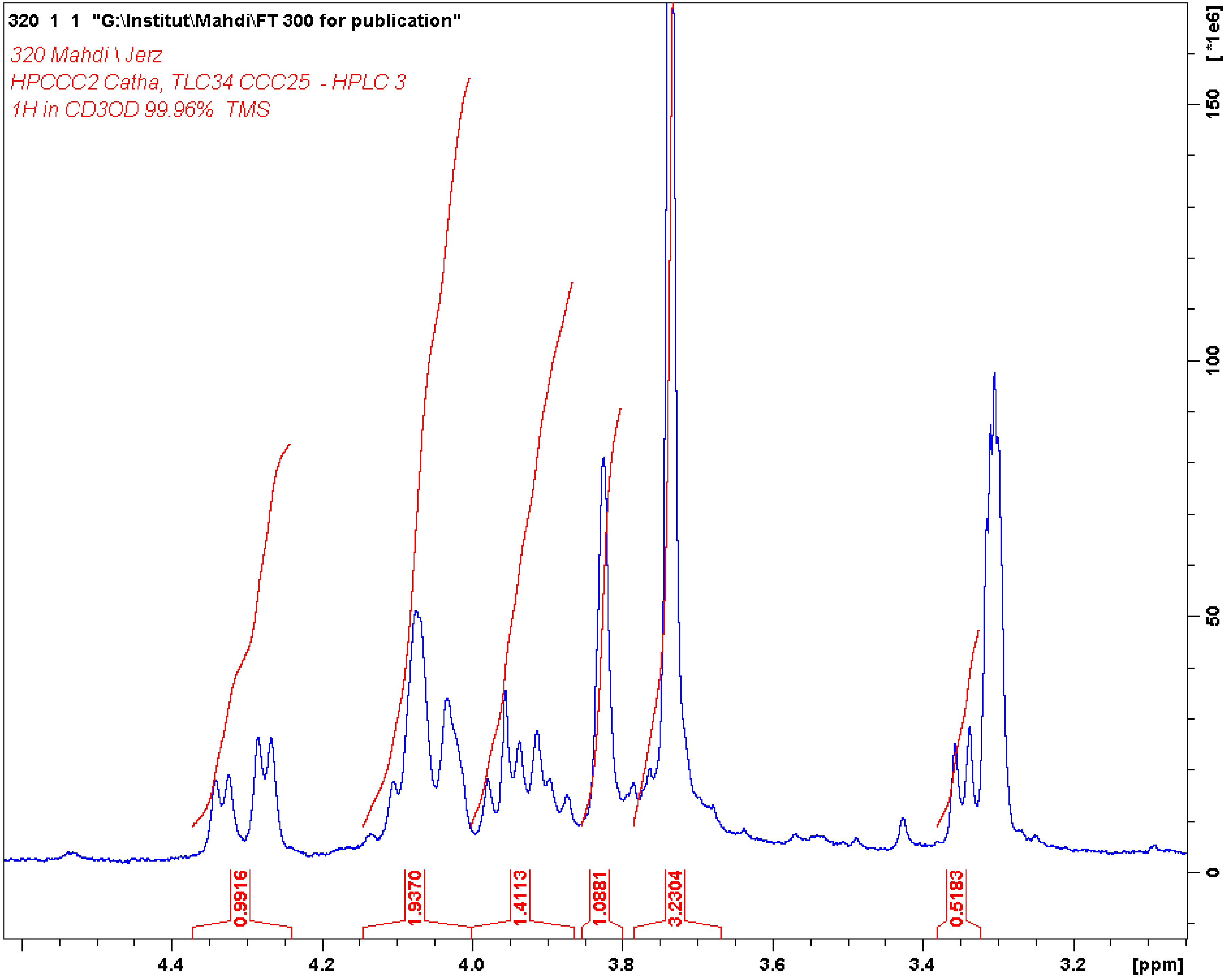

<u>**Figure NMR-S7**</u>

^{1}H NMR – Vindolinine (*337-b*)
in CD_3OD
(300 MHz)

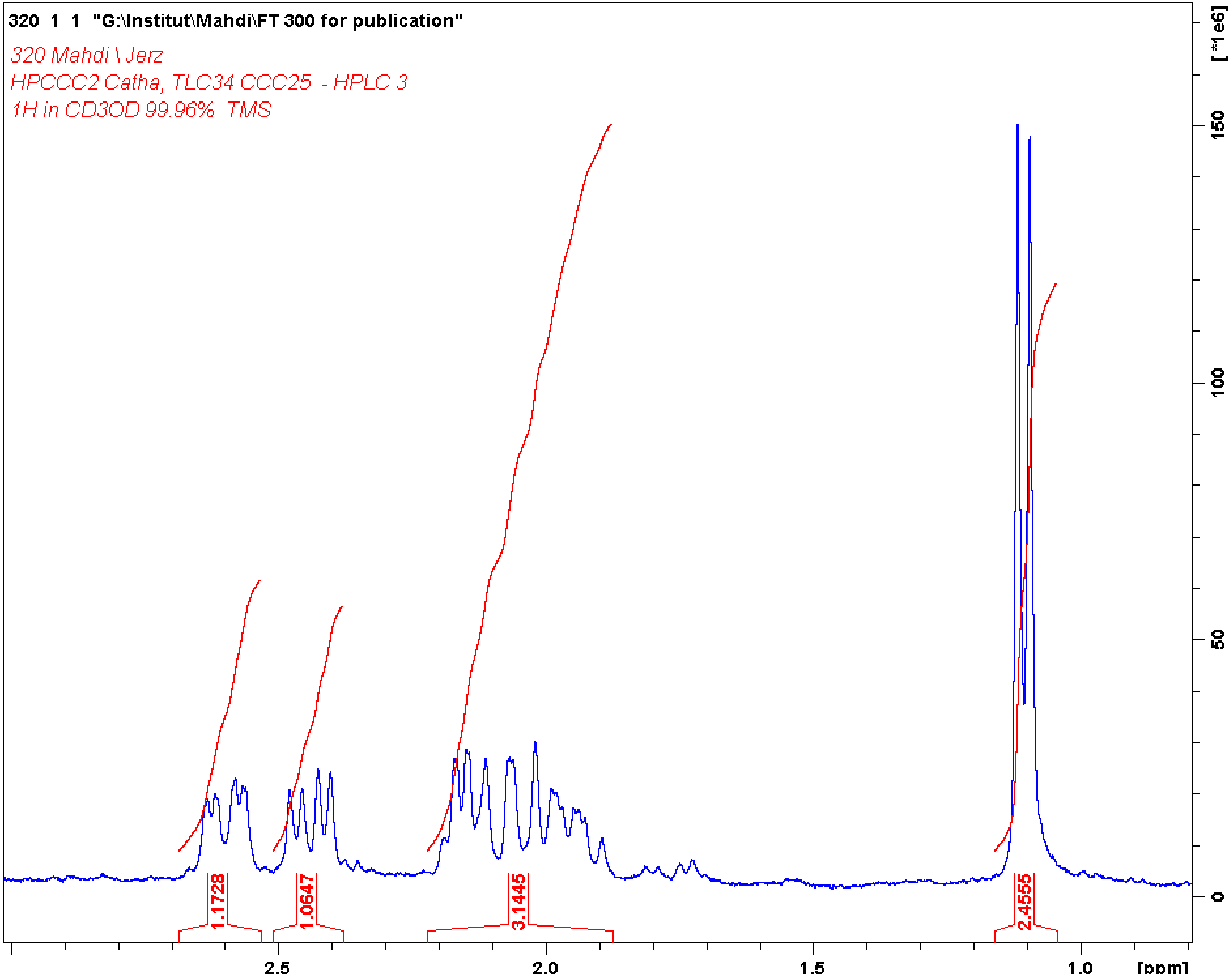

Figure NMR-S7

^{13}C NMR – Vindolinine (*337-b*)
in CD_3OD
(300 MHz)

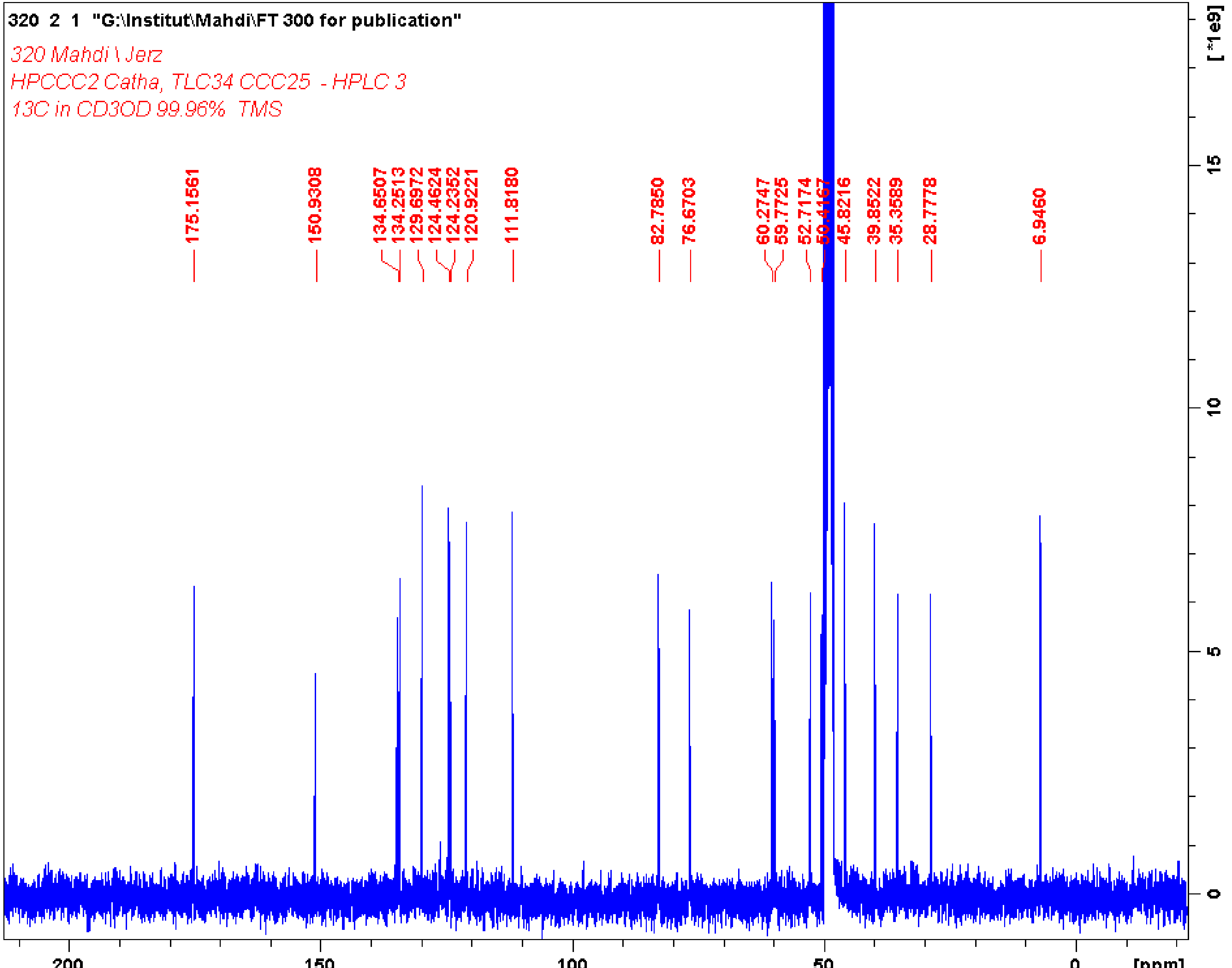

Figure NMR-S7

^{13}C NMR – Vindolinine (*337-b*) in CD_3OD (300 MHz)

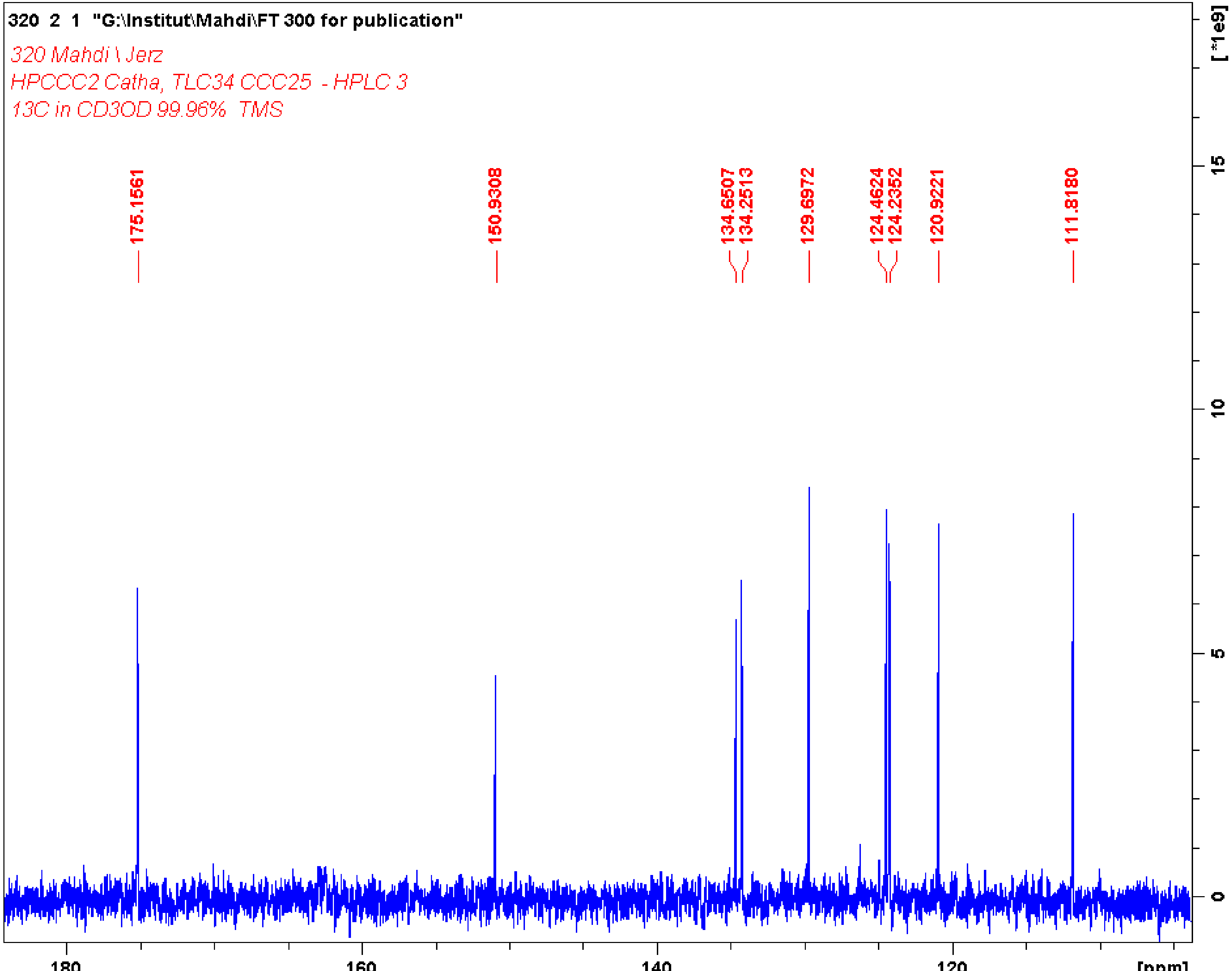

Figure NMR-S7

^{13}C NMR – Vindolinine (*337-b*)
in CD_3OD
(300 MHz)

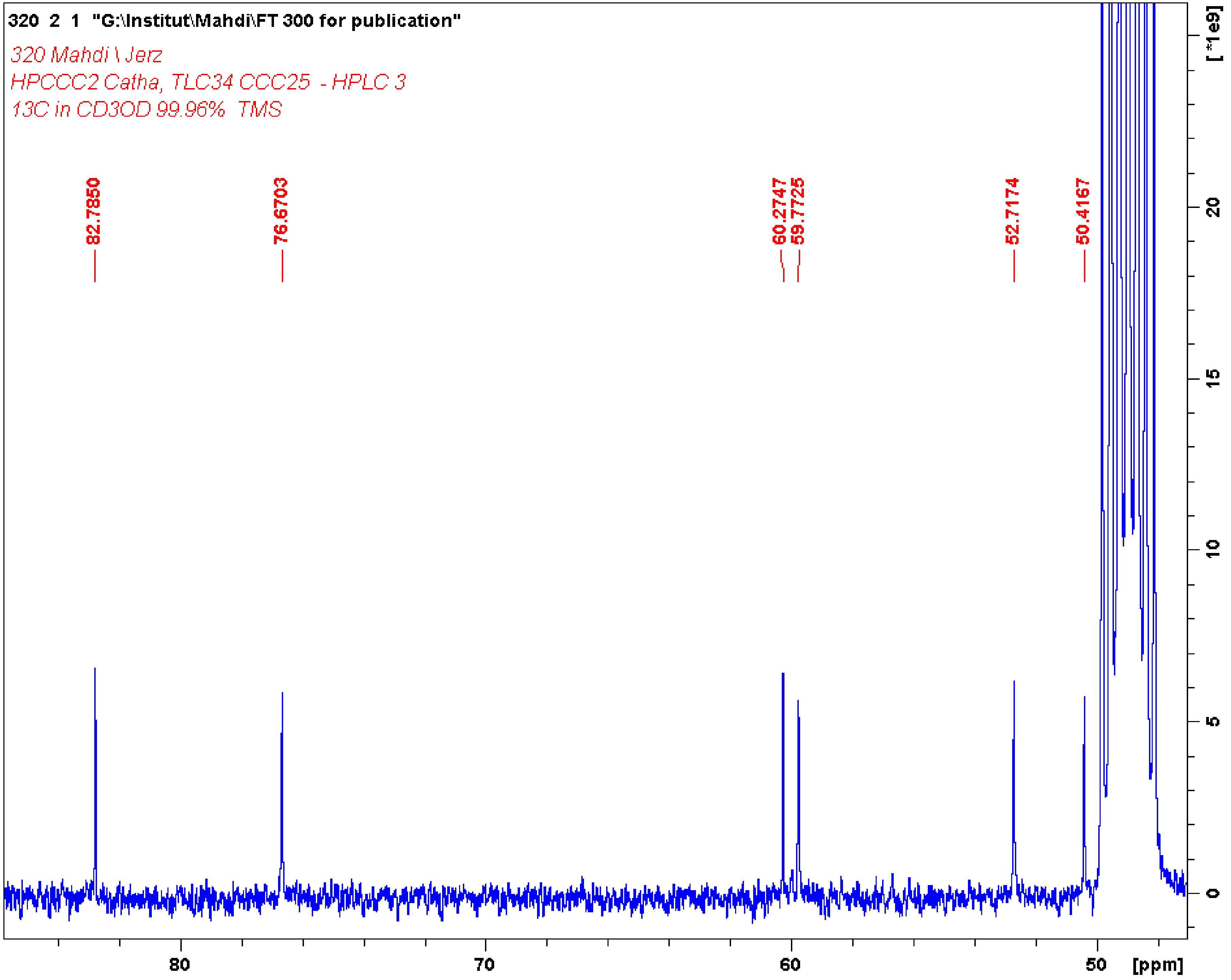

Figure NMR-S7

^{13}C NMR – Vindolinine (*337-b*)
in CD_3OD
(300 MHz)

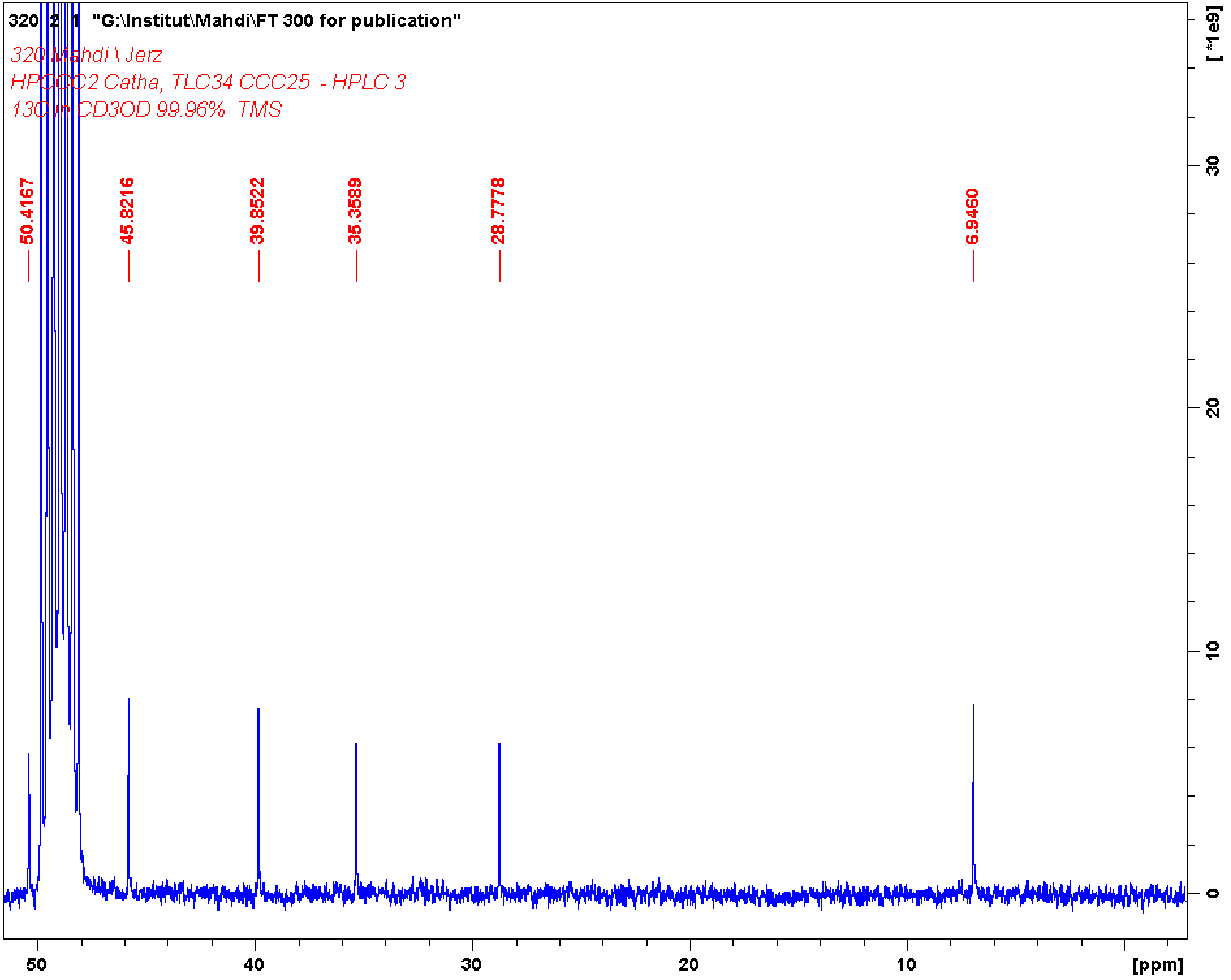

Figure NMR-S7

DEPT 135 NMR
Vindolinine (*337-b*)
in CD_3OD
(300 MHz)

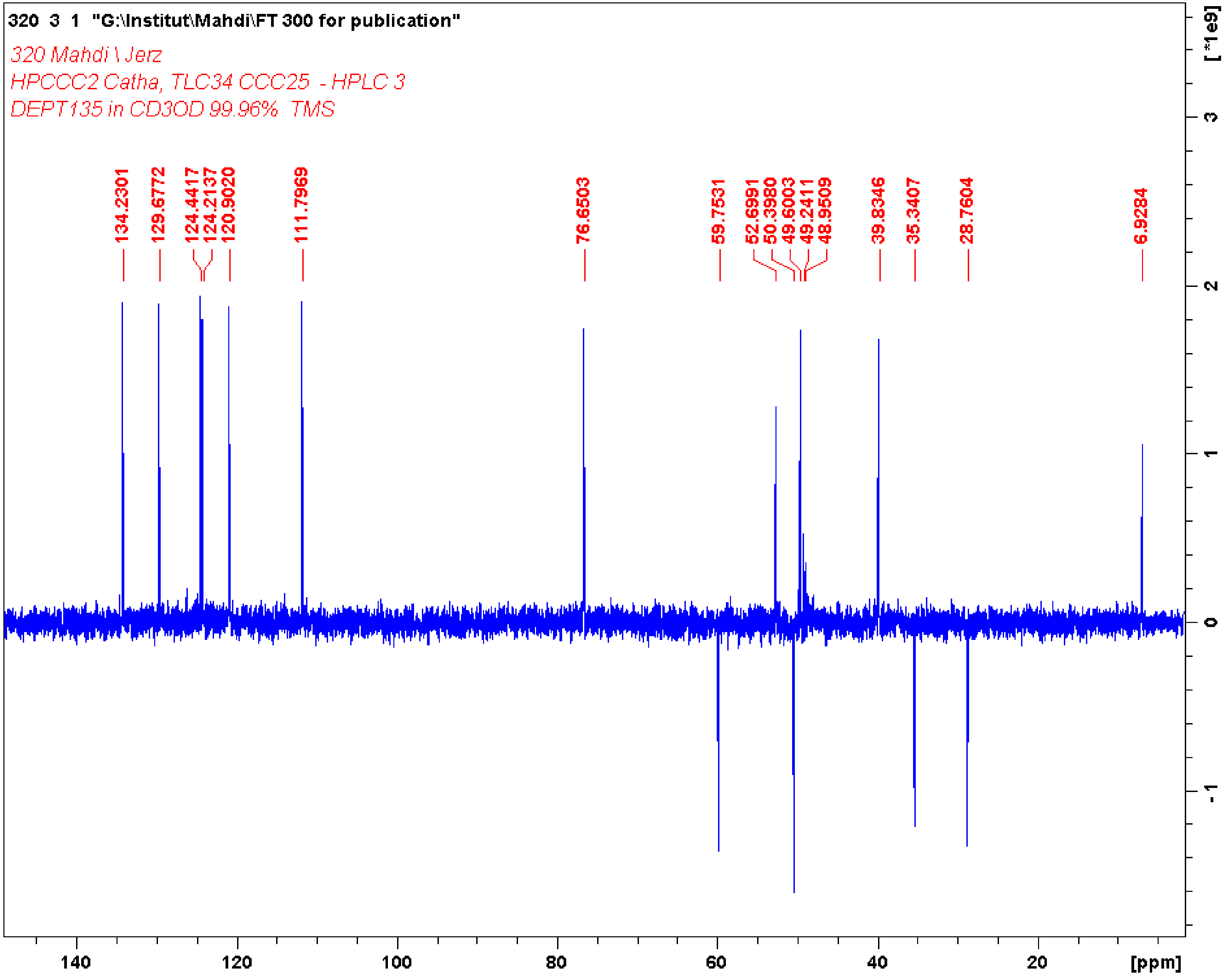

Figure NMR-S7

DEPT 135 NMR
Vindolinine (*337-b*)
in CD_3OD
(300 MHz)

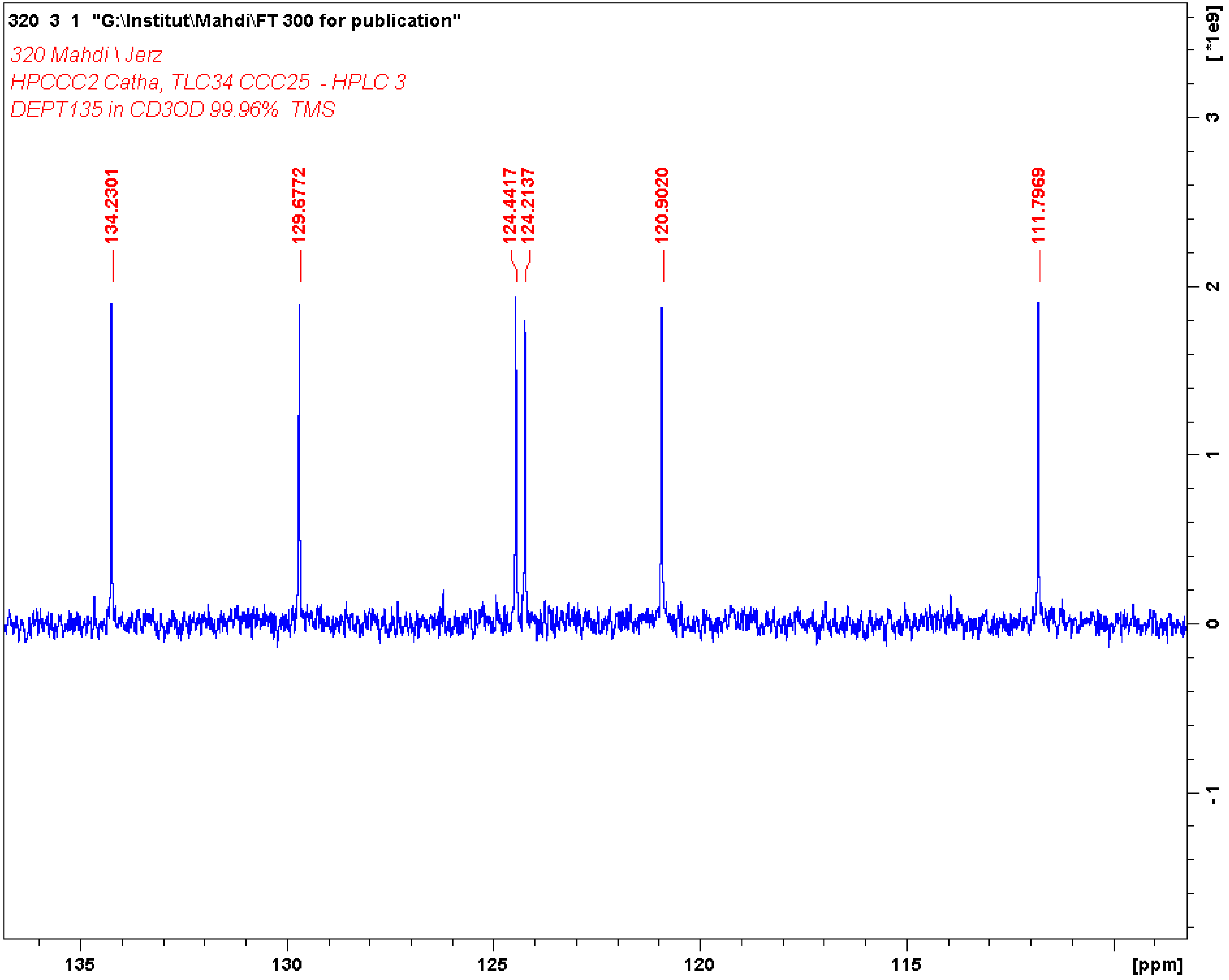

Figure NMR-S7

DEPT 135 NMR
Vindolinine (*337-b*)
in CD_3OD
(300 MHz)

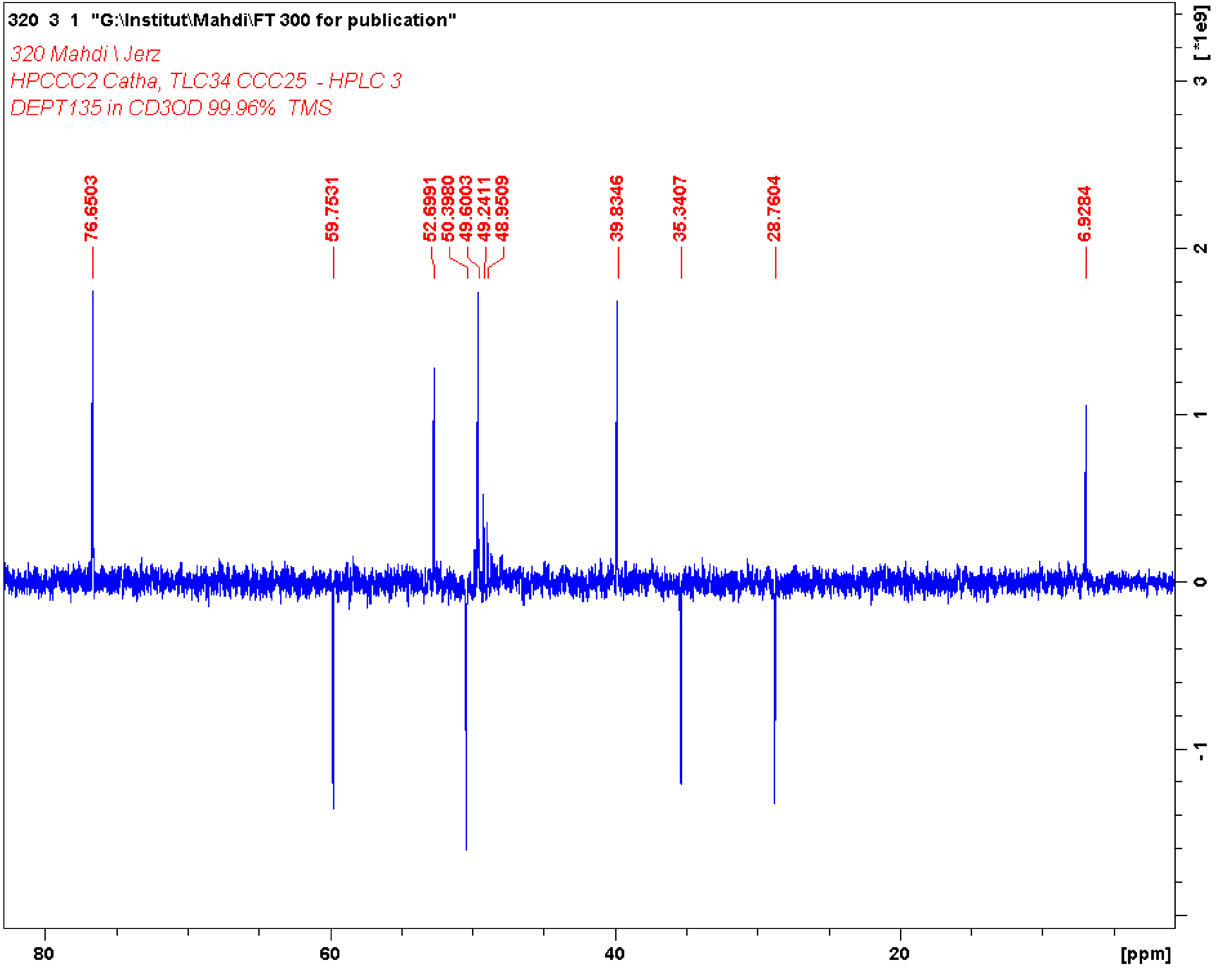

Figure NMR-S7
HSQC phase edited ^{1}J-HC

Vindolinine (*337-b*)
in CD_3OD
(300 MHz)

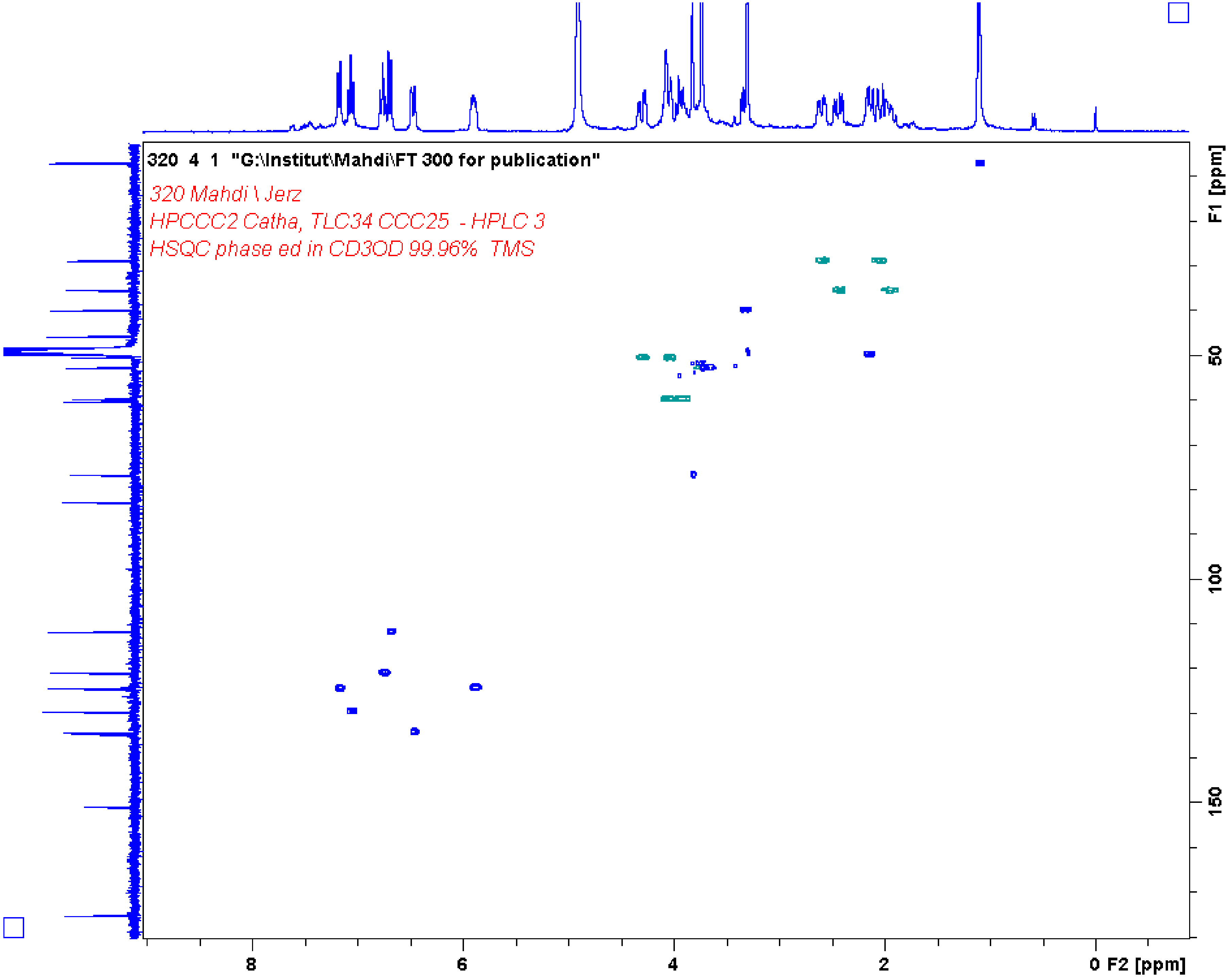

Figure NMR-S7

HSQC phase edited ^{1}J-*HC*

Vindolinine (*337-b*)
in CD_3OD
(300 MHz)

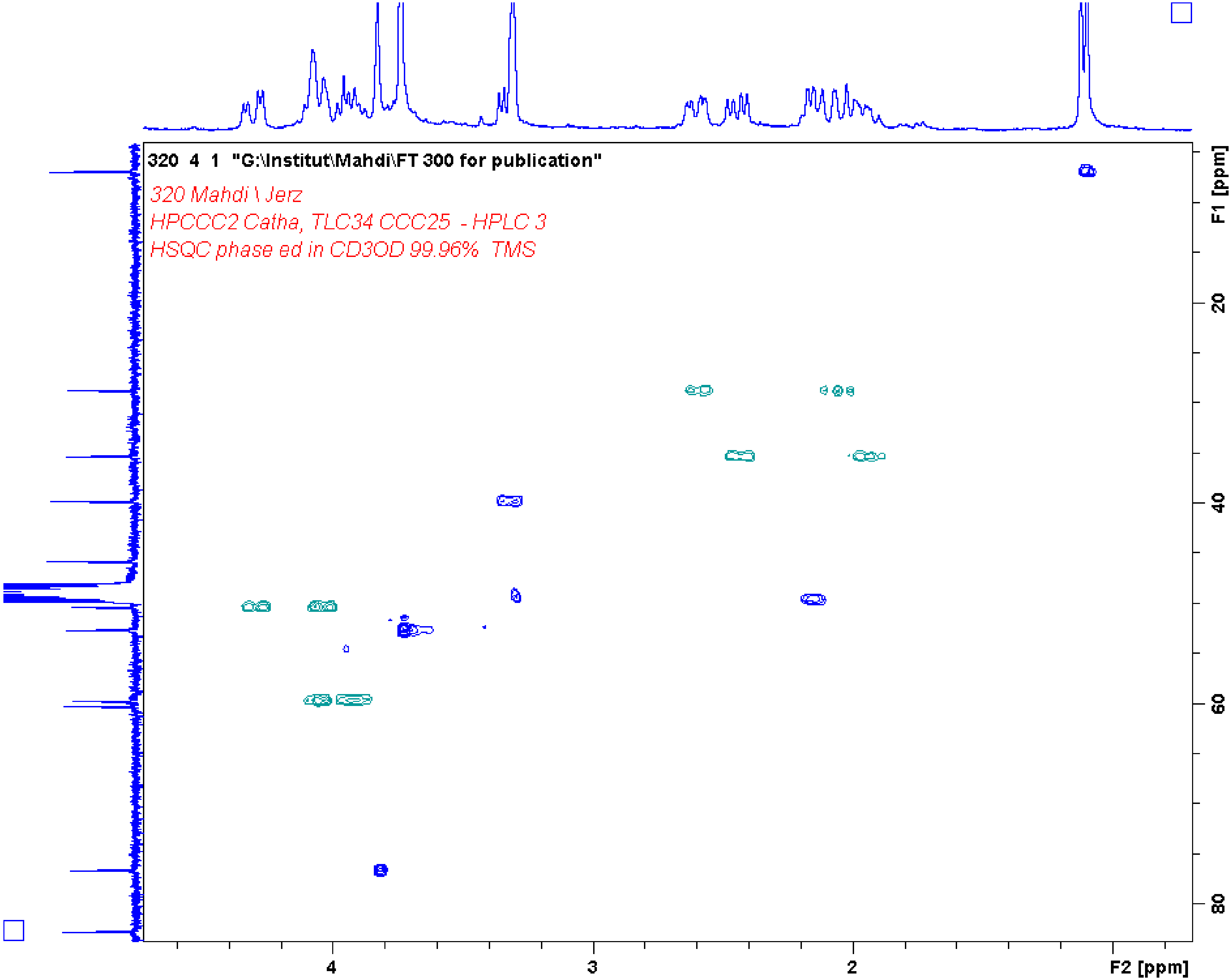

Figure NMR-S7
HSQC phase edited ^{1}J-HC

Vindolinine (*337-b*)
in CD_3OD
(300 MHz)

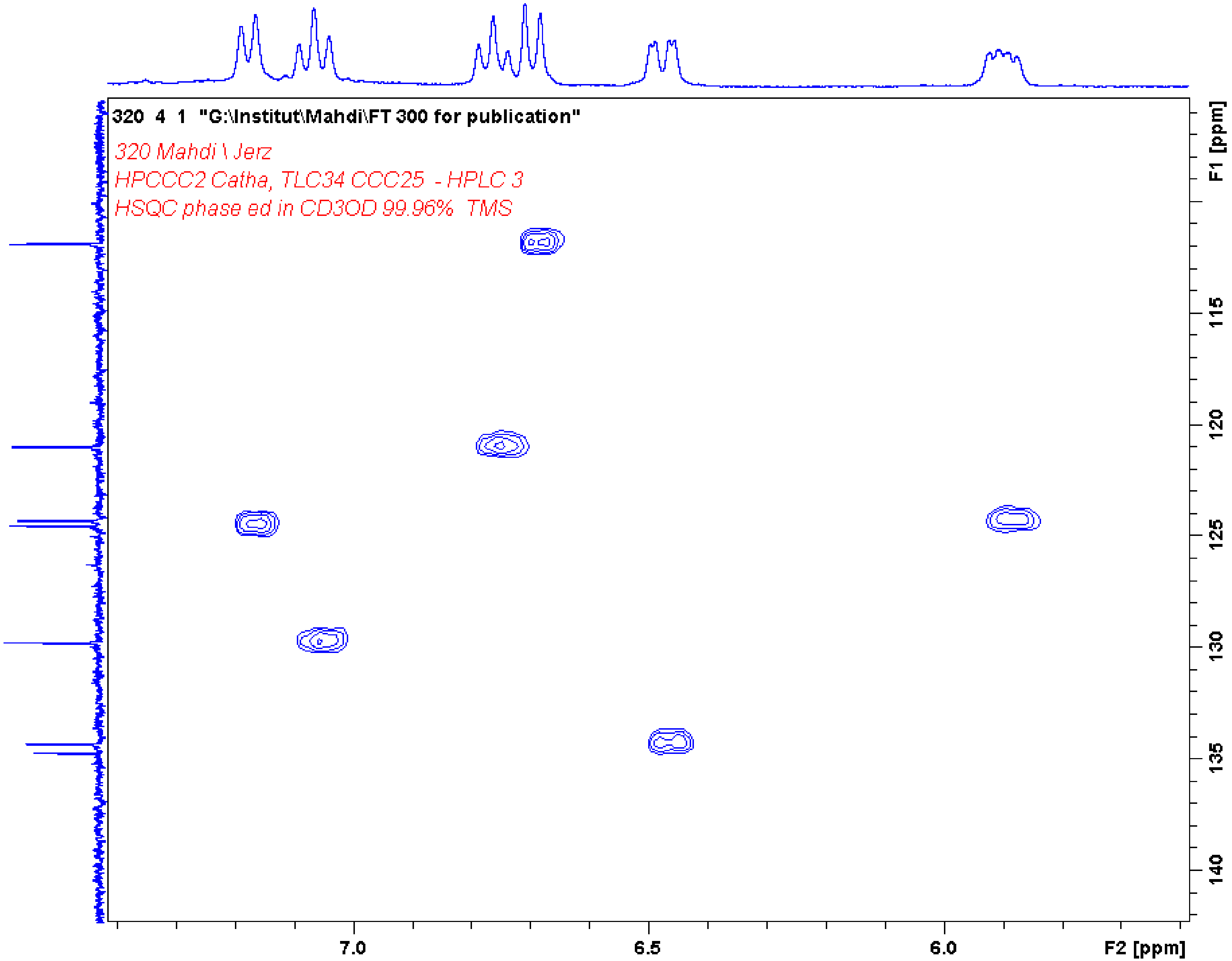

Figure NMR-S7

HMBC, long-range $^{2,3}J$-HC

Vindolinine (*337-b*)
in CD_3OD
(300 MHz)

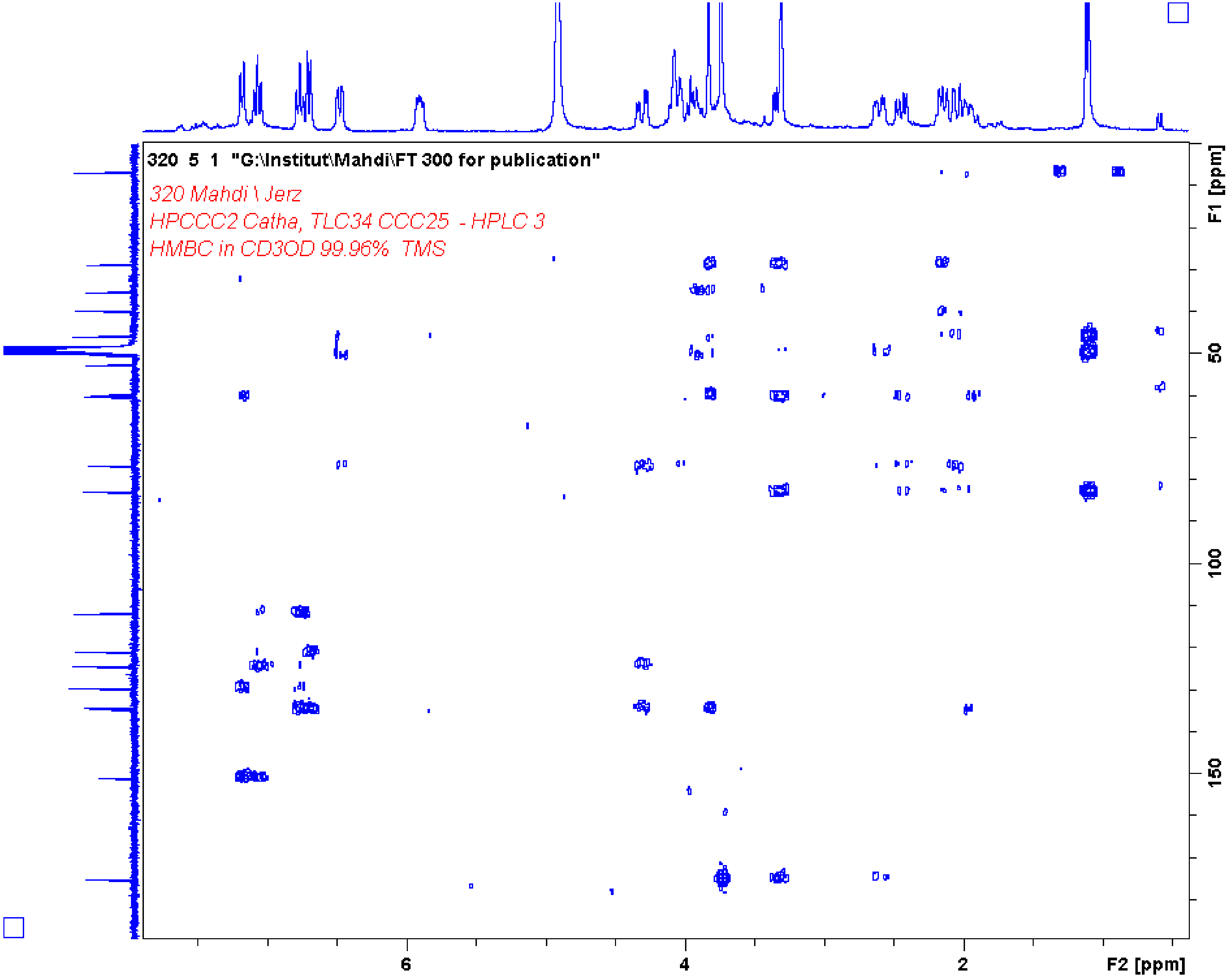

Figure NMR-S7

HMBC, long-range $^{2,3}J$-HC

Vindolinine (*337-b*)
in CD_3OD
(300 MHz)

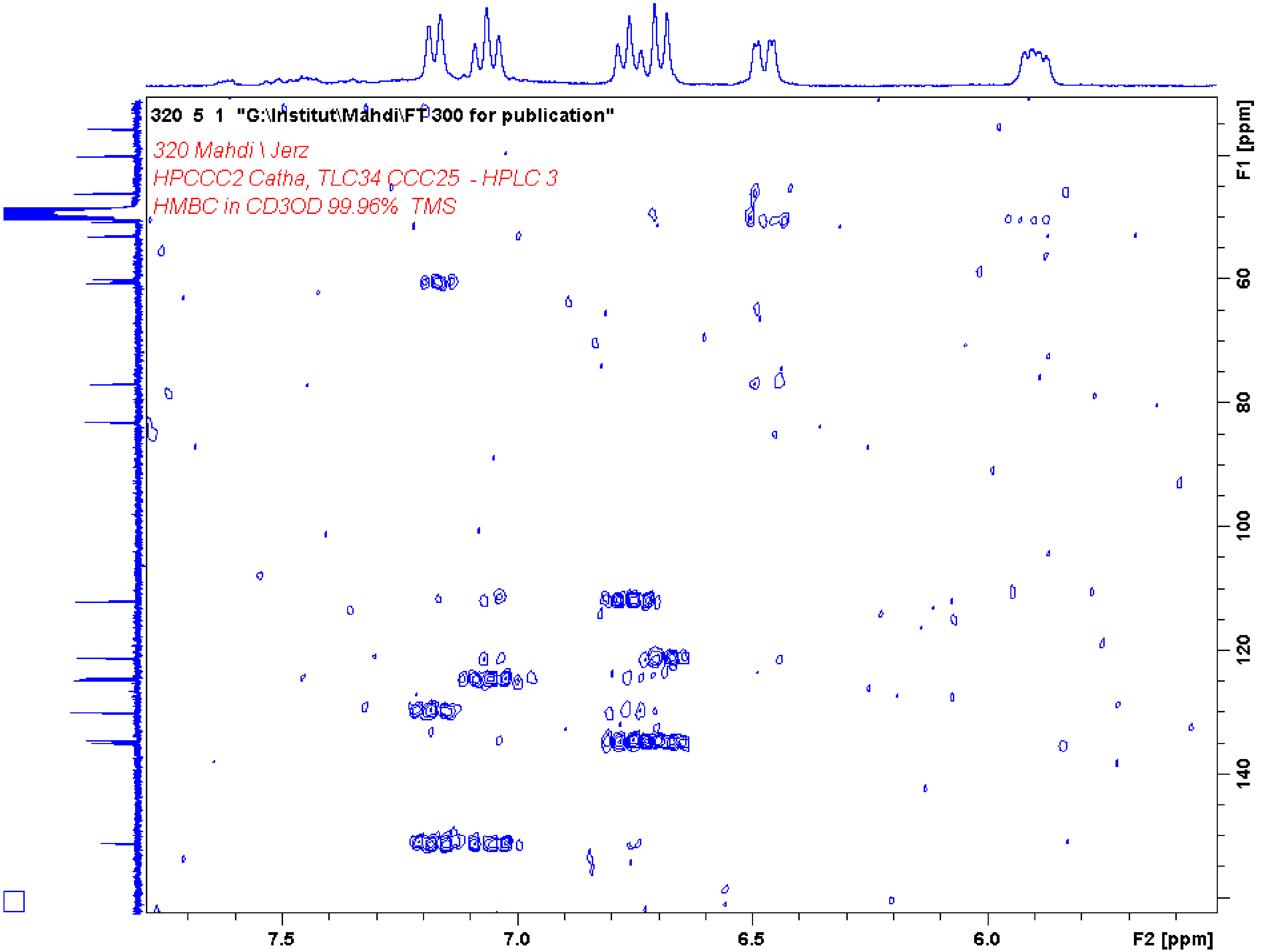

Figure NMR-S7

HMBC, long-range $^{2,3}J$-HC

Vindolinine (*337-b*)
in CD_3OD
(300 MHz)

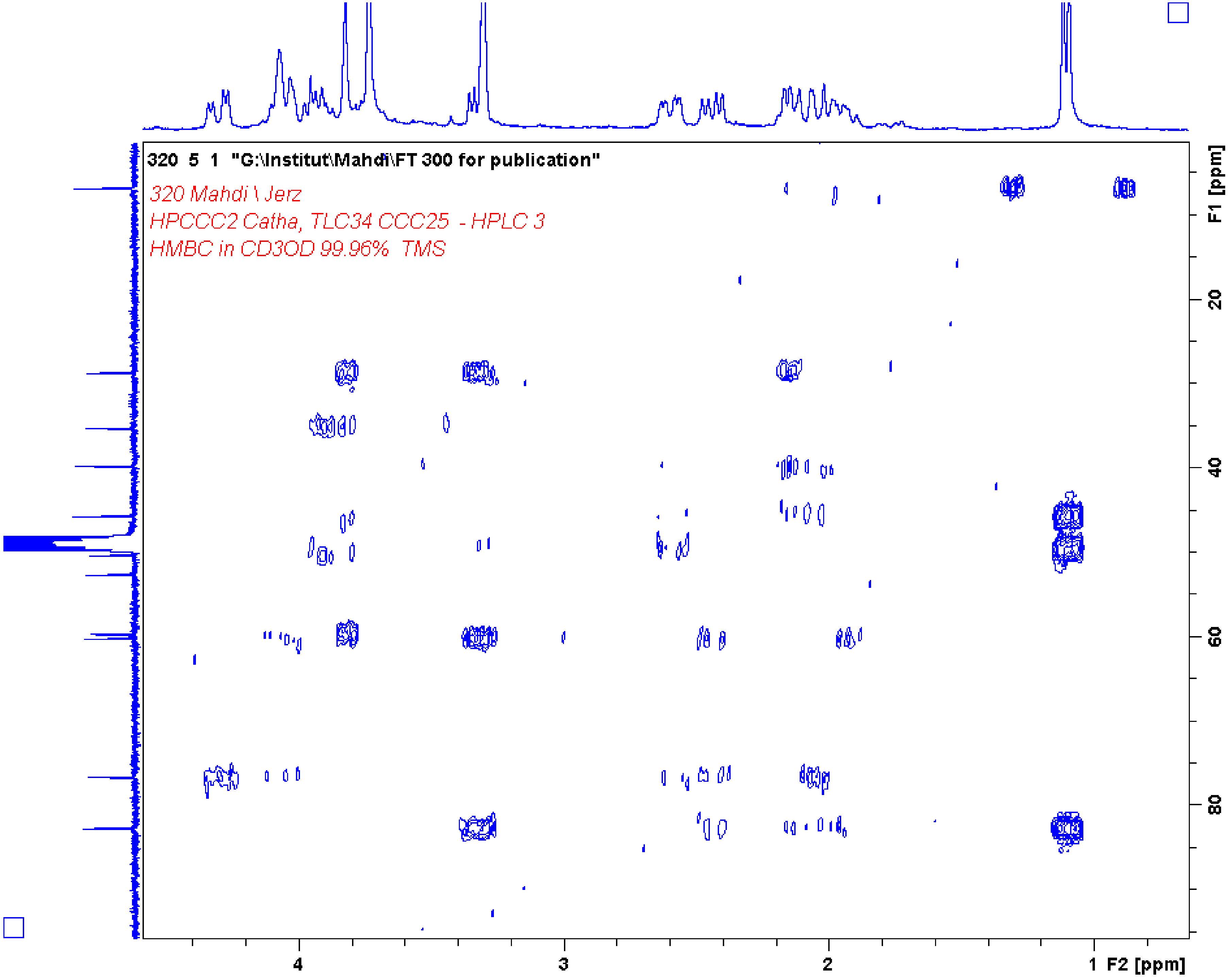

Figure NMR-S7

HMBC, long-range $^{2,3}J$-HC

Vindolinine (*337-b*)
in CD_3OD
(300 MHz)

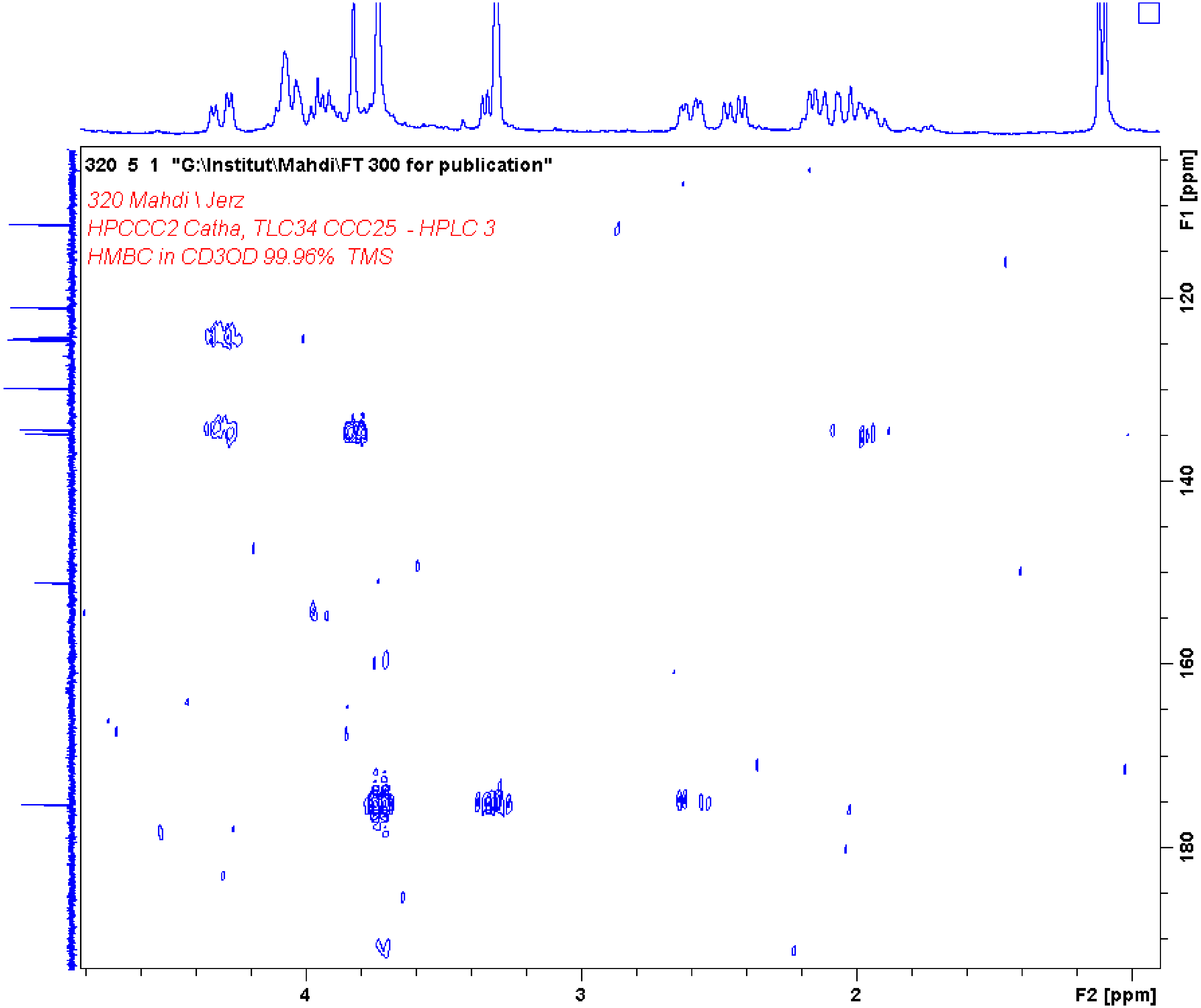

Figure NMR-S7

$^{1}H/^{1}H$-NOESY

Vindolinine (*337-b*)
in CD_3OD
(300 MHz)

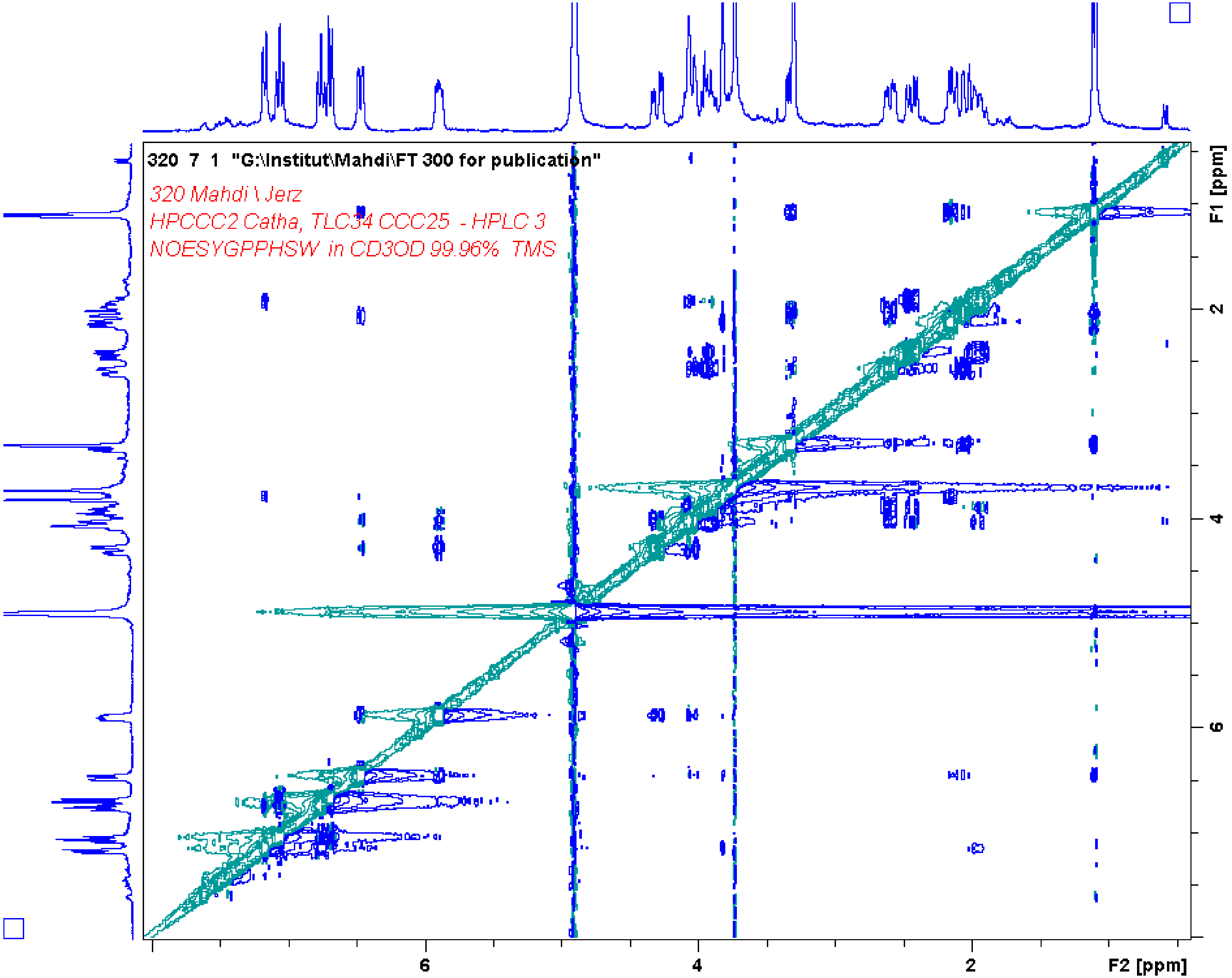

Figure NMR-S7

$^1H/^1H$-NOESY

Vindolinine (*337-b*)
in CD_3OD
(300 MHz)

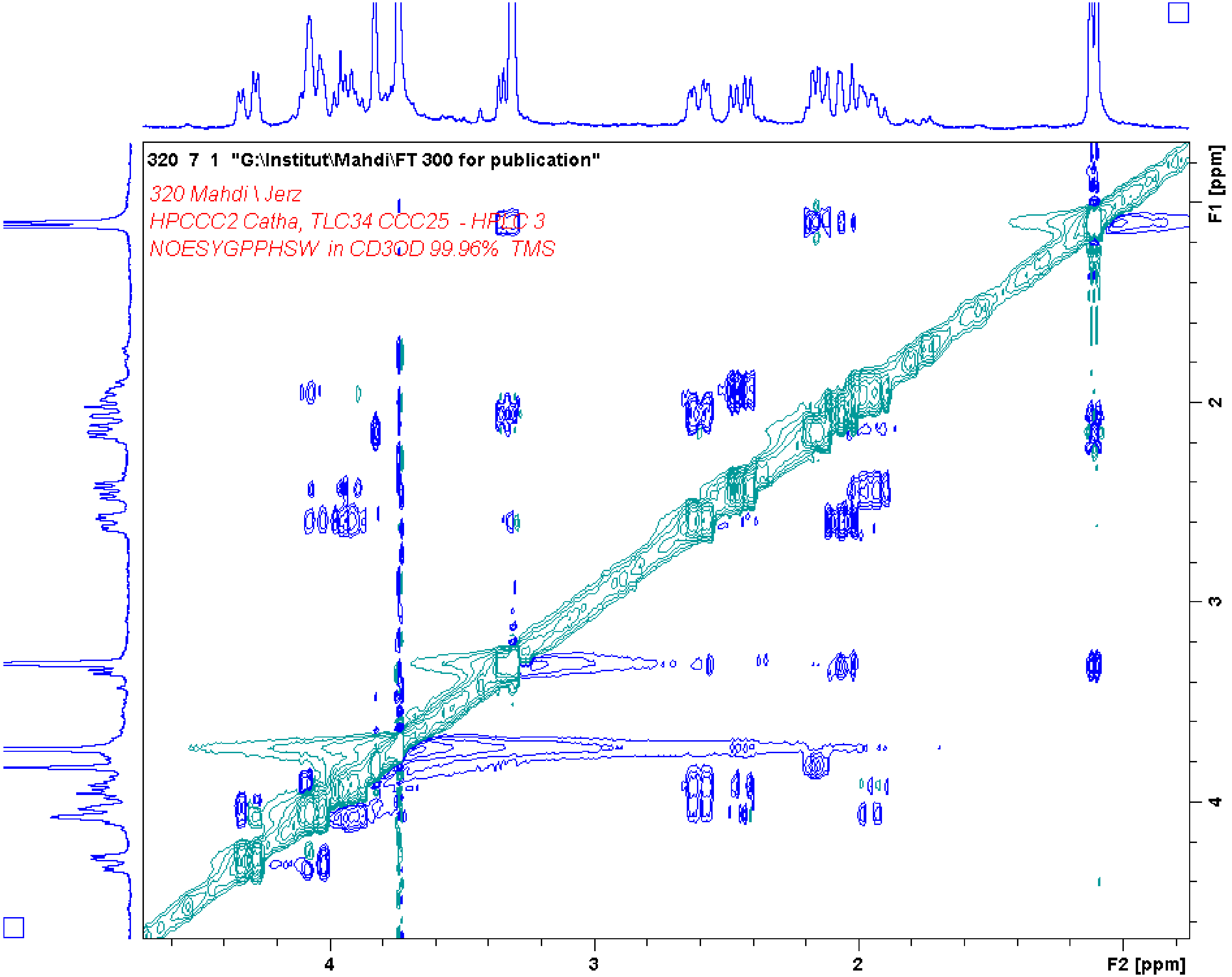

Figure NMR-S7

$^{1}H/^{1}H$-NOESY

Vindolinine (*337-b*)
in CD_3OD
(300 MHz)

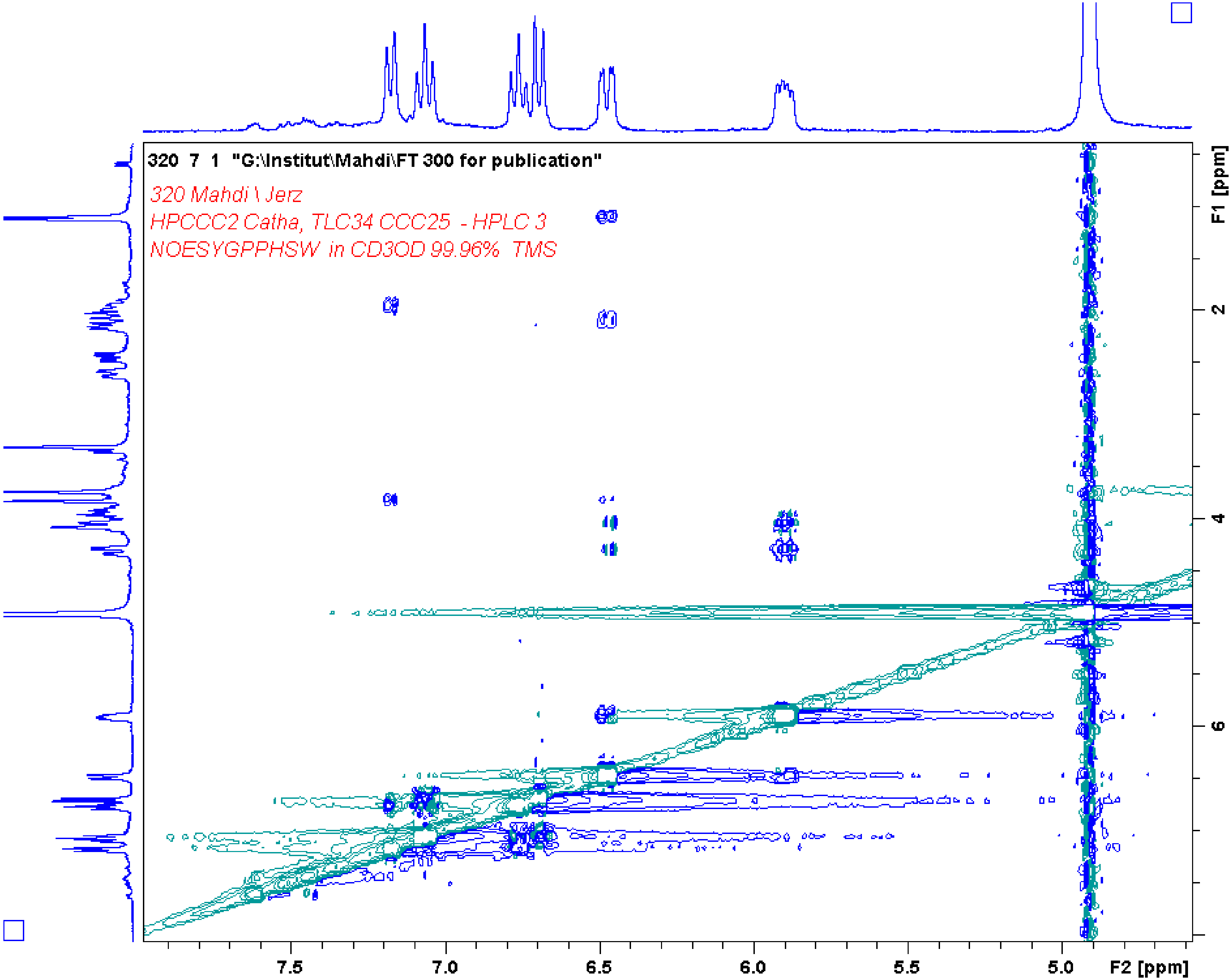

Figure NMR-S8

^{1}H NMR – Vindolinine (*337-b*) in $CDCl_3$ (300 MHz)

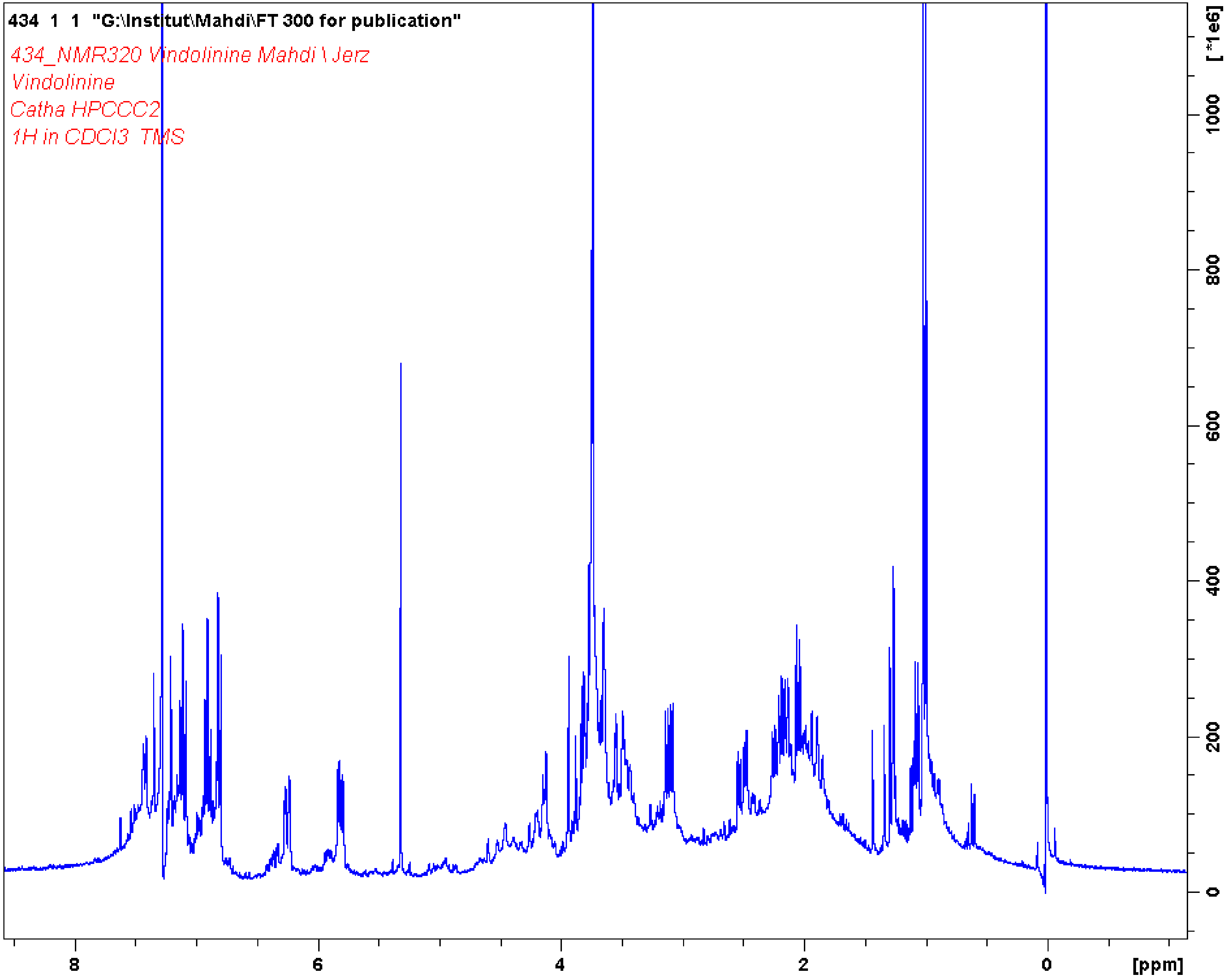

Figure NMR-S8

^{13}C NMR – Vindolinine (*337-b*)
in $CDCl_3$
(300 MHz)

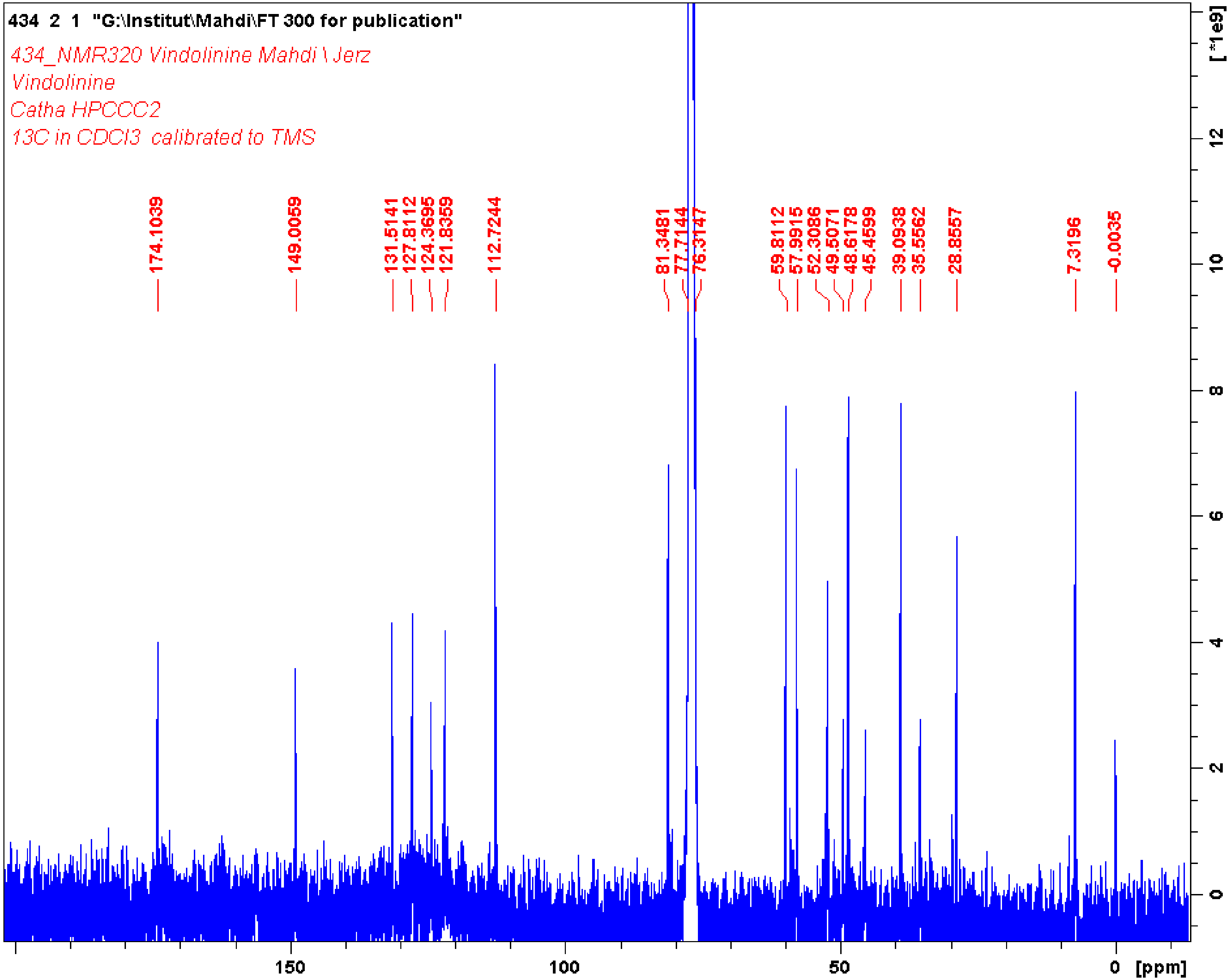

Figure NMR-S8

^{13}C NMR – Vindolinine (*337-b*) in $CDCl_3$ (300 MHz)

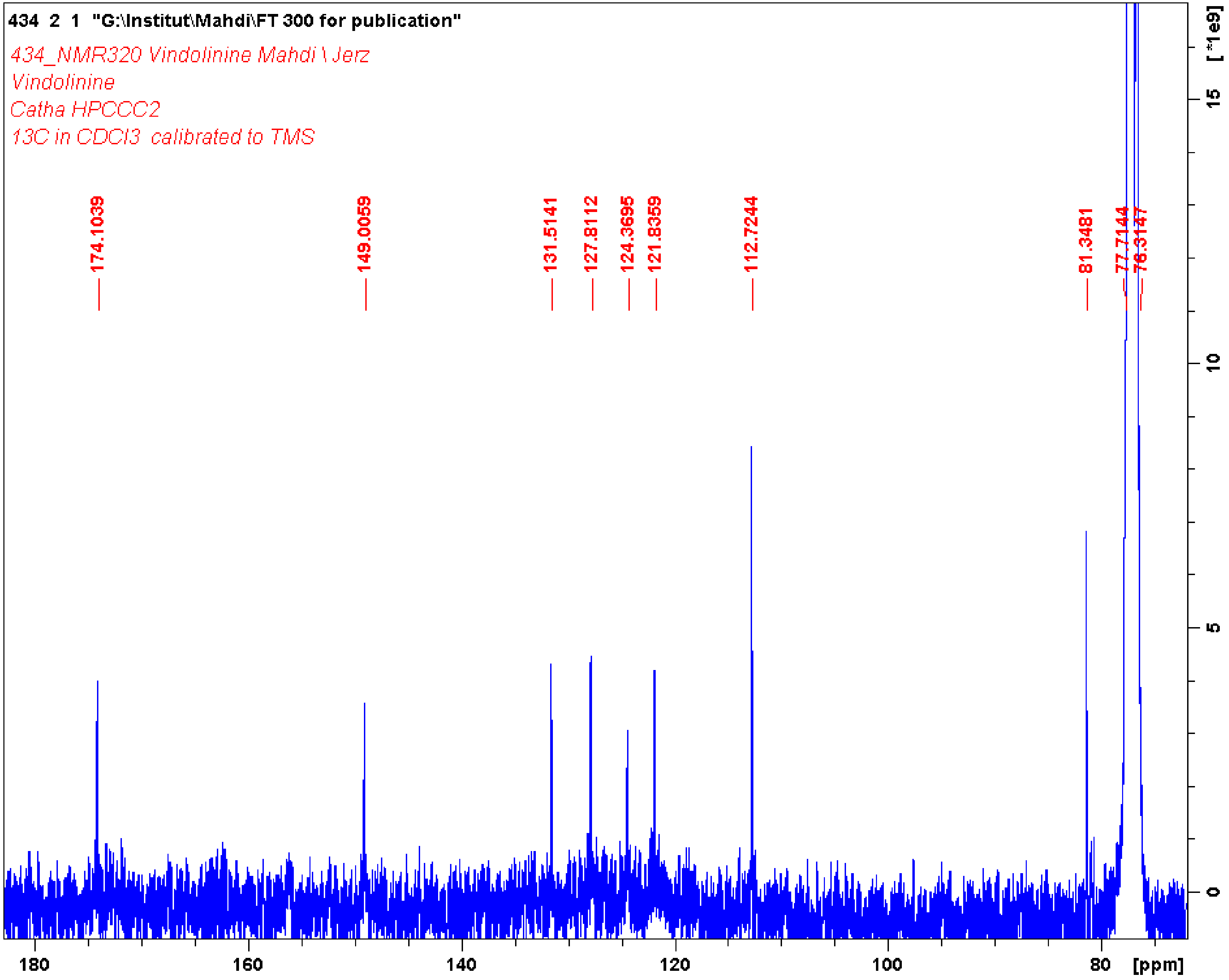

Figure NMR-S8

^{13}C NMR – Vindolinine (*337-b*)
in $CDCl_3$
(300 MHz)

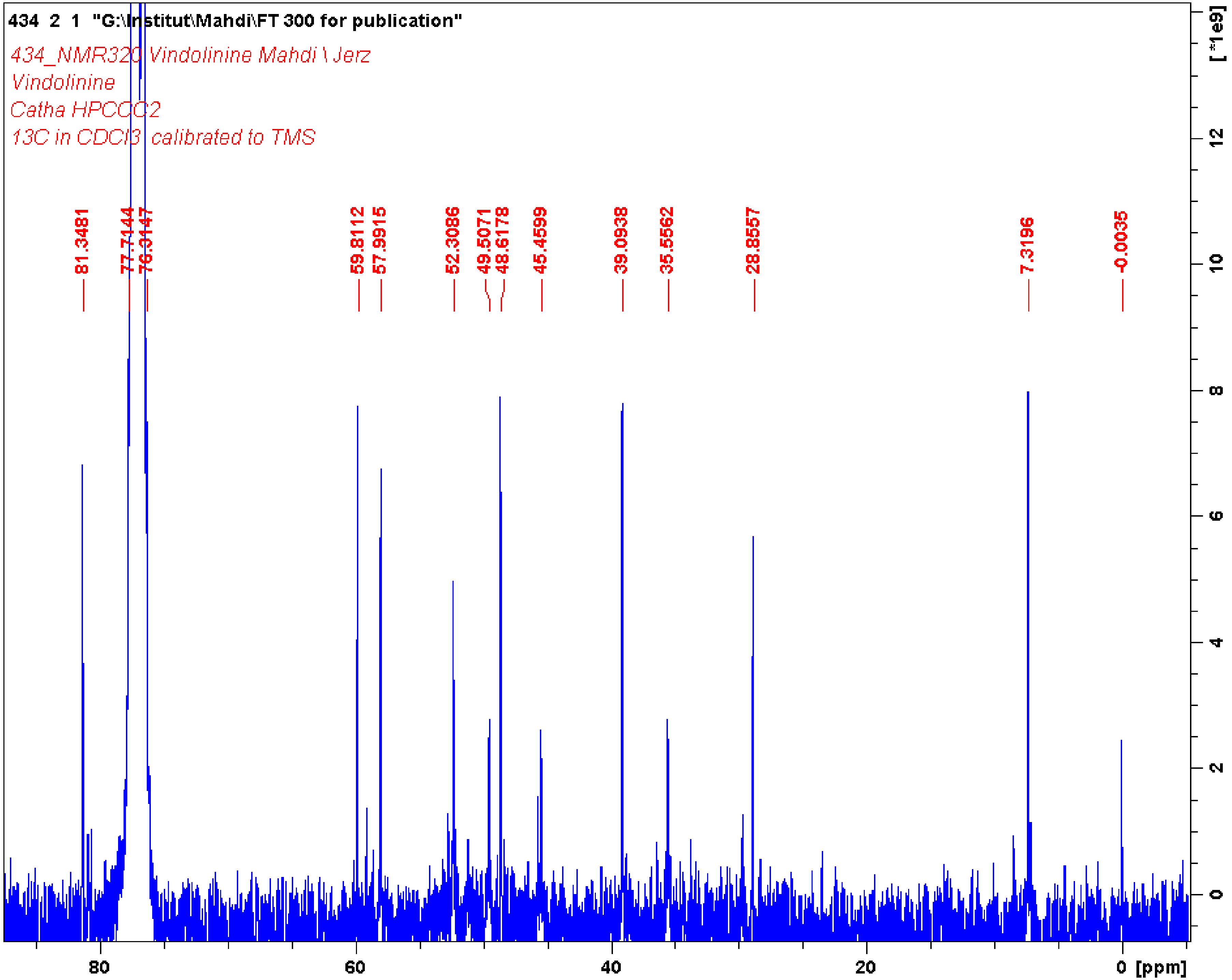

Figure NMR-S9

^{1}H NMR – Vindoline (*457*) in CD_3OD (300 MHz)

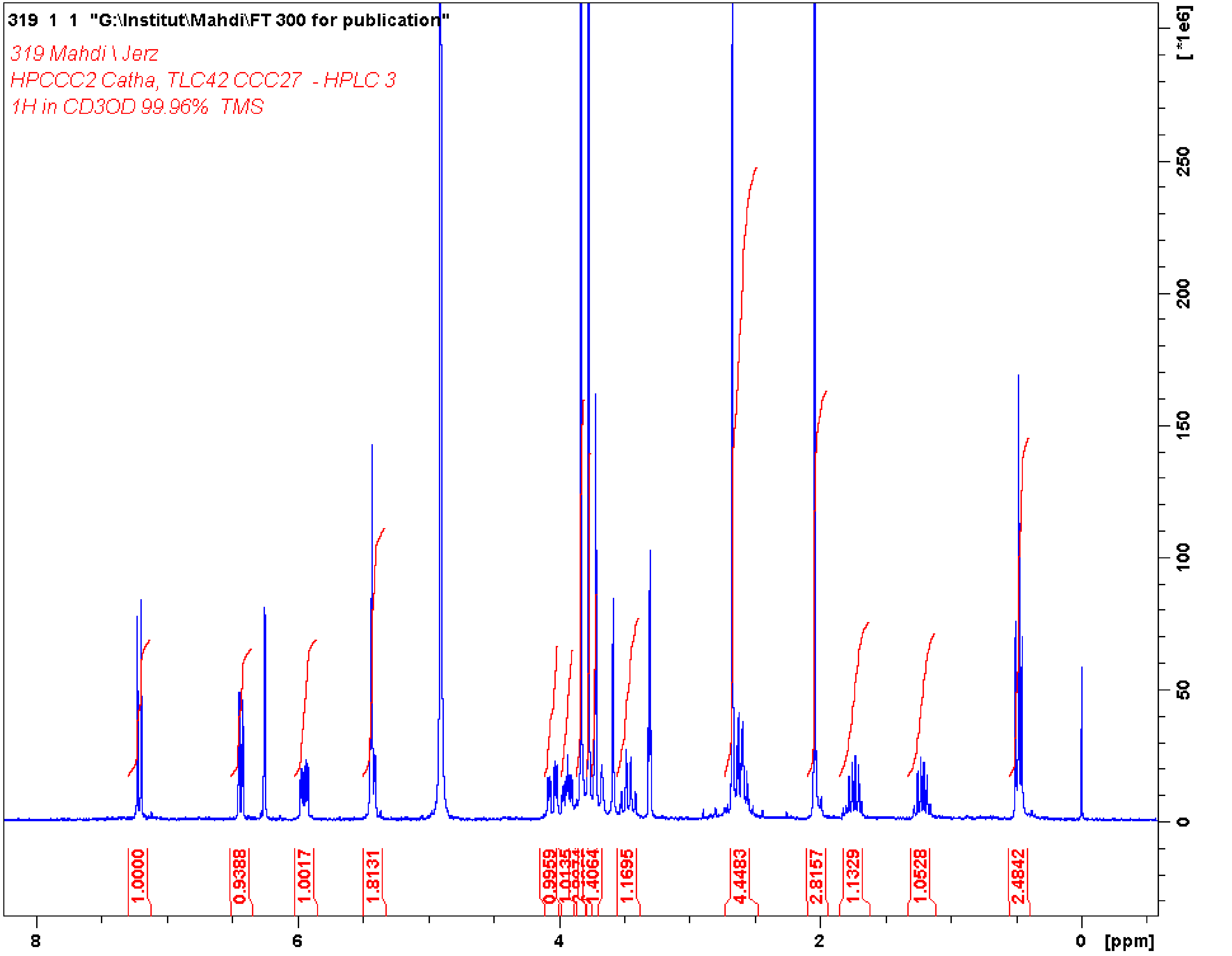

Figure NMR-S9

^{1}H NMR – Vindoline (*457*)
in CD_3OD
(300 MHz)

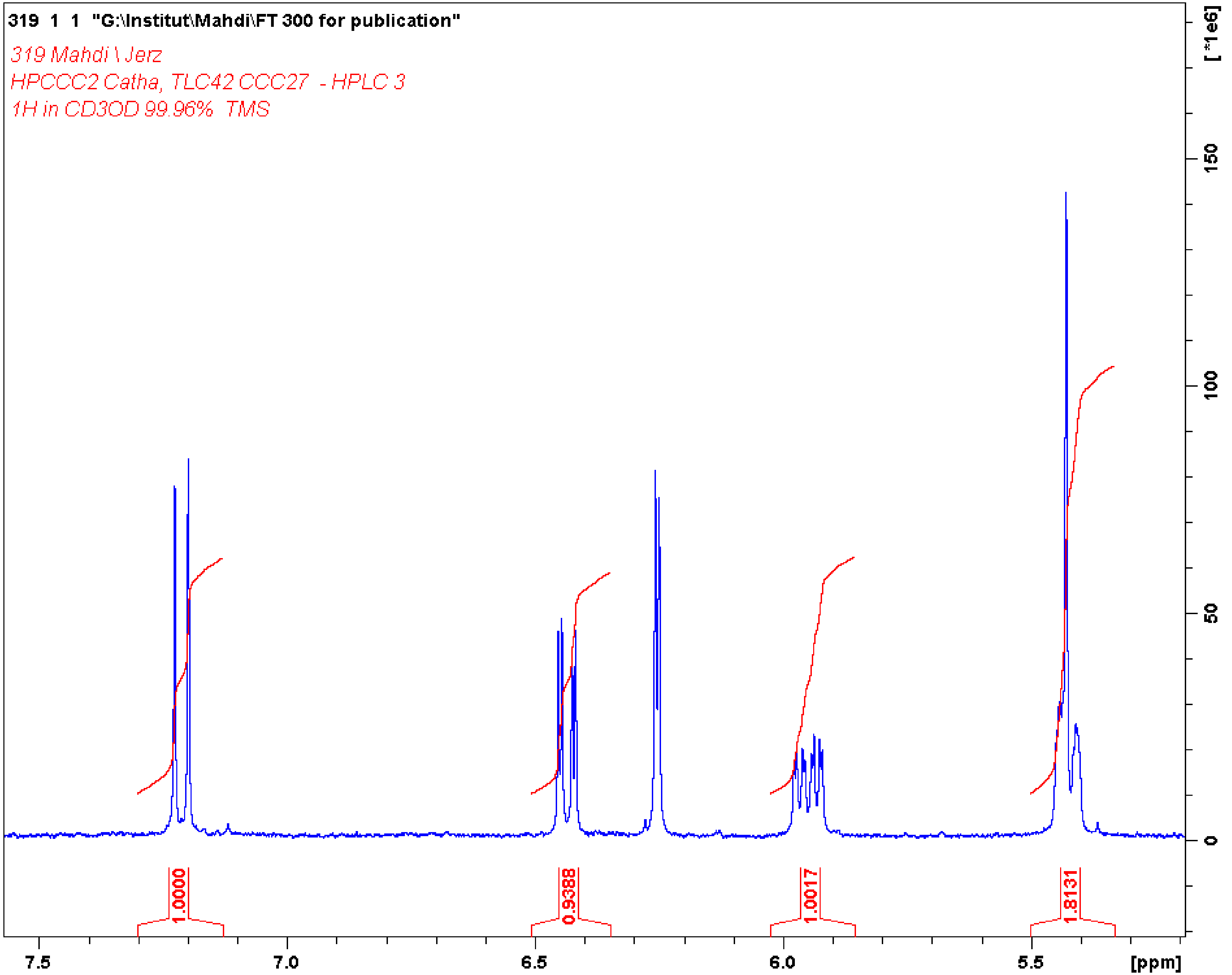

<u>**Figure NMR-S9**</u>

^{1}H NMR – Vindoline (*457*)

in CD_3OD
(300 MHz)

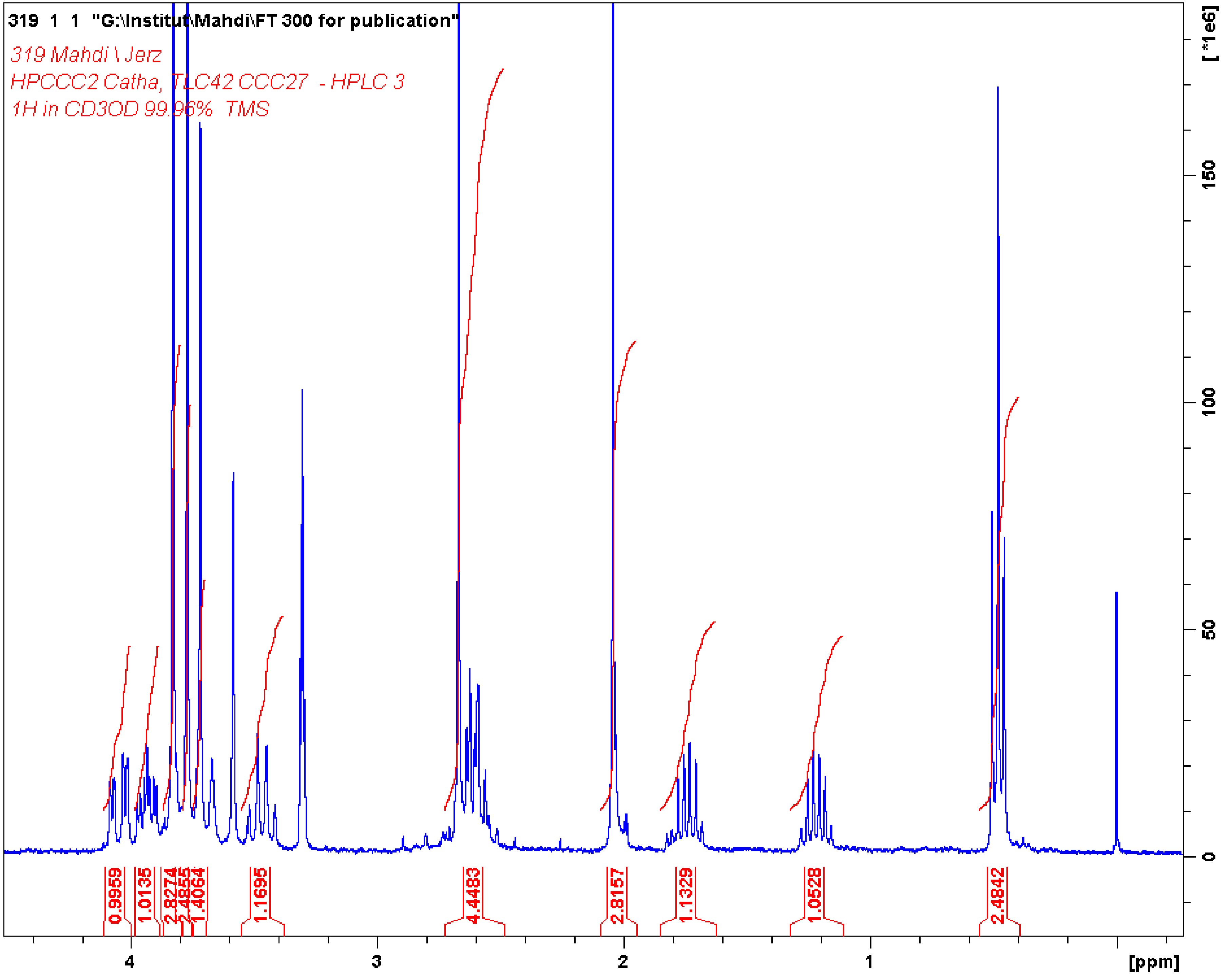

Figure NMR-S9

^{13}C NMR – Vindoline (*457*)

in CD_3OD
(300 MHz)

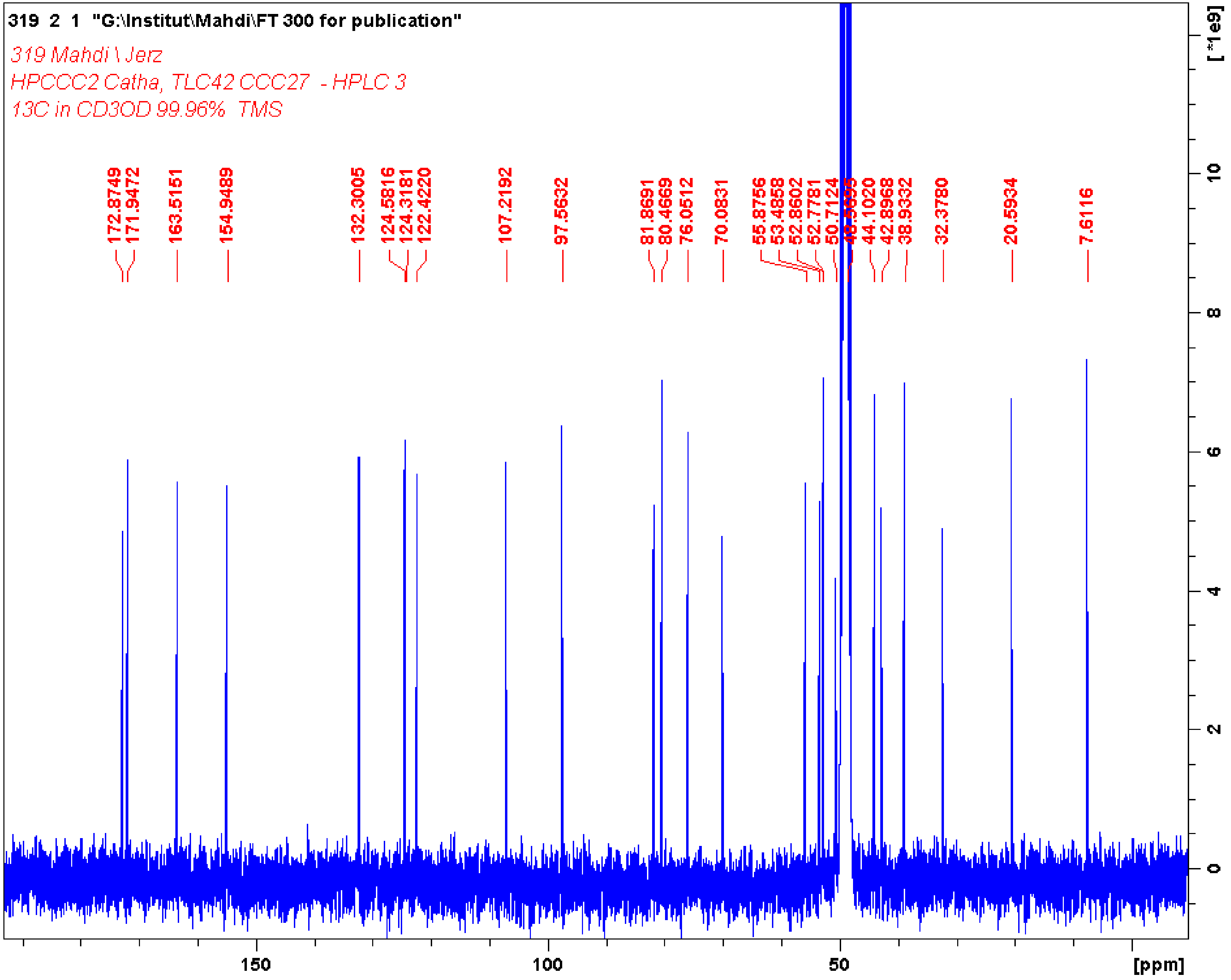

Figure NMR-S9

^{13}C NMR – Vindoline (*457*)

in CD_3OD
(300 MHz)

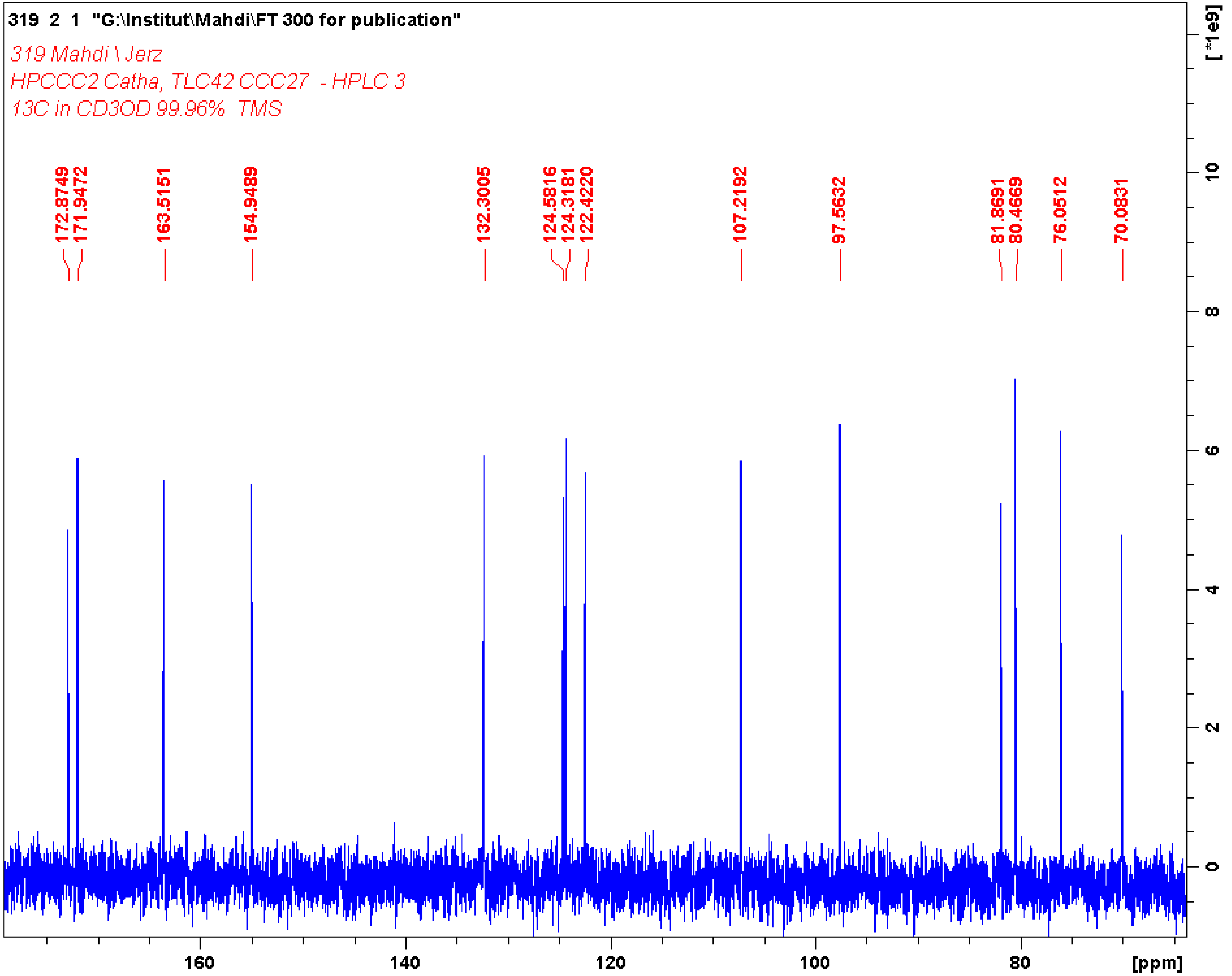

Figure NMR-S9

^{13}C NMR – Vindoline (*457*) in CD_3OD (300 MHz)

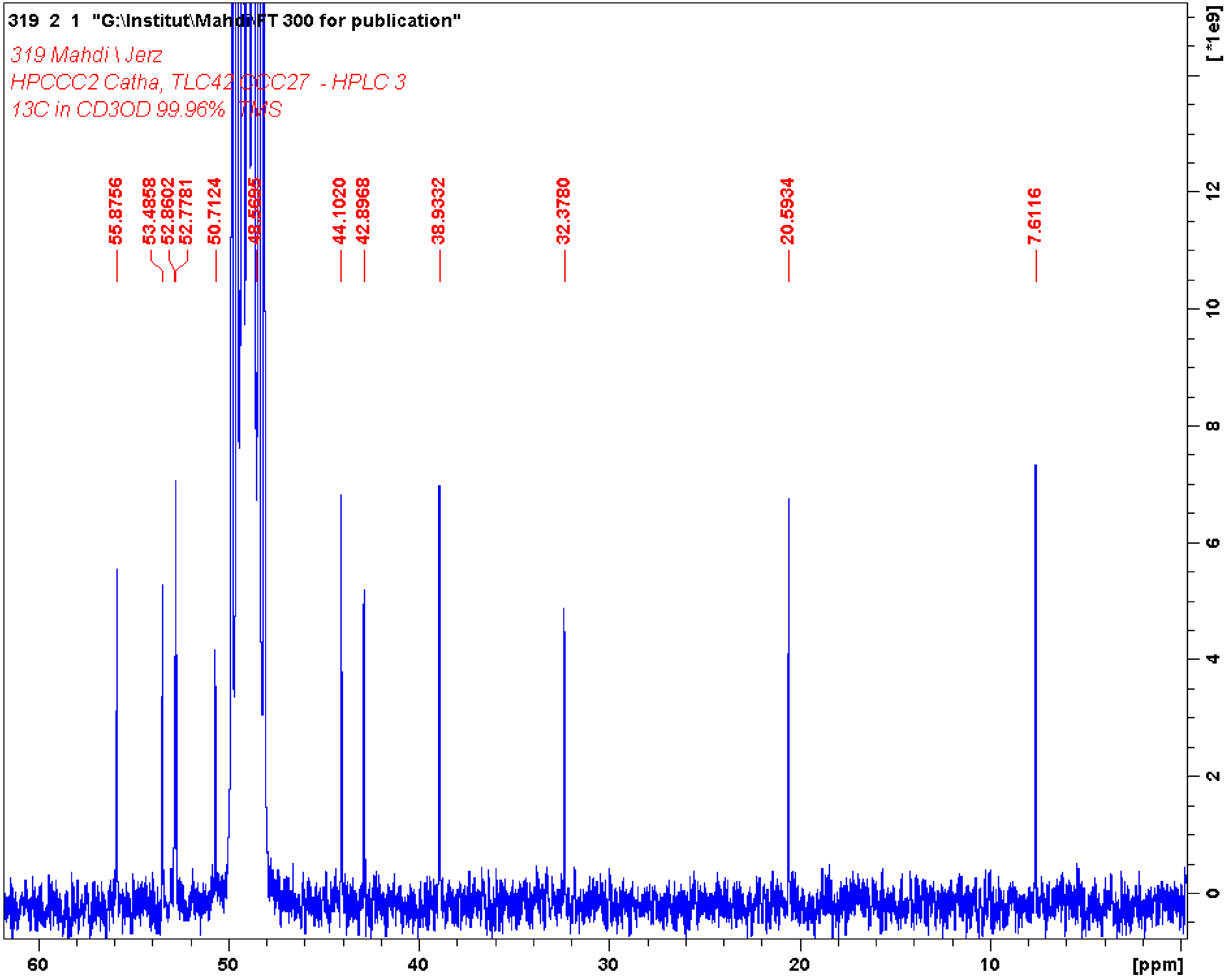

Figure NMR-S9

DEPT 135 NMR

Vindoline (*457*)
in CD_3OD
(300 MHz)

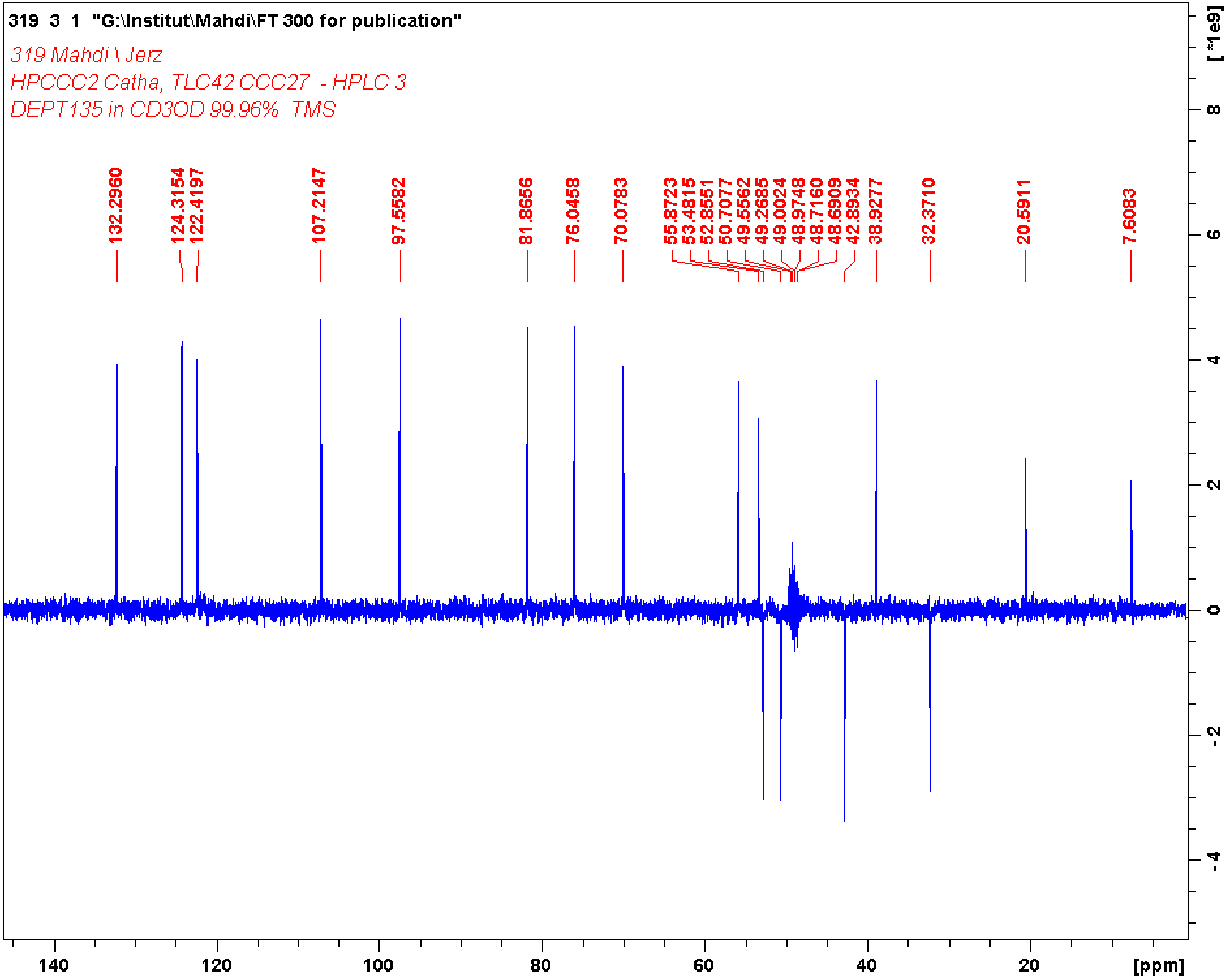

Figure NMR-S9

$^1H/^1H$-COSY
Vindoline (*457*)
in CD_3OD
(300 MHz)

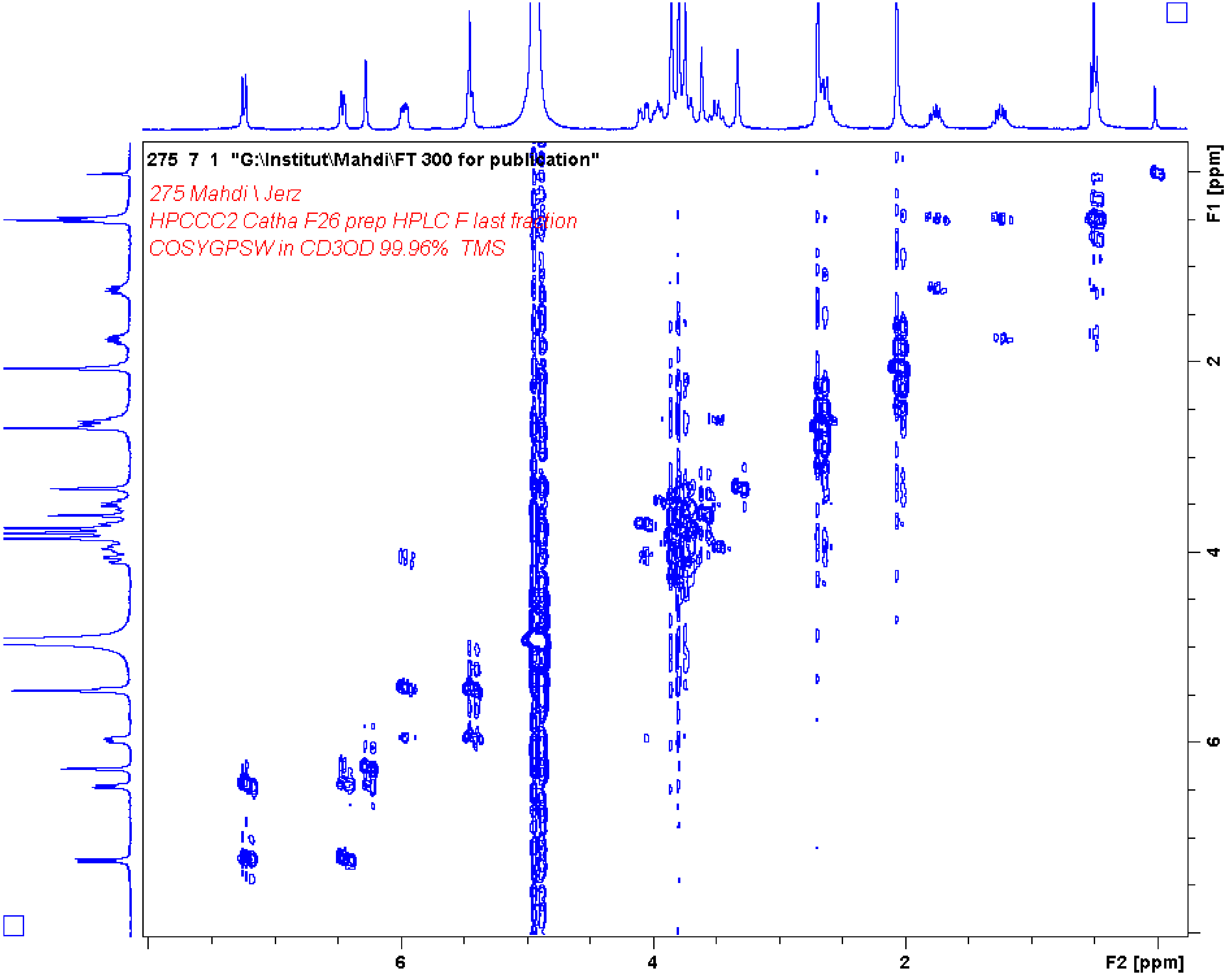

Figure NMR-S9

$^{1}H/^{1}H$-COSY
Vindoline (*457*)
in CD_3OD
(300 MHz)

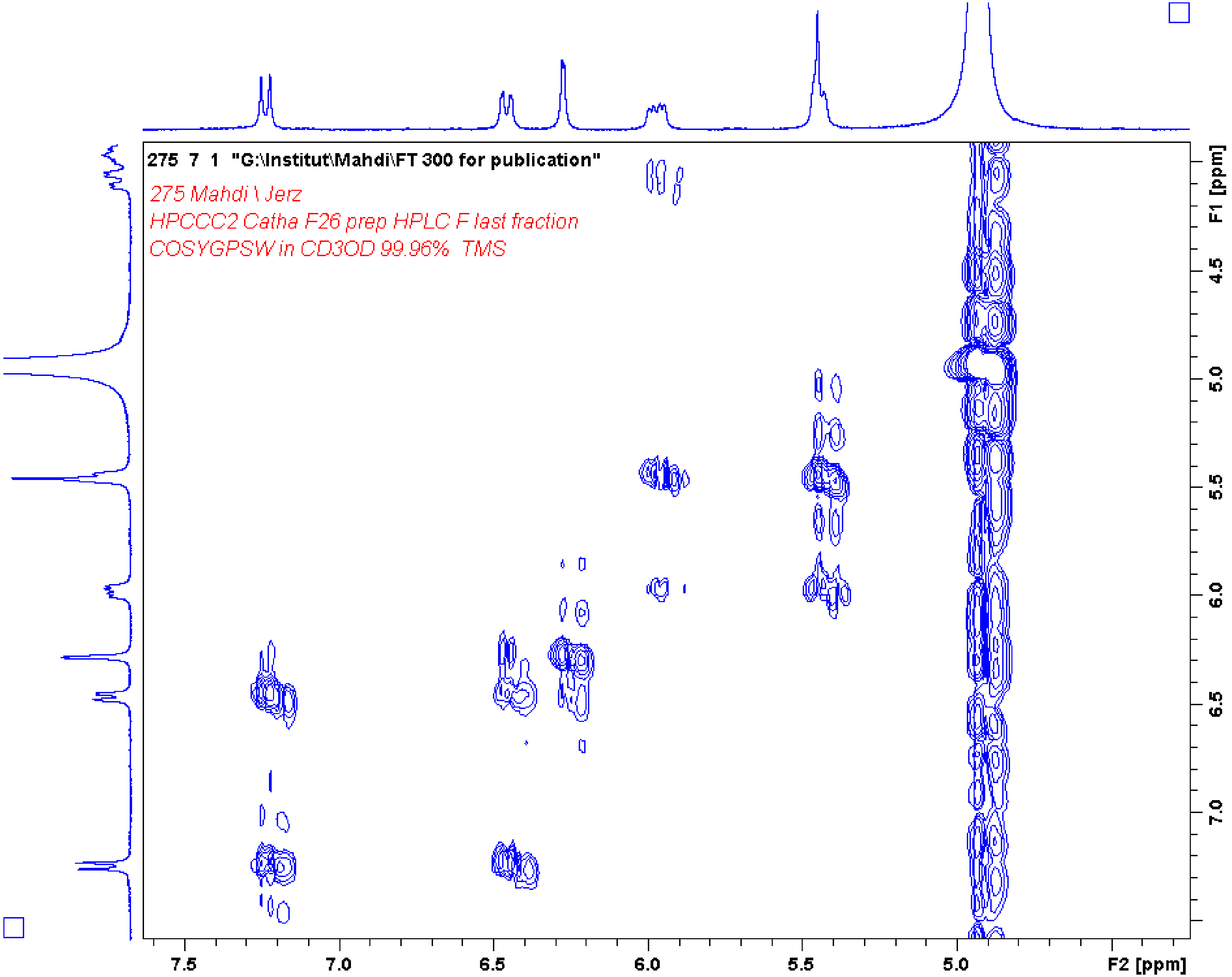

Figure NMR-S9

^{1}H/^{1}H-COSY
Vindoline (*457*)
in CD_3OD
(300 MHz)

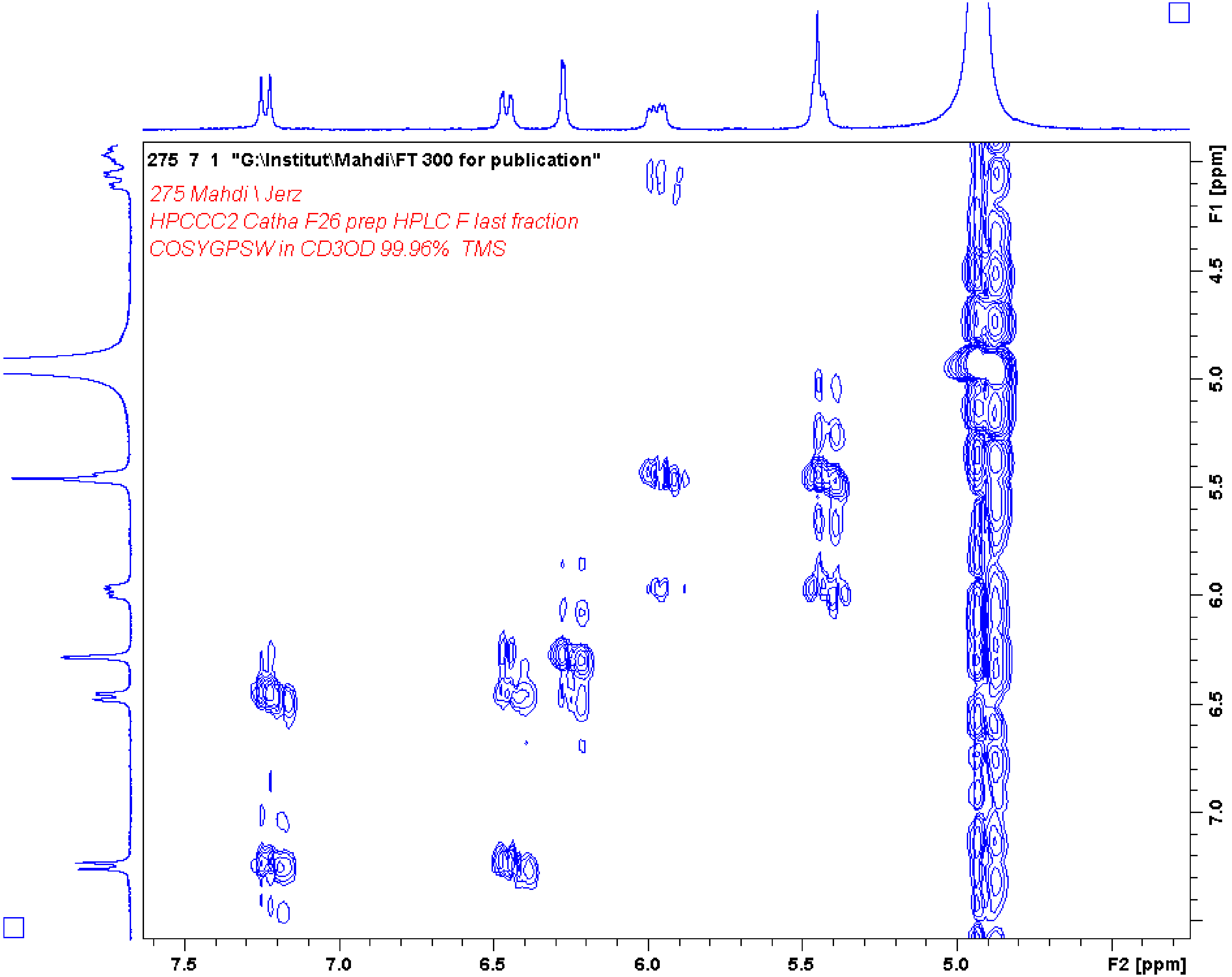

<u>**Figure NMR-S9**</u>

HSQC phase edited ^{1}J-HC

Vindoline (*457*)
in CD_3OD
(300 MHz)

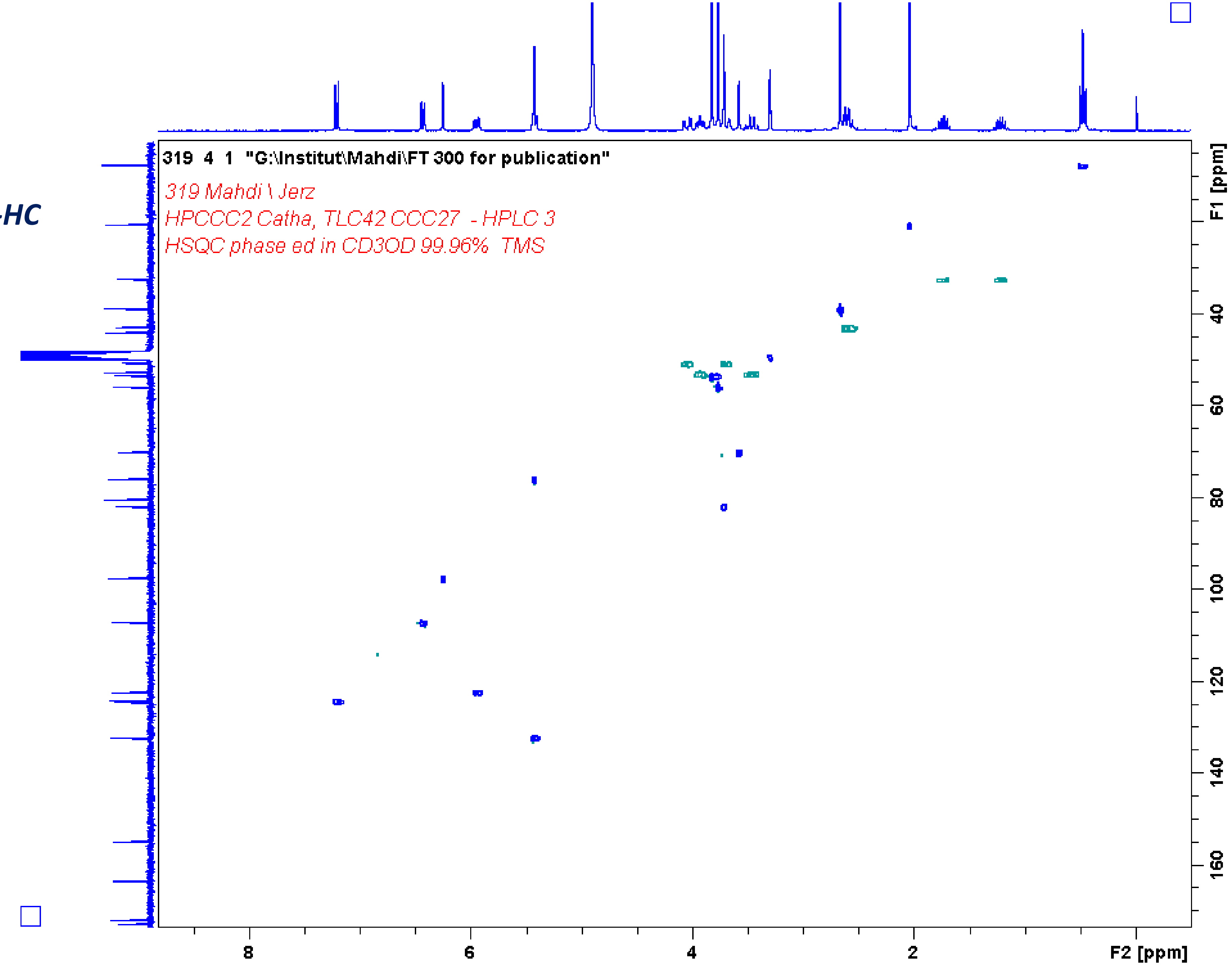

Figure NMR-S9

HSQC phase edited ^{1}J-HC

Vindoline (*457*)
in CD_3OD
(300 MHz)

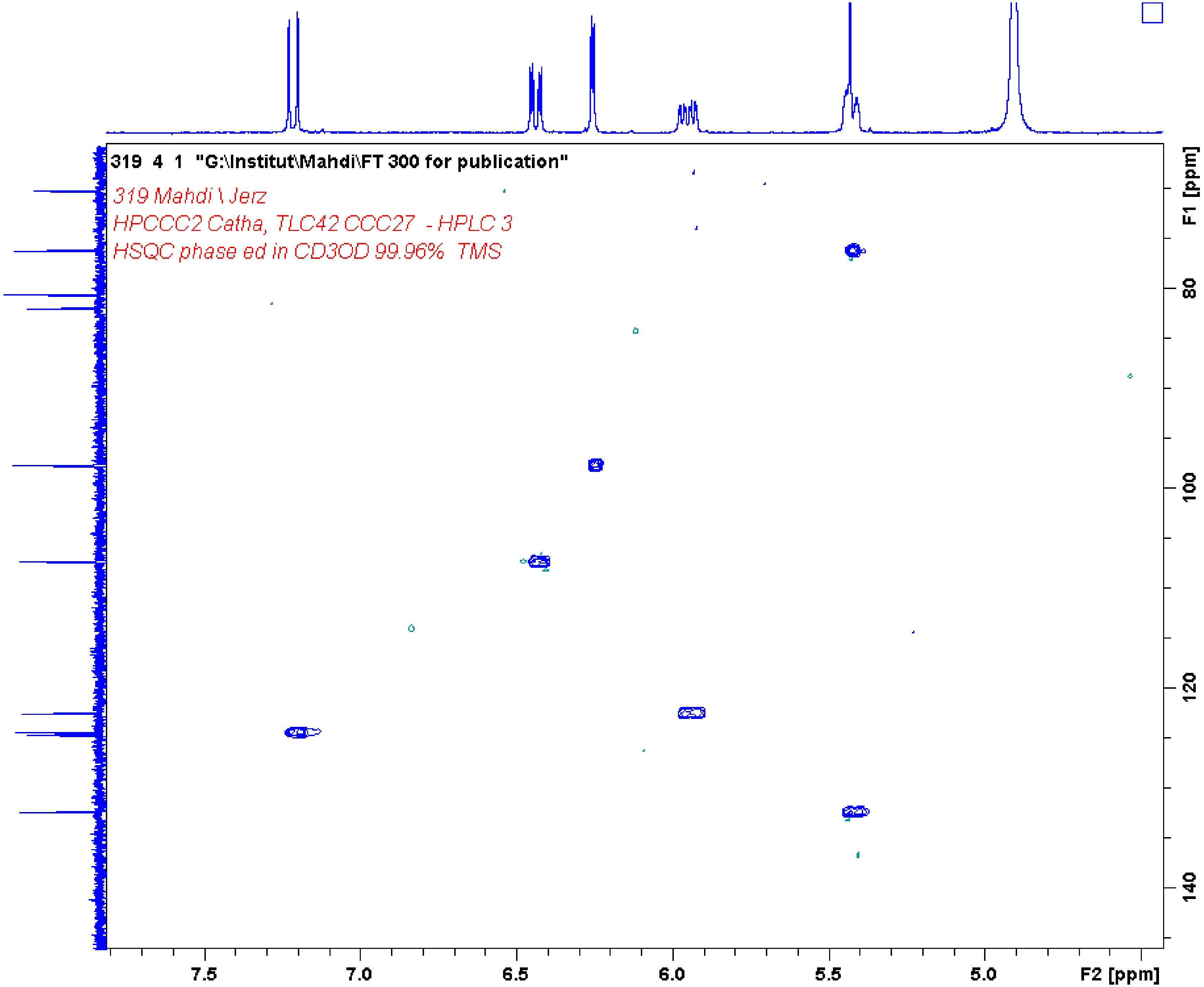

Figure NMR-S9

HSQC phase edited ^{1}J-*HC*

Vindoline (*457*)
in CD_3OD
(300 MHz)

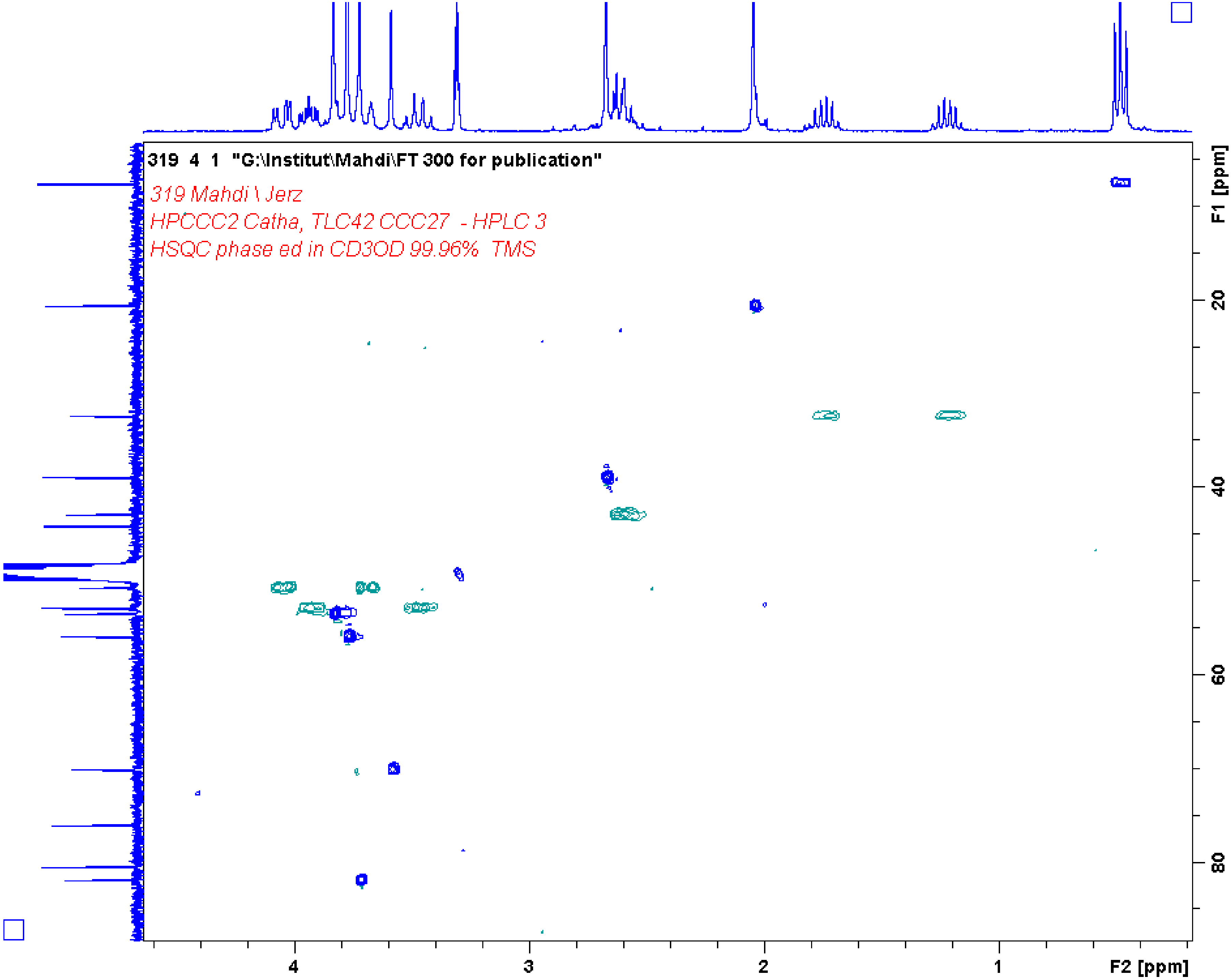

Figure NMR-S9

HMBC, long-range $^{2,3}J$-HC

Vindoline (*457*)
in CD_3OD
(300 MHz)

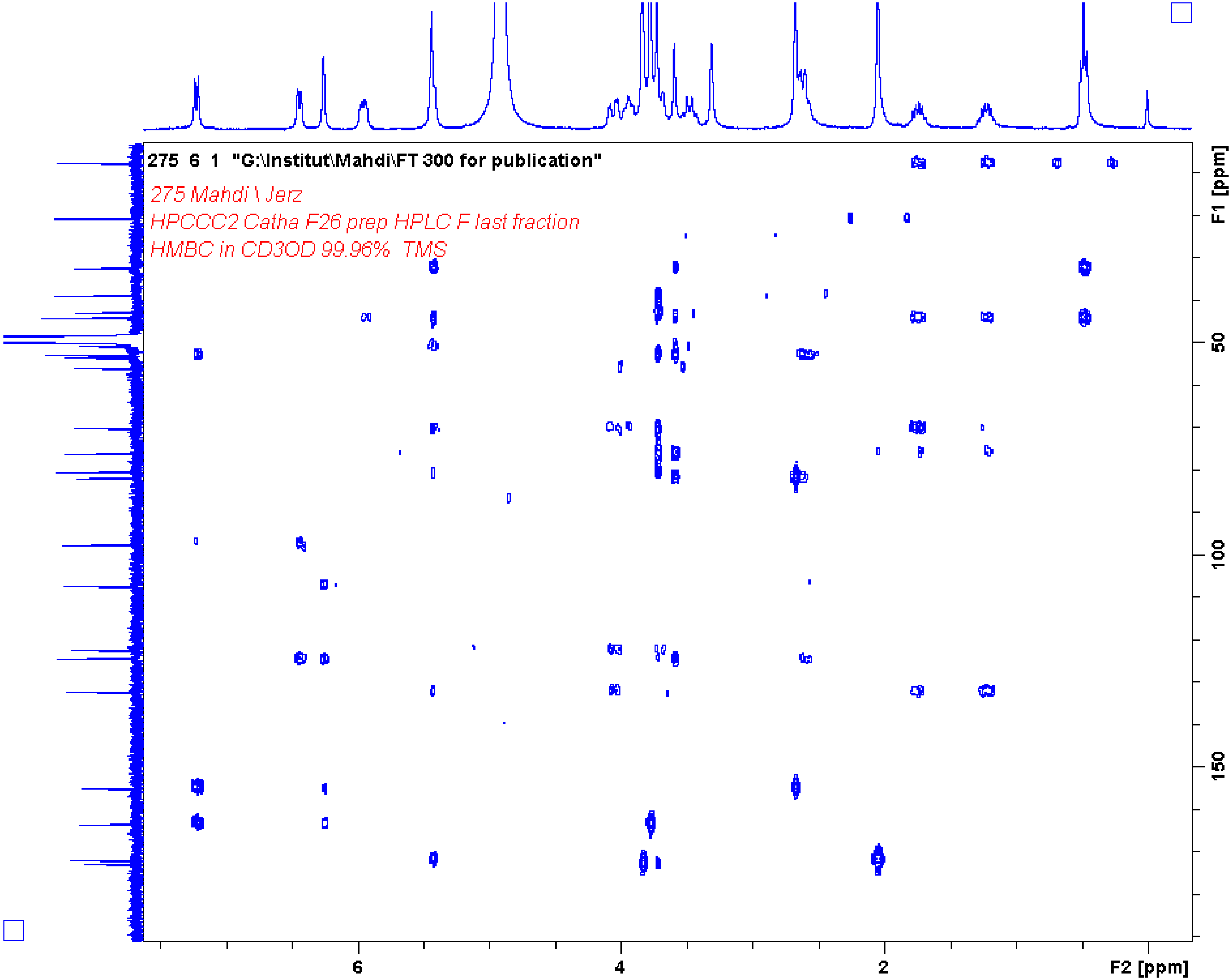

Figure NMR-S9

HMBC, long-range $^{2,3}J$-HC

Vindoline (*457*)
in CD_3OD
(300 MHz)

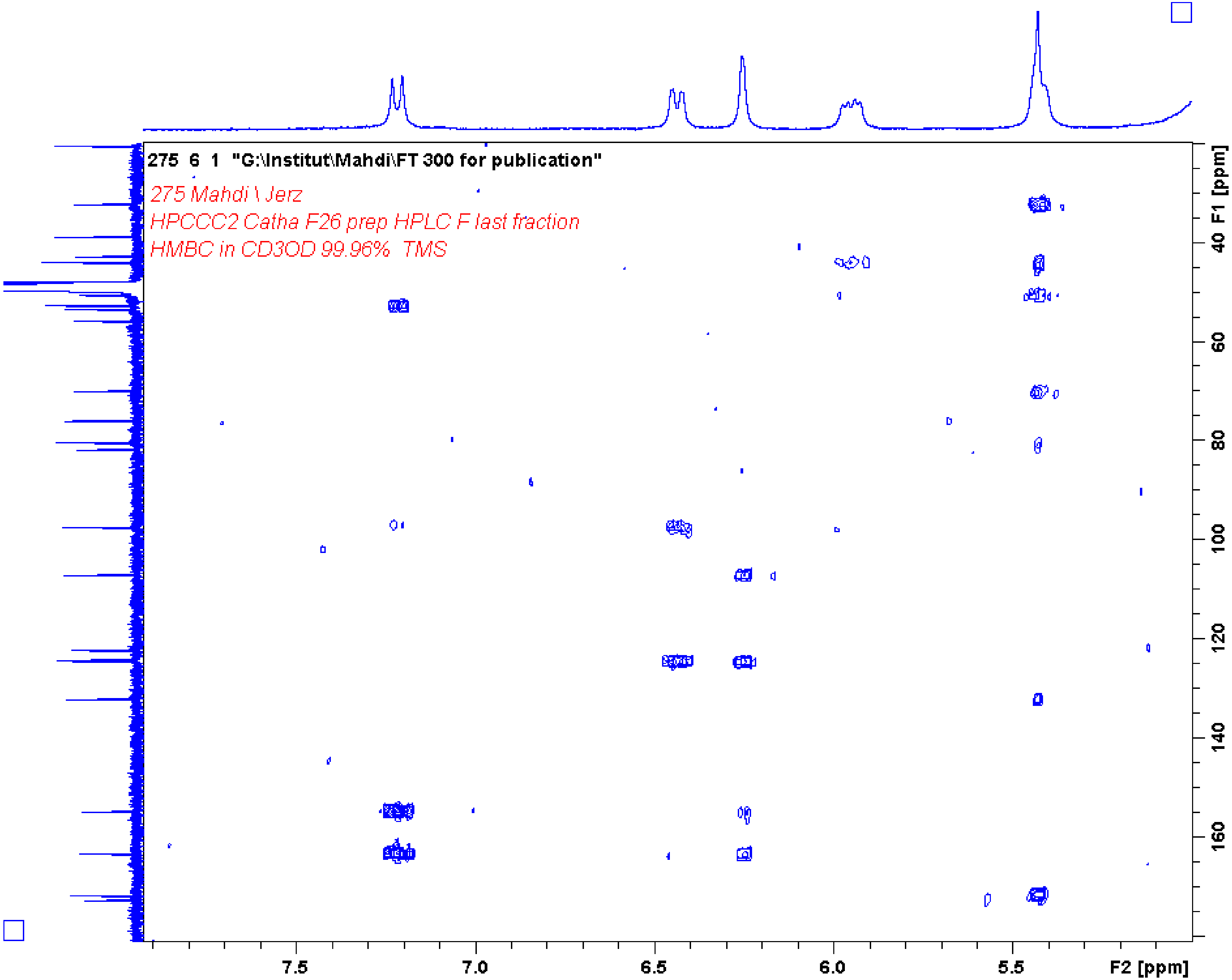

Figure NMR-S9

HMBC, long-range $^{2,3}J$-*HC*

Vindoline (*457*)
in CD_3OD
(300 MHz)

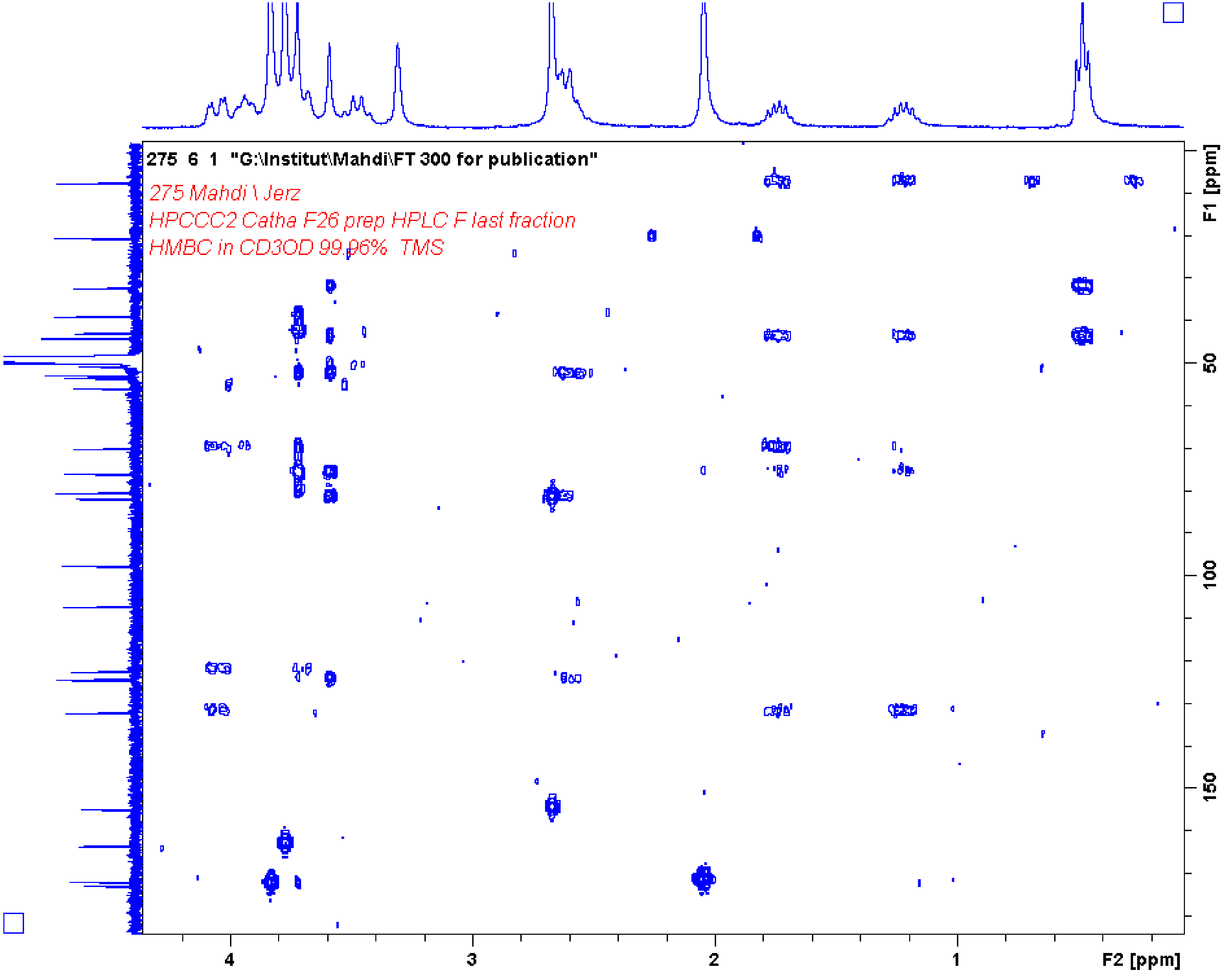

Figure NMR-S9

$^1H/^1H$-NOESY

Vindoline (*457*)
in CD_3OD
(300 MHz)

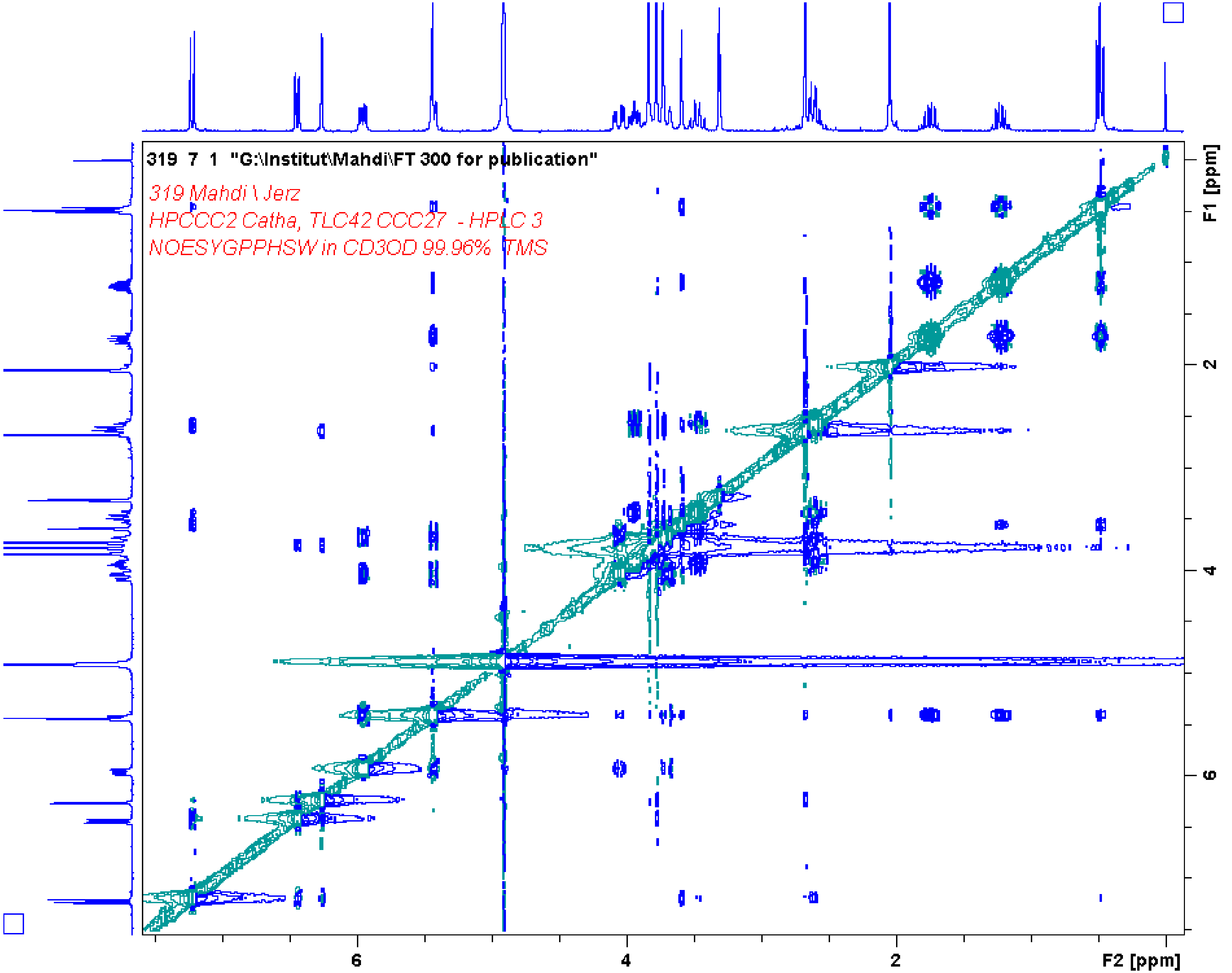

Figure NMR-S9

$^{1}H/^{1}H$-NOESY

Vindoline (*457*)
in CD_3OD
(300 MHz)

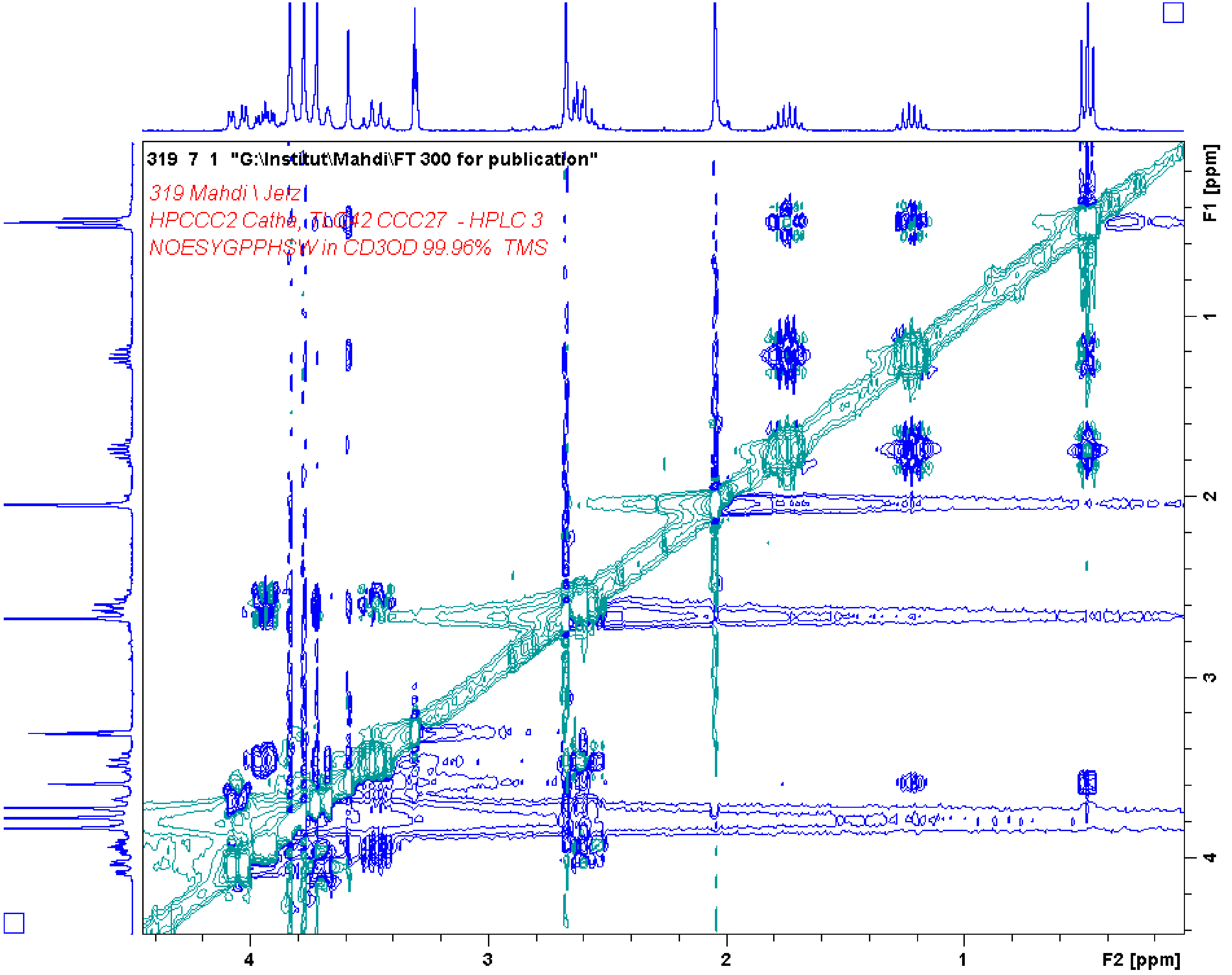

Figure NMR-S9

$^1H/^1H$-NOESY

Vindoline (*457*)
in CD_3OD
(300 MHz)

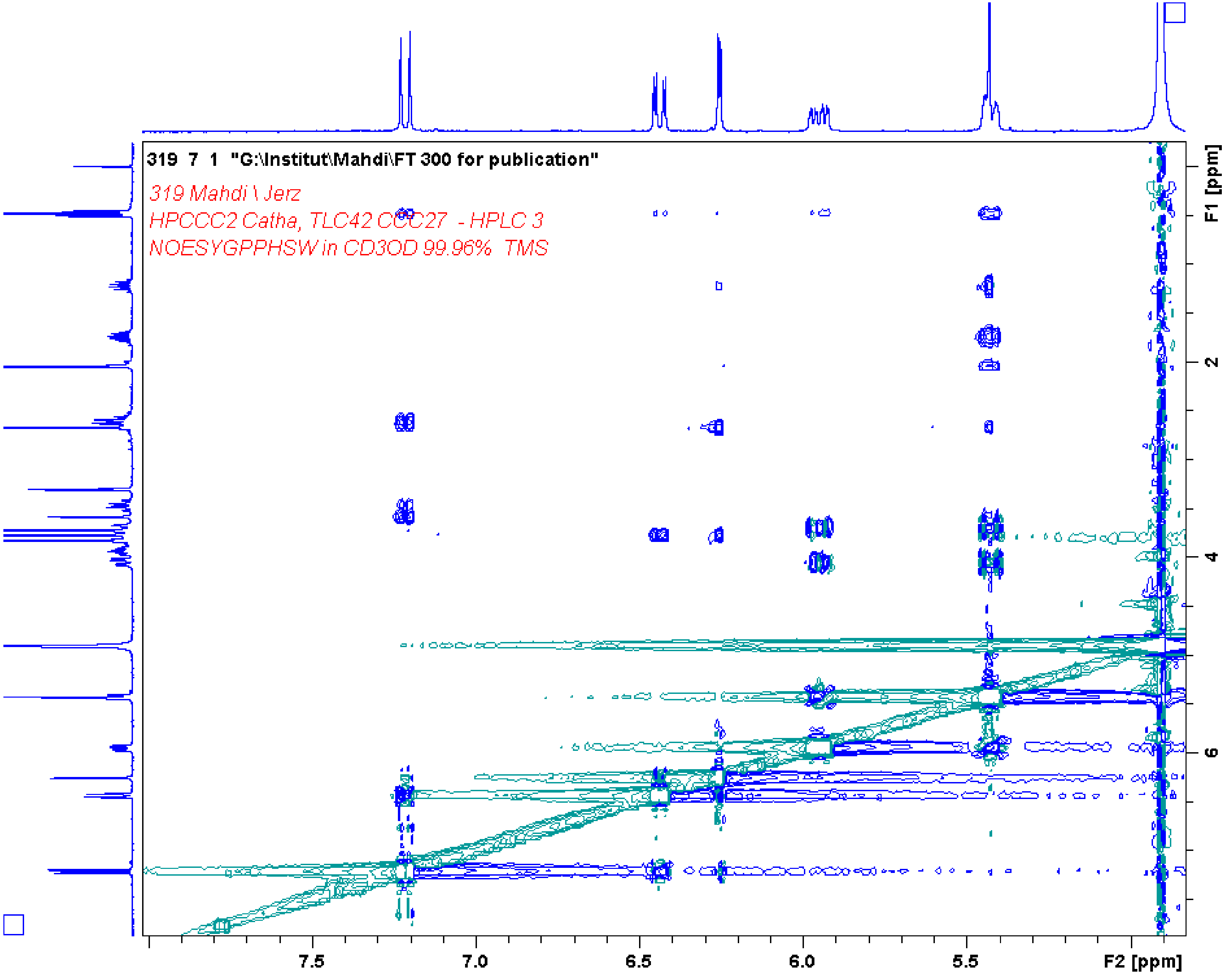

Supplement: Supplementary file 1 [file molecules-30-02115-s001.zip › Supplement Figures S2-S9.pdf]
