# Supplementary material for: Electrospray–Mass Spectrometry-Guided Targeted Isolation of Indole Alkaloids from Leaves of Catharanthus roseus by Using High-Performance Countercurrent Chromatography
Source: Molecules. 2025 May 9;30(10):2115. doi: 10.3390/molecules30102115 (PMC12113773; doi:10.3390/molecules30102115)
Supplement: Supplementary file 1 [file molecules-30-02115-s001.zip › Supplement Table S1.pdf]

Supplement Table 1: 1D/2D-NMR data

Electrospray-Mass-Spectrometry Guided Recovery Profiling of Indole Alkaloids from Leaves of *Catharanthus roseus* by High-Performance Countercurrent Chromatography

Mahdi Yahyazadeh, Dirk Selmar, and Gerold Jerz *

All 1D/2D-NMR measurements ^1H: at 300 MHz and ^{13}C: 75 MHz

Original spectral data cf. Supplement Figures 1 - NMR SP1 – SP8.

Catharanthine (337-*f*): in $CDCl_3$ calibrated to TMS (^{1}H) and to solvent signal (^{13}C) δ 77.26 [ppm], *J* [Hz].

Pos.	^{1}H	^{13}C	DEPT 135	HMBC	COSY	NOESY
2	-	133.27	q	-	-	-
3a	3.51 dt_{br}, J_1 3.1, J_2 10.5	52.51	CH_2	C-5, 14, 17	H-3b	H-3b, 14, 15
3b	2.87 d_{br} * *J* 10.5				H-3a	H-3a, 6b
5a	4.07 *dd*, J_1 9.5, J_2 10.5	56.23	CH_2	C-3, 6, 7, 21	H-6a, 5b	H-5b, 21
5b	3.43 **				H-5a	H-5a
6a	3.41 **	19.47	CH_2	C-2, 5, 7, 8	H-5b	H-6b, 10, 21
6b	3.15 *dd*, J_1 8.0, J_2 13.0				H-5a	H-3b, 6a, 10
7	-	110.06	q	-	-	-
8	-	127.63	q	-	-	-
9	7.49 d_{br}, *J* 7.8	118.45	CH	C-7, 11, 13	H-10	H-6a/b, 10
10	7.16 *dt*, J_1 1.5, J_2 7.7	120.55	CH	C-8, 12	H-9	H-9
11	7.23 *dt*, J_1 1.2, J_2 7.0	123.41	CH	C-9, 13	n.res.	n.res.
12	7.31 *d*, *J* 6.7	111.26	CH	C-8, 10	n.res.	n. res.
13	-	135.45	q	-	-	-
14	3.01 m_{br}	29.22	CH	C-15, 16	H-15	H-3a, 15, 17a/b
15	6.21 *dd*, J_1 6.7, J_2 1.3	127.73	CH	C-3 (weak), 14, 19, 21	H-14, 19a/b, 21	$C\underline{H}_3$-18, H-3a, 14, 19a/b
16	-	51.02	q	-	-	-
17a	1.93 *dd*, J_1 13.4, J_2 1.8	37.07	CH_2	C-2, 3, 14, 15, -$\underline{C}OOCH_3$	H-17a	H-14, 17b, >N-$\underline{H}$
17b	2.85 *				H-17b	H-14, 17-a, >N-$\underline{H}$

18	1.07 *t*, *J* 7.2	10.35	CH_3	C-19, 20	H-19a/b	H-15, 19a/b, 21, -COOCH_3
19a	2.10 *qdd*, J_1 18.0, J_2 7.1, J_3 1.8	26.28	CH_2	C-15, 18, 21	H-18, 19b	H-15, 18, 21
19b	2.48 *qdd*, J_1 18.0, J_2 6.8, J_3 2.1				H-18, 19a	H-15, 18, 21
20	-	145.74	q	-	-	-
21	4.88 *d*, *J* 1.1	59.64	CH	C-2, 5, 15, 16, 17, 19, 20	H-15	>N-H, H-5a, 6a, 18, 19a/b, -COOCH_3
>N-H-1	8.11 *s*	-	-	C-2, 8, 12, 13	n.det.	H-12, 17a/b, 21, -COOCH_3
-COOCH_3	3.75 *s*	53.58	CH_3	-COOCH_3	-	H-21, CH_3-18
-COOCH_3	-	171.75	q			-

*/**signal overlappings

Akuammicine (*323-j*): in $CDCl_3$ calibrated to TMS (^{1}H) δ0.00 [ppm], and to solvent signal (^{13}C) δ 77.26 [ppm], *J* [Hz].

Pos.	^{1}H	^{13}C	DEPT 135	HMBC	COSY	NOESY
2	-	164.0	q	-	-	-
3	4.71 s_{br}	61.5	CH	n.d.	H-14b	H-14a/14b, 22-OCH_3, 5b
5a	3.31 *dd*, J_1 12.0, J_2 6.5	54.1	CH_2	C-21	H-5b	22-OCH_3, H-6a, 5b
5b	3.85 *m*				H-5a, 6b	H-3, 5a, 9
6a	2.19 *dd*, J_1 13.0, J_2 6.0	43.2	CH_2	C-3, 5, 8	H-6b, 5a, 5b	H-5a, 22-OCH_3
6b	2.68 *dt*, J_1 13.5, J_2 6.5			C-2, 8	H-5b, 6a	H-9
7	-	55.0	q	-	-	-
8	-	133.2	q	-	-	-
9	7.39 *d*, *J* 7.5	121.4	CH	C-7, 11, 13	H-10	H-3, 10, 5b
10	6.98 *t*, J_1 7.5 J_2 <1 Hz	122.3	CH	C-8,12	H-9, 11	H-9
11	7.24 *dt*, *J1* 8.0, *J2* 0.8	129.6	CH	C-10, 13	H-10, 12	-
12	6.87 *d*, *J* 7.5	110.5	CH	C-10, 8	H-11	NH-1
13	-	143.1	q	-	-	
14a	2.59 *dm*, *J* 15.0	29.3	CH_2	n.d	H-14b, 15	H-3, 15, 21b, 14b
14b	1.51 *dt*, J_1 15.0, J_2 3.0				H-3, 14a	H-3, 15, 14a
15	4.11 s_{br}	28.5	CH	n.d.	H-14a, 19	H-14a/14b, 19, CH3-18
16	-	102.3	q	-	-	-
17	-	167.2	q	-	-	-

18	1.71 *d*, *J* 7.0	13.8	CH_3	H-19, 20	H-19	H-19, 15, 22-OCH_3
19	5.72 *q*, *J* 7.0	129.8	CH	H-15, 21	H-18	C<u>H</u>3-18, H-15, H-21a
20	-	130.9	q	-	-	-
21a	3.37 *d*, *J* 15.0	55.1	CH_2	H-3, 5, 20	H-21b	H-19, 21b
21b	4.37 *d*, *J* 15.0				H-21a	H-14a, 21a
22-OCH_3	3.84 *s*	51.8	CH_3	C-17	-	H-5a, 6a C<u>H</u>3-18,
N<u>H</u>-1	8.97 *s*	-	-	C-7, 8, 13	-	H-12, O-CH_3

Perivine (*339-a*): in CD_3OD calibrated to TMS (^{1}H) δ 0.00 [ppm], and solvent signal (^{13}C) δ 49.0 [ppm] (^{13}C), *J* [Hz].

Pos.	^{1}H	^{13}C	DEPT 135	HMBC	COSY	NOESY
2	-	135.6	q	-	-	
3	-	190.9	q	-	-	
5	2.90 *t*, *J* 3.2	45.7	CH	C-6, 16, 17, 21	H-6, 16	H-16, 6a
6a	3.89 *m*	23.8	CH_2	C-2, 7, 8, 16		H-5
6b	3.76 *			C-5, 7, 16	H-16	H-9
7	-	117.6	q	-	-	
8	-	138.7	q	-	-	
9	7.77 d_{br}, 8.0	121.5	CH	C-7, 8, 11	-	H-6a
10	7.35 *td*, J_1 6.5, J_2 1.0	127.9	CH	C-8, 9, 11	-	n.d.
11	7.16 *td*, J_1 7.5, J_2 1.0	121.7	CH	C-12, 13	-	H-12
12	7.44 d_{br}, 8.0	113.4	CH	C-11, 13	-	H-11
13	-	129.1	q	-	-	-
14a	3.55 *dd*, J_1 13.8, J_2 13.5	43.3	CH_2	C-3, 15, 20	H-15	H-16
14b	2.68**, J_1 ~14.0, J_2 7.4			C-2, 3, 15, 20	H-15	H-15
15	3.88 *m*	31.0	CH	C-16	H-14a/b	CH_3-18, H-14b
16	4.58 ***	51.9	CH	C-5, 15	H-5	H-14a, 5
17	-	169.9	q	-	-	
18	1.81 *s*	12.6	CH_3	C-19, 20	H-19	H-15, H-19
19	5.81 *qd*, J_1 6.6, J_2 1.2	127.7	CH	C-15, 18, 21	CH_3-18	CH_3-18, 21b
20	-	131.7	q	-	-	
21a	3.76 *	42.9	CH_2	C-15, 19, 20	H-21a	H-21b
21b	4.58 ***			C-5, 15, 20	H-21b	21a
-COOC<u>H</u>3	2.73 *s* **	51.6	CH_3	C-17	-	

*/**/*** signal overlappings

Vindorosine (*427*) (syn. demethyoxy-vindoline, vindolidine)**:** in CD_3OD calib. to TMS (^{1}H) δ0.00 [ppm], and solvent signal (^{13}C) δ49.0 [ppm] (^{13}C), *J* [Hz]:

Numbering related to Ishikawa et al 2006

**Pos.**	**^{1}H**	**^{13}C**	**DEPT 135**	**HMBC**	**NOESY**
N-1	-	-	-		
2	3.72 *s*	81.3	CH	C-3, 4, 12, 13, 19, -$\underline{C}OOCH_3$	H-11, >N-$C\underline{H}_3$
3	-	80.4	q	-	
4	5.44 * *s*	76.0	CH	C-3, 5*, 6, 19*, 20*, -$\underline{C}OOCH_3$	-$COCH_3$, H-20a/b, >N-$C\underline{H}_3$
5	-	44.1	q	-	
6	5.41* *d* 10	132.1	CH	C-5*, 8, 19*, 20*	H-7, H-21, H-20, -$CO\underline{CH}_3$
7	5.95 *ddd*, J_1 10.5, J_2 5, J_3 1.5	122.6	CH	C-5, 8	H-6, 8a/8b, -CH_3-21 -$COCH_3$,
8a	3.70 d_{br},16.0	50.7	CH_2	C-6,7, 10	H-7, 8b
8b	4.06 *ddd*, J_1 16, J_2 5, J_3 1				H-7, 8a
N-9	-	-	-	-	
10a	3.95 *dt*, J_1 9.0, J_2 1.5	53.0	CH_2	C-8, 11	H-10b, 11
10b	3.49 *q* 10.5				H-10a, 11, 14
11	2.64 *m*	43.1	CH_2	n.d.	H-10a
12	-	53.3	q	-	
13	-	132.8	q	-	
14	7.36 *dd*, J_1 7.5, J_2 1	123.9	CH	C-13, 18	H-10b, 11b, 15, 19, 20a/b, 21-CH_3,
15	6.88 *td, J_1* 8.0 J_2 1	121.7	CH	C-13	H-14, 16
16	7.23 *td*, J_1 7.5, J_2 1.5	131.7	CH	C-14, 18	H-17, H-15
17	6.70 *d*, *J* 8.5	111.4	CH	C-13	>N-$C\underline{H}_3$, H-16
18	-	153.6	q	-	
19	3.60, *s*	70.1	CH	C-5, 6, 8, 10, 20	H-14, 20a, 21
20a	1.18 *dd, J* 7.3 1.72 *m*	32.4	CH_2	C-5	H-6, 19, 20b, 21
20b					H-6, 20a, 21
21	0.43 *t, J* 7.0	7.55	CH_3	C-5, 20	H-14, 4, 6, 19, 20a/b
>N-$C\underline{H}_3$	2.68 *s*	39.05	CH_3	C-2, 18	H-2, 4 (weak), 17, -$COOC\underline{H}_3$
-$COOC\underline{H}_3$	3.83, *s*	53.4	CH_3	-$\underline{C}OOCH_3$	>N-$C\underline{H}_3$, H-4
-$\underline{C}OOCH_3$	-	172.9	q	-	
-$CO\underline{CH}_3$	2.04 *s*	20.6	CH_3	-$\underline{C}OCH_3$	H-4, 6
-$\underline{C}OCH_3$	-	172.0	q	-	

*signal overlapping

Vindorosine (*427*) (syn. demethyoxy-vindoline, vindolidine)**:** in C_6D_6 calib. TMS (^{1}H) δ: 0.00 [ppm], and solvent signal (^{13}C) δ 128.5 [ppm] (^{13}C), *J* [Hz].

Numbering related to Ishikawa et al 2006

**Pos.**	**^{1}H**	**^{13}C**	**DEPT 135**	**HMBC**
N-1	-	-	-	
2	3.73 *s*	80.6	CH	N-CH_3, C-3, 11, 12, 19, -<u>C</u>OOCH_3
3	-	79.35	q	-
4	5.80 *s*	75.6	CH	C-3, 5, 19, 20, -<u>C</u>OOCH_3
5	-	44.0	q	-
6	5.12 *d, J* 10	131.8	CH	C-5, 8, 19, 20
7	5.28 d_{br}, *J* 10	122.4	CH	C-5, 8
8a	2.39 m_{br}	51.25	CH_2	n.d.
8b	3.80 m_{br}			
N-9	-	-	-	-
10a	2.31 m_{br}	53.6	CH_2	C-12
10b	3.65 m_{br}			
11a	2.51 m_{br}	42.3	CH_2	n.d.
11b	2.07 m_{br}			
12	-	52.9*	q	-
13	-	133.4	q	-
14	6.71 *s*	122.8	CH	C-16, 18
15	6.72 *s*	120.8	CH	C-17
16	7.02 *m*	130.2	CH	C-14, 18
17	6.21 *d, J* 8.0	110.7	CH	C-13, 15
18	-	153.3	q	-
19	2.62 *s*	70.6	CH	C-2, 12, 5, 20
20a	1.43 *dt, J 7.2*	32.0	CH_2	C-4, 5, 6
20b	1.92 *dt, J 7.2*			
21	0.15 *t, J* 7.4	8.05	CH_3	C-5, 20
>N-<u>CH</u>$_3$	2.31 *s*	38.0	CH_3	C-2, 18
-COO<u>CH</u>$_3$	3.42 *s*	52.7*	CH_3	-<u>C</u>OOCH_3
-<u>C</u>OOCH_3	-	172.9	q	-
-CO<u>CH</u>$_3$	2.00 *s*	20.95	CH_3	-<u>C</u>OCH_3
-<u>C</u>OCH_3	-	171.11	q	-

* signals overlapped

Vindolinine (*337-b*): in CD_3OD calib. TMS (^1H) δ 0.00 [ppm], and solvent signal (^{13}C) δ: 49.0 [ppm] (^{13}C), *J* [Hz]:

Pos.	^1H	^{13}C	DEPT 135	HMBC	COSY	NOESY
NH-1	-	-	-	-		
2	-	82.8	q	-		
3a	4.31 *dd*, J_1 17.5, J_2 5.0	50.4	CH_2	C-14, 15, 21	H-14	H-3b, 14
3b	4.04*					H-3a, 14
N-4	-	-	-	-	-	-
5a	3.93 *m*	59.8 ***	CH_2	C-3, 6		H-5b, 6a
5b	4.05* *m*					H-5a, 6b, 17b
6a	2.44 *dd*, J_1 16.0, J_2 7.3	35.4	CH_2	C-2, 7, 21		H-5a, 6b
6b	1.96 ** *m*					H-9, 5b, 6a
7	-	60.3 ***	q	-	-	-
8	-	134.7	q	-	-	-
9	7.18 *d*, *J* 7.5	124.5	CH	C-7, 11, 13		H-6b, 10, 21
10	6.76 *t*, *J* 7.5	120.9	CH	C-8, 12		H-9
11	7.06 *t*, *J* 7.5	129.7	CH	C-9, 12, 13		
12	6.69 *d*, *J* 8.2	111.8	CH	C-8, 10		
13	-	150.9	q	-	-	-
14	5.90 *q*, *J* 3.8	124.2	CH	C-3	H-3a, 15	H-15, H3-3a/b
15	6.47 *dd*, J_1 9.5, J_2 2.9	134.3	CH	C-3, 21	H-14	CH_3-18, H-14,19
16	3.34 *d*, *J* 5.3	39.9	CH	C-2, 7, 17, -<u>C</u>OOCH₃	H-17a	CH_3-18, H-17a
17a	2.06 *m***	28.8	CH_2	C-19, 21, -<u>C</u>OOCH₃		H-17b, 21
17b	2.59 *ddd*, J_1 15.5, J_2 5.8, J_3 1.5				H-16	H-5a, 15, 17a, CH_3-18
18	1.11 *d*, *J* 6.5	6.9	CH_3	C-2, 19, 20	H-19	H-15, 16, 17b, 19
19	2.16 *q*, *J* 6.5	49.6	CH	C-2, 16, 17, 20	CH_3-18	H-15, 21, CH_3-18
20	-	45.8	q	-	-	-
21	3.82 *s*	76.7	CH	C-5***, 6, 7***, 15, 20		H-9, 19
-COO<u>CH</u>₃	3.73 *s*	52.7	CH_3	-<u>C</u>OOCH₃		n.det.
-<u>C</u>OOCH₃	-	175.2	q	-	-	-

*/** Signal overlappings *** potential signal overlapping in HMBC

***19R*-Vindolinine (*337-b*):** in $CDCl_3$ calibrated to TMS (^1H) δ 0.00 [ppm], and to solvent signal (^{13}C), δ 77.26 [ppm], *J* [Hz]. Reference data Att-ur-Rahman 1983 Z. Naturforsch.

	**^{13}C**	***19R*-vidolinine $^+$**	**$\Delta\delta$ ppm**	**DEPT135**
NH-1	-	-	-	-
2	81.35	81.4	0.05	q
3	58.0	58.0	0.00	CH_2
N-4	-	-	-	-
5	49.5	50.3	0.80	CH_2
6	35.6	36.3	0.30	CH_2
7	59.8	59.8	0.00	q
8	n. det.	139.8	-	q
9	124.4	123.6	0.80	CH
10	121.8	121.0	0.80	CH
11	127.8	127.2	0.60	CH
12	112.7	112.0	0.70	CH
13	149.0	149.4	0.40	q
14	n. det.	128.5	-	CH
15	131.5	130.7	0.80	CH
16	39.1	39.2	0.10	CH
17	28.9	29.1	0.20	CH_2
18	7.3	7.4	0.10	CH_3
19	48.6	48.4	0.20	CH
20	45.5	46.2	0.70	q
21	n. det.	78.0		CH
-COO<u>CH</u>$_3$	52.3	51.8	0.50	CH_3
-<u>C</u>OOCH$_3$	174.1	174.2	0.10	q

Vindoline (*457*): in CD_3OD calib. TMS (^1H) δ 0.00 [ppm], and solvent signal (^{13}C), δ 49.0 [ppm] (^{13}C), *J* [Hz]:

Numbering: Ishikawa et al 2006.

**Pos.**	**^1H**	**^{13}C**	**DEPT 135**	**HMBC**	**COSY**	**NOESY**
N-1	-	-	-			
2	3.72 **, *s*	81.9	CH	C-3, 4, 11, 12, 19, N-CH_3, -<u>C</u>OOCH$_3$		H-11, >N-C<u>H</u>$_3$
3	-	80.5	q	-		
4	5.43 *, *s*	76.0	CH	C-3, 5, 6, 19, 20, -<u>C</u>OOCH$_3$		H-20B!, -COCH$_3$, >N-<u>CH</u>$_3$
5	-	44.1	q	-		
6	5.42 *, *dq, J_1 10.2, J_2 1.3*	132.3	CH	C-5, 8, 19, 20	H-7	H-7, 21, 20a/b, -CO<u>CH</u>$_3$

7	5.95, *ddd*, J_1 10.2, J_2 5.0, J_3 1.5	122.4	CH	C-5	H-6, 8b	H-6, $-COCH_3$, 8a/8b
8a	3.70 ** d_{br},16.0	50.7	CH_2	C-6,7, 19	H-8b	H-7, 8b
8b	4.05 *ddd*, J_1 16.0, J_2 5.0, J_3 1.3				H-8a, 7	H-7, 8a
N-9	-	-	-	-	-	-
10a	3.93 *ddd*, J_1 11.3, J_2 8.5, J_3 3.5	52.85	CH_2	C-8, 11		H-10b, 11
10b	3.47, *q* 10.3				H-11	H-10a, 11, 14
11	2.61, *m*	42.9	CH_2	C-13	H-10b	H-2, H-10b, 14
12	-	52.78	q	-	-	-
13	-	124.6	q	-	-	-
14	7.21, *d*, *J* 7.5	124.3	CH	C-12, 16, 18		H-10b, 11b, 14, 15, 19, $21-C\underline{H}_3$
15	6.43, *dd*, J_1 8.0, J_2 2.2	107.2	CH	C-13, 14, 17		H-14, $16-OCH_3$
16	-	163.6	q			
17	6.25 *d*, *J* 2.2	97.6	CH	C-13, 15, 16, 18		$16-OCH_3$, $-COOC\underline{H}_3$ $>N-C\underline{H}_3$
18	-	155.0	q	-		
19	3.58, *s*	70.1	CH	C-2, 4, 5, 12, 13, 20		H-14, H-21, H-20a, H-4
20a	1.22 *qd*, *J* 7.5 1.74 *dd*, *J* 7.5	32.4	CH_2	C-4, 5, 19	CH_3-21	H-6, 20b, 21
20b					CH_3-21	H-6, 20a, 21
21	0.48 *t*, *J* 7.4	7.59	CH_3	C-5, 20	H-20a/b	H-14, 6, 19, 20a/b
$>N-C\underline{H}_3$	2.67, *s*	38.9	CH_3	C-2, 18		H-2, 4 (weak), 17, $-COOC\underline{H}_3$
$-COOC\underline{H}_3$	3.82, *s*	53.5	CH_3	$-\underline{C}OOCH_3$		$>N-C\underline{H}_3$, H-4, H-17
$-\underline{C}OOCH_3$	-	172.9	q	-		
$-COC\underline{H}_3$	2.04, *s*	20.6	CH_3	$-\underline{C}OCH_3$		H-4, 6
$-\underline{C}OCH_3$	-	172.0	q	-		
$16-OCH_3$	3.77**, *s*	55.9	CH_3	C-16		H-15, 17

*/**signal overlappings
