# Supplementary figures and images for: Electrospray–Mass Spectrometry-Guided Targeted Isolation of Indole Alkaloids from Leaves of Catharanthus roseus by Using High-Performance Countercurrent Chromatography
Source: Molecules. 2025 May 9;30(10):2115. doi: 10.3390/molecules30102115 (PMC12113773; doi:10.3390/molecules30102115)

# HPCCC Elution

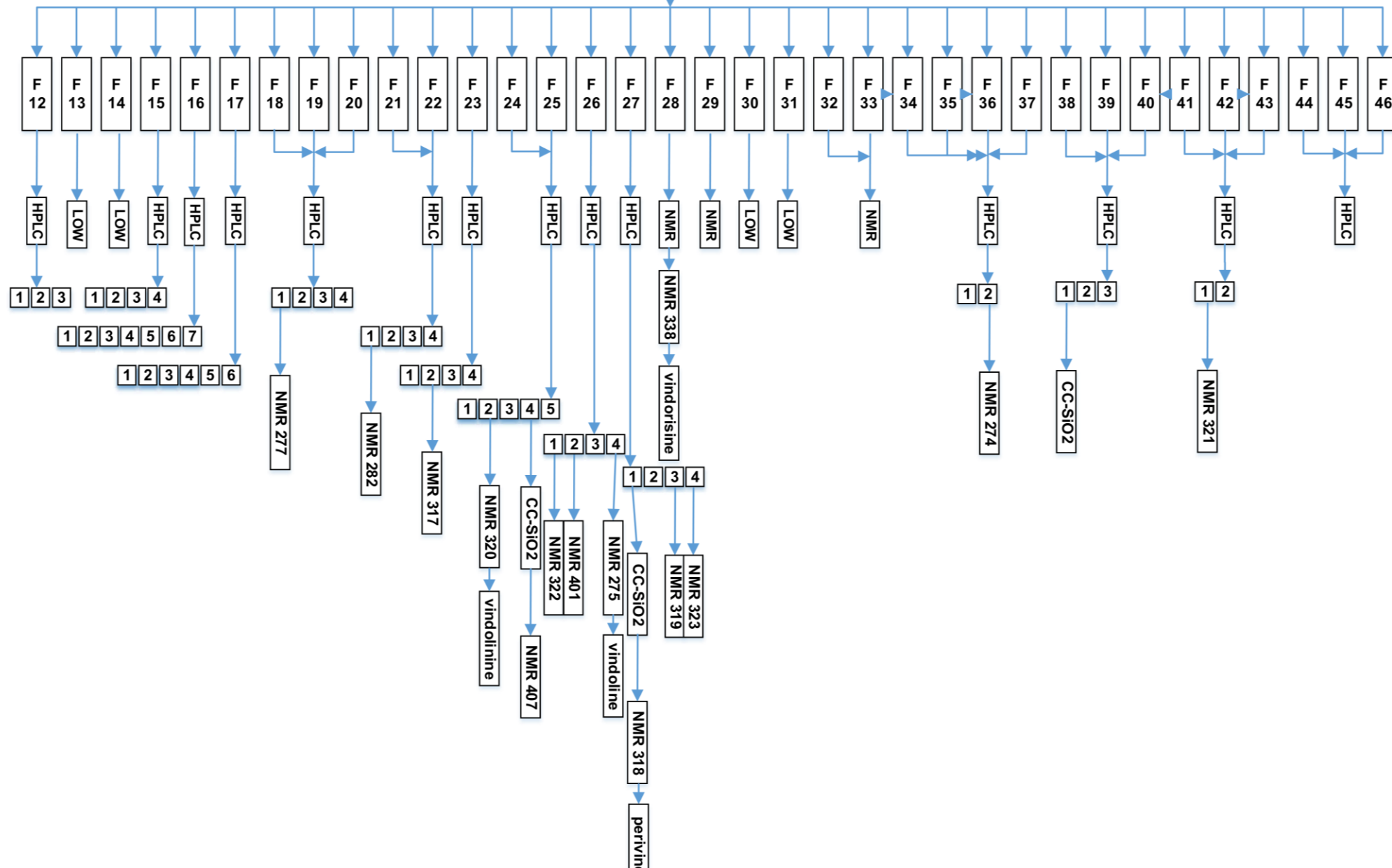

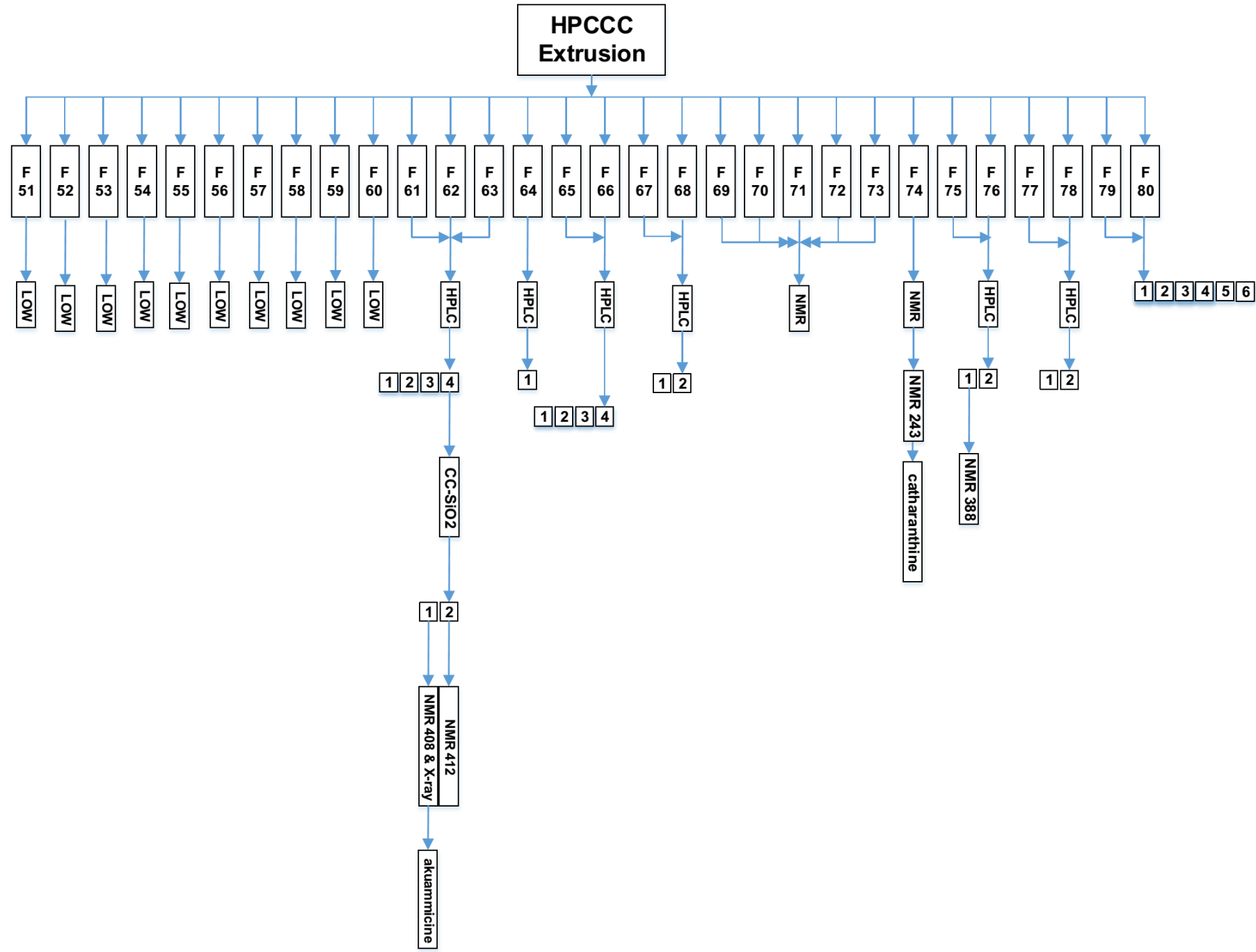

Supplement: Supplementary file 1 [file molecules-30-02115-s001.zip › Supplement Figure S10.pdf]
